# Mechanistic models of signaling pathways deconvolute the glioblastoma single-cell functional landscape

**DOI:** 10.1093/narcan/zcaa011

**Published:** 2020-06-25

**Authors:** Matías M Falco, María Peña-Chilet, Carlos Loucera, Marta R Hidalgo, Joaquín Dopazo

**Affiliations:** Clinical Bioinformatics Area, Fundación Progreso y Salud (FPS), Hospital Virgen del Rocío, 41013 Sevilla, Spain; Bioinformatics in Rare Diseases (BiER), Centro de Investigaciones Biomédicas en Red en Enfermedades Raras (CIBERER), 41013 Sevilla, Spain; Clinical Bioinformatics Area, Fundación Progreso y Salud (FPS), Hospital Virgen del Rocío, 41013 Sevilla, Spain; Bioinformatics in Rare Diseases (BiER), Centro de Investigaciones Biomédicas en Red en Enfermedades Raras (CIBERER), 41013 Sevilla, Spain; Clinical Bioinformatics Area, Fundación Progreso y Salud (FPS), Hospital Virgen del Rocío, 41013 Sevilla, Spain; Unidad de Bioinformática y Bioestadística, Centro de Investigación Príncipe Felipe (CIPF), 46012 Valencia, Spain; Clinical Bioinformatics Area, Fundación Progreso y Salud (FPS), Hospital Virgen del Rocío, 41013 Sevilla, Spain; Bioinformatics in Rare Diseases (BiER), Centro de Investigaciones Biomédicas en Red en Enfermedades Raras (CIBERER), 41013 Sevilla, Spain; Functional Genomics Node, FPS/ELIXIR-ES, Hospital Virgen del Rocío, 41013 Sevilla, Spain; Computational Systems Medicine Group, Institute of Biomedicine of Seville (IBIS), Hospital Virgen del Rocío, 41013 Sevilla, Spain

## Abstract

Single-cell RNA sequencing is revealing an unexpectedly large degree of heterogeneity in gene expression levels across cell populations. However, little is known on the functional consequences of this heterogeneity and the contribution of individual cell fate decisions to the collective behavior of the tissues these cells are part of. Here, we use mechanistic modeling of signaling circuits, which reveals a complex functional landscape at single-cell level. Different clusters of neoplastic glioblastoma cells have been defined according to their differences in signaling circuit activity profiles triggering specific cancer hallmarks, which suggest different functional strategies with distinct degrees of aggressiveness. Moreover, mechanistic modeling of effects of targeted drug inhibitions at single-cell level revealed, how in some cells, the substitution of VEGFA, the target of bevacizumab, by other expressed proteins, like PDGFD, KITLG and FGF2, keeps the *VEGF* pathway active, insensitive to the VEGFA inhibition by the drug. Here, we describe for the first time mechanisms that individual cells use to avoid the effect of a targeted therapy, providing an explanation for the innate resistance to the treatment displayed by some cells. Our results suggest that mechanistic modeling could become an important asset for the definition of personalized therapeutic interventions.

## INTRODUCTION

Since the beginning of the century, transcriptomic technologies, which evolved from microarrays (1) to RNA sequencing (RNA-seq) (2), have provided an increasingly accurate insight into mRNA expression (3). The technological advances of RNA-seq technologies have increased the resolution in the quantification of transcripts until the unprecedented level of the mRNA component of individual single cells. The possibility of studying gene expression at the single-cell level opens the door to novel biological questions that were not possible using current tissue-level RNA-seq approaches. For example, single-cell RNA-seq (scRNA-seq) has allowed a high-resolution analysis of developmental trajectories (4,5), the detailed characterization of tissues (6,7), the identification of rare cell types (8) or the analysis of stochastic gene expression and transcriptional kinetics (9,10), just to cite a few cases.

The continuous publication of scRNA-seq studies is producing an increasingly large wealth of data on cell-level gene activity measurements under countless conditions. However, the functional consequences of such gene activity at single-cell level remains mostly unknown. Among the many methods and applications published for the management of scRNA-seq data (11), only a small proportion of them provide some functional insights on the results. For example, MetaNeighbor (12), SCDE (13) or PAGODA (14) annotates cell types based on conventional gene set enrichment analysis (15,16). Other algorithms, such as SCENIC (17), PIDC (18), SCODE (19) or SINCERITIES (20), offer the possibility of inferring regulatory networks as well. However, functional profiling methods have evolved from the analysis of simple gene sets or inferred regulatory gene networks to more sophisticated computational systems biology approaches that allow a mechanistic understanding on how molecular cell signaling networks enable cells to make cell fate decisions that ultimately define a healthy tissue or organ, and how deregulation of these signaling networks leads to pathological conditions (21–23). Specifically, mechanistic models have helped to understand the disease mechanisms behind different cancers (24–27), rare diseases (28,29), the mechanisms of action of drugs (29,30) and other physiologically interesting scenarios such as obesity (31) or the postmortem cell behavior of a tissue (32). Although there are several mechanistic modeling algorithms available that model different aspects of signaling pathway activity, Hipathia (24) has been demonstrated to outperform other competing algorithms in terms of sensitivity and specificity (23).

Here, we propose the use of mechanistic models of signaling activities (24,33) that trigger cell functionalities related with cancer hallmarks (34), as well as other cancer-related relevant cellular functions to understand the consequences of gene expression profiles on cell functionality at single-cell level. Such mechanistic models use gene expression data to produce an estimation of activity profiles of signaling circuits defined within pathways (24,33). An additional advantage of mechanistic models is that they can be used not only to understand molecular mechanisms of disease or of drug action but also to predict the potential consequences of gene perturbations over the circuit activity in a given condition (35). Actually, in a recent work, our group has successfully predicted therapeutic targets in cancer cell lines with a precision of over 60% (25).

An interesting model to be studied from the viewpoint of mechanistic models is glioblastoma, the most common and aggressive of gliomas (36). The current treatment for glioblastoma includes maximal safe surgical resection followed by radiotherapy and chemotherapy (37), often combined with other drugs such as bevacizumab in an attempt to overcome resistances (38). Despite this intense treatment, the mean survival of glioblastoma patients is only 15 months and resistances to the therapy are quite common (39–41). This high rate of treatment failure has been attributed to the lack of specific therapies for individual tumor types (42,43). Moreover, it is well known that glioblastoma tumors with a common morphological diagnosis display a high heterogeneity at the genomic level (44).

The availability of glioblastoma single-cell gene expression data (45) provides a unique opportunity to understand the behavior of a cancer type at the cell level. Here, we show for the first time how mechanistic models applied at single-cell level provide an unprecedentedly detailed dissection of the tumor into functional profiles at the scale of individual cells that throw new light on how cells ultimately determine its behavior. Moreover, since mechanistic models allow simulating interventions on the system studied, we show a comprehensive simulation of the potential effect of drugs at single-cell level that discloses, for the first time, the mechanisms and strategies used by subpopulations of cells to evade the effect of the drug.

## MATERIALS AND METHODS

### Data

A large scRNA-seq dataset containing 3589 cells of different types obtained in four patients from a glioblastoma study (45) was downloaded from GEO (GSE84465). Cells corresponded to the tumor, and to the periphery of the tumor.

### Data imputation and primary processing

Count values for the scRNA-seq were downloaded from GEO. Since many of these data are affected by dropout events (13), they were subjected to the three imputation methods, MAGIC (46), scImpute (47) and DrImpute (48), as implemented in the corresponding software packages. Each method has its own preprocessing pipeline explained in the corresponding publication. The Rand index (49), which represents the frequency of occurrence of agreements of elements in the same cluster with respect to the random expectation, was used as an objective criterion for clustering comparison.

Once imputed, samples were log transformed and a truncation by quantile 0.99 was applied. Finally, the values were normalized between 0 and 1, as required by the downstream functional analysis with Hipathia.

### Hipathia mechanistic model

The Hipathia method uses KEGG pathways (50) to define circuits that connect any possible receptor protein to specific effector proteins. Gene expression values are used in the context of these circuits to model signaling activity, which ultimately triggers specific cell activities, as described in (24). A total of 98 KEGG pathways involving a total of 3057 genes that form part of 4726 nodes were used to define a total of 1287 signaling circuits. The intensity value of signal transduced to the effector is estimated by the following recursive formula:

(1) $$\begin{equation*}{S_n} = {\upsilon _n} \cdot \left( {1 - \mathop \prod \limits_{{s_{\rm a}} \in A} \left( {1 - {s_{\rm a}}} \right)} \right) \cdot \mathop \prod \limits_{{s_{\rm i}} \in I} \left( {1 - {s_{\rm i}}} \right),\end{equation*}$$

where *S_n_* is the signal intensity for the current node *n*, *v_n_* is its normalized gene expression value, *A* is the set of activation signals (*s*_a_) arriving to the current node from activation edges and *I* is the set of inhibitory signals (*s*_i_) arriving to the node from inhibition edges (24).

The Hipathia algorithm (27) is implemented as an R package available in Bioconductor (https://bioconductor.org/packages/release/bioc/html/hipathia.html) as well as at a web server (http://hipathia.babelomics.org/) and as a Cytoscape application (http://apps.cytoscape.org/apps/cypathia).

### Differential signaling activity

Two groups of circuit activity profiles can be compared and the differences in activity of any circuit can be tested by means of different tests. Although non-parametric tests seem more adequate, and are suitable for small size studies, it has been noted that for larger sizes and, especially, when data display a highly skewed distribution, which is exactly this case, they tend to systematically give smaller *P*-values and parametric tests are preferable (51). In particular, limma (52), which has been demonstrated to be very efficient for gene expression data analysis, will be used.

### Signaling circuits associated with cancer hallmarks

Each effector is known to be associated with one or several cell functions. This information is extracted from both the UniProt (53) and Gene Ontology (54) annotations corresponding to the effector gene (24). However, in some cases, the annotations are too ambiguous or refer to roles of the gene in many different conditions, tissues, developmental stages, etc., thus making it difficult to understand its ultimate functional role. In addition, in this study the activity of signaling circuits relevant in cancer is particularly interesting. Since a number of these effector genes have been related specifically with one or several cancer hallmarks (34) in the scientific literature, the CHAT tool (55), a text mining-based application to organize and evaluate scientific literature on cancer, allows linking gene names with cancer hallmarks.

### Subtyping of cancer cells

The SubtypeME tool from the GlioVis data portal (56) was used to obtain the subtype of cancer (classical, proneural or mesenchymal), based on the signature of 50 genes (57). This tool provides three methods to assign subtype: single-sample gene set enrichment analysis, *K*-nearest neighbors and support vector machine. Subtype was assigned when at least two of the methods made an identical subtype prediction. The subtyping tools use gene data without imputation.

## RESULTS

### Selection of the optimal imputation method

Since mechanistic models consider the topology of signaling circuits to estimate signal transduction activity in the cell, the discrimination between genes with missing expression values and genes that are not expressed is crucial, given that, depending on the location of the gene within the circuit, it can play the role of a switch. Since dropout events (the observation of a gene at a moderate expression level in one cell that cannot be detected in another cell) are quite common in scRNA-seq experiments (13), and taking them as zero values can disturb the inferred activity of the circuit, the use of imputation methods is crucial for the application of the mechanistic model. Among the best performer imputation methods available (58), three of them were checked to decide which one is optimal in the context of signaling pathway activity inference: MAGIC (46), scImpute (47) and DrImpute (48).

**Figure 1.**
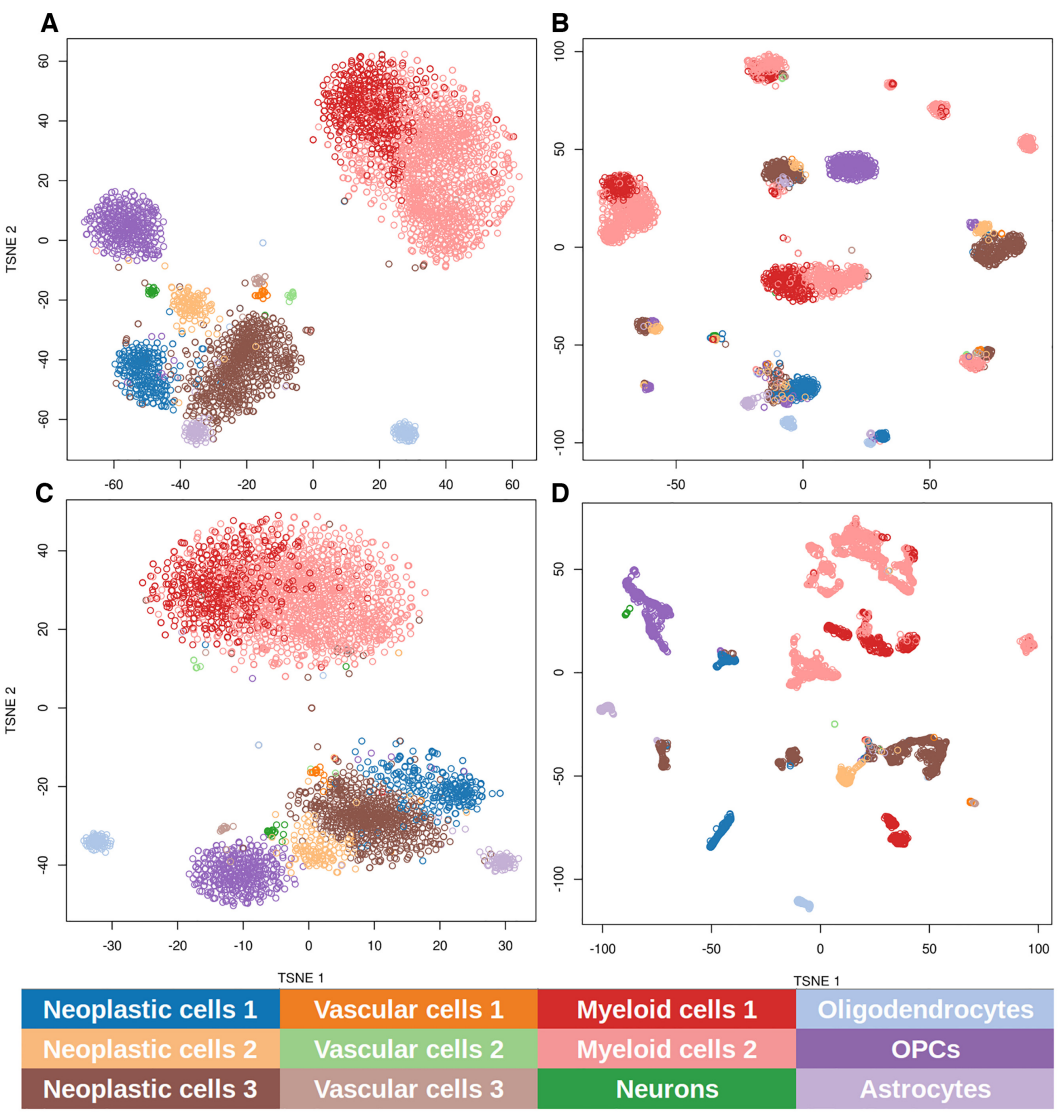
Clustering of the samples based on gene expression and signaling circuit activities obtained with different gene imputation methods. Data were subjected to t-SNE dimensionality reduction and the *k*-means clustering of the two main components is represented. (**A**) The clustering obtained with the gene expression values following the procedure described in the original glioblastoma study (45). Clustering obtained using all the circuit activities inferred using gene expression values imputed with (**B**) scImpute, which imputes 48% of the genes, (**C**) DrImpute, which imputes 85% of the genes, and (**D**) MAGIC, which makes the imputation over the whole set of genes. Cell types are labeled with colors.

**Figure 2.**
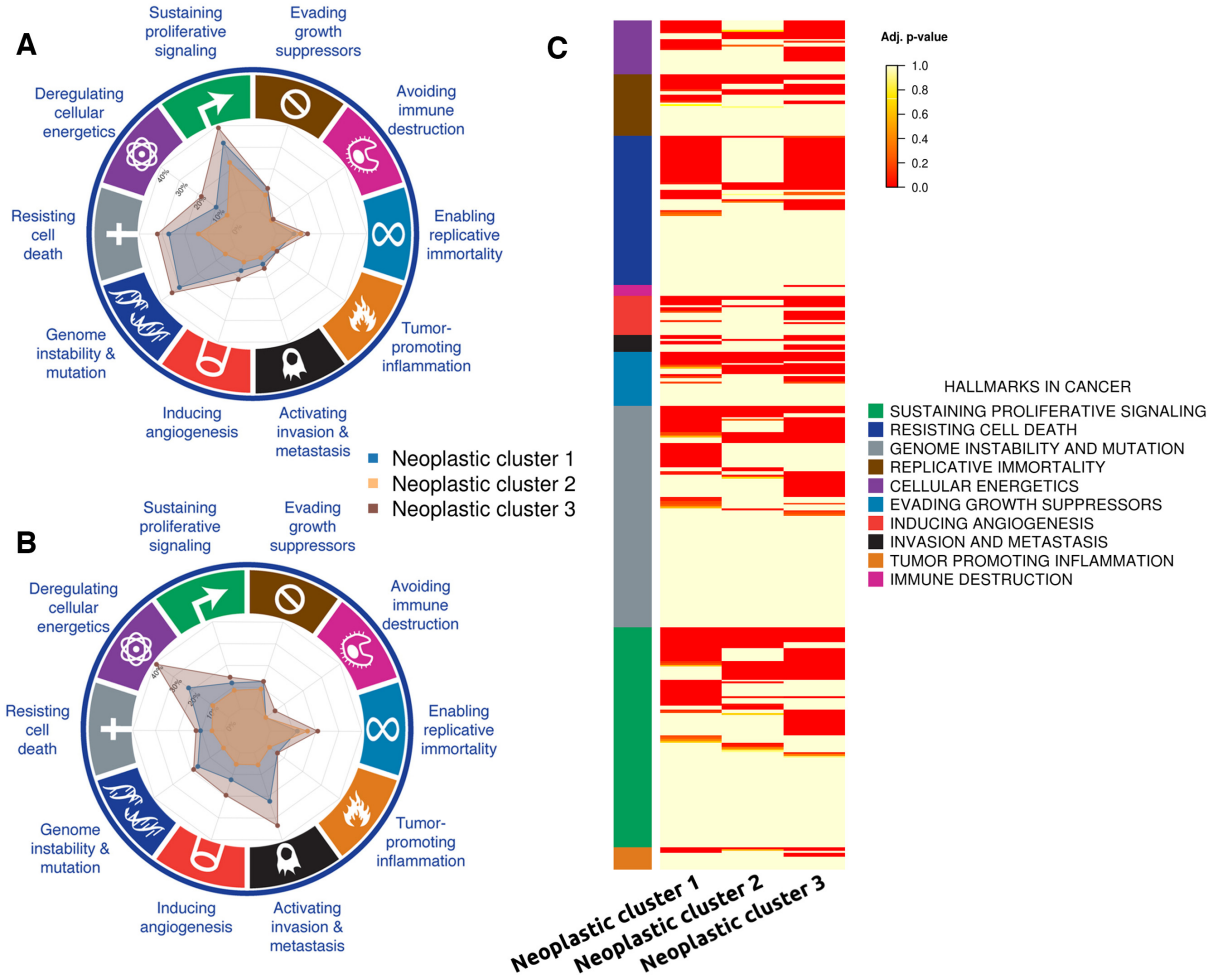
Circuits related to cancer hallmarks observed in the three neoplastic cell clusters. (**A**) Percentage of the total number of circuits with a significant differential activity in the neoplastic cells. The most internal division is 10% and every division increases a 10%. (**B**) Percentage of circuits with a differential activity with respect to the total number of circuits annotated to any of the cancer hallmarks. (**C**) Heat map with the signaling circuits related to the different cancer hallmarks that have been found to be differentially activated in cells of each neoplastic cluster.

In order to decide which imputation method produced the most realistic results, we used the clustering produced by the highly expressed genes in the original single-cell glioblastoma study (45) as ground truth. There, the authors applied t-SNE (59) over the 500 most variable and highly expressed genes and then clustered the resulting data with *k*-means. They found 12 main clusters with a homogeneous cell composition that was further experimentally validated, which were astrocytes, two myeloid cell clusters, three neoplastic cell clusters, neurons, oligodendrocytes, oligodendrocyte progenitor cells and three vascular cell clusters. Then, gene expression values were imputed using the above-mentioned methods (MAGIC, scImpute and DrImpute). Next, gene expression values were used to infer signaling circuit activities with the Hipathia algorithm (24) as implemented in the Bioconductor application (https://bioconductor.org/packages/release/bioc/html/hipathia.html). The values of circuit activity were subjected to the same procedure (t-SNE dimensionality reduction and *k*-means clustering) and the resulting clusters were compared to the original ones obtained in the glioblastoma study using the Rand index (49). Figure 1A shows the clustering obtained with the genes following the procedure described above [equivalent to Figure 2 of the original study (45)], which can be compared with the clustering of the samples using the circuit activities obtained with the gene expression values imputed with scImpute (Figure 1B), DrImpute (Figure 1C) and MAGIC (Figure 1D). The comparison of the clusters obtained with the three imputation methods was follows: scImpute, 0.745; DrImpute, 0.852; and MAGIC, 0.858. Although MAGIC rendered a slightly better Rand index, DrImpute was chosen as the imputation method because the dispersion of the clusters obtained was very similar to the one observed in the ground truth clustering (Figure 1A). The similarity in the clustering, which accounts for cell types, suggests that the imputation method is rendering values that result in coherent signaling circuit estimations.

### Functional characterization of cancer cells

Once verified that cell types defined by gene expression profiles (45) are supported by signaling profiles as well, the obvious comparison is the glioblastoma cell clusters versus the clusters composed by the different brain cells (oligodendrocytes, neurons, astrocytes and oligodendrocyte progenitor cells). It is interesting to note that normal cells, no matter which patient they were sampled from, display a similar functional profile; that is, the patients are intermingled within the clusters corresponding to any cell type. However, in the case of the neoplastic clusters, although some among-cluster overlap exists, their composition is mainly driven by the patient sampling origin (see Supplementary Figure S1). Since circuit activity bridges gene expression to signaling activity and ultimately cell functionality, an assessment of the differences between cell types from a functional perspective can be achieved by means of a differential cell activity statistical contrast. The cell functional responses triggered by the circuits differentially activated can be easily retrieved, and among them, those related with cancer hallmarks (34) can be identified using the CHAT tool (55), as explained in the ‘Materials and Methods’ section.

In order to detect which of the circuits display a significant change in activity, the three neoplastic cell clusters (1, 2 and 3 in Figure 1C) are compared to the normal brain cells (oligodendrocytes, oligodendrocyte precursor cells, astrocytes and neurons, labeled as O, OPC, A and N, respectively, in Figure 1C).

The comparison between the neoplastic clusters against the brain normal cells resulted in two different patterns of circuit activity: neoplastic clusters 1 and 3 present a higher number of signaling circuits differentially activated (309 and 336, respectively) than neoplastic cluster 2 (only 96 circuits; see Supplementary Table S1). Figure 2 represents the number of differentially activated signaling circuits involved in cancer hallmarks observed in the three neoplastic cell clusters. This representation provides a summary of the strategy used by any particular neoplastic cluster in terms of the number of signaling circuits that control cell functionalities identifiable as cancer hallmarks. Figure 2A depicts the absolute number of circuits with a significant differential activity in the neoplastic cells and Figure 2B depicts the same results but as percentages with respect to the total number of circuits annotated to any of the cancer hallmarks. Table 1 summarizes the number of signaling circuits related to cancer hallmarks common to the three clusters (first column) and specific for each cancer type (subsequent columns). The common functional signature of this cancer is clearly driven by circuits related to ‘Resisting cell death’, ‘Sustaining proliferative signaling’ and ‘Enabling replicative immortality’ hallmarks, completed with circuits related to ‘Evading growth suppressors’, ‘Inducing angiogenesis’ and ‘Tumor promoting inflammation’ hallmarks. From Table 1 it becomes apparent that neoplastic clusters 1 and 3 are using a functional strategy different from that used by neoplastic cluster 2. The first two display a functional signature compatible with a more aggressive behavior: they have many extra circuits related to ‘Resisting cell death’ and ‘Sustaining proliferative signaling’ hallmarks but, in addition, both clusters have circuit activity related to ‘Deregulation of cellular energetics’, ‘Genome instability and mutation’ and ‘Invasion and metastasis’ hallmarks (see Table 1 and Figure 2C for details). It is also interesting to note from Figure 2C that the individual circuits involved in triggering the same functions are not exactly the same across the neoplastic clusters (Supplementary Table S2 lists details of the circuits involved in the figure). Conversely, neoplastic cluster 2 does not seem to have much more extra functional activity beyond the common functional signature, which suggests a less aggressive character, especially because of the absence of circuit activity related to cell energetics or to invasion and metastasis.

**Table 1.** Summary of the different functional strategies followed by the different cells in the three neoplastic clusters in terms of the circuits differentially activated with respect to the normal tissue

Cancer hallmark	Common circuits	Neoplastic cluster 1	Neoplastic cluster 3	Neoplastic cluster 2
Resisting cell death	4	13	14	2
Sustaining proliferative signaling	8	14	14	2
Deregulating cellular energetics		4	8	
Genome instability and mutation		5	6	
Inducing angiogenesis	2	2	5	
Enabling replicative immortality	5	1	3	
Activating invasion and metastasis		2	3	
Evading growth suppressors	3	3	2	
Tumor promoting inflammation	1	1	1	
Avoiding immune destruction			1	

### Function-based stratification of glioblastoma cells

Neoplastic clusters have been defined according to the individual profiles of signaling circuit activities observed for each cell. The advantage of this way of cell stratification is that the functional profiles of each group are well defined. Current glioblastoma classification stratifies tumors into three subtypes, classical, proneural, and mesenchymal, from less to more aggressive, based on the signature of 50 genes (58). The SubtypeME tool from the GlioVis data portal (56) was used to assign subtype to each individual cell using this signature. Interestingly, when cells of the three neoplastic clusters are typed, the distribution of markers is very coherent with their functional activity profiles. Thus, neoplastic cluster 2 is mainly composed by cells belonging to the classical subtype (see Table 2), in coincidence with its functional profile being less aggressive. On the other hand, neoplastic cluster 1 has an important component of proneural cells, as well as a smaller proportion of mesenchymal cells, which is coherent with its more aggressive functionality triggered by its signaling activity, which includes modifications in circuits related to cell metabolism, genomic instability and metastasis. Moreover, the functional profile of neoplastic cluster 3 seems to be even more aggressive than that of neoplastic cluster 1. This group of glioblastoma cells not only has more circuits related to the same hallmarks as neoplastic cluster 1 but also has circuits that trigger functionalities for ‘Avoiding immune destruction’ (Table 2 and Figure 1). It is interesting to note that, although the conventional stratification in classical, mesenchymal and proneural classes is illustrative of the behavior of the cells, it does not completely fit with the stratification based on whole cell functional profiles.

**Table 2.** Distribution of the different glioblastoma subtypes across the three neoplastic cell clusters

	Classical	Mesenchymal	Proneural	Total
Neoplastic cell cluster 1	92	44	135	271
Neoplastic cell cluster 2	107	3	15	125
Neoplastic cell cluster 3	540	141	14	697

### Effect of a drug at single-cell level

Mechanistic models can be used to simulate the effect of an intervention over the system studied (25,35). Specifically, single-cell transcriptomic data offer, for the first time, the possibility of modeling the effects of a targeted drug at the level of individual cells.

The current indication for the treatment of glioblastoma patients is temozolomide, which induces DNA damage, that can be combined with other drugs such as bevacizumab to overcome resistances (38). Moreover, bevacizumab, which is indicated for several advanced cancer types, has recently been suggested for glioblastoma targeted treatment (60–62). Actually, the effect of bevacizumab, a humanized murine monoclonal antibody targeting the vascular endothelial growth factor ligand (VEGFA), can easily be simulated in the mechanistic model. *VEGFA* gene participates in six pathways (‘VEGF signaling pathway’, ‘Ras signaling pathway’, ‘Rap1 signaling pathway’, ‘HIF-1 signaling pathway’, ‘PI3K–Akt signaling pathway’ and ‘Focal adhesion pathway’) and is part of 81 circuits, 39 of them directly related to cancer hallmarks (18 to ‘Resisting cell death’, 9 to ‘Sustaining proliferative signaling’, 4 to ‘Genome instability and mutation’, 3 to ‘Evading growth suppressors’, 2 to ‘Enabling replicative immortality’, 2 to ‘Inducing angiogenesis’ and 1 to ‘Deregulation of cellular energetics’). As described in the ‘Materials and Methods’ section, the inhibition of VEGFA can be simulated by taking the gene expression profile of a single cell, creating a simulated profile by setting the inhibited gene to a low value and comparing two profiles (24,35).

**Figure 3.**
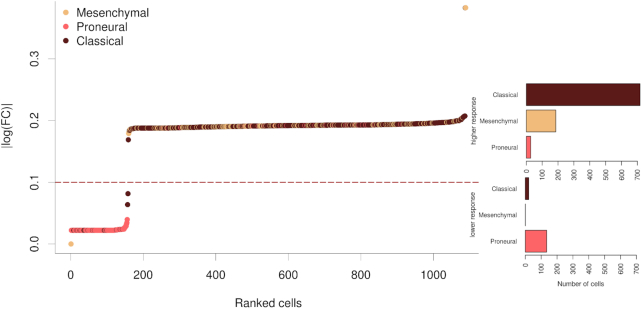
Impact of the inhibition of *VEGFA* by bevacizumab over the different neoplastic cells in terms of changes in the activities of signaling circuits in which this protein participates. The *Y*-axis depicts the magnitude of this change in the activities of signaling circuits. In the right part, two bar plots represent the proportion of the different cell types in the responder and non-responder groups.

Figure 3 shows the impact of the inhibition of VEGFA on the different cells in terms of changes in the activities of signaling circuits in which this protein participates. The *Y*-axis depicts the magnitude of this change in the activities of signaling circuits. There are clearly two different behaviors in the response: most of the cells present a drastic change in many signaling circuit activities (responders), while a smaller number of them present a much lower affectation on them (low responders). It is interesting to note that the distribution of cell types between both groups is also asymmetric: the responder group is mainly composed of cells that have been typed as classical or mesenchymal, while the non-responder group is predominantly composed of proneural cells.

**Figure 4.**
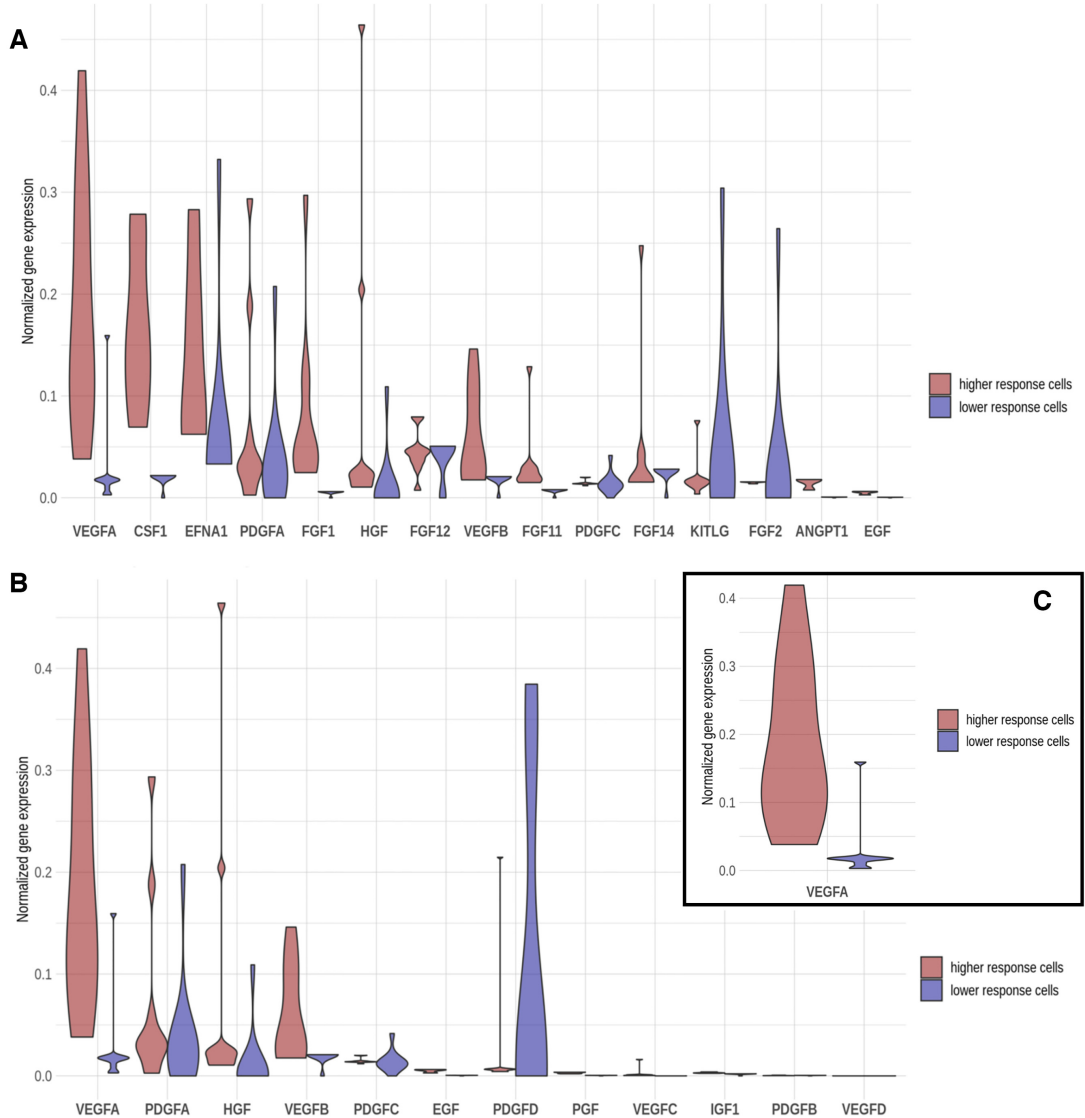
Distribution of the values of imputed and normalized gene expression values of the genes located within the effector node of the different signaling circuits affected by the bevacizumab inhibition. The distribution of observed expression levels in responder cells appears in red and in the low-responder cells in blue. (**A**) In the receptor node of the circuits within ‘Ras signaling pathway’, ‘Rap1 signaling pathway’ and ‘PI3K–Akt signaling pathway’, the VEGFA protein potentially shares the role of signal transducer with other 40 proteins (CSF1, EFNA1, PDGFA, FGF1, HGF, FGF12, VEGFB, FGF11, PDGFC, FGF14, KITLG, FGF2, ANGPT1, EGF, PDGFD, EFNA5, ANGPT2, PGF, VEGFC, FGF18, EFNA3, FGF5, EFNA4, IGF1, EFNA2, FGF9, FGF13, FGF17, PDGFB, NGF, ANGPT4, FGF7, FGF22, FGF16, FGF23, FGF19, FGF20, FGF8 and VEGFD). (**B**) In the receptor node of the circuits within ‘Focal adhesion pathway’, the VEGFA potentially shares the signal transduction role with other 12 proteins (PDGFA, HGF, VEGFB, PDGFC, EGF, PDGFD, PGF, VEGFC, IGF1, PDGFB and VEGFD). (**C**) In the six signaling circuits belonging to the ‘HIF signaling pathway’ and ‘VEGF signaling pathway,’ the protein VEGFA is the only signal transducer in the node.

A close look at the consequences of the inhibition of VEGFA in different cells provides an interesting explanation for the observed differences. VEGFA is upstream in the chain of signal transduction in several circuits of different pathways. In the circuits within ‘Ras signaling pathway’, ‘Rap1 signaling pathway’ and ‘PI3K–Akt signaling pathway’, the VEGFA protein potentially shares the role of signal transducer with other 40 proteins. Figure 4A clearly depicts how the balance between the expression level of VEGFA in the responsive cells and KITLG and FGF2 proteins, which can take a similar signaling role, changes. Supplementary Figure S2 shows the impact of the simulation of VEGFA inhibition in the ‘PI3K–Akt signaling pathway’, where the differences in the impact of this inhibition, measured as the log fold change in signaling activity, are remarkable between responder and low-responder cells. The inhibition of VEGFA in the responsive cells will radically inhibit the signal. However, the low-responder cells have already *VEGFA* at low expression levels and the signal is transmitted by KITLG and FGF2 instead, which ultimately compromises the success of the drug. A similar scenario occurs with the ‘Focal adhesion pathway’, in which VEGFA shares the signal transduction role with other 12 proteins. In this case, the low-responder cells are characterized by a low level of *VEGFA* compensated with a high level of *PDGFD*, which makes these signaling circuits in the low-responder cells virtually insensitive to the inhibition of VEGFA (see Figure 4B). Only in the case of six signaling circuits belonging to the ‘HIF signaling pathway’ and ‘VEGF signaling pathway’, the protein VEGFA is the only signal transducer in the node. In this case, low-responder cells have this circuit constitutively down and, consequently, are not affected by the inhibition (Figure 4C). Actually, except for *FGF2*, the genes potentially responsible for this switch presented a significant differential expression when responders were compared to low-responders [applying the limma (52) test, the FDR-adjusted *P*-values for *VEGFA*, *PGDFD*, *KITLG* and *FGF2* were, respectively, 6.4 × 10^−4^, 9.2 × 10^−2^, 1.39 × 10^−2^ and 5.59 × 10^−1^, using the gene expression values prior to the imputation). Supplementary Figure S3 represents the expression values of the same genes as in Figure 4 but with no imputation, showing no significant differences.

The same modeling strategy used with bevacizumab can be applied to simulate the effect of other drugs. Recently, a drug repurposing *in silico* experiment that combines human genomic data with mouse phenotypes has suggested the possible utility of a number of drugs with different indications (see Table 3) for potential glioblastoma treatment (63). The intensity and the degree of heterogeneity in the response are very variable across the eight drugs tested here. At the scale we tested the drugs, there are no correlations either between the number of genes targeted by the drug and the intensity of the effect or between the number of circuits potentially affected and the intensity of the effect. For example, pentoxifylline targets four proteins (PDE4B, ADORA1, PDE4A and ADORA2A) that participate in a total of 111 circuits and the fold change caused in the circuit activity after simulating its effect is comparatively low (log fold change <0.05 for all the cell types; see Supplementary Figure S4), while the simulation of the effect of fenofibrate, which targets only one protein PPARA that participates in only 32 circuits, renders a comparatively high effect (log fold change >2 for all the cell types; see Supplementary Figure S4). It is interesting to note that, depending on the case, the different drug effects simulated can affect a larger or a smaller number of cells with distinct intensity in their impacts on the activity of the signaling circuits affected, but always, no matter which drug is simulated, there are some cells that manage to escape from the inhibitory effect of the drug.

**Table 3.** The eight drugs whose effect on the neoplastic population cell has been simulated

Drug name	Gene name	Action	Circuits	Invasion and metastasis	Immune destruction	Cellular energetics	Replicative immortality	Evading growth suppressors	Genome instability and mutation	Inducing angiogenesis	Resisting cell death	Sustaining proliferative signaling	Tumor promoting inflammation
**Bevacizumab**	*VEGFA*	Inhibitor	81			1	2	3	4	2	18	9	
**Imiquimod**	*TLR7*	Agonist	11		1					1	1		4
**Sulfasalazine**	*PTGS2*	Inhibitor	5						1				
**Sulfasalazine**	*PTGS1*	Inhibitor	2						1				
**Sulfasalazine**	*PPARG*	Agonist	21			2				1			
**Pentoxifylline**	*PDE4B*	Inhibitor	1										
**Pentoxifylline**	*ADORA1*	Antagonist	59						7		6	4	
**Pentoxifylline**	*PDE4A*	Inhibitor	1										
**Pentoxifylline**	*ADORA2A*	Antagonist	50						6	1	3	5	
**Fenofibrate**	*PPARA*	Agonist	32			1			1	1			
**Doxepin**	*HRH1*	Antagonist	4										
**Doxepin**	*HRH2*	Antagonist	0										
**Quetiapine**	*HTR2A*	Antagonist	13										
**Quetiapine**	*DRD2*	Antagonist	52						5		3	4	
**Nilotinib**	*ABL1*	Inhibitor	14								2		

The hallmarks affected are displayed in the columns on the right-hand side of the table.

## DISCUSSION

The goal of most scRNA-seq publications revolves around the characterization of cell populations, which can be accurately achieved using only a subset of the total number of genes (those displaying the highest variability across cells). However, the use of mechanistic models to estimate global signaling circuit activity profiles for individual cells requires reasonably accurate measures of the expression levels of all the genes involved in the signaling circuits. Dropout events, quite common in scRNA-seq experiments (13), are particularly problematic given that taking by mistake a missing value by a real zero value can cause erroneous determinations of the inferred activity of the circuits. Thus, we explored the performance of three different imputation methods in producing cell-specific profiles of signaling circuit activity whose clustering resulted in a grouping similar to that observed and validated in the original glioblastoma study. Here, two machine learning-based methods, DrImpute and MAGIC, produced a clustering compatible with the original validated clustering, and specifically, DrImpute, the method of choice, rendered clusters with a similar shape as well (see Figure 1).

Focusing on neoplastic cells, the existence of three different clusters is also apparent at the level of functional profiles, which suggests the existence of different functional behaviors. Several attempts to stratify glioblastoma patients have been proposed by discriminating different subtypes according to different properties, such as patient survival (64), mutational status of some genes (65) or the tumor microenvironment (66). In the most used classification, glioblastoma tumors were divided into three subtypes (from less to more aggressive: classical, proneural and mesenchymal) based on the signature of 50 genes (57). Although this conventional subtyping provides an approximate descriptor of tumor aggressiveness, subtyping based on functional profiles related to cancer hallmarks provides an interesting alternative for the stratification of glioblastoma that offers, in addition, a mechanistic description on the functional activity of the tumor. Actually, it has been reported in neuroblastoma that signaling pathway models used as biomarkers outperform traditional biomarkers as predictors of patient survival (26). Supplementary Figure S5 provides an interactive view of the circuits activated and deactivated within the different pathways.

Among pathways that are commonly altered in all three clusters, we find well-known factors contributing to carcinogenesis, such as those related to hypoxia (HIF-1, SOD2), cancer stem cells (CSCs), cell cycle proteins, like CDK family, signal transduction pathways and hormone signaling (67–69). Moreover, these are mainly related to ‘Sustaining proliferative signaling’, ‘Enabling replicative immortality’ and ‘Resisting cell death’ hallmarks that can be defined as the core cell functions involved in glioblastoma initiation and proliferation.

Each cluster exhibits a characteristic deregulation of pathways; however, cluster 2 barely has four unique sub-pathways, all related to common hallmarks ‘Resisting cell death’ and ‘Sustaining proliferative signaling’. Neoplastic clusters 1 and 3, but not cluster 2, exhibit ‘Genome instability’, a hallmark observed in almost all sporadic human cancers, including glioblastoma (70,71). Besides, clusters 1 and 3 show deregulated ‘Cellular energetics’, a process that has been suggested as a suitable target for tumor cell elimination (72). Furthermore, both clusters show pathways associated with matrix metalloproteins and Snail family that have been linked to cancer invasion and metastasis also in glioblastoma (73–75). Interestingly, only cluster 3 may be avoiding immune destruction due to the deregulation of ‘Toll-like receptor signaling pathway’. Glioblastoma is known to have a strongly immunosuppressive microenvironment; thus, blocking these cells by activating downstream TLR signaling pathways can reduce tumor growth and disrupt CSC self-renewal (76,77).

We have demonstrated that not all the cells in a tumor are driven by the same cancer processes, and that those alterations can define subpopulations that may confer tumors different aggressiveness and invasion abilities, highlighting the relevance of heterogeneity, beyond the widely accepted stratification of glioblastoma in three/four subtypes (78,79).

The emergence of mechanisms of resistance in targeted therapies has been attributed to either the selection of rare pre-existing genetic alterations upon drug treatment (80) or the transient acquisition of a drug-refractory phenotype by a small proportion of cancer cells through epigenetic modifications (81). In both cases, these alterations would be detectable in the expression of the corresponding genes. An interesting property of mechanistic models is that they can be used to model the effect of an intervention over the system studied (25,35). Thus, the use of mechanistic models on single-cell transcriptomic data offers for the first time the possibility of modeling the effects of a targeted drug in individual cells. From Figure 4 it becomes apparent that low-responder cells have a constitutive level of *VEGFA* lower than high-responder cells. However, these cells maintain active the same *VEGFA*-activated pathways by the upregulation of others alternative TRK receptor ligands (i.e. FGF2, PDGFD and KITLG) that have been implicated in the development and drug resistance in other cancer types (82–84). Interestingly, the switch in the expression levels of *FGF1* and *FGF2* (downregulated and upregulated in low-responder cells, respectively) as well as the upregulation of one member of the PDGF family (i.e. *PDGFD*) might be potentially driving the tumor progression in these GBM low-responder cells. Specifically, FGF2 is the main member of the FGF family implicated in cancer development and drug resistance (85), and PDGFD and its receptor (PDGFRB) have been recently defined as key drivers of tumor progression since a *PDGFRB* downregulation impairs immediately GBM progression (A.C.V.-B. Fuentes-Fayos et al., submitted for publication). Moreover, although the relevance of *KITLG* has not been defined in GBM, the upregulation found in GBM low-responder cells might be linked to the drug resistance of these cells as has been reported in other tumor pathologies (84). In fact, this particular expression phenotype found in low-responder cells could be similar to that previously described in neural stem cell progenitors that are directly associated with the development and drug resistance in GBMs (86,87). Thus, the mechanistic model provides a simple potential interpretation of the molecular mechanisms behind the differential effect of drugs over cells with different signaling profiles that ultimately cause different functional strategies. Obviously, in order to gain insights into the true mechanisms driving cell resistance, further studies are needed in line with these findings. Nevertheless, we have proven that functional single-cell analyses, and the methodology here presented, are a helpful tool for discovering tumor heterogeneity, and the results can be applied by clinical community to forward tailored treatment, therefore improving patient’s prognosis.

Supplementary Figure S4 depicts the simulated response of individual cells to the treatment with different targeted drugs. Despite the variety of effects in the cells, it is worth noting that there is always a group of cells that manages to escape from the inhibition of the drug. The heterogeneity observed in the cell population in terms of use of different strategies to activate the essential cancer hallmark through different signaling circuits produces a consequent diversity in the response to drugs. Although the number of drugs simulated is relatively low, given that drug repurposing was beyond the scope of this paper, the results obtained here suggest that the escape of a relatively small number of cells from the effect of the drug could be a relatively frequent event that occurs as a natural consequence of the heterogeneity in the signaling strategies followed by the cell population. When this subpopulation becomes dominant some time later, it becomes resistant to the drug.

## CONCLUSIONS

The use of mechanistic models provides a detailed insight into the functional strategies used by tumors to proliferate and open new avenues for the design of interventions *à la carte*. The extension of this analytical approach to single-cell transcriptomic data allows an unprecedented detail on how cancer cells display different functional strategies to proliferate that have consequences in their respective vulnerabilities to targeted therapies.

Although the existence of resistant clones in a tumoral cell population is well known, the specific mechanisms used by resistant cells to escape from the inhibitory effects of targeted therapies remain unknown yet. Mechanistic models offer for the first time a plausible and contrastable hypothesis on how and why some cells are insensitive to treatments, illustrated here with bevacizumab in the case of glioblastoma. Mechanistic modeling of effects of bevacizumab inhibitions at single-cell level revealed, how in some cells, with low *VEGFA* expression, the VEGF pathway remains active because the initial signaling was assumed by other proteins like PDGFD, KITLG and FGF2, thus making the signaling circuit insensitive to the VEGFA inhibition by the drug.

The use of this modeling strategy offers a systematic way for detecting tumoral cells that may be resistant to specific targeted treatments. Conversely, the same models could be used to find an alternative treatment for resistant drugs. In fact, our results suggest that the search for new, more efficient therapeutic targets would be benefited by the use of mechanistic models that guide to the intervention points with more likelihood of success in inhibiting the proliferation of the largest possible part of the spectrum of functional strategies in the tumor cell ecosystem.

## Supplementary Material

zcaa011_Supplemental_Files
