# Supplementary material for: Mechanistic models of signaling pathways deconvolute the glioblastoma single-cell functional landscape
Source: NAR Cancer. 2020 Jun 25;2(2):zcaa011. doi: 10.1093/narcan/zcaa011 (PMC8210212; doi:10.1093/narcan/zcaa011)

Supplementary Figures

Mechanistic models of signaling pathways deconvolute the functional landscape of glioblastoma at single cell resolution

Matías M. Falco[1,2], María Peña-Chilet[1,2], Carlos Loucera[1], Marta R. Hidalgo[3], Joaquín Dopazo[1,2,4,5]

1. Clinical Bioinformatics Area, Fundación Progreso y Salud (FPS), Hospital Virgen del Rocío, Sevilla, 41013, Spain

2. Bioinformatics in Rare Diseases (BiER), Centro de Investigaciones Biomédicas en Red en Enfermedades Raras (CIBERER).

3. Unidad de Bioinformática y Bioestadística, Centro de Investigación Príncipe Felipe (CIPF).

4. Functional Genomics Node, FPS/ELIXIR-ES, Hospital Virgen del Rocío, Sevilla, Spain

5. Computational Systems Medicine group, Institute of Biomedicine of Seville (IBIS) Hospital Virgen del Rocío, Sevilla, 41013, Spain

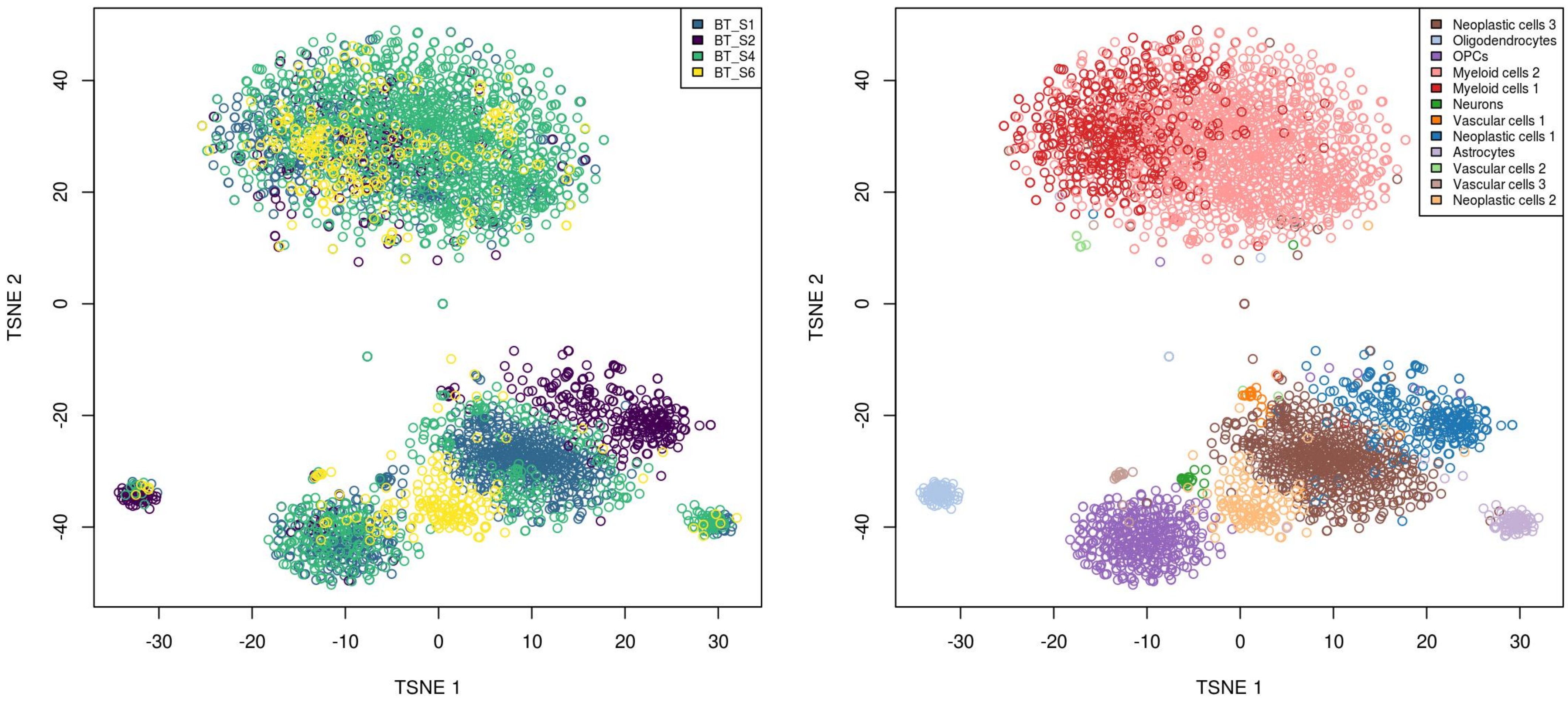

Supplementary Figure 1. **Clustering of the samples based on gene expression and on signaling circuit activities obtained with the DRImpute imputation method**. Data were subjected to tSNE dimensionality reduction and the k-means clustering of the two main components is represented. Left panel shows the cells labelled by individual and right panel shows the same cells labeled by cell type.

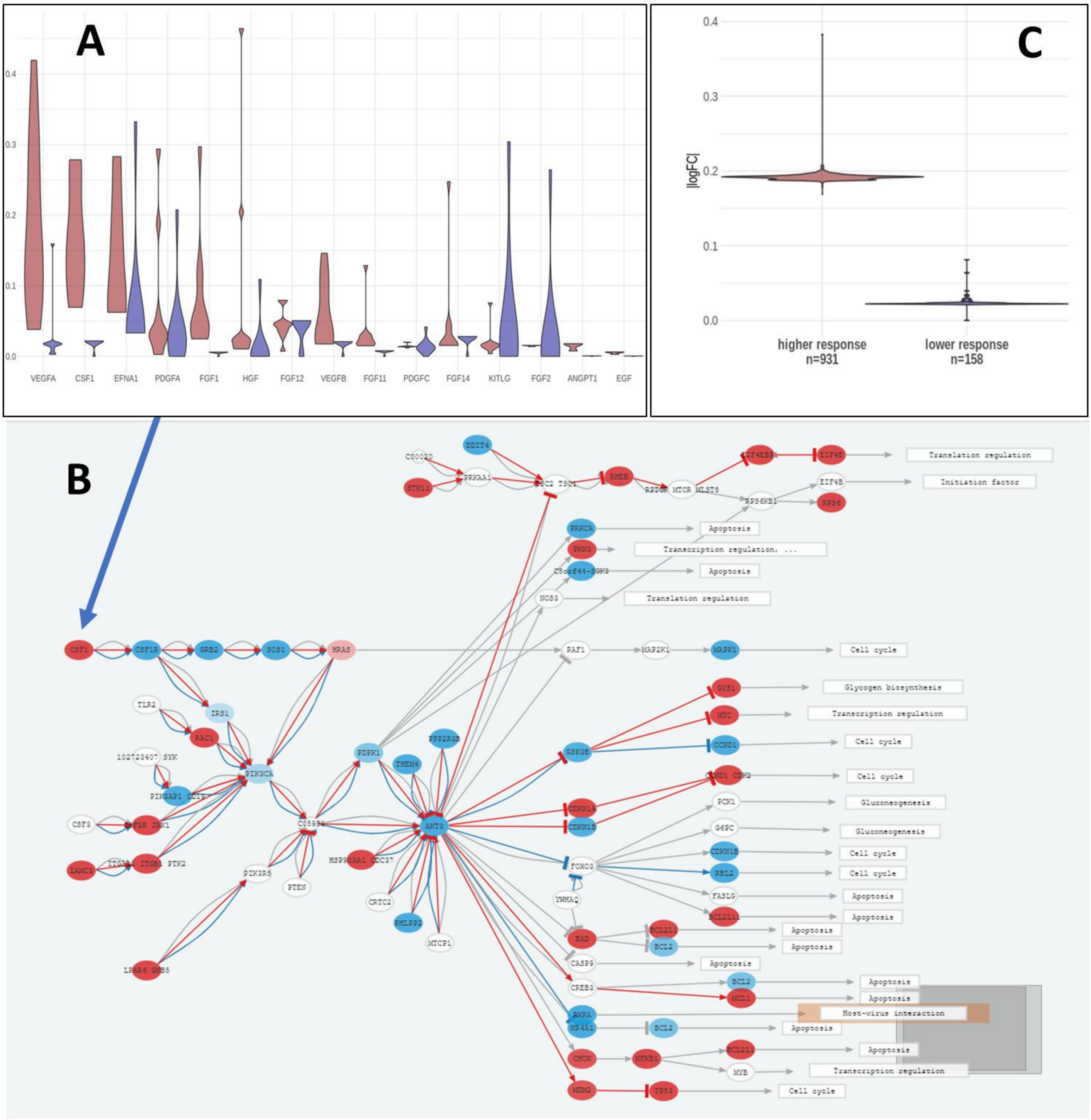

Supplementary Figure 2. The PI3K-Akt signaling pathway in a comparison between the three neoplastic clusters and the normal cells carried out with the Hipathia algorithm. Colors in the nodes refer to over-expression of the gene(s) of the node in the Neoplastic cells with respect to the normal cells, and red color vice-versa. Pale gray nodes do not change across the compared conditions. Similarly, red edges mean over-activated circuits in neoplastic cells with respect to normal cells, while blue are under-activated, and grey do not change the signaling status. Interestingly, the *VEGFA* protein potentially share the role of primary signal transducer with other 40 proteins (*CSF1, EFNA1, PDGFA, FGF1, HGF, FGF12, VEGFB, FGF11, PDGFC, FGF14, KITLG, FGF2, ANGPT1, EGF, PDGFD, EFNA5, ANGPT2, PGF, VEGFC, FGF18, EFNA3, FGF5, EFNA4, IGF1, EFNA2, FGF9, FGF13, FGF17, PDGFB, NGF, ANGPT4, FGF7, FGF22, FGF16, FGF23, FGF19, FGF20, FGF8* and *VEGFD*). A) shows the distribution of gene expression values for the 12 most expressed genes in the node in responder cells (red) and low-responder cells. B) the first node of the PI3K-Akt signaling pathway is the primary signal transducer. C) If the effect of Bevacizumab is simulated as an inhibition of *VEGFA*, the panel shows the log fold change of signaling activities caused by the inhibition in responder cells (red) and low-responder cells (blue).

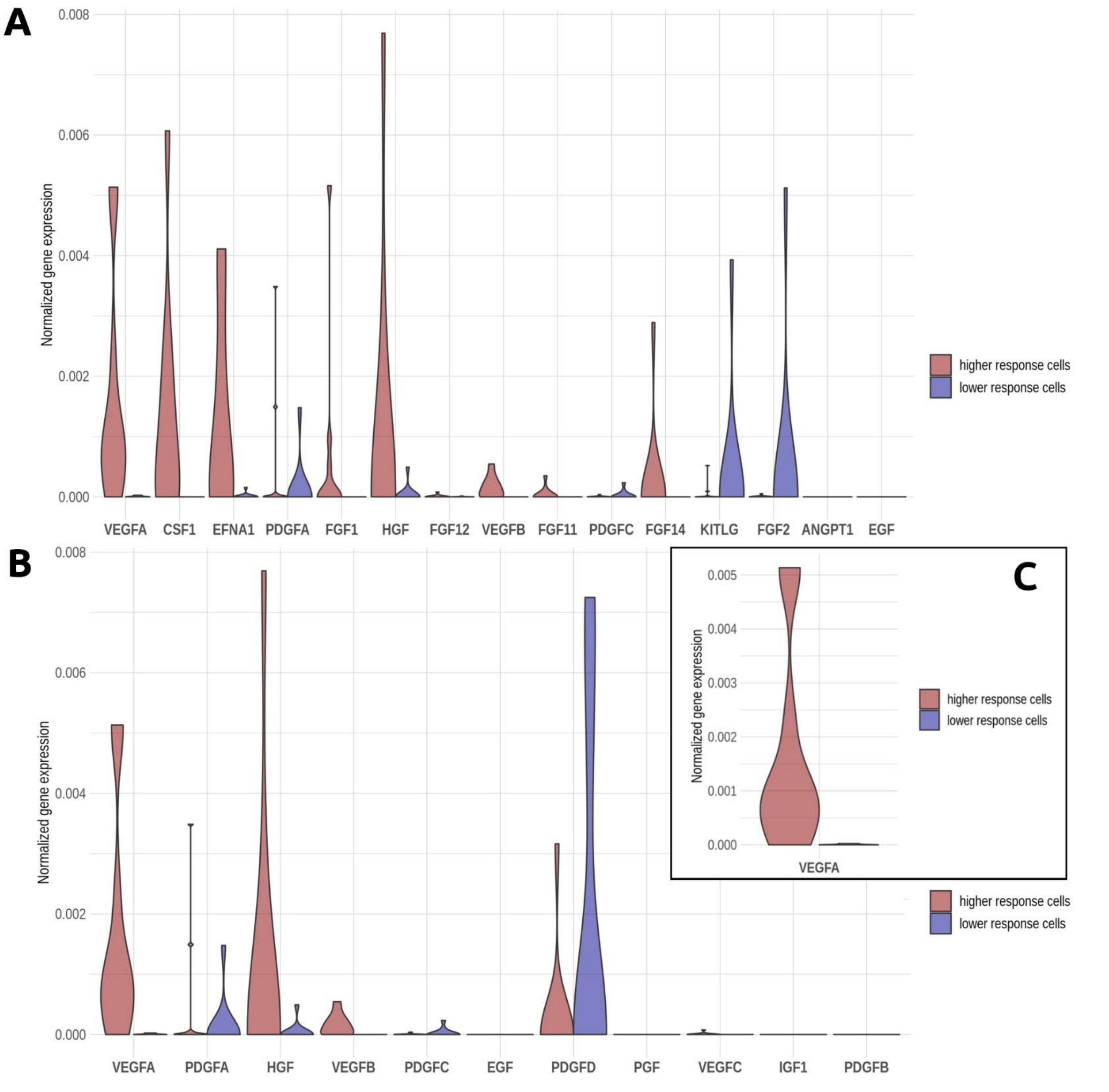

Supplementary Figure 3. Distribution of the values of normalized gene expression values of the genes located within the effector node of the different signaling circuits affected by the bevacizumab inhibition. The distribution of observed expression levels in responder cells appears in red and in the low-responders in blue. A) in the receptor node of the circuits within *Ras signaling pathway, Rap1 signaling pathway* and *PI3K-Akt signaling pathway,* the *VEGFA* protein potentially share the role of signal transducer with other 40 proteins (*CSF1, EFNA1, PDGFA, FGF1, HGF, FGF12, VEGFB, FGF11, PDGFC, FGF14, KITLG, FGF2, ANGPT1, EGF, PDGFD, EFNA5, ANGPT2, PGF, VEGFC, FGF18, EFNA3, FGF5, EFNA4, IGF1, EFNA2, FGF9, FGF13, FGF17, PDGFB, NGF, ANGPT4, FGF7, FGF22, FGF16, FGF23, FGF19, FGF20, FGF8* and *VEGFD*). B) in the receptor node of the circuits within *Focal adhesion pathway* the *VEGFA* potentially shares the signal transduction role with other 12 proteins (*PDGFA, HGF, VEGFB, PDGFC, EGF, PDGFD, PGF, VEGFC, IGF1, PDGFB* and *VEGFD*). C) in the 6 signaling circuits belonging to the *HIF signaling pathway* and *VEGF signaling pathway* the protein *VEGFA* is the only signal transducer in the node.

Supplementary Figure 4. Impact of the inhibition different targeted treatments over the different neoplastic cells in terms of changes in the activities of signaling circuits in which this protein participates. The figure on the left shows the global differences observed in the three clusters and the figure on the right depicts the change individualized by cells (labeled as mesenchymal, proneural and classical). The Y axis depicts the magnitude of this change in the activities of signaling circuits. The targeted treatments are: A) Bevacizumab, B) Doxepin, C) Fenofibrate, D) Imiquimod, E) Nilotinib, F) Pentoxifylline, G) Quetiapine, H) Sulfasalacine.

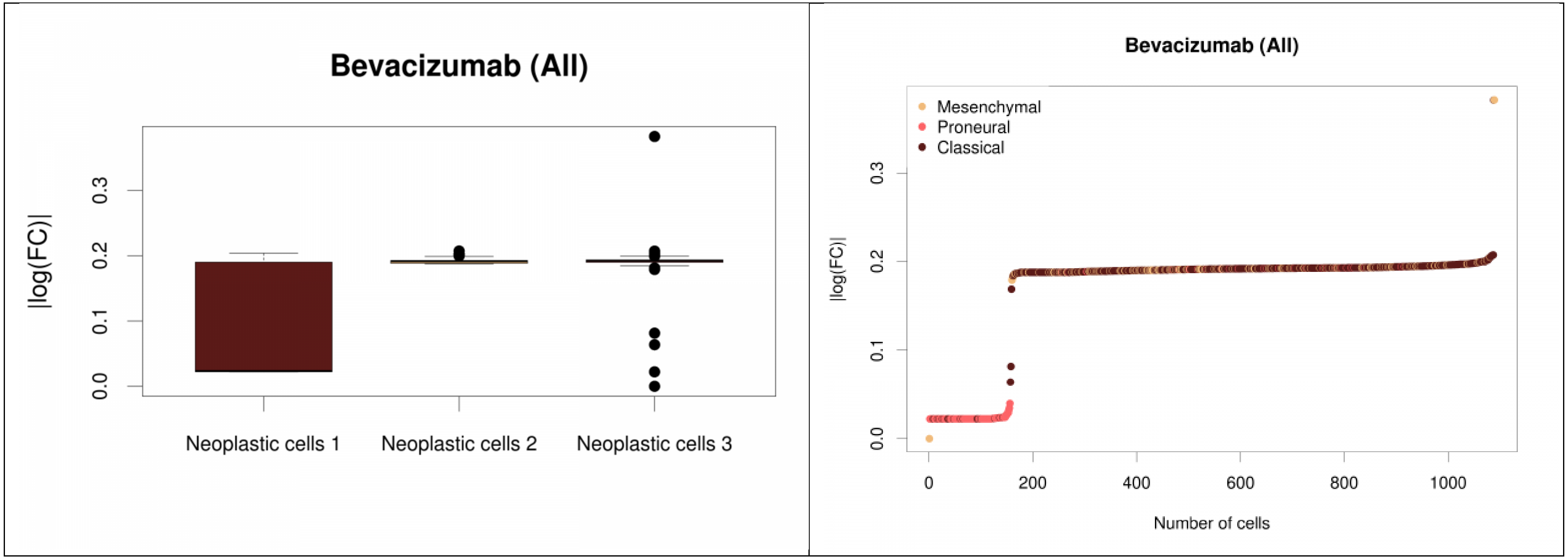
Bevacizumab (All)
|log(FC)|
0.0
0.1
0.2
0.3
Neoplastic cells 1
Neoplastic cells 2
Neoplastic cells 3
Bevacizumab (All)
Mesenchymal
Proneural
Classical
|log(FC)|
0.0
0.1
0.2
0.3
0
200
400
600
800
1000
Number of cells

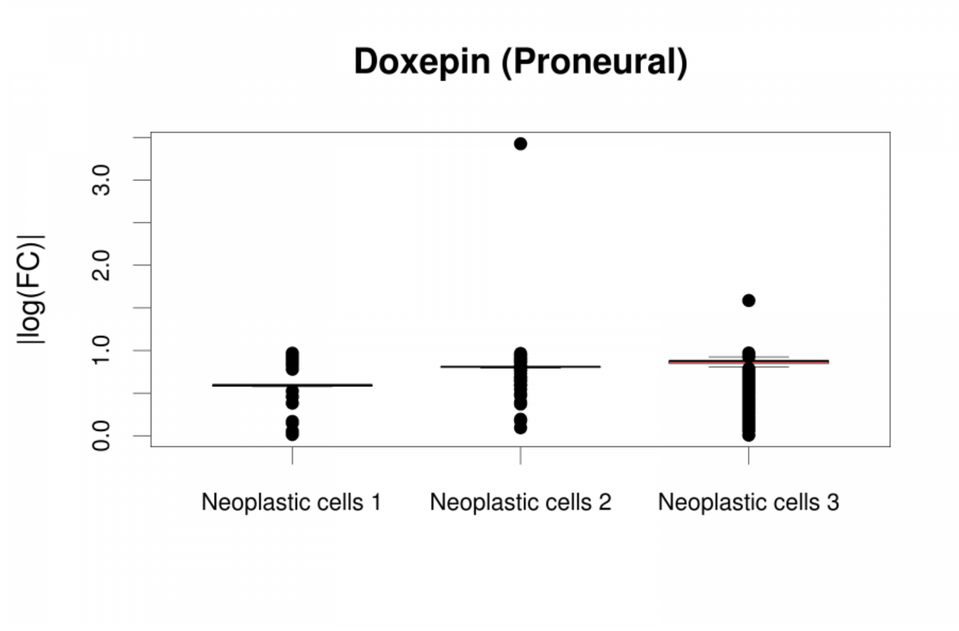

Doxepin (Proneural)
|log(FC)|
0.0
1.0
2.0
3.0
Neoplastic cells 1
Neoplastic cells 2
Neoplastic cells 3

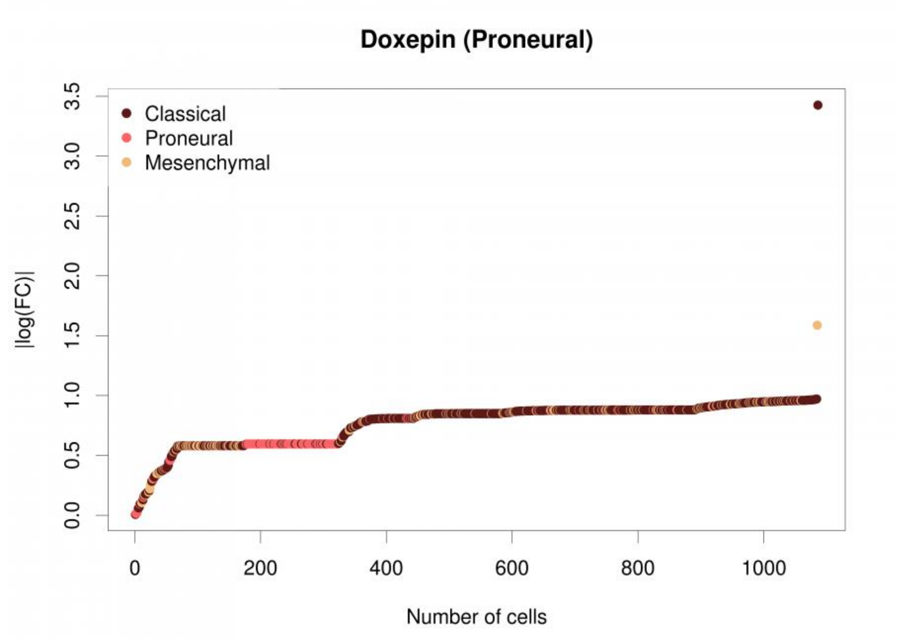

Doxepin (Proneural)
Classical
Proneural
Mesenchymal
|log(FC)|
0.0
0.5
1.0
1.5
2.0
2.5
3.0
3.5
0
200
400
600
800
1000
Number of cells

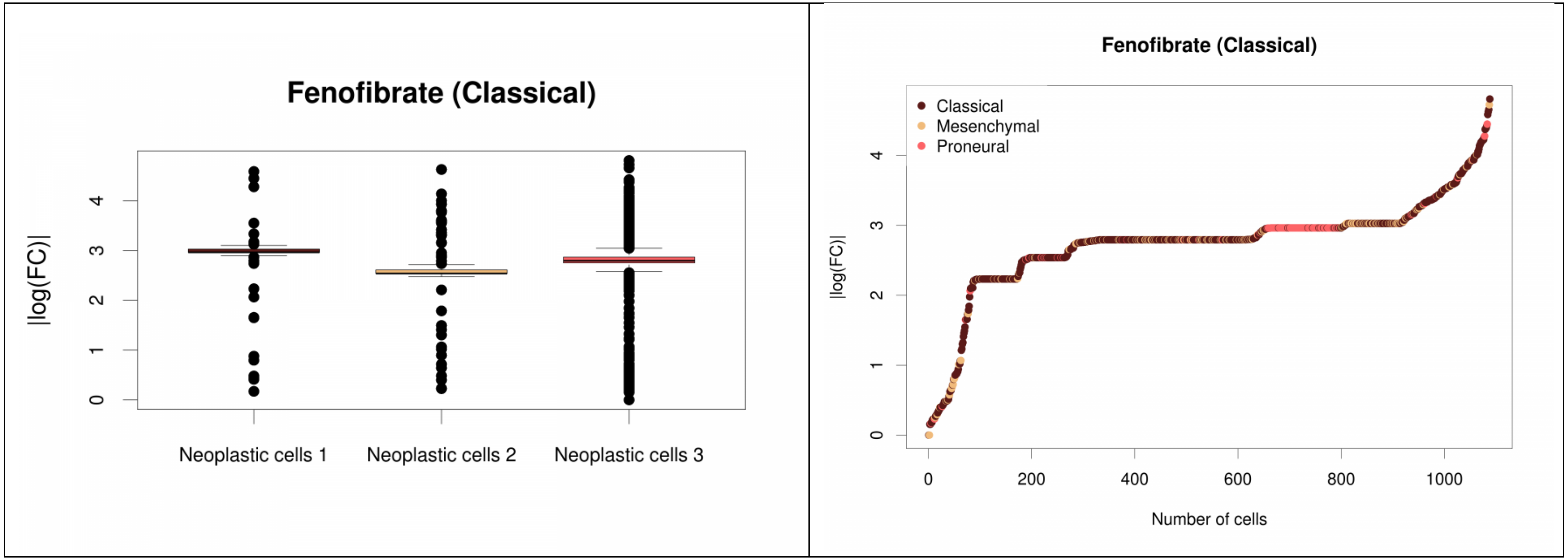
Fenofibrate (Classical)
|log(FC)|
0
1
2
3
4
Neoplastic cells 1
Neoplastic cells 2
Neoplastic cells 3
Fenofibrate (Classical)
Classical
Mesenchymal
Proneural
|log(FC)|
0
1
2
3
4
0
200
400
600
800
1000
Number of cells

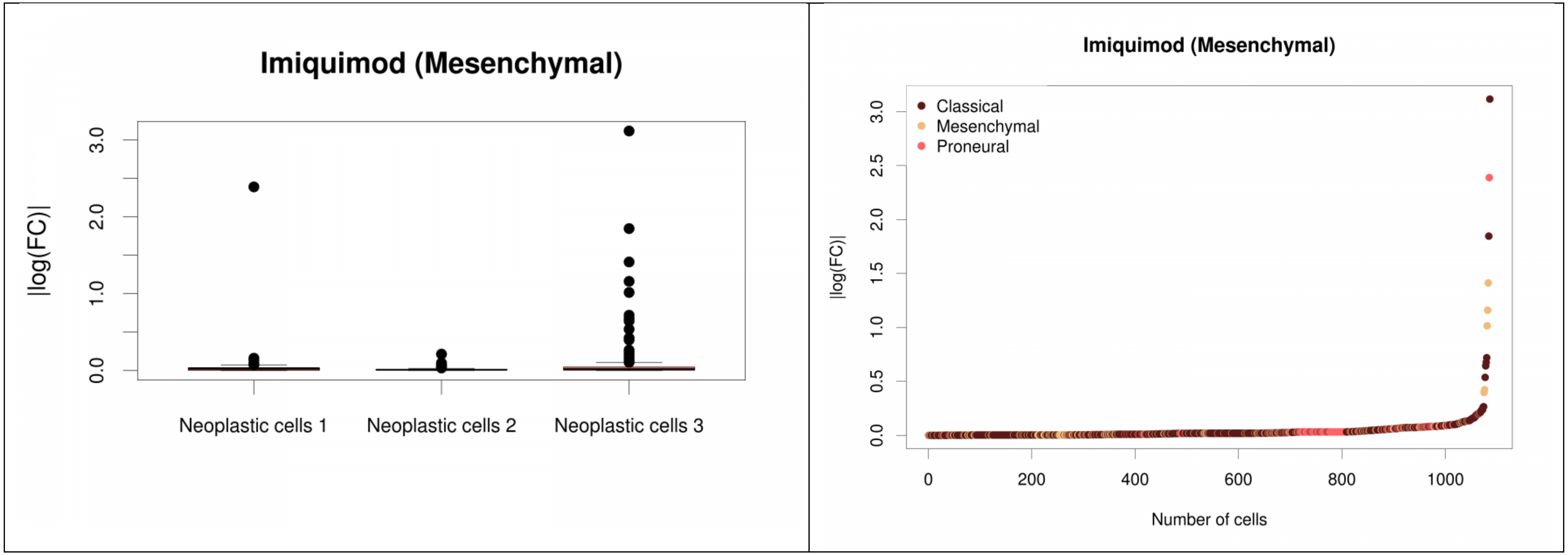
Imiquimod (Mesenchymal)
|log(FC)|
0.0
1.0
2.0
3.0
Neoplastic cells 1
Neoplastic cells 2
Neoplastic cells 3
Imiquimod (Mesenchymal)
Classical
Mesenchymal
Proneural
|log(FC)|
0.0
0.5
1.0
1.5
2.0
2.5
3.0
0
200
400
600
800
1000
Number of cells

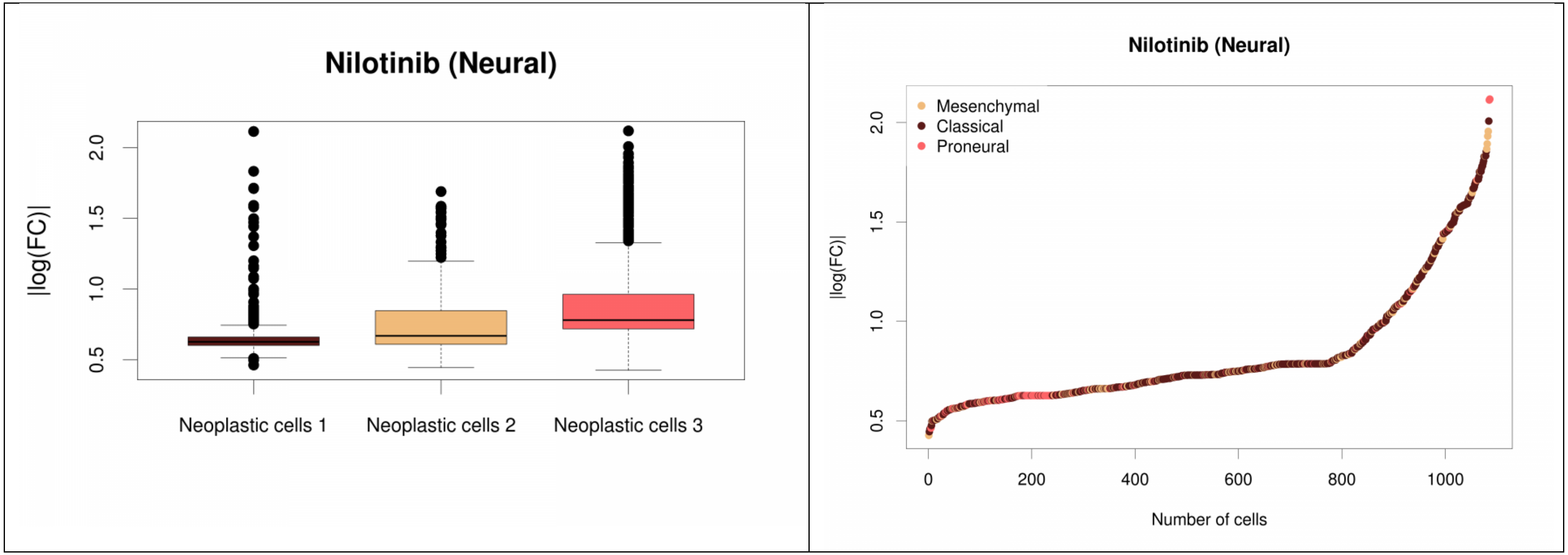
Nilotinib (Neural)
|log(FC)|
0.5
1.0
1.5
2.0
Neoplastic cells 1
Neoplastic cells 2
Neoplastic cells 3
Nilotinib (Neural)
Mesenchymal
Classical
Proneural
|log(FC)|
0.5
1.0
1.5
2.0
0
200
400
600
800
1000
Number of cells

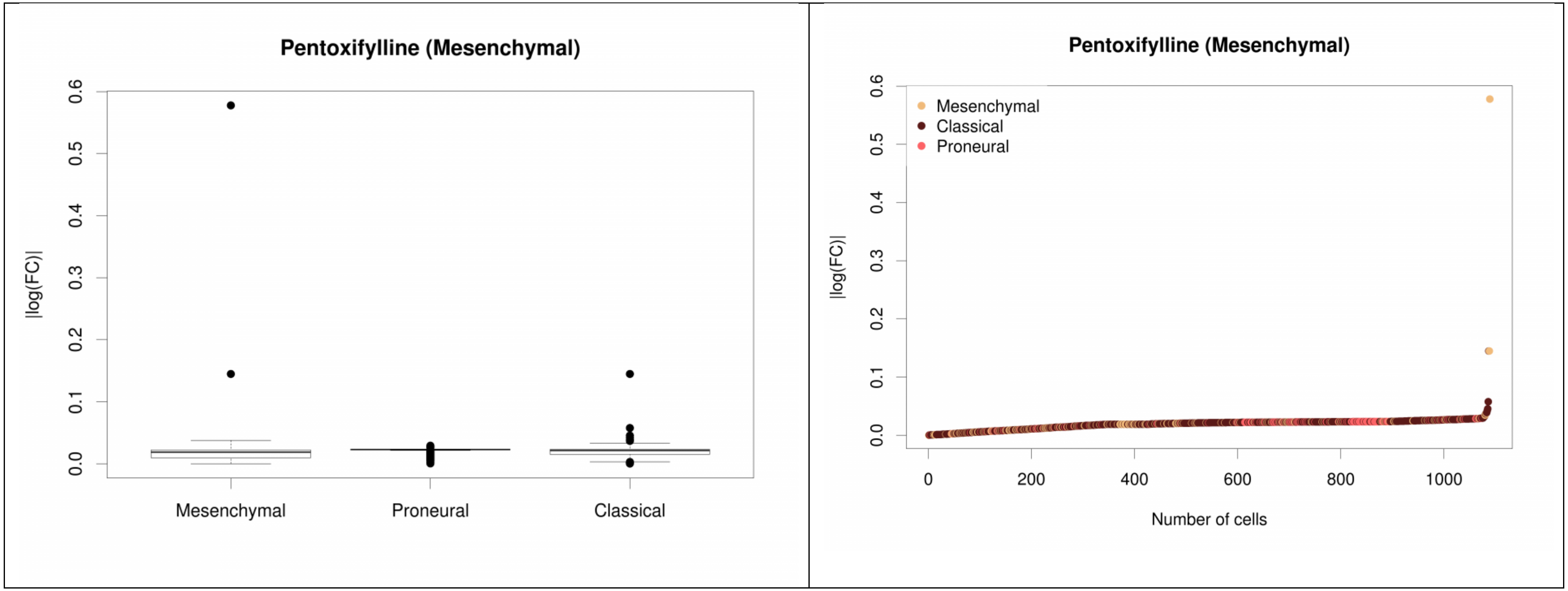

Pentoxifylline (Mesenchymal)
|log(FC)|
0.0
0.1
0.2
0.3
0.4
0.5
0.6
Mesenchymal
Proneural
Classical
Pentoxifylline (Mesenchymal)
Mesenchymal
Classical
Proneural
|log(FC)|
0.0
0.1
0.2
0.3
0.4
0.5
0.6
0
200
400
600
800
1000
Number of cells

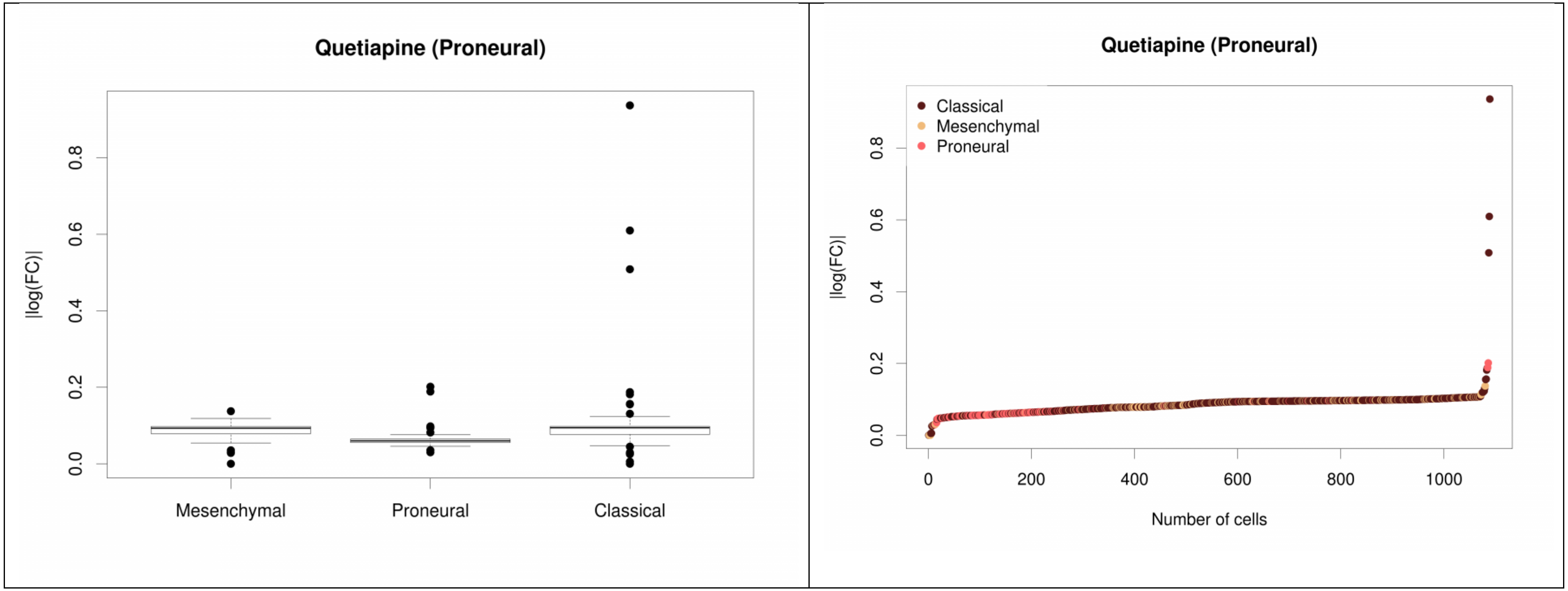
Quetiapine (Proneural)
|log(FC)|
0.0
0.2
0.4
0.6
0.8
Mesenchymal
Proneural
Classical
Quetiapine (Proneural)
Classical
Mesenchymal
Proneural
|log(FC)|
0.0
0.2
0.4
0.6
0.8
0
200
400
600
800
1000
Number of cells

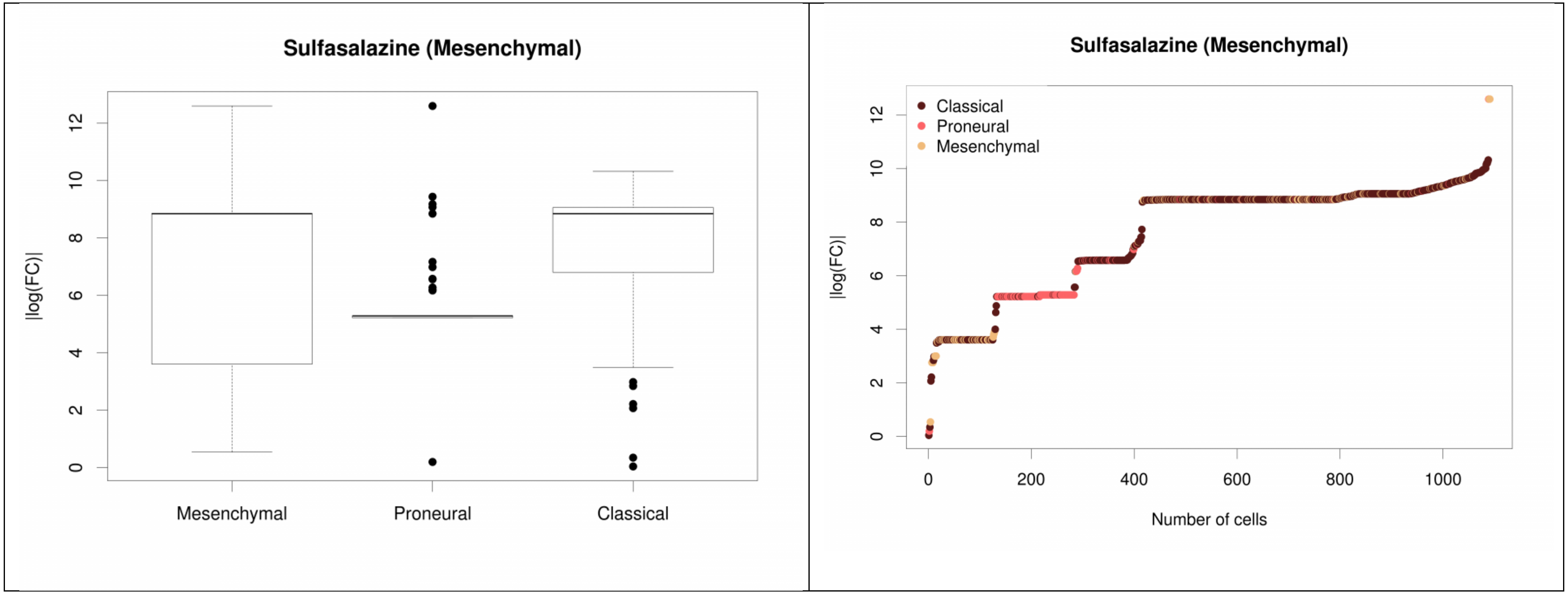
Sulfasalazine (Mesenchymal)
|log(FC)|
0
2
4
6
8
10
12
Mesenchymal
Proneural
Classical
Sulfasalazine (Mesenchymal)
Classical
Proneural
Mesenchymal
|log(FC)|
0
2
4
6
8
10
12
0
200
400
600
800
1000
Number of cells

Supplementary Figure 5. Comparison between the neoplastic cells and the neural cells in detail. The supplementary material contains a collection of pathways mentioned in the text: Focal adhesion, HIF-1, PI3K-Akt, Rap1, Ras and VEGF, along with details of the corresponding differentially activated circuits.

Additionally, an interactive visualization of this comparison is also possible. In order to interactively visualize the pathways and circuits you must:

i) download the comparison file in Zenodo (https://doi.org/10.5281/zenodo.3856679),

ii) download and install the the Hipathia Bioconductor package (https://www.bioconductor.org/packages/release/bioc/html/hipathia.html),

iii) use the function visualize.report(), specifying the path to supplementary data (the file "Supplementary Figure 5") in your computer. The function invokes a local web server that provides the functionality of the HiPathia web application. The user can navigate across the pathways and focus on specific circuits that are up- or down-activated.

Focal adhesion (hsa04510)

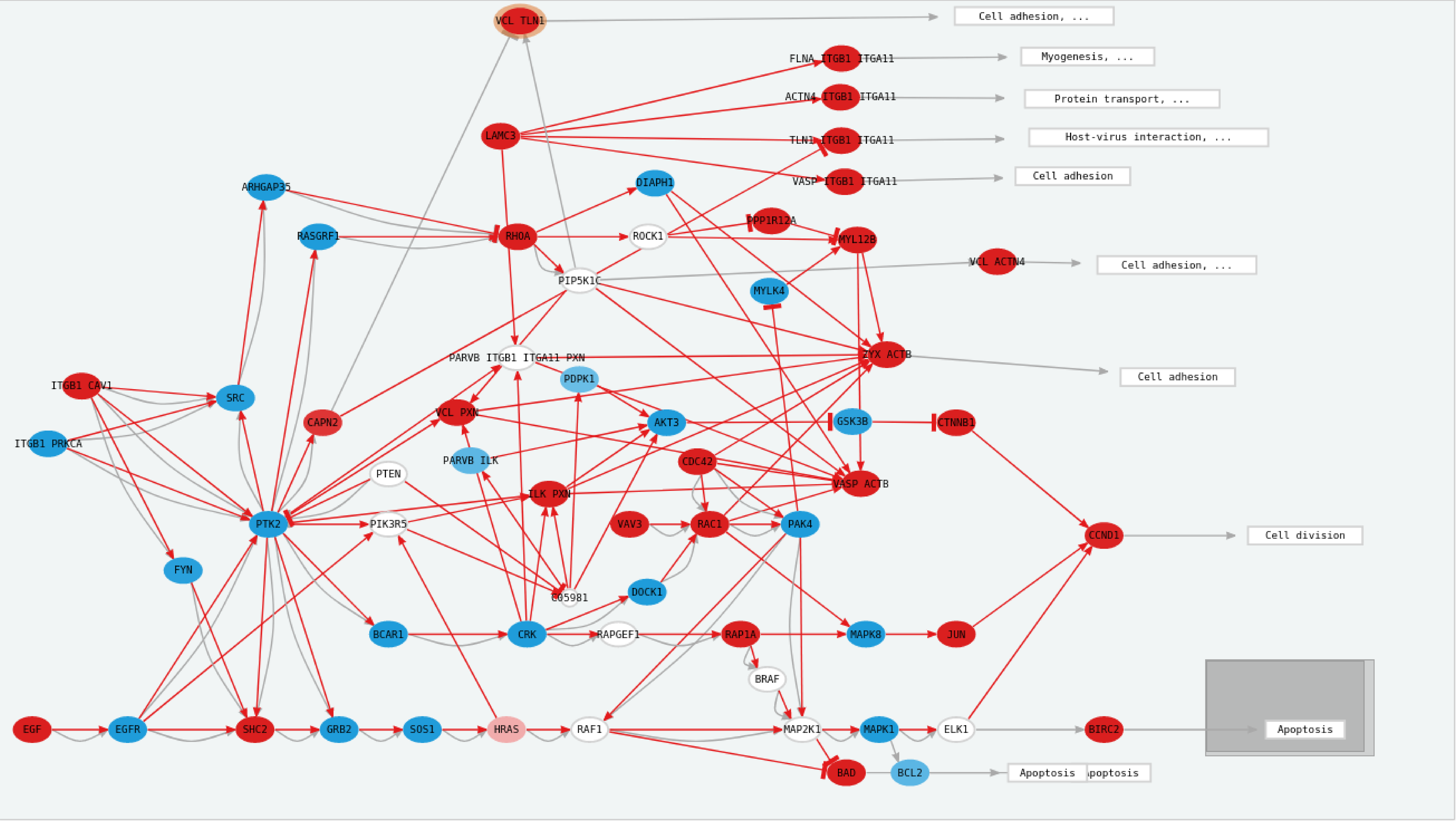

Focal adhesion (hsa04510)

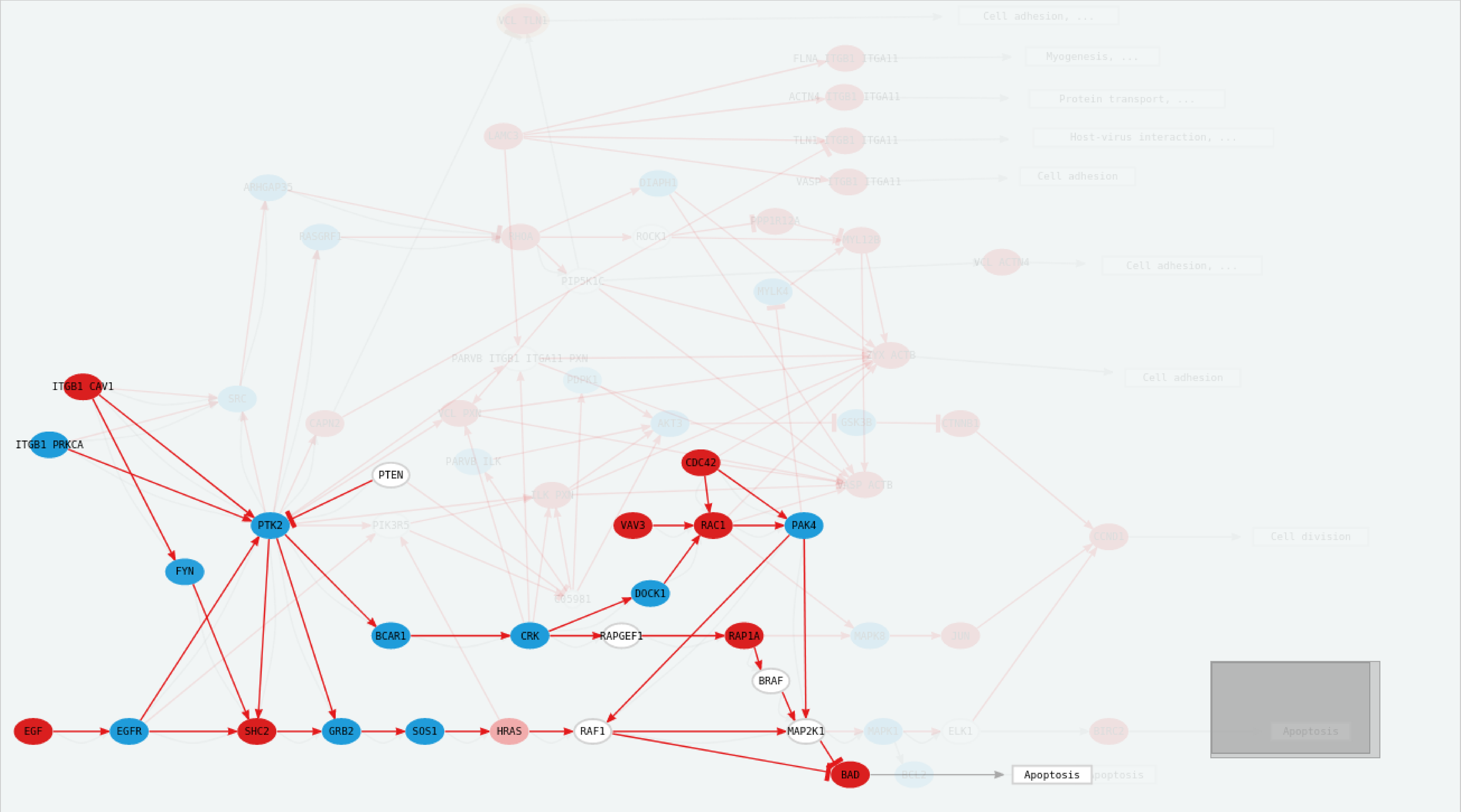

Focal adhesion (hsa04510)

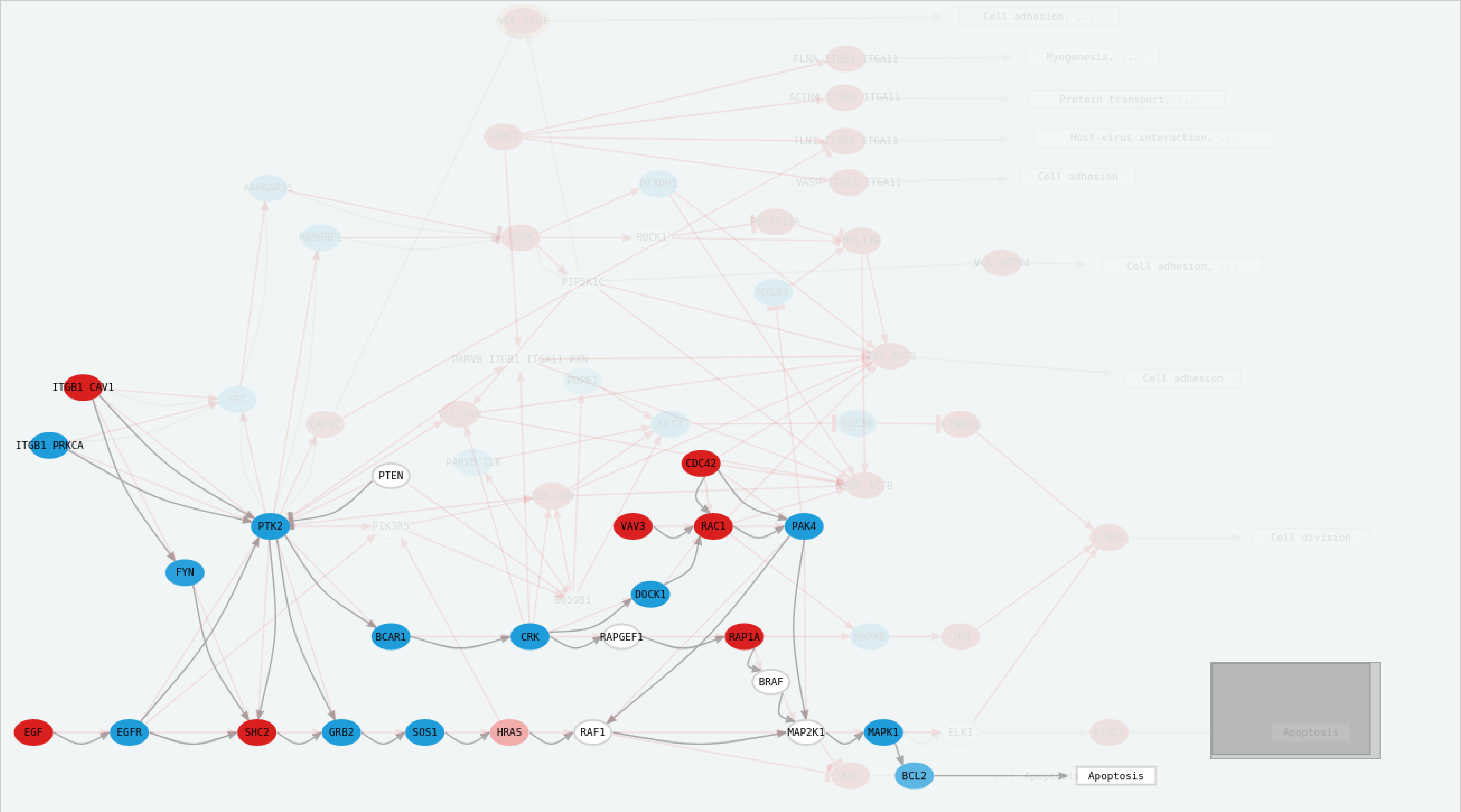

Focal adhesion (hsa04510)

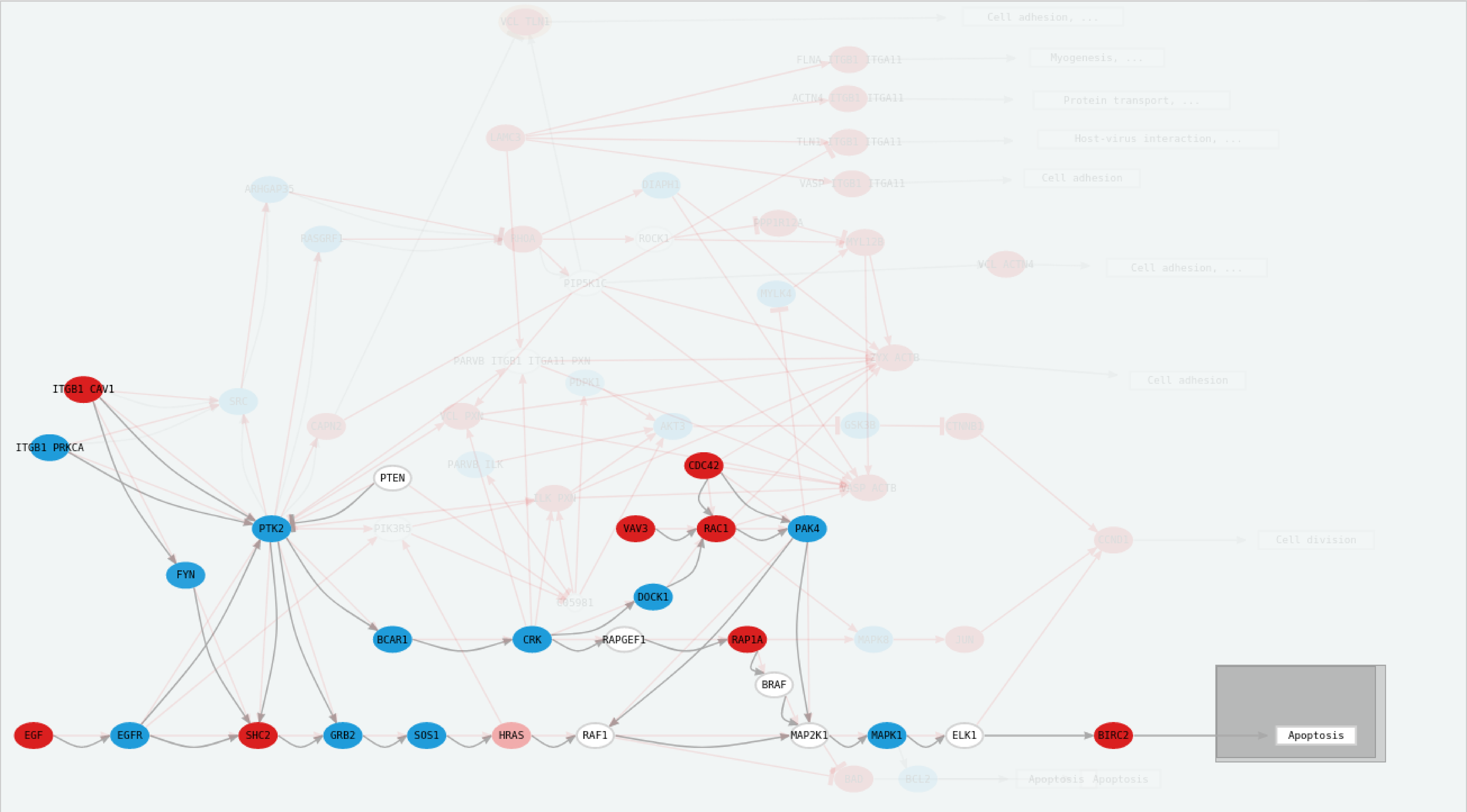

Focal adhesion (hsa04510)

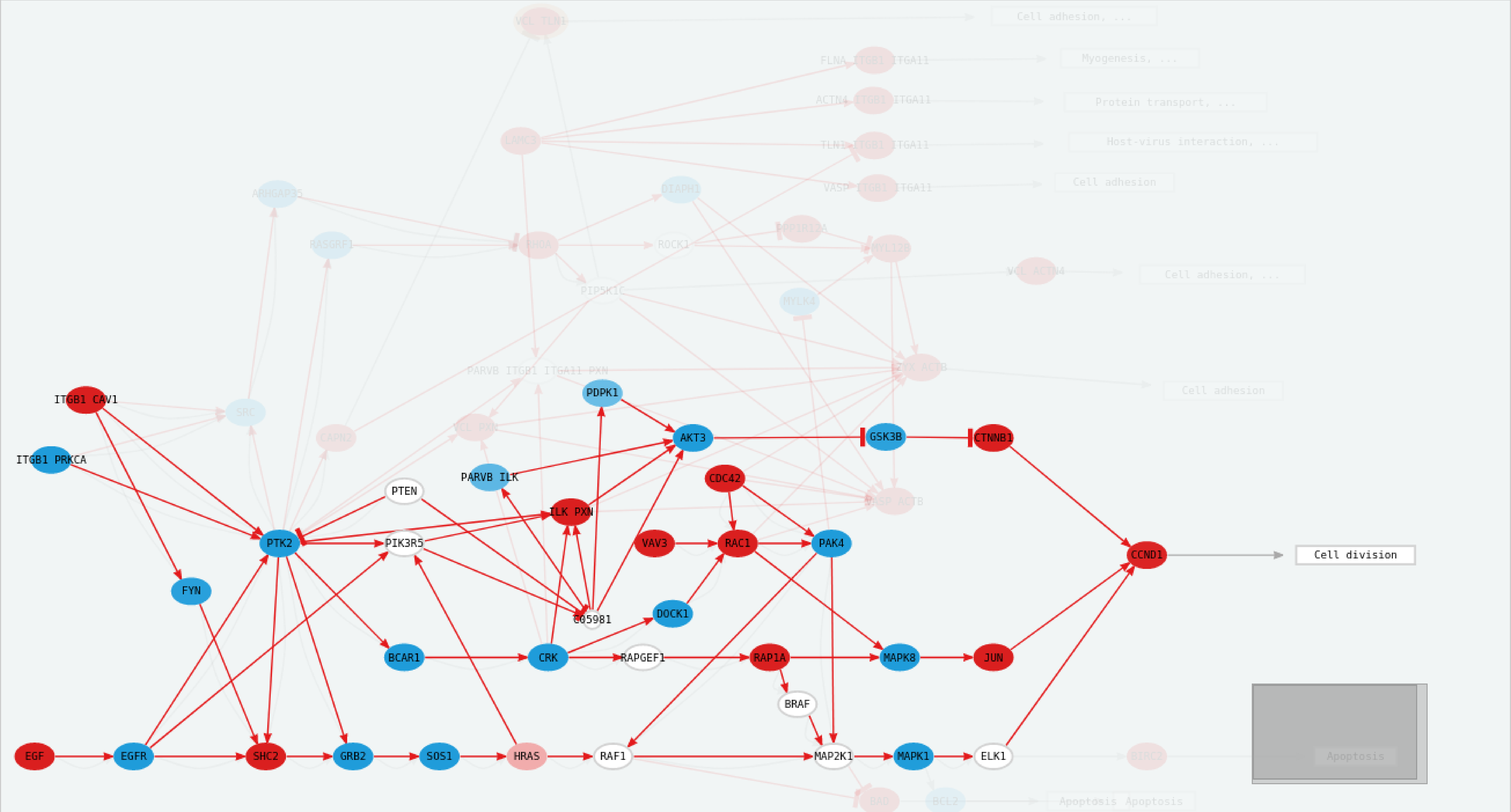

Focal adhesion (hsa04510)

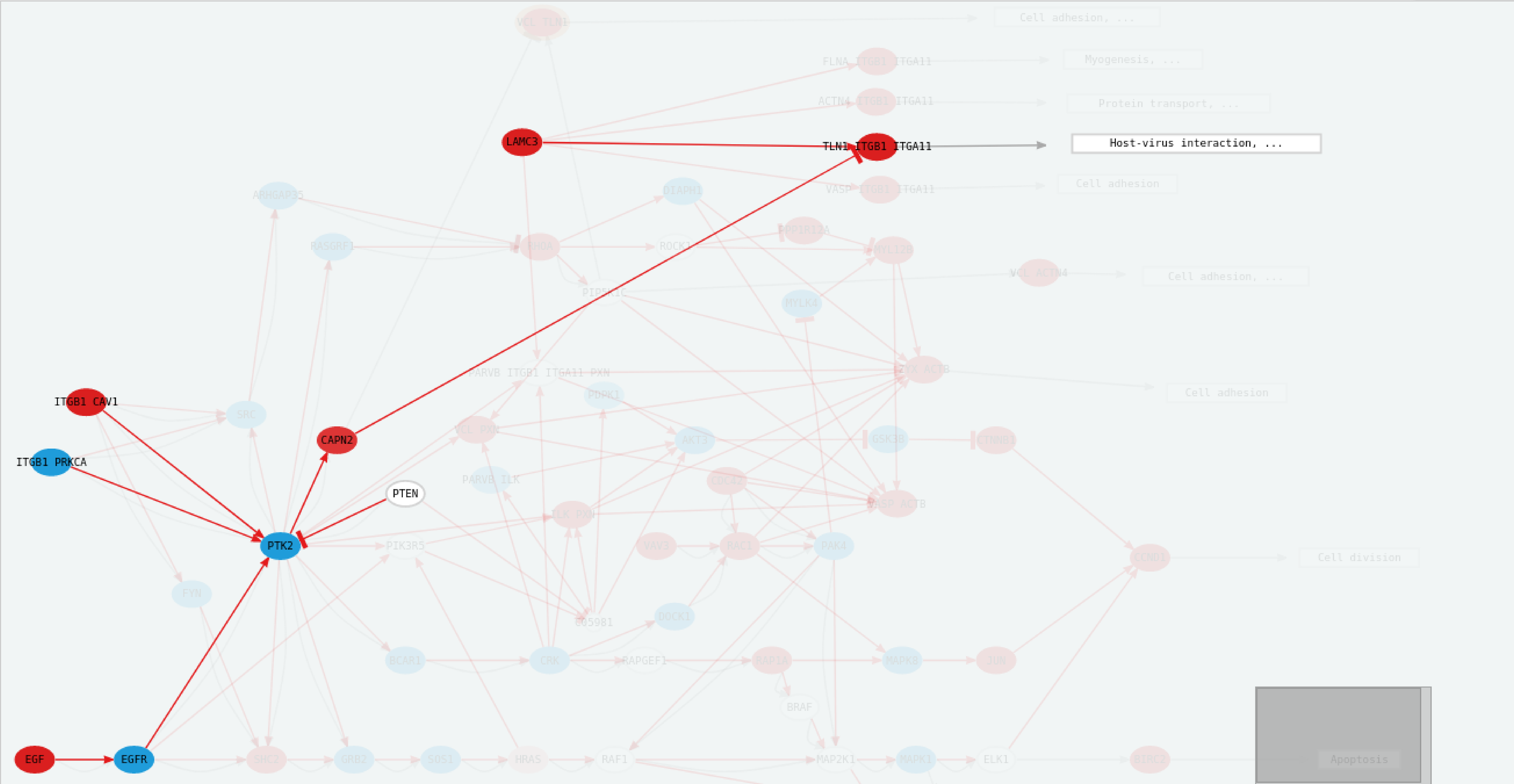

Focal adhesion (hsa04510)

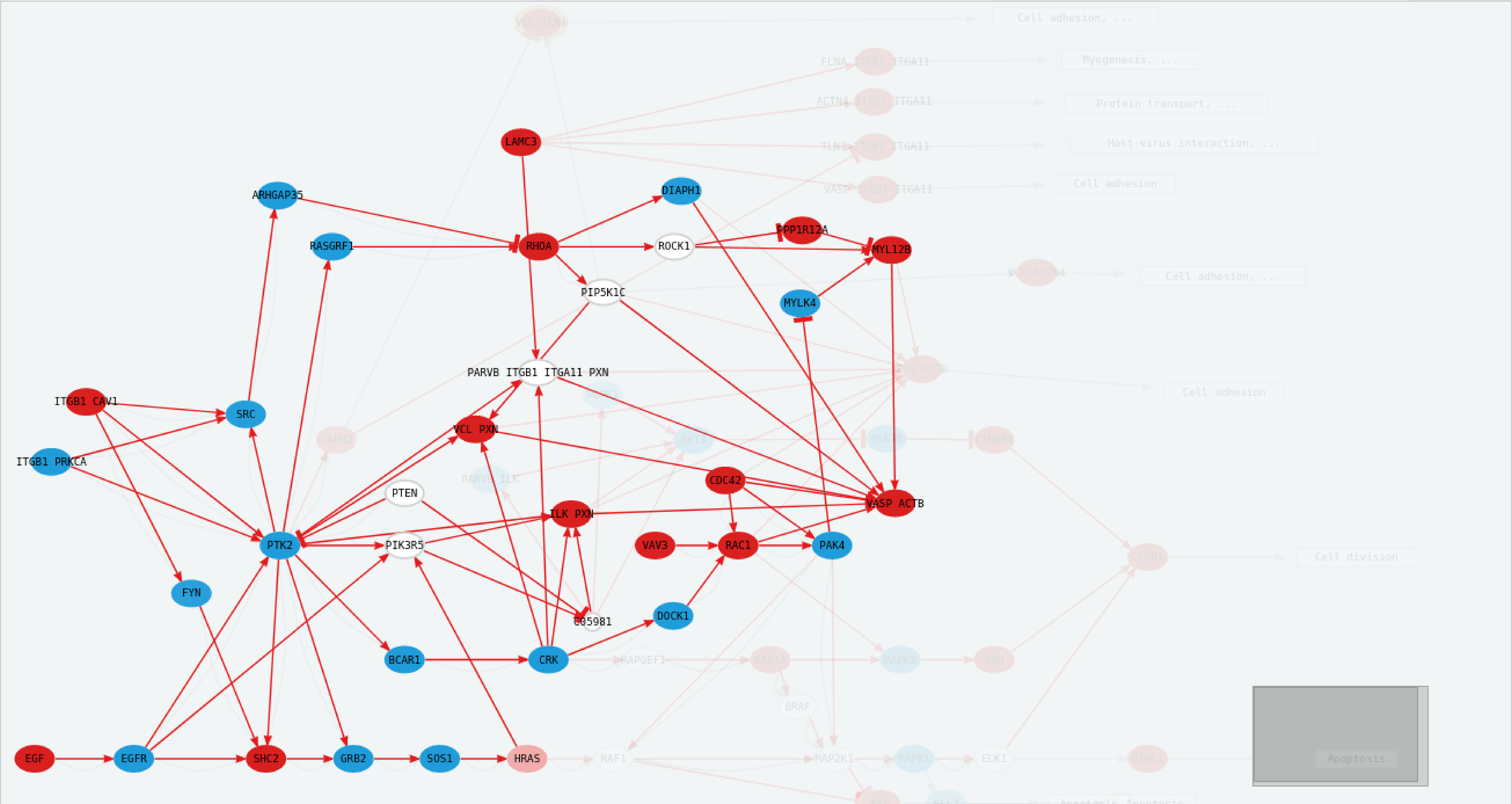

Focal adhesion (hsa04510)

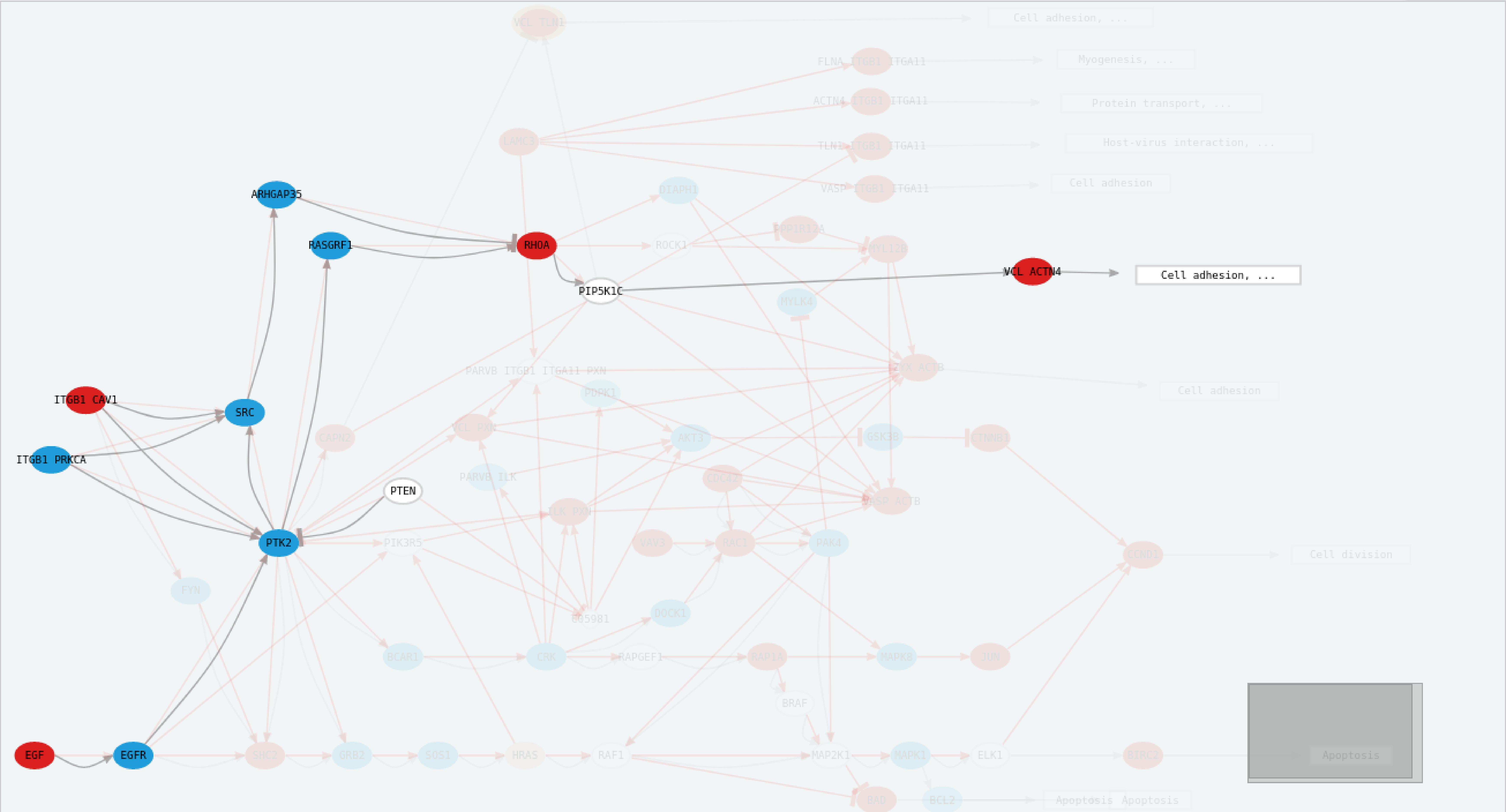

Focal adhesion (hsa04510)

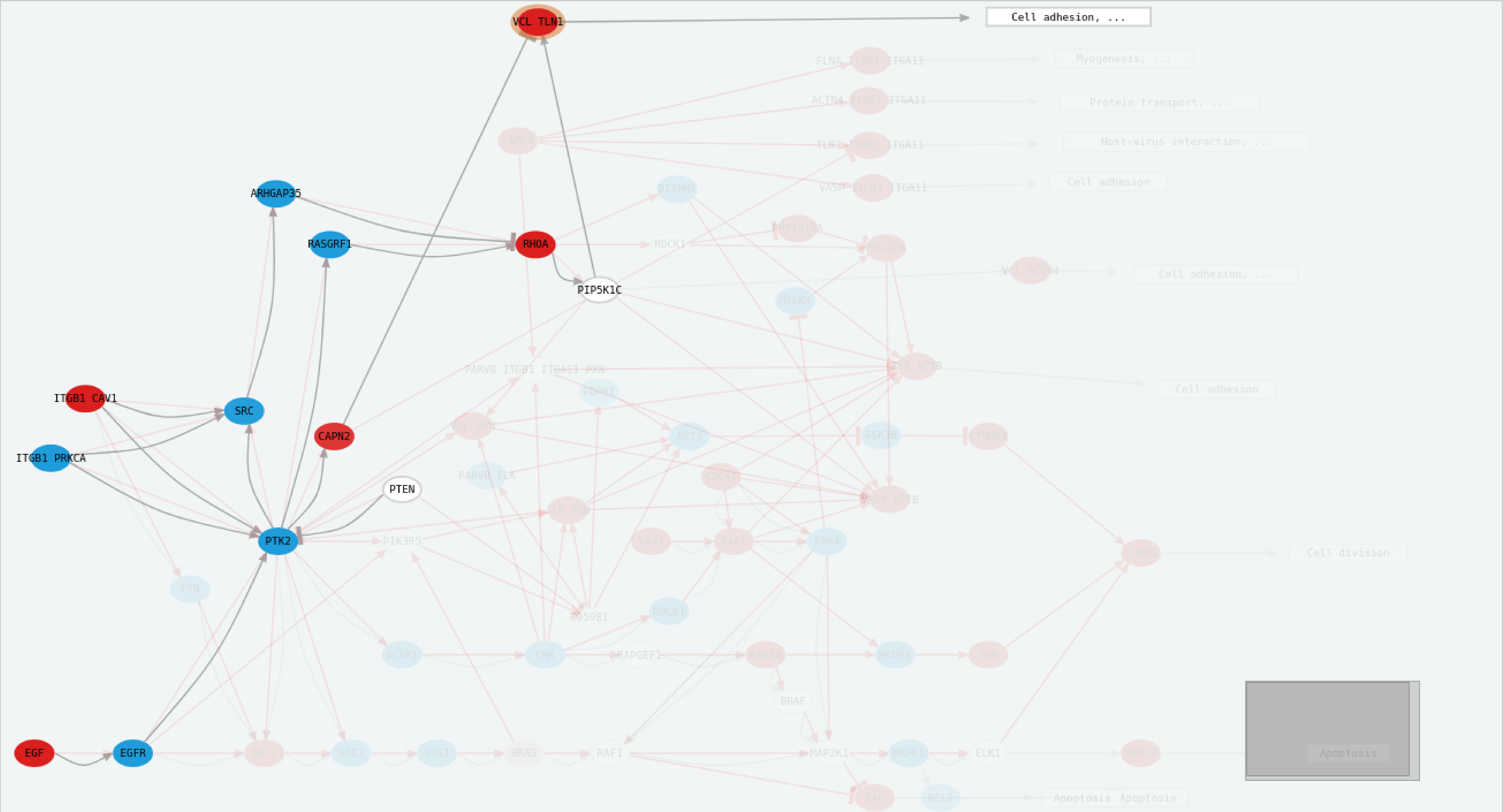

Focal adhesion (hsa04510)

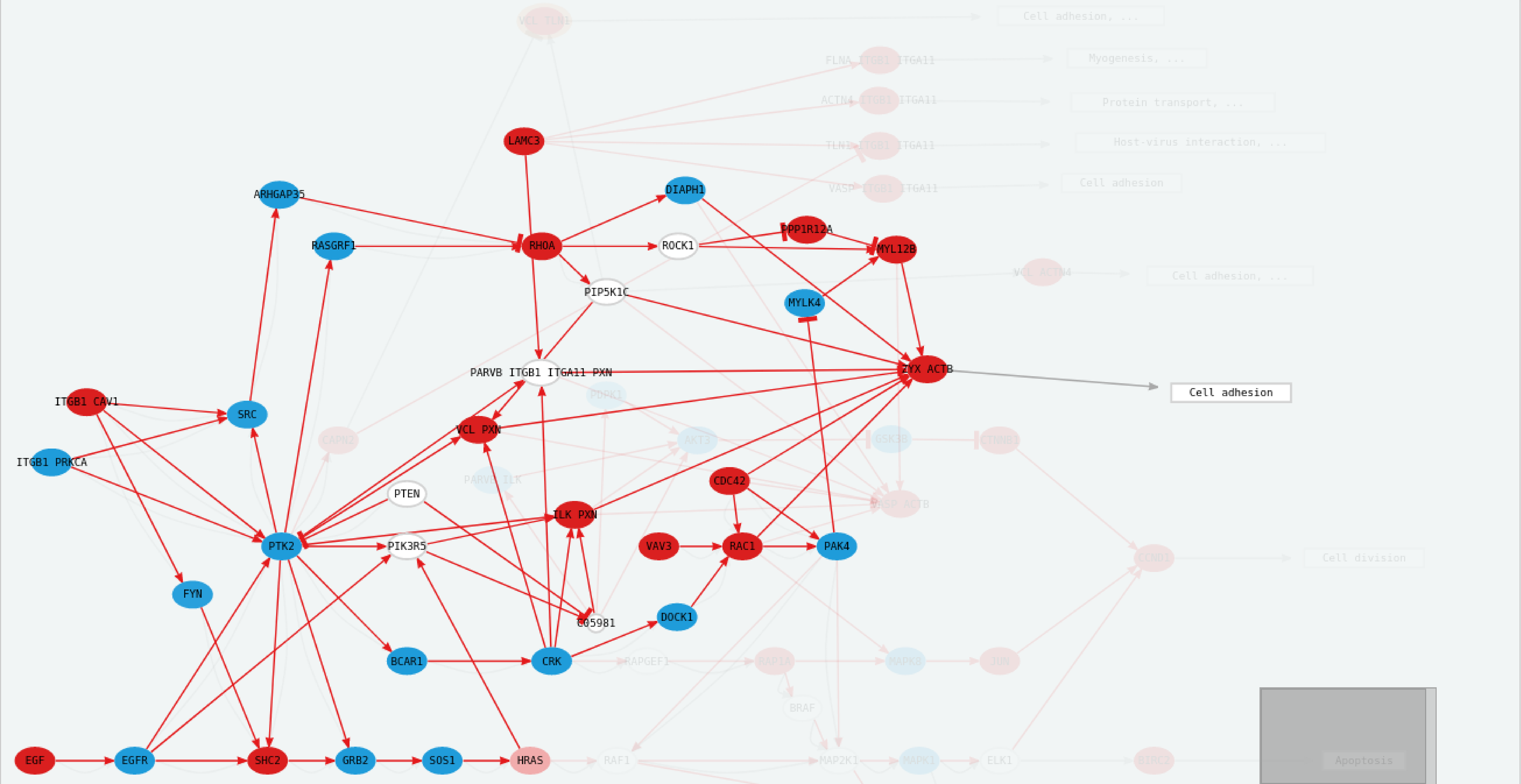

HIF-1 signaling pathway (hsa04066)

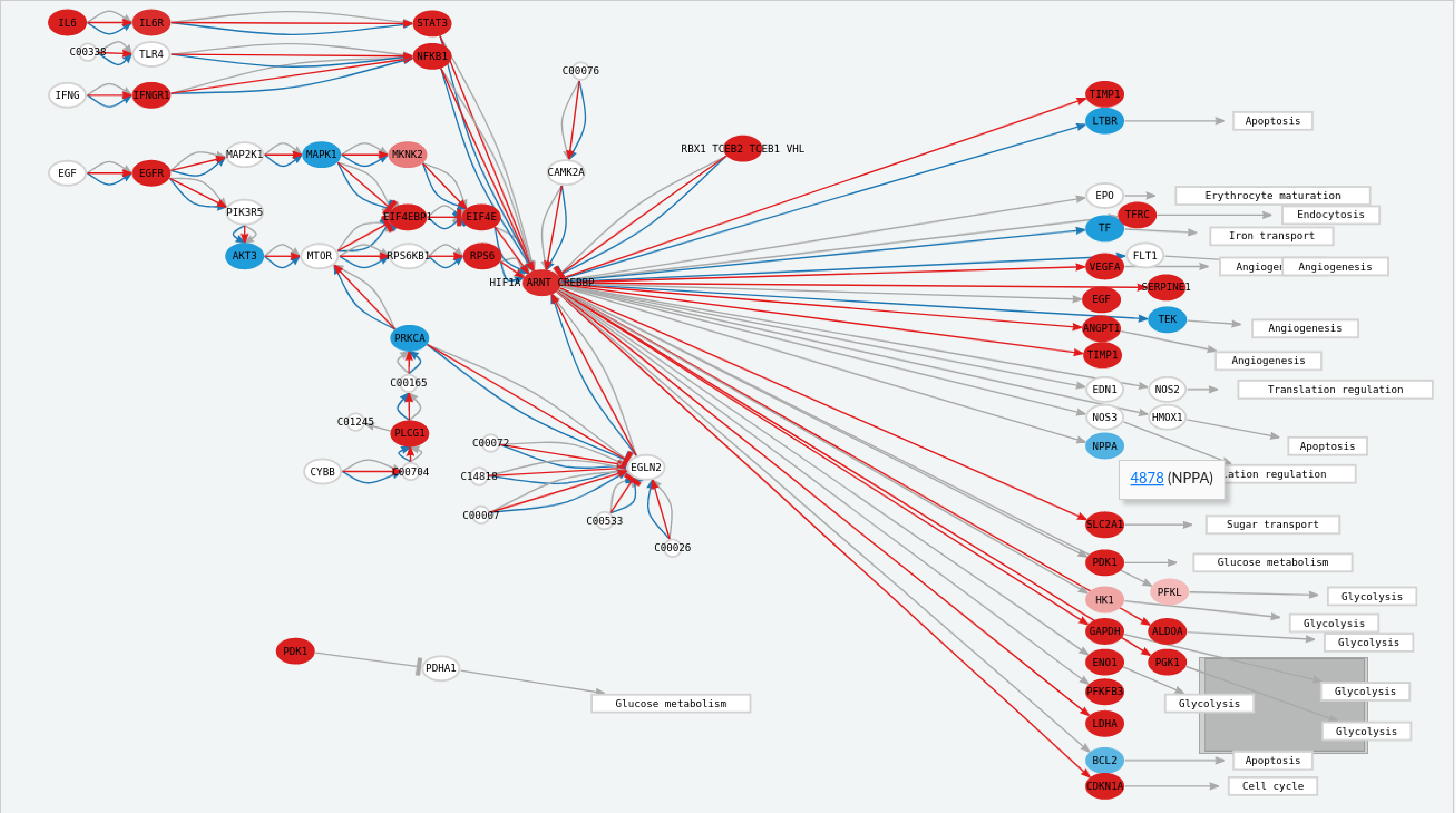

HIF-1 signaling pathway (hsa04066)

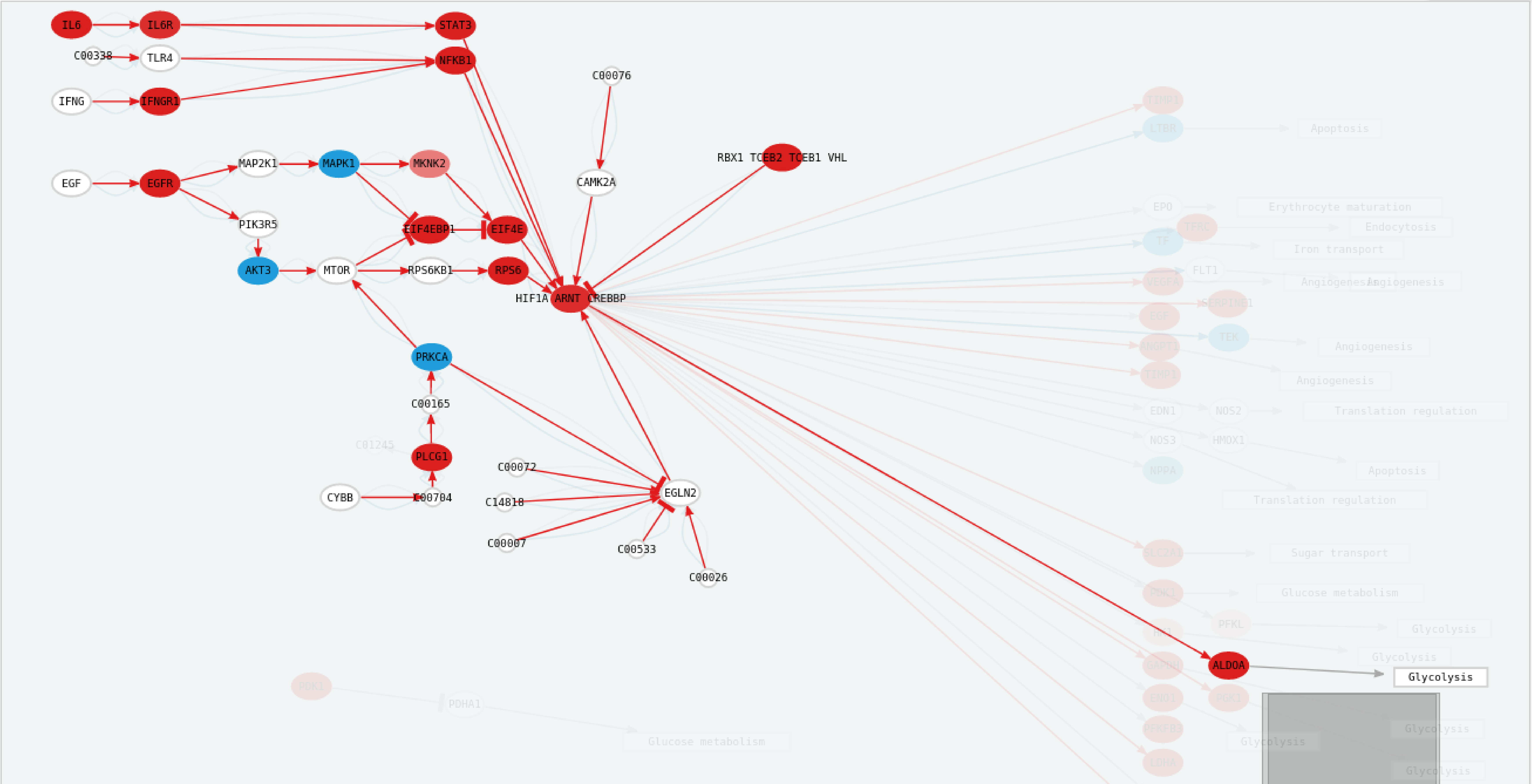

HIF-1 signaling pathway (hsa04066)

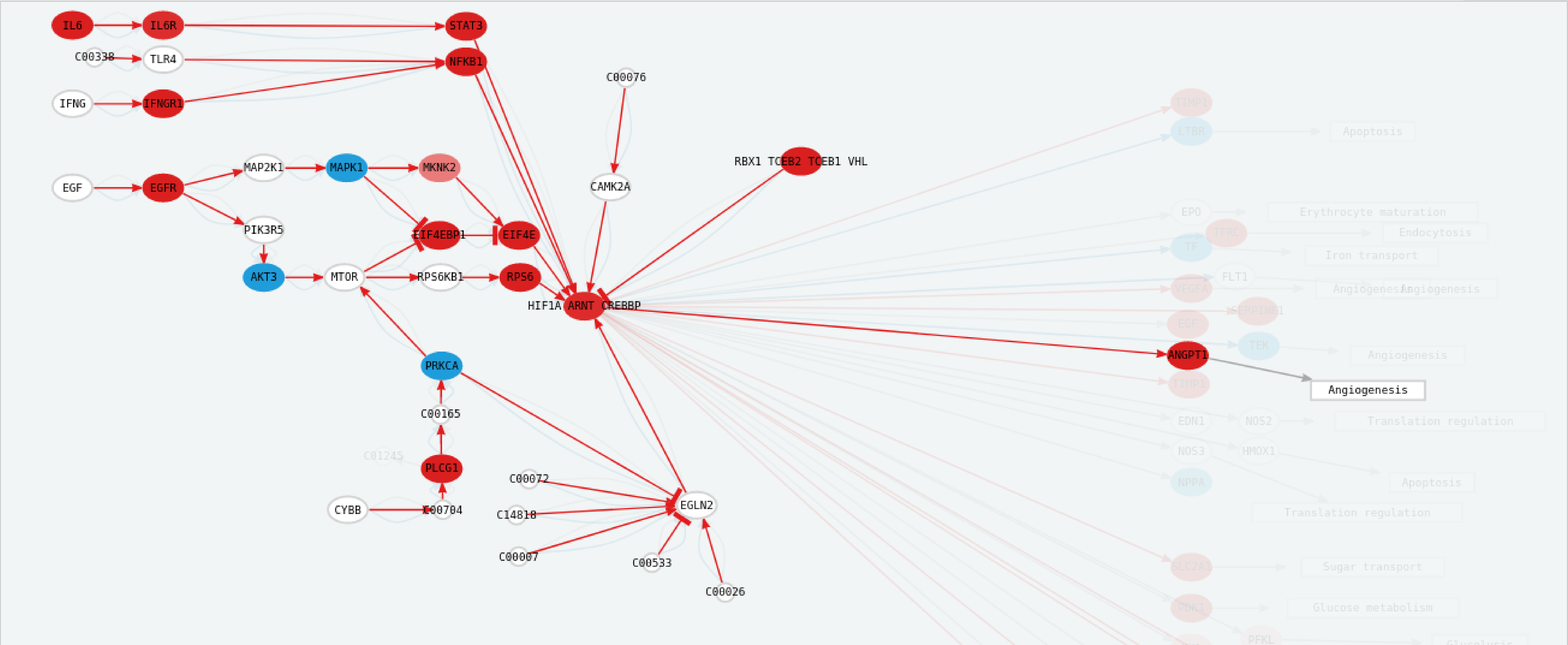

HIF-1 signaling pathway (hsa04066)

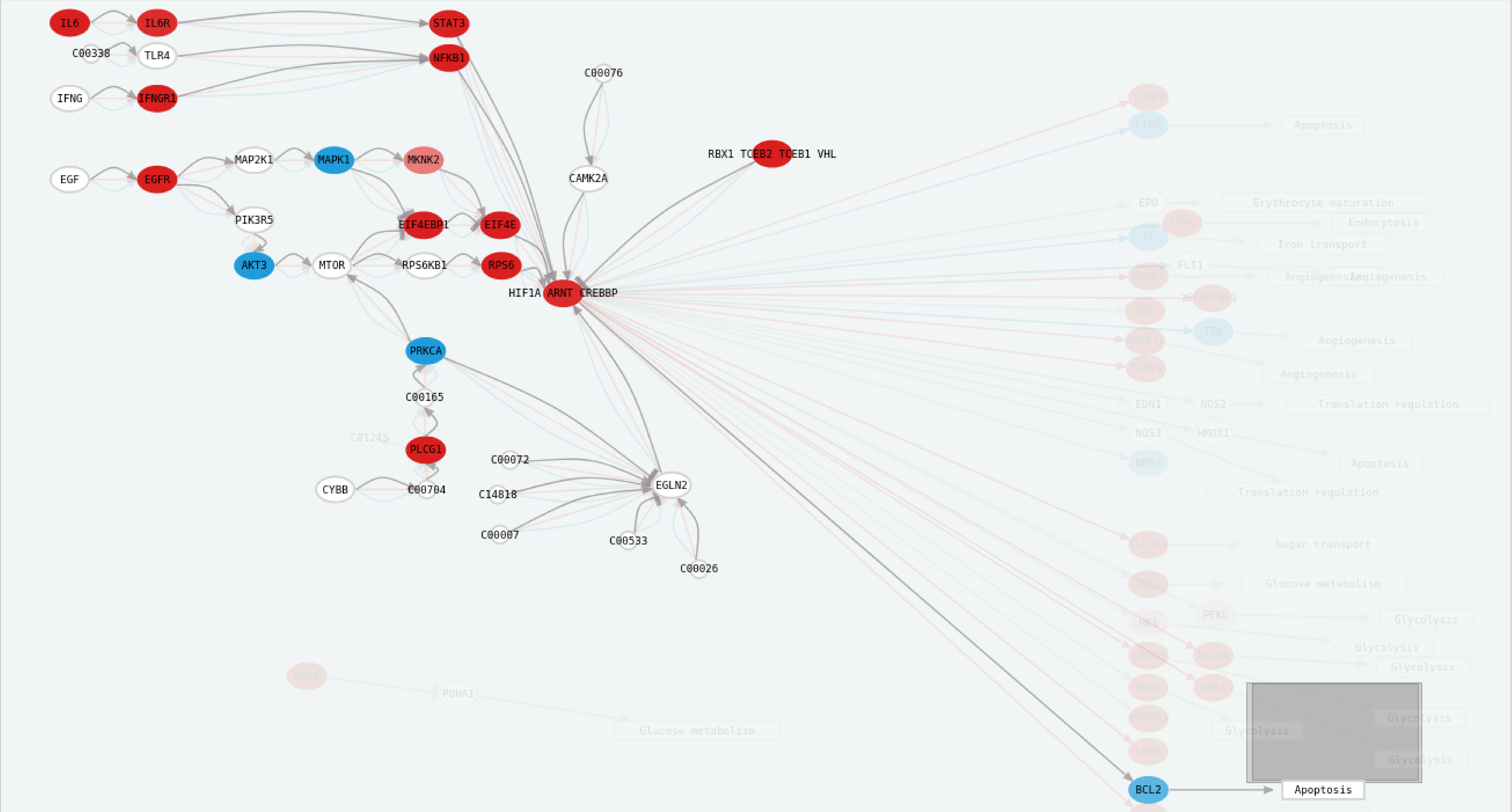

HIF-1 signaling pathway (hsa04066)

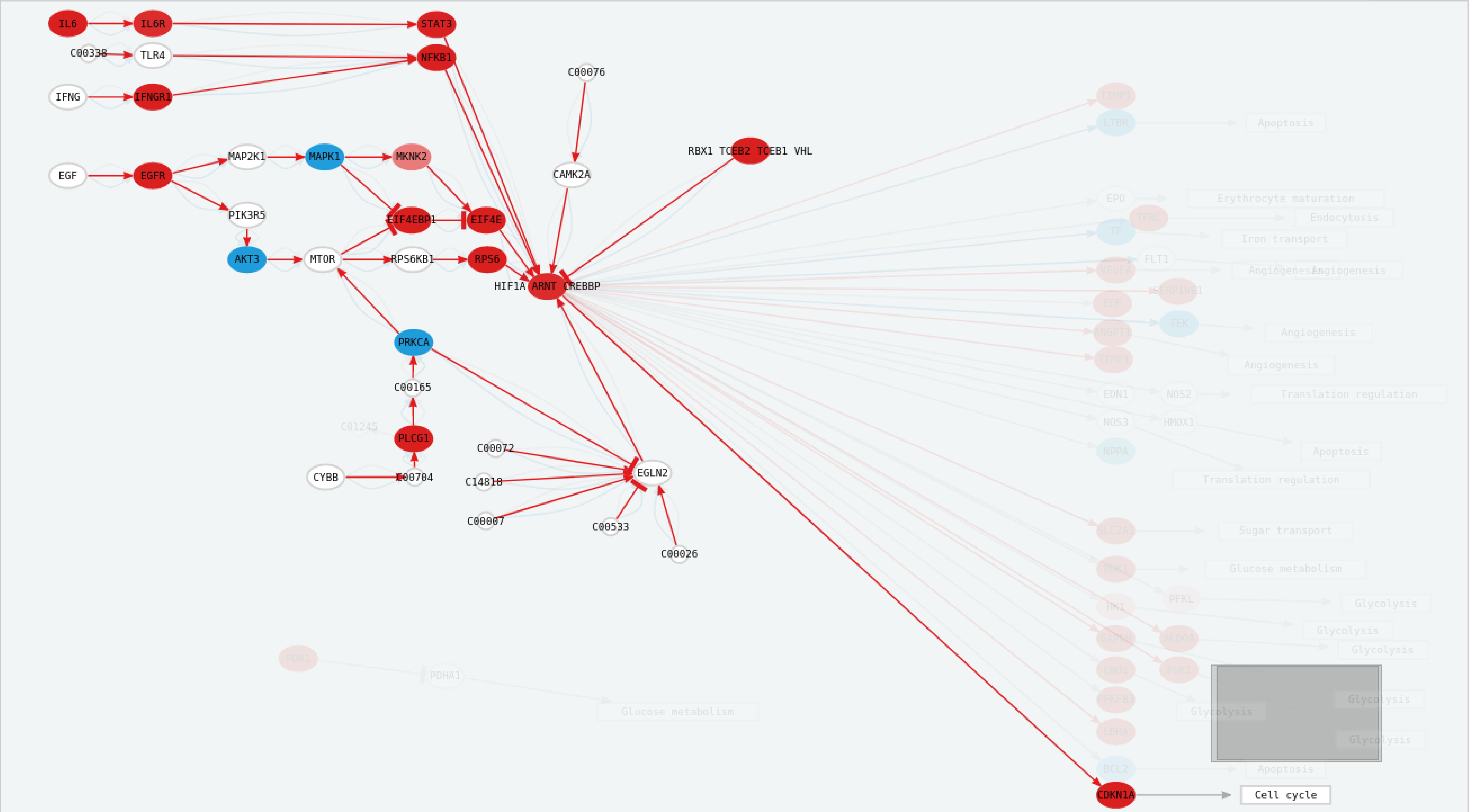

HIF-1 signaling pathway (hsa04066)

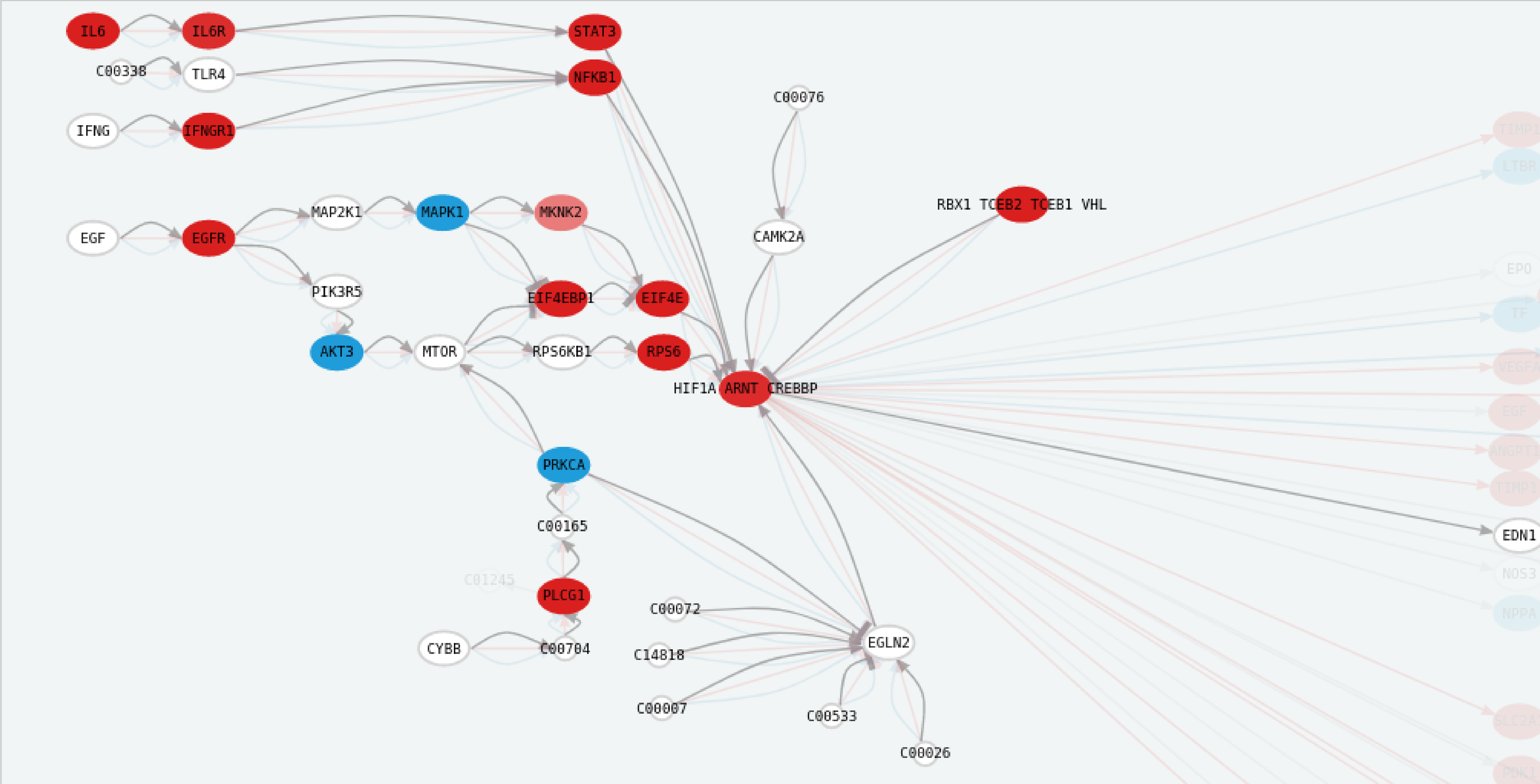

HIF-1 signaling pathway (hsa04066)

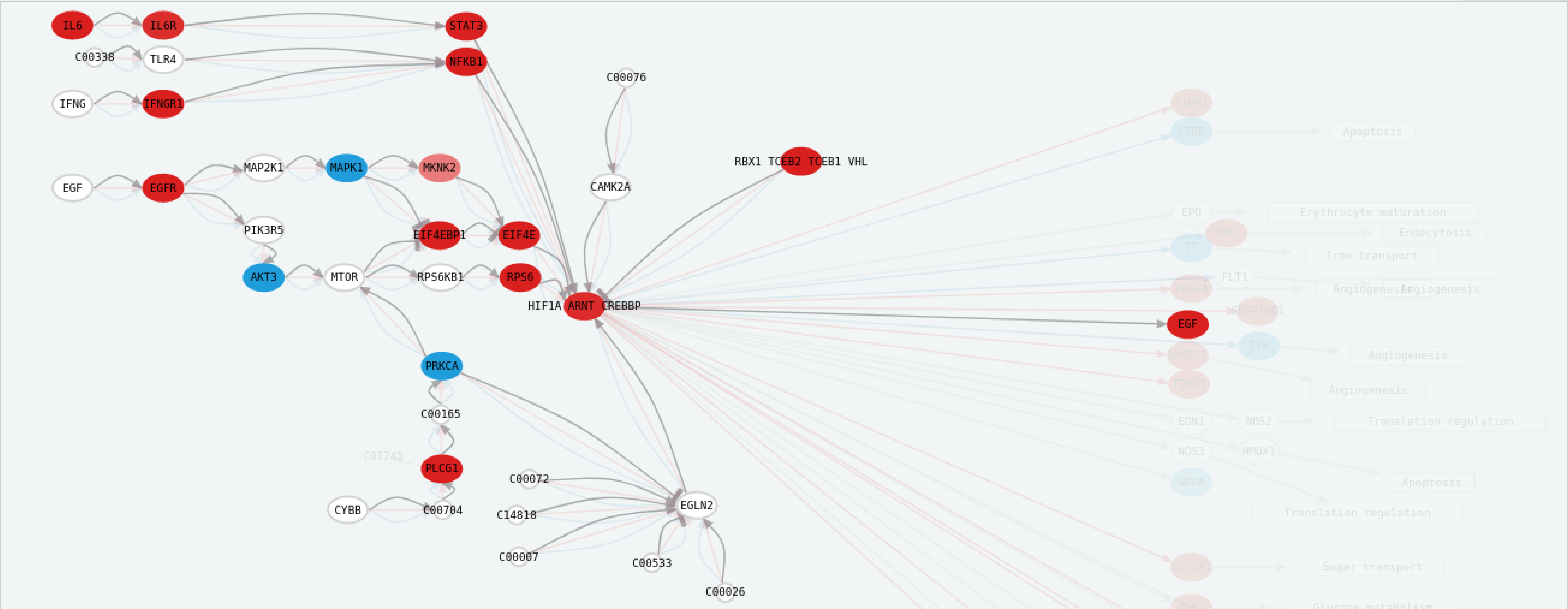

HIF-1 signaling pathway (hsa04066)

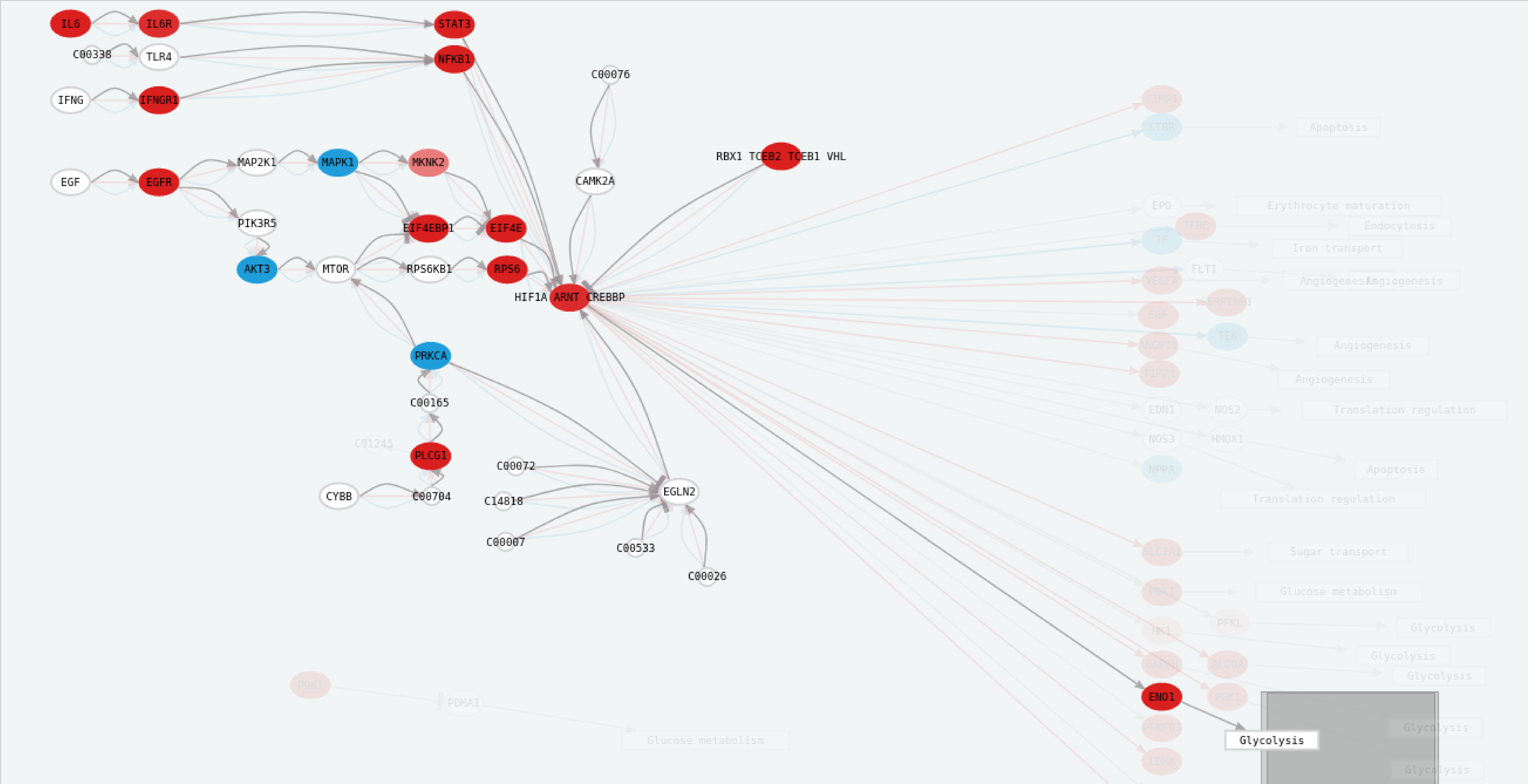

HIF-1 signaling pathway (hsa04066)

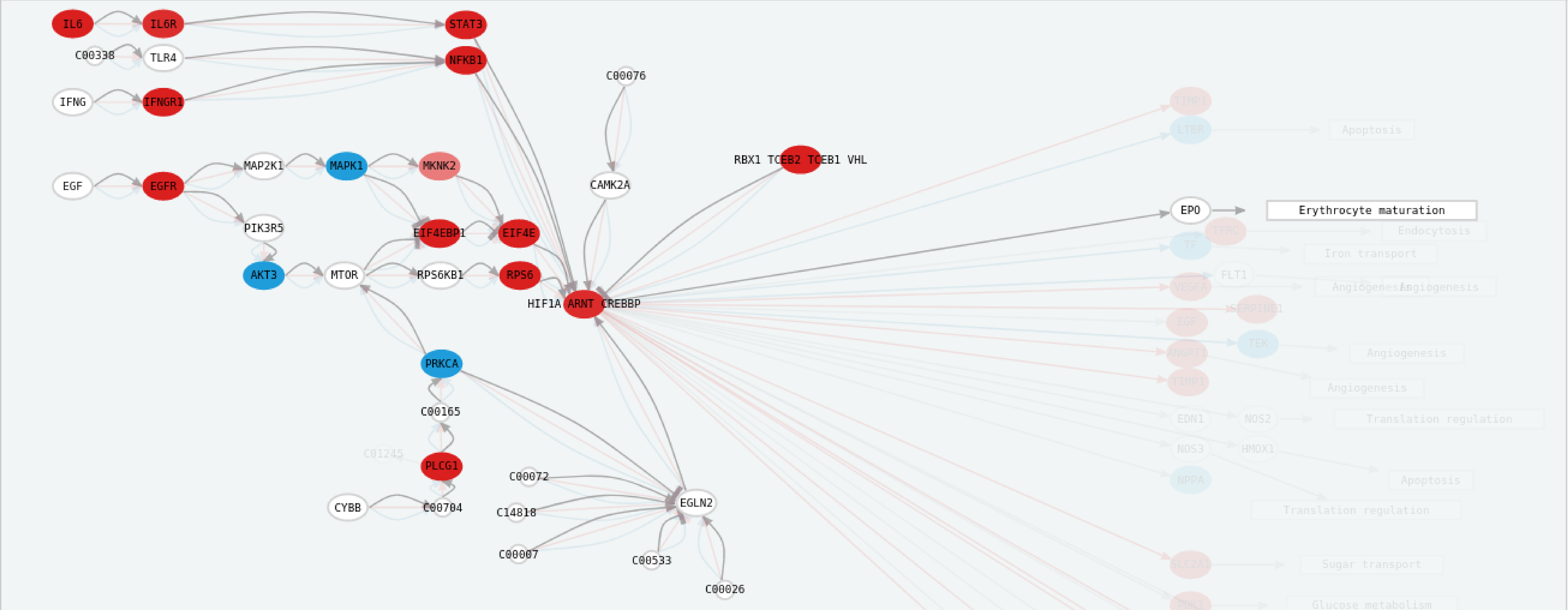

HIF-1 signaling pathway (hsa04066)

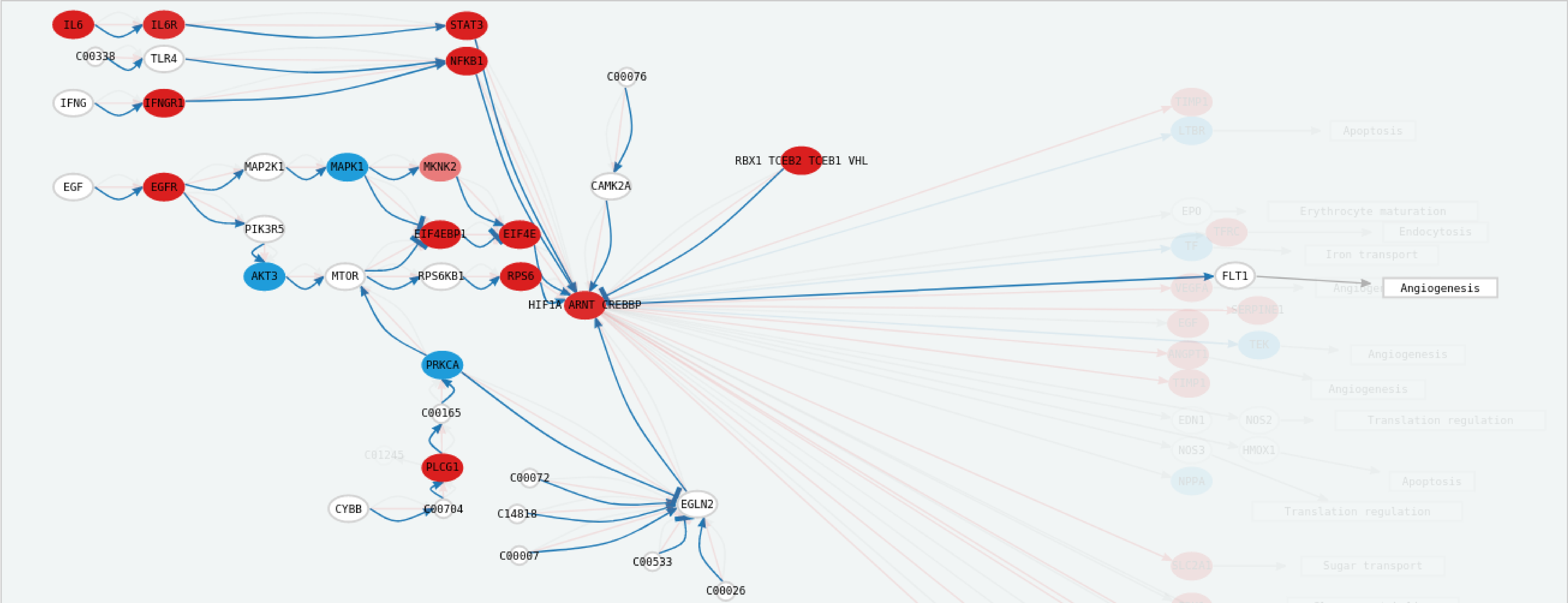

HIF-1 signaling pathway (hsa04066)

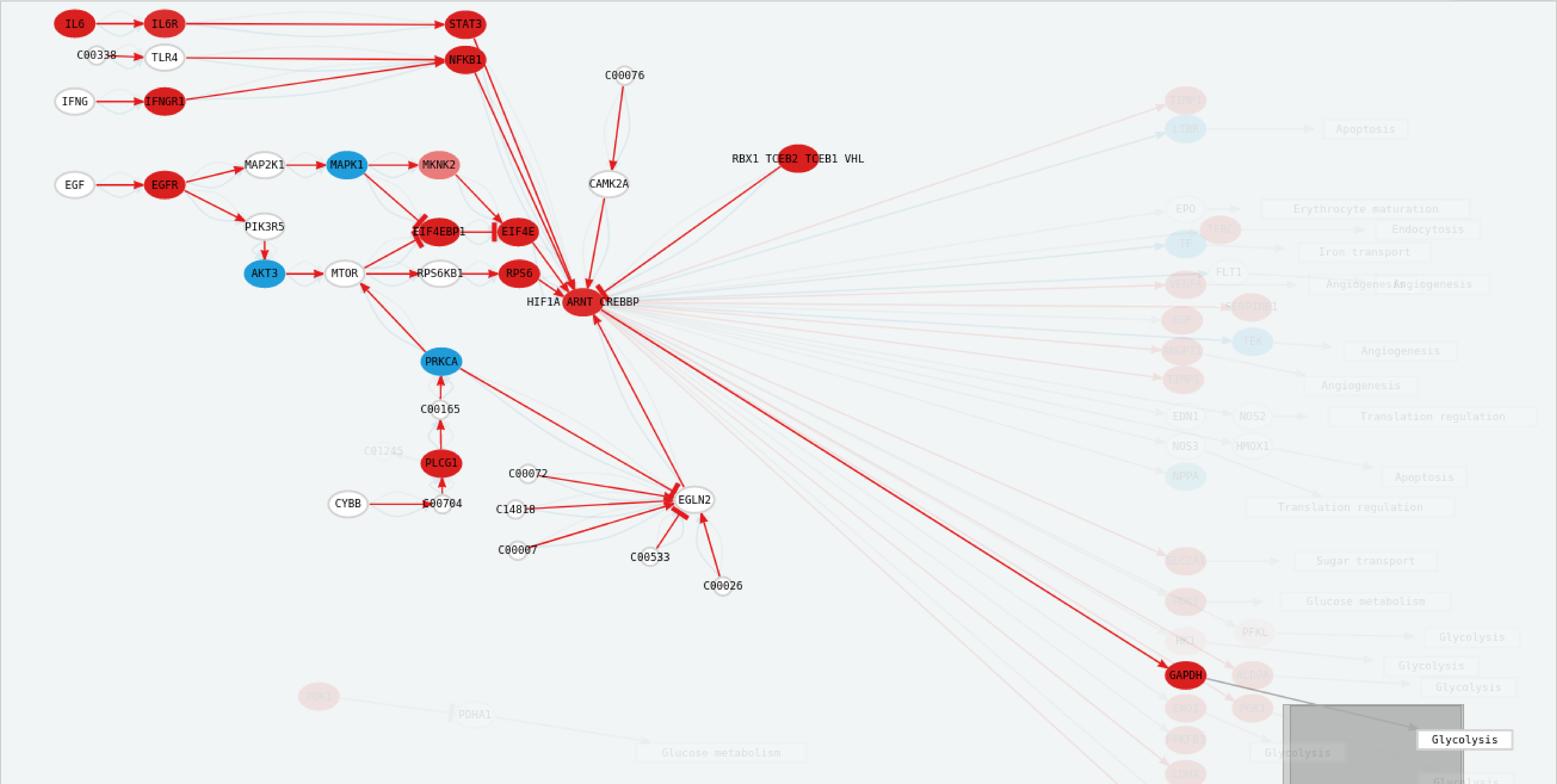

HIF-1 signaling pathway (hsa04066)

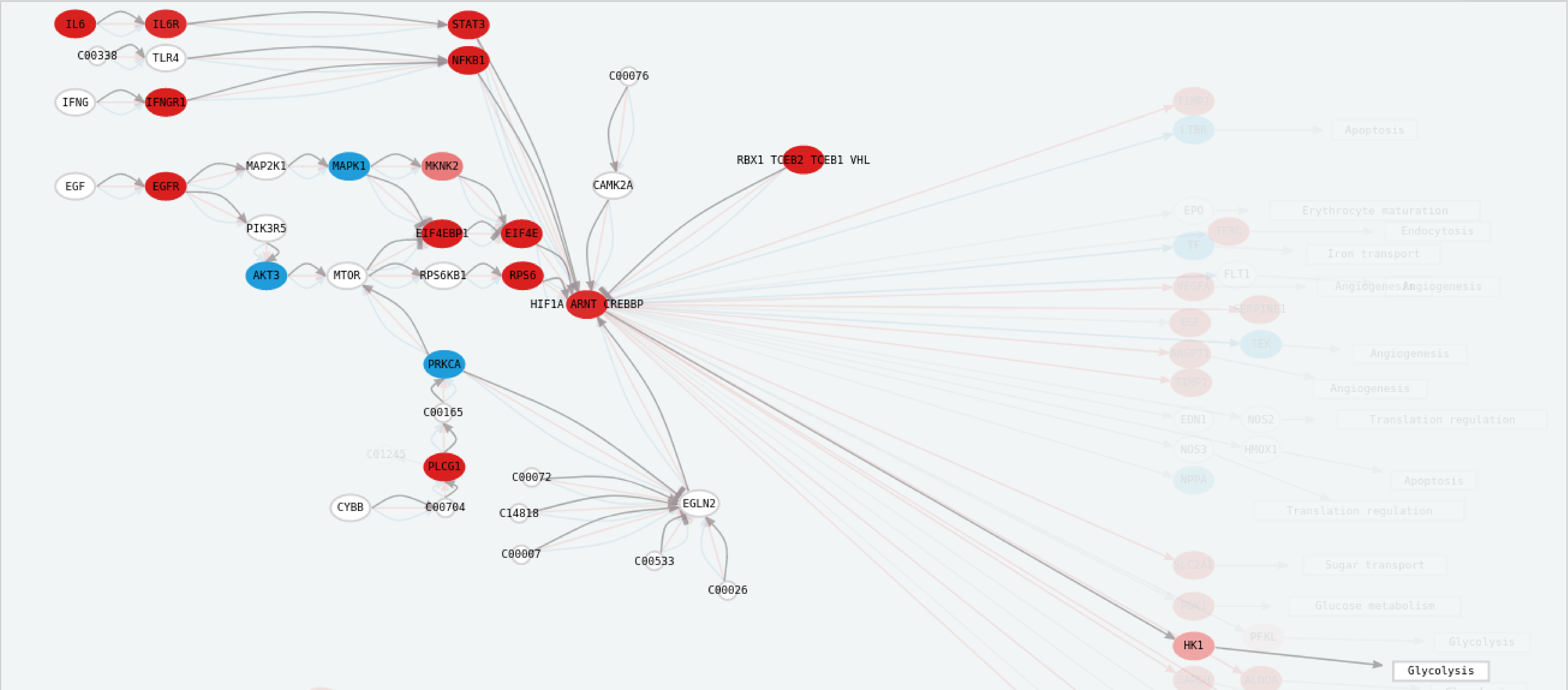

HIF-1 signaling pathway (hsa04066)

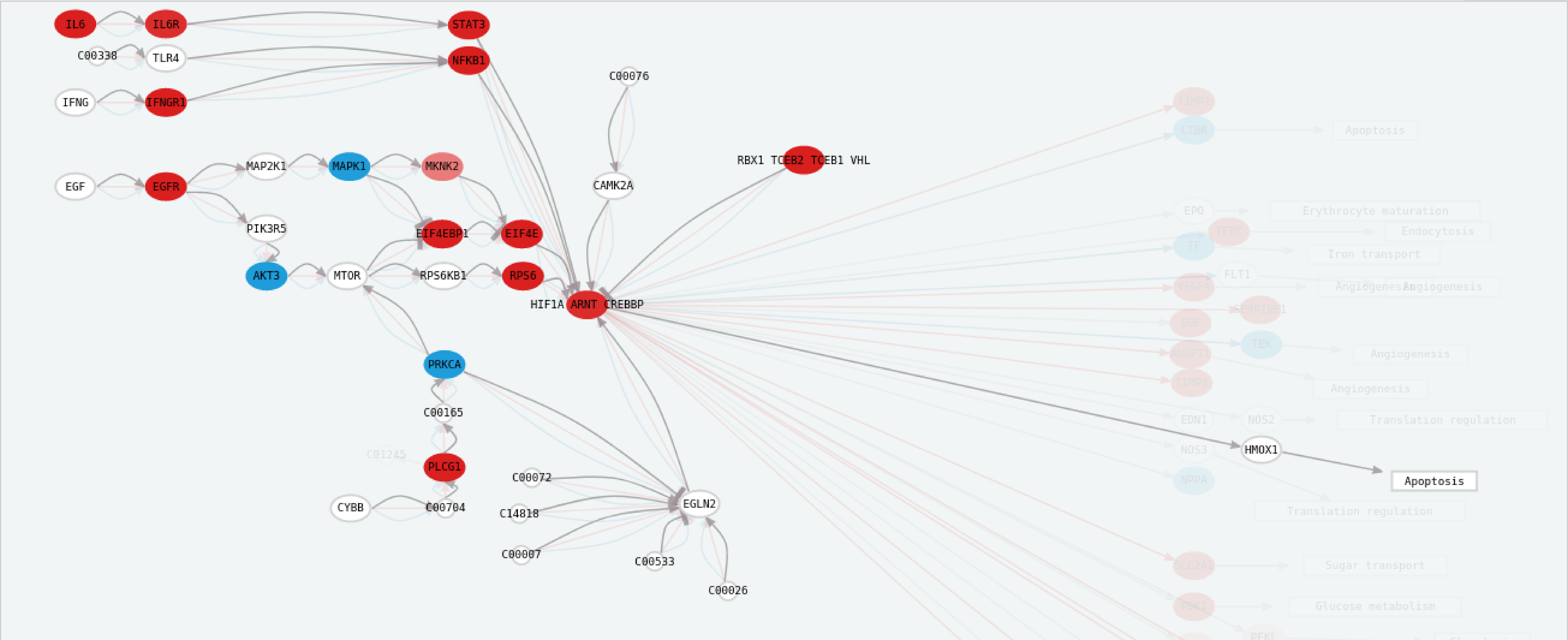

HIF-1 signaling pathway (hsa04066)

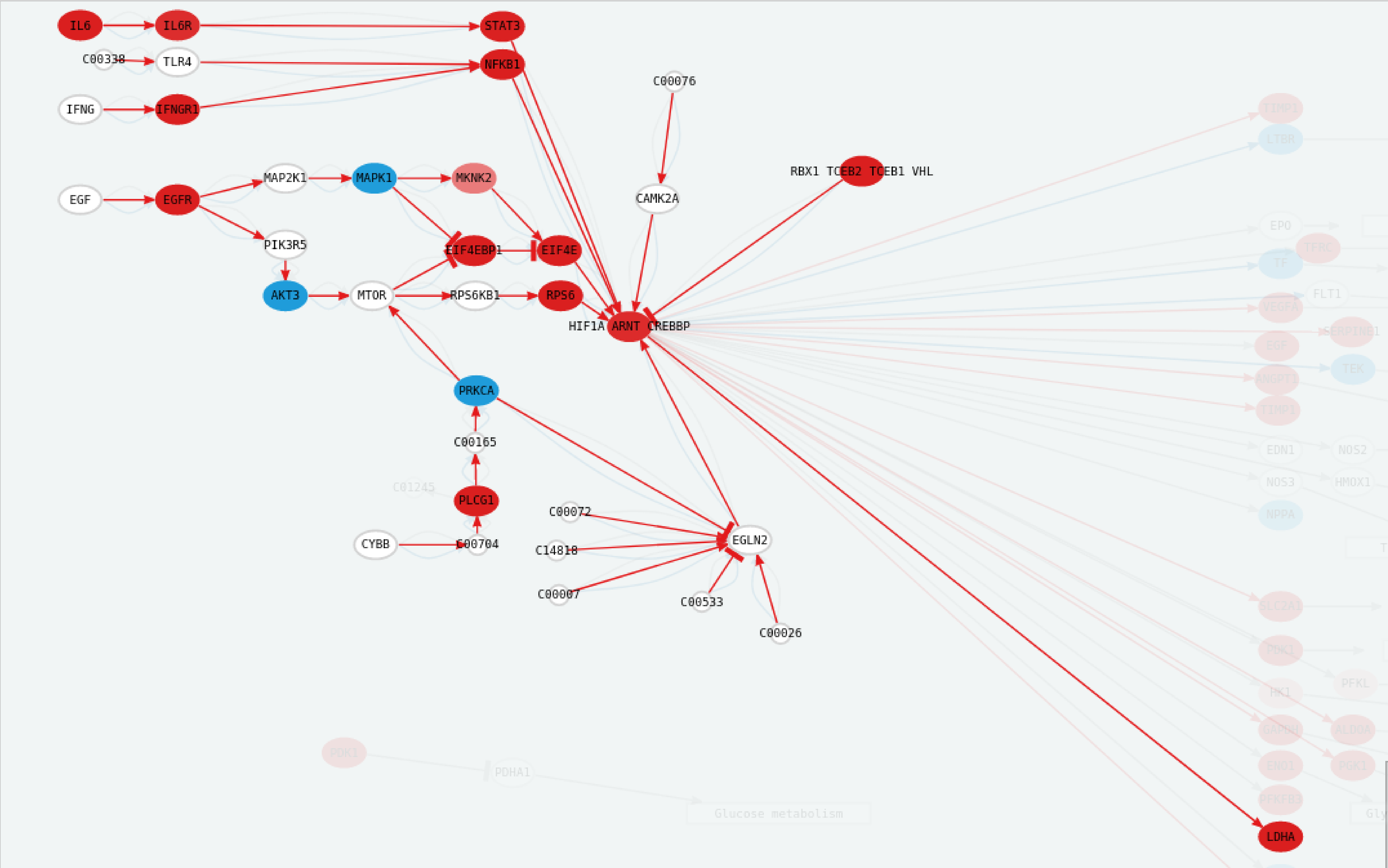

HIF-1 signaling pathway (hsa04066)

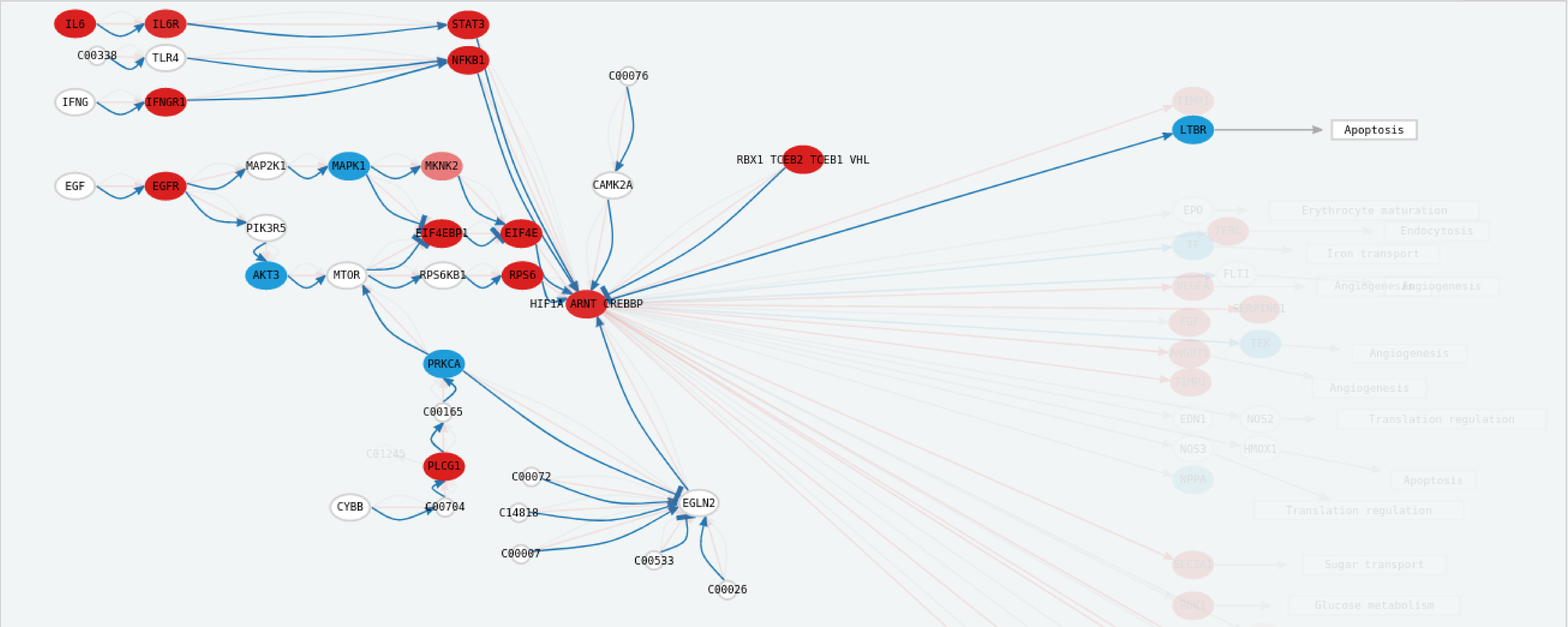

HIF-1 signaling pathway (hsa04066)

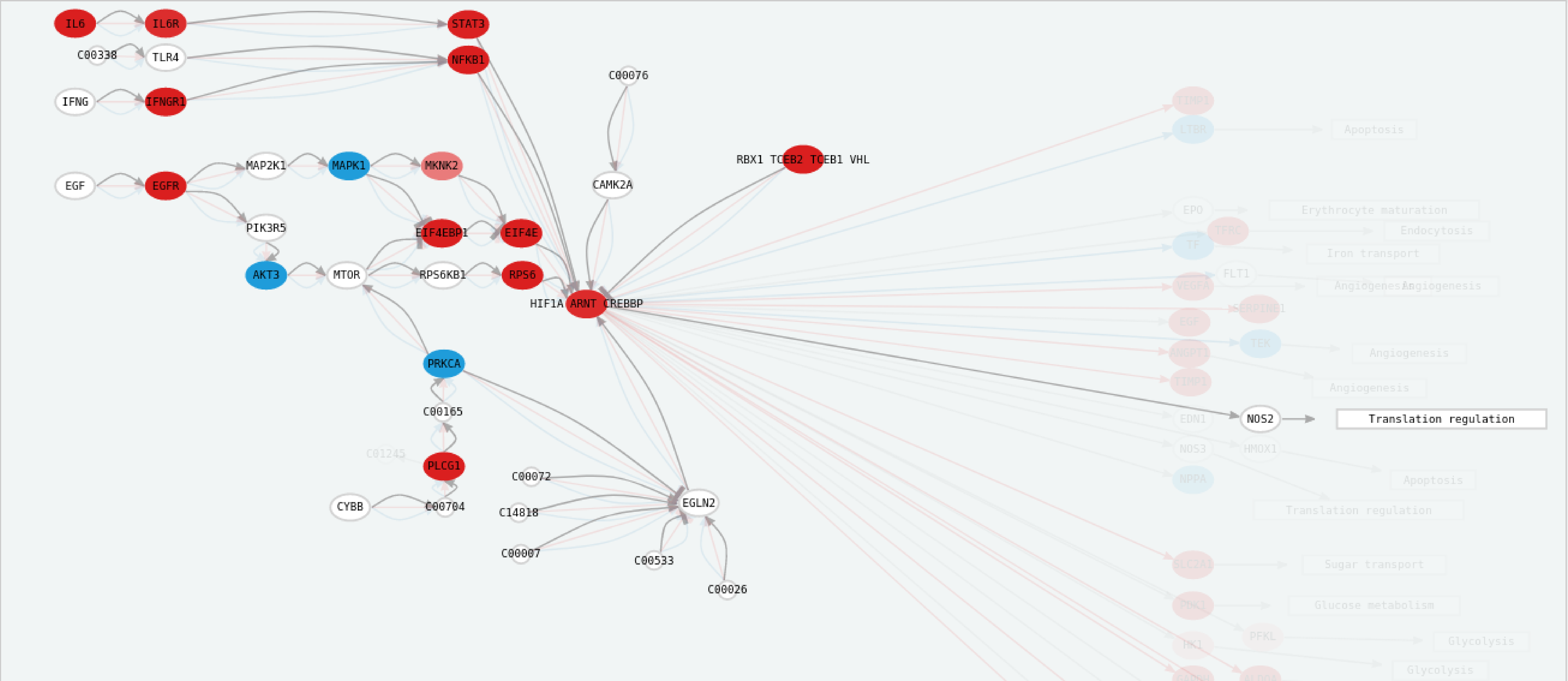

HIF-1 signaling pathway (hsa04066)

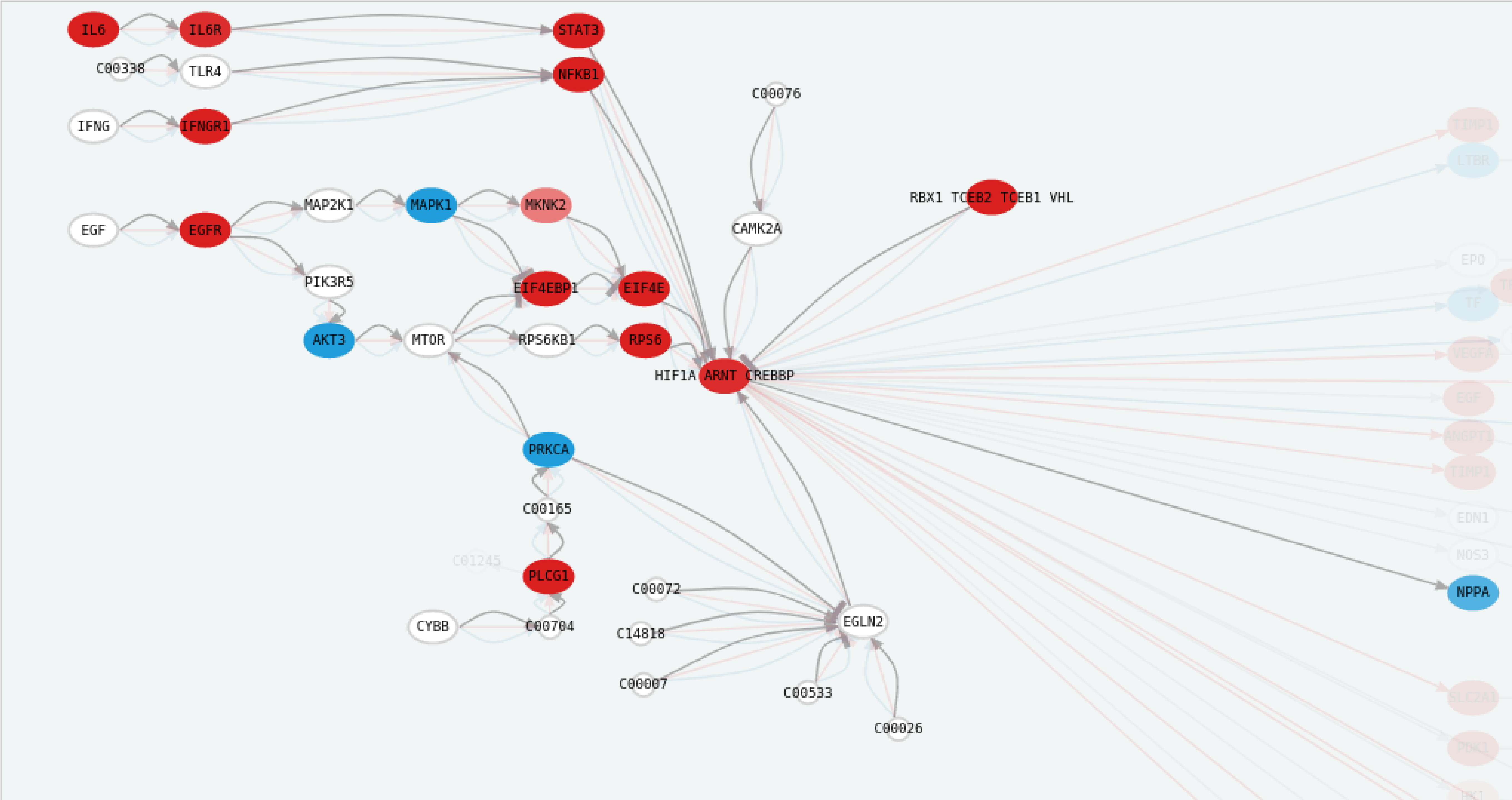

HIF-1 signaling pathway (hsa04066)

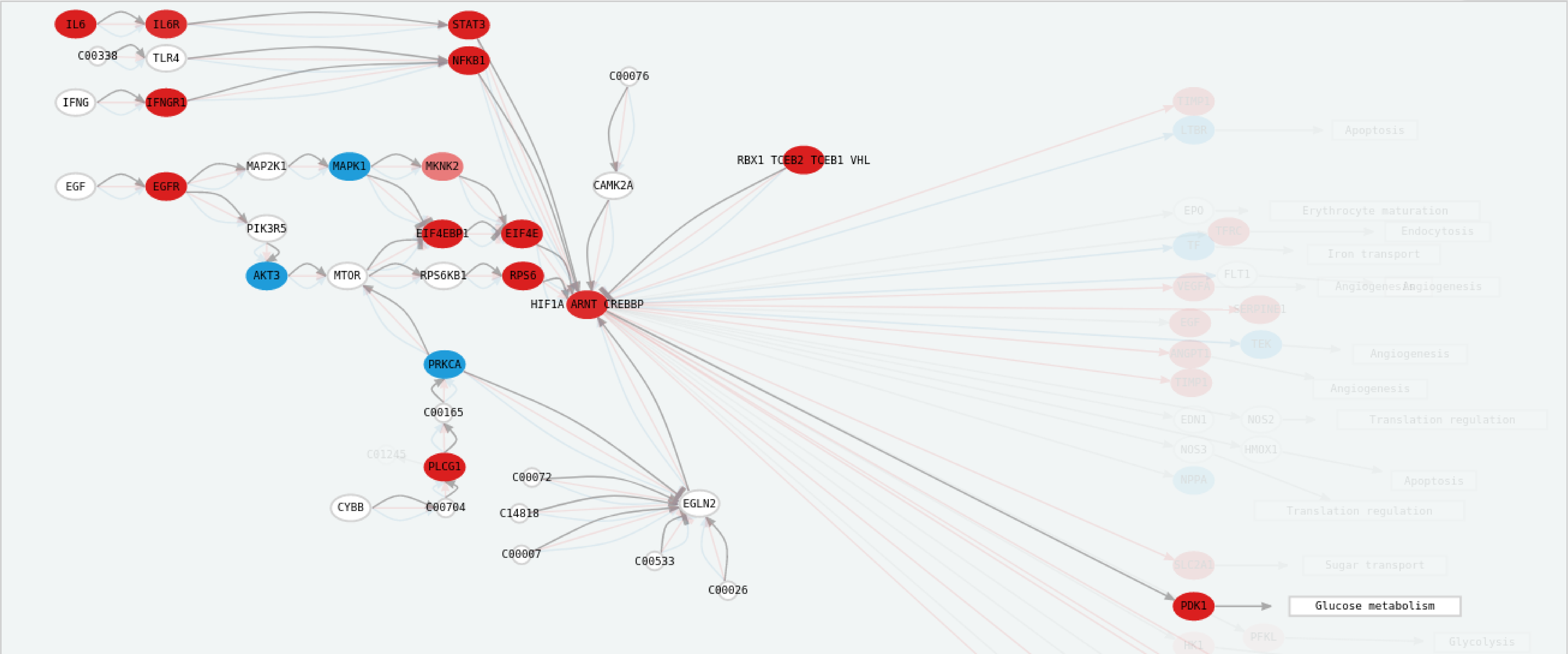

HIF-1 signaling pathway (hsa04066)

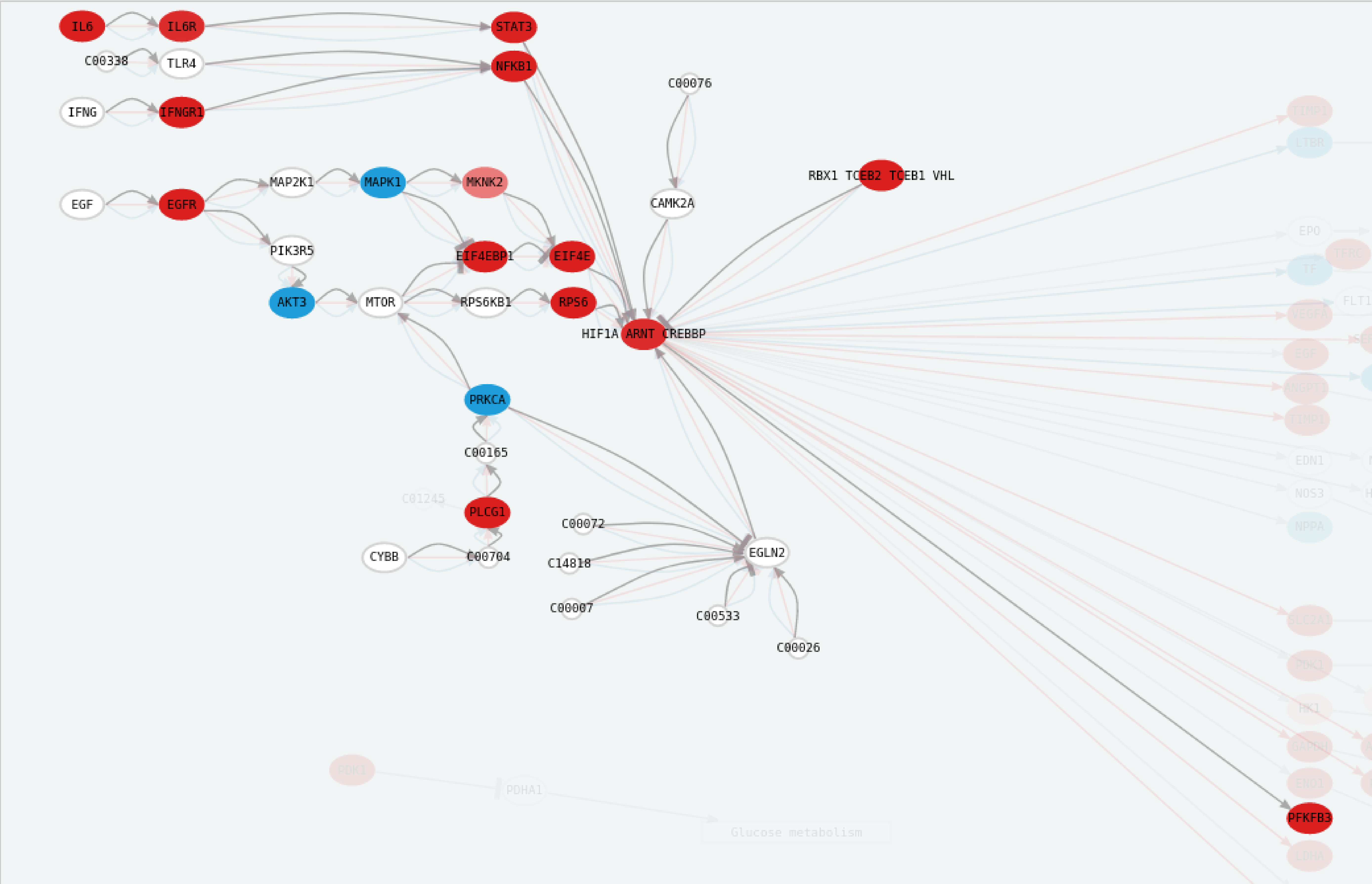

HIF-1 signaling pathway (hsa04066)

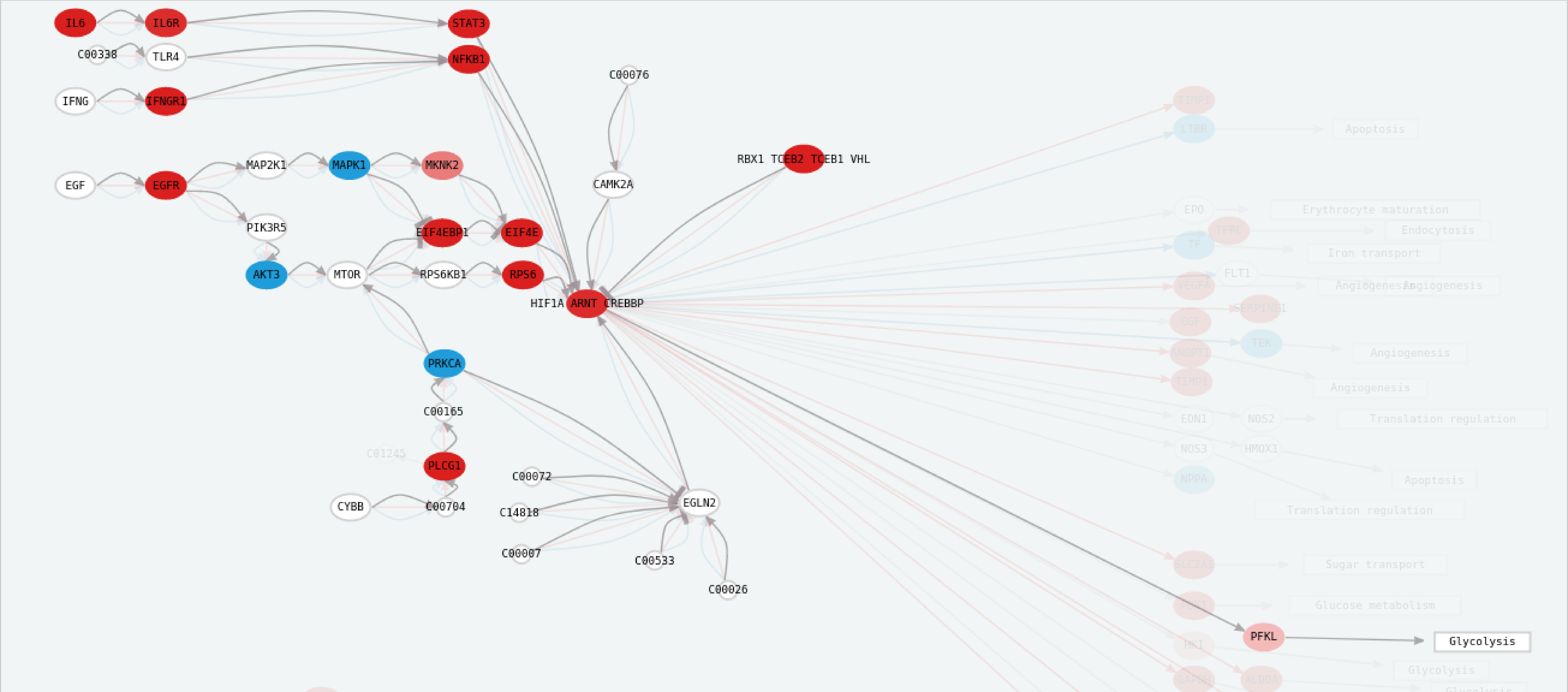

HIF-1 signaling pathway (hsa04066)

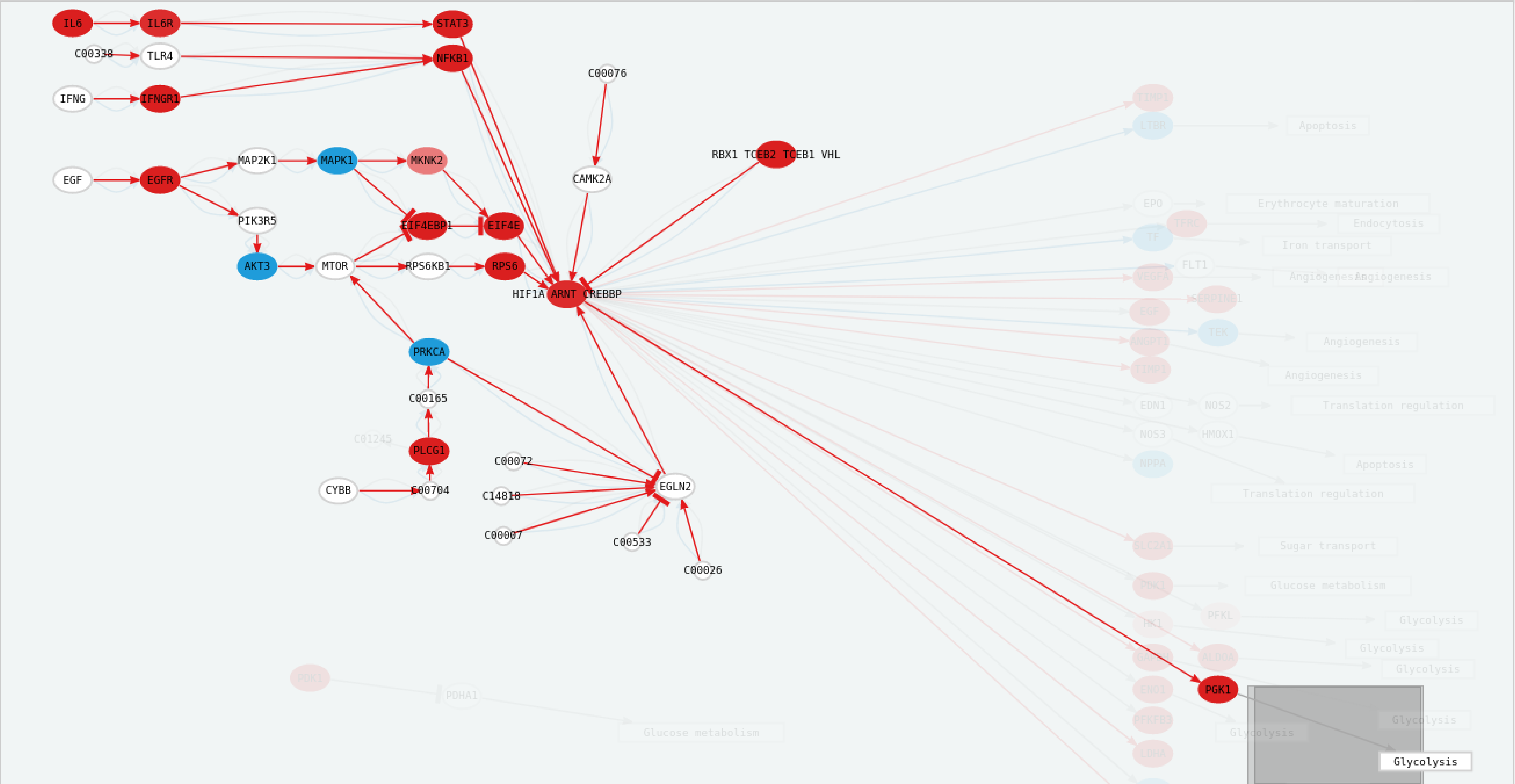

HIF-1 signaling pathway (hsa04066)

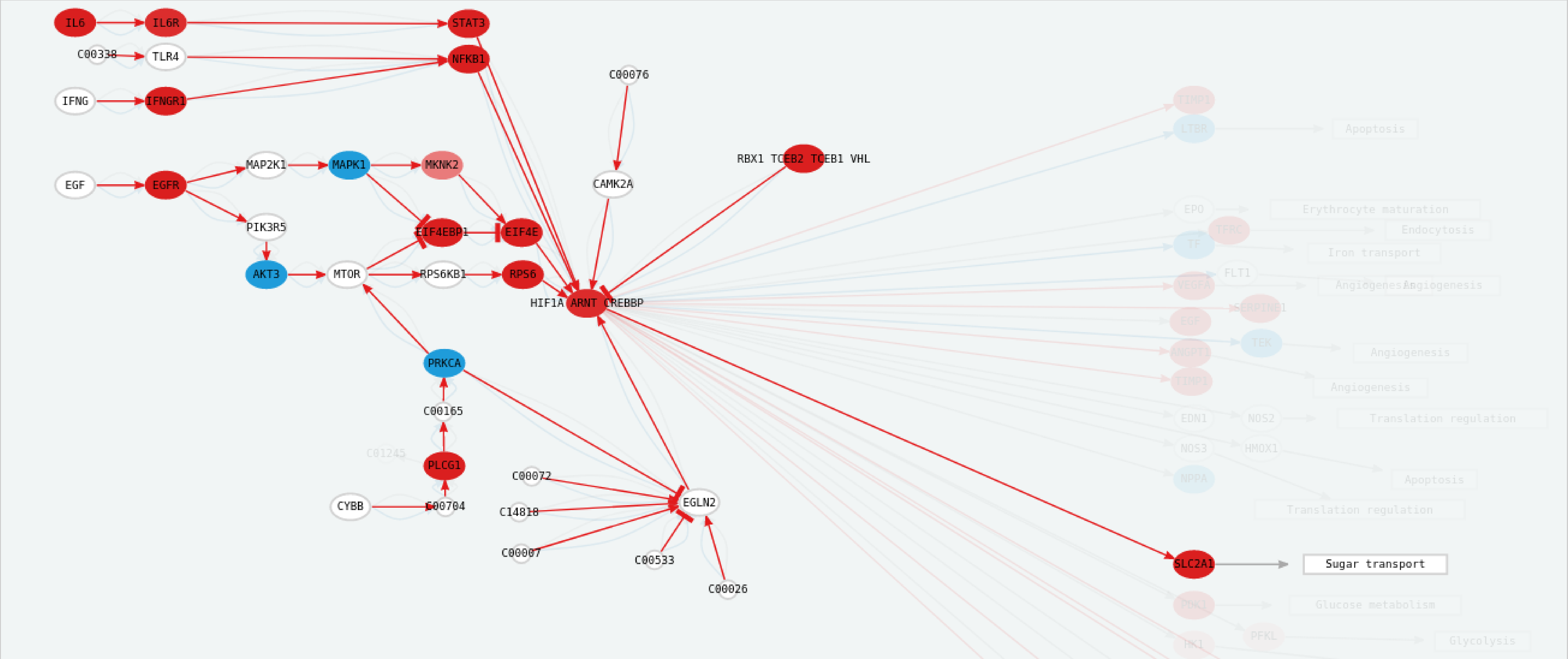

HIF-1 signaling pathway (hsa04066)

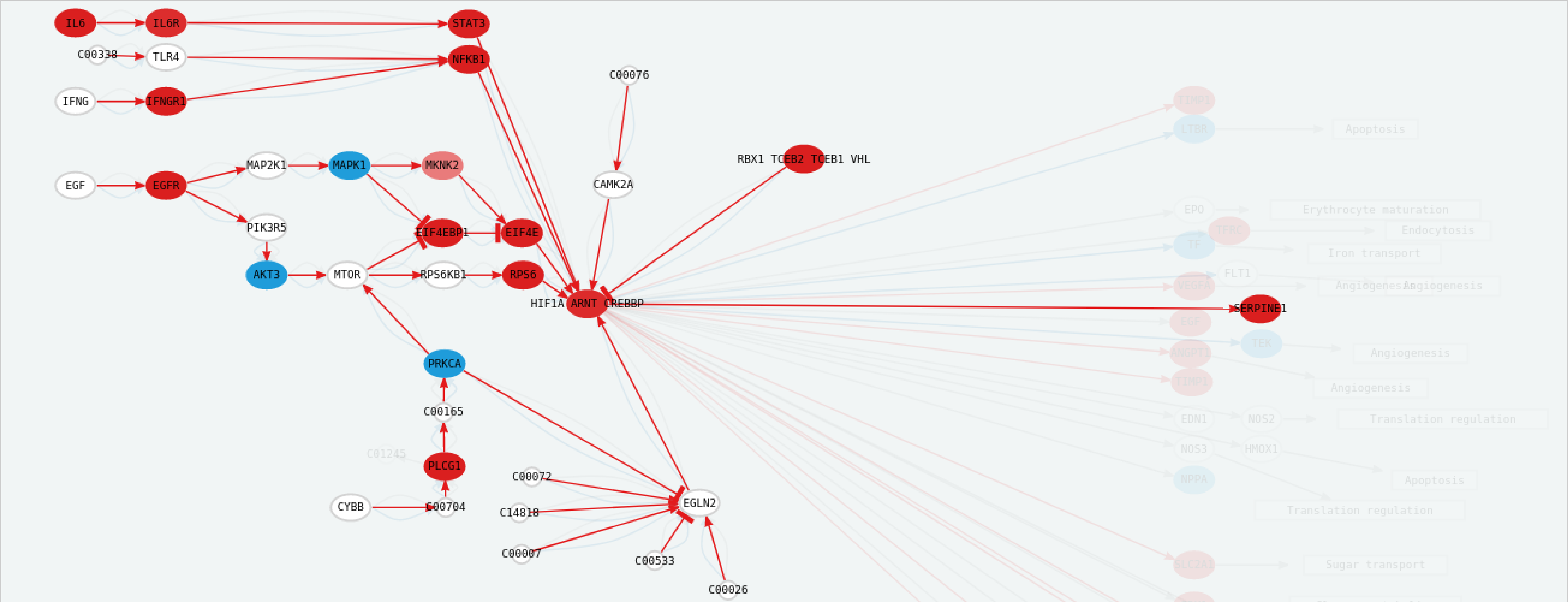

HIF-1 signaling pathway (hsa04066)

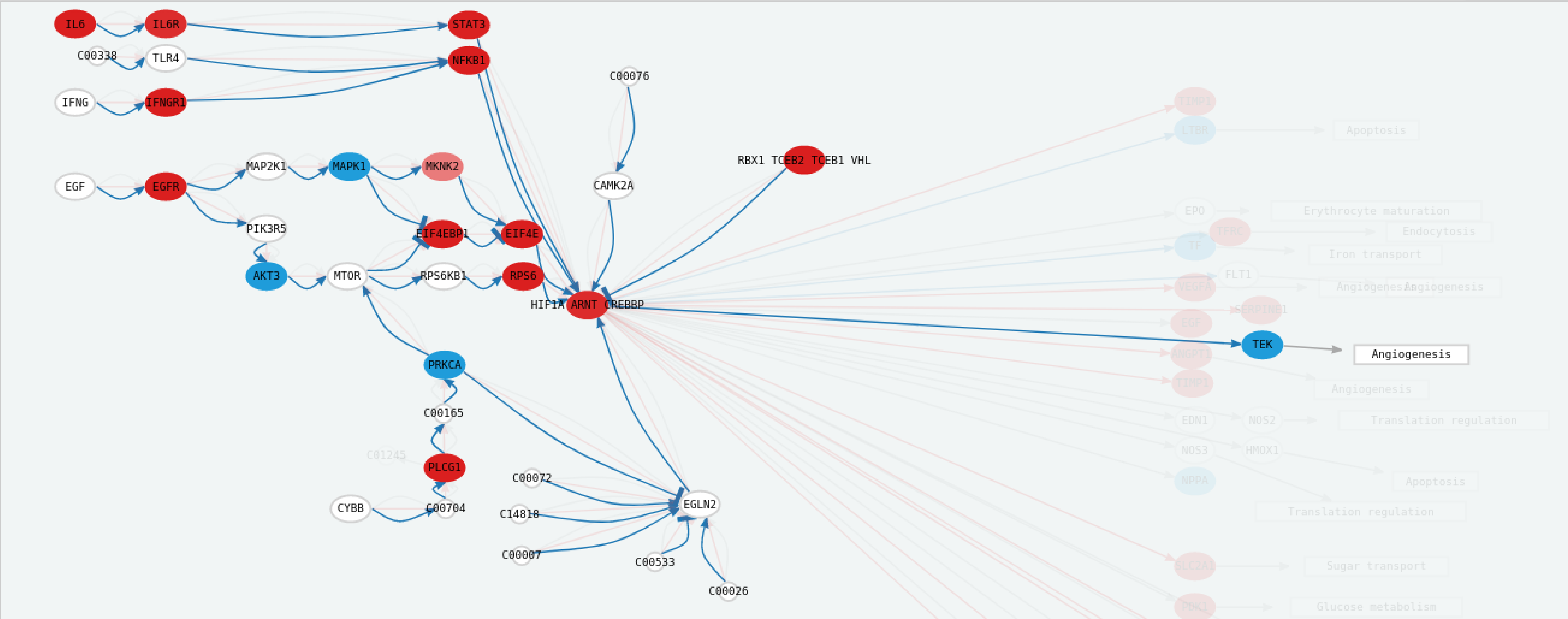

HIF-1 signaling pathway (hsa04066)

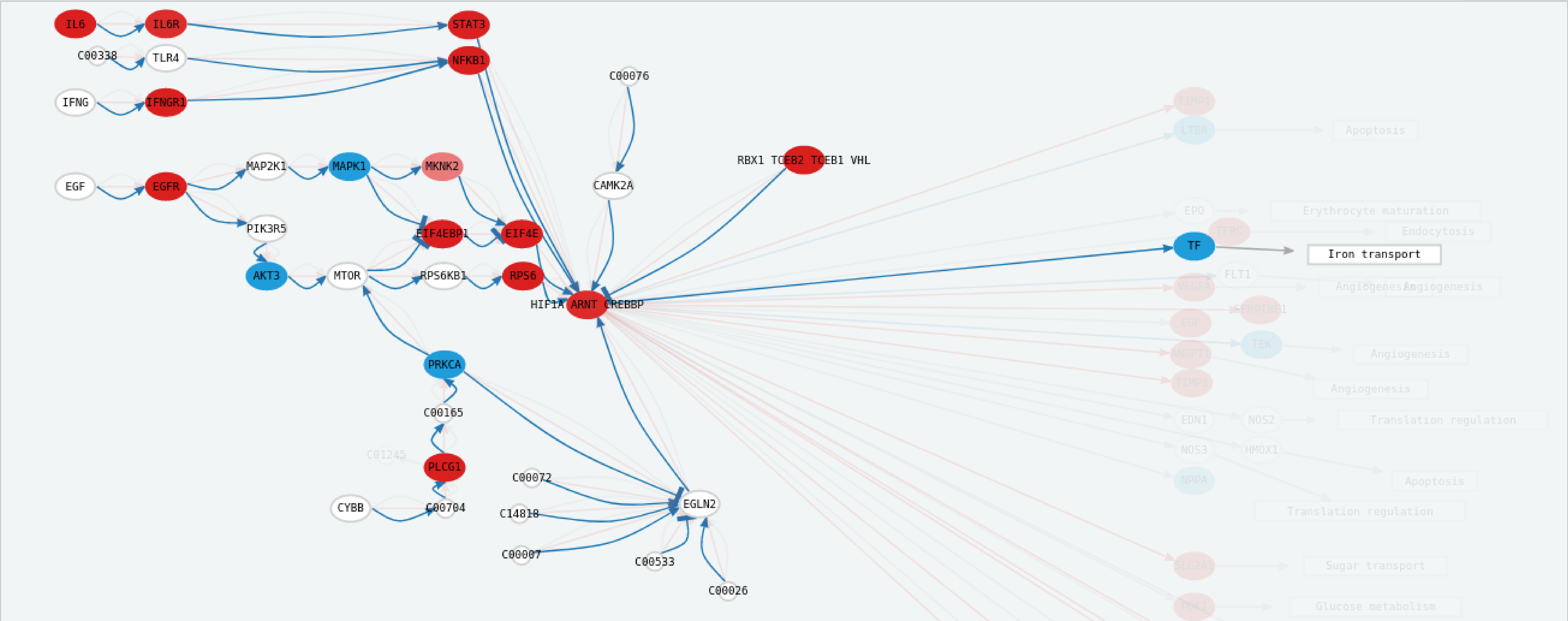

HIF-1 signaling pathway (hsa04066)

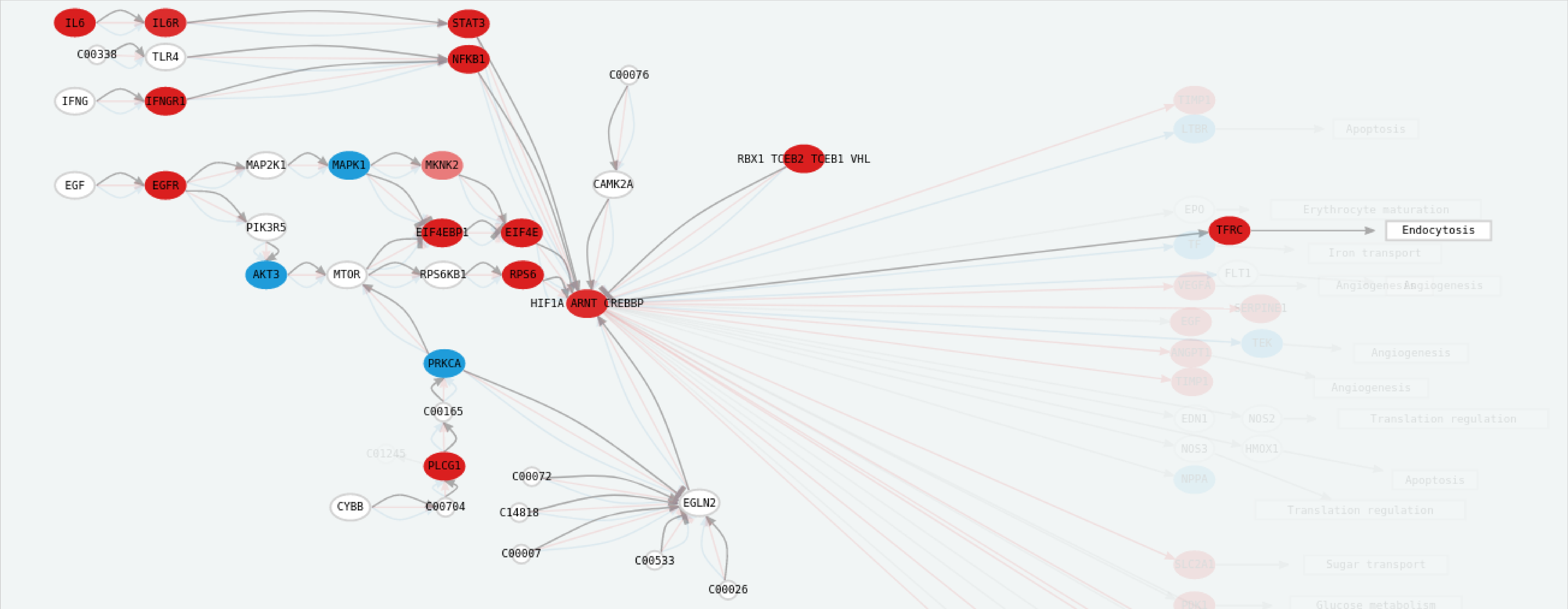

HIF-1 signaling pathway (hsa04066)

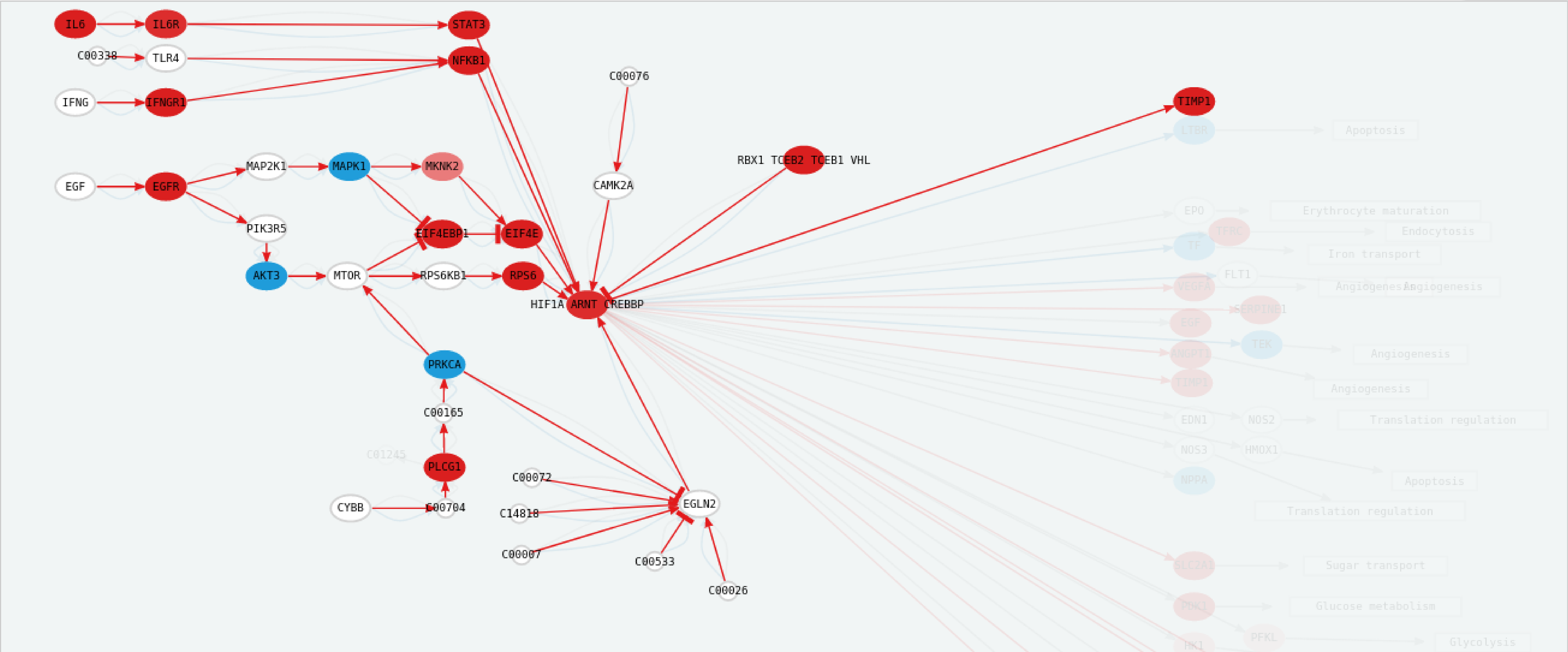

HIF-1 signaling pathway (hsa04066)

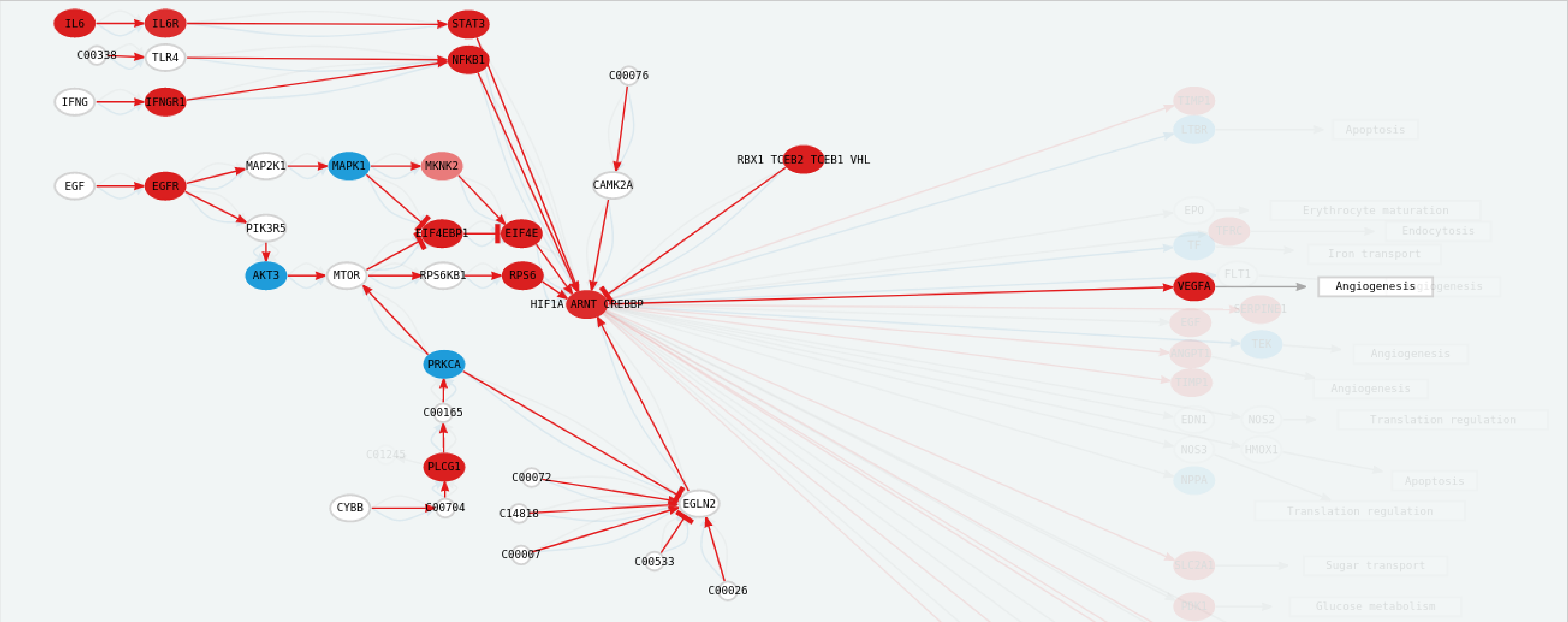

PI3K-Akt signaling pathway (hsa04151)

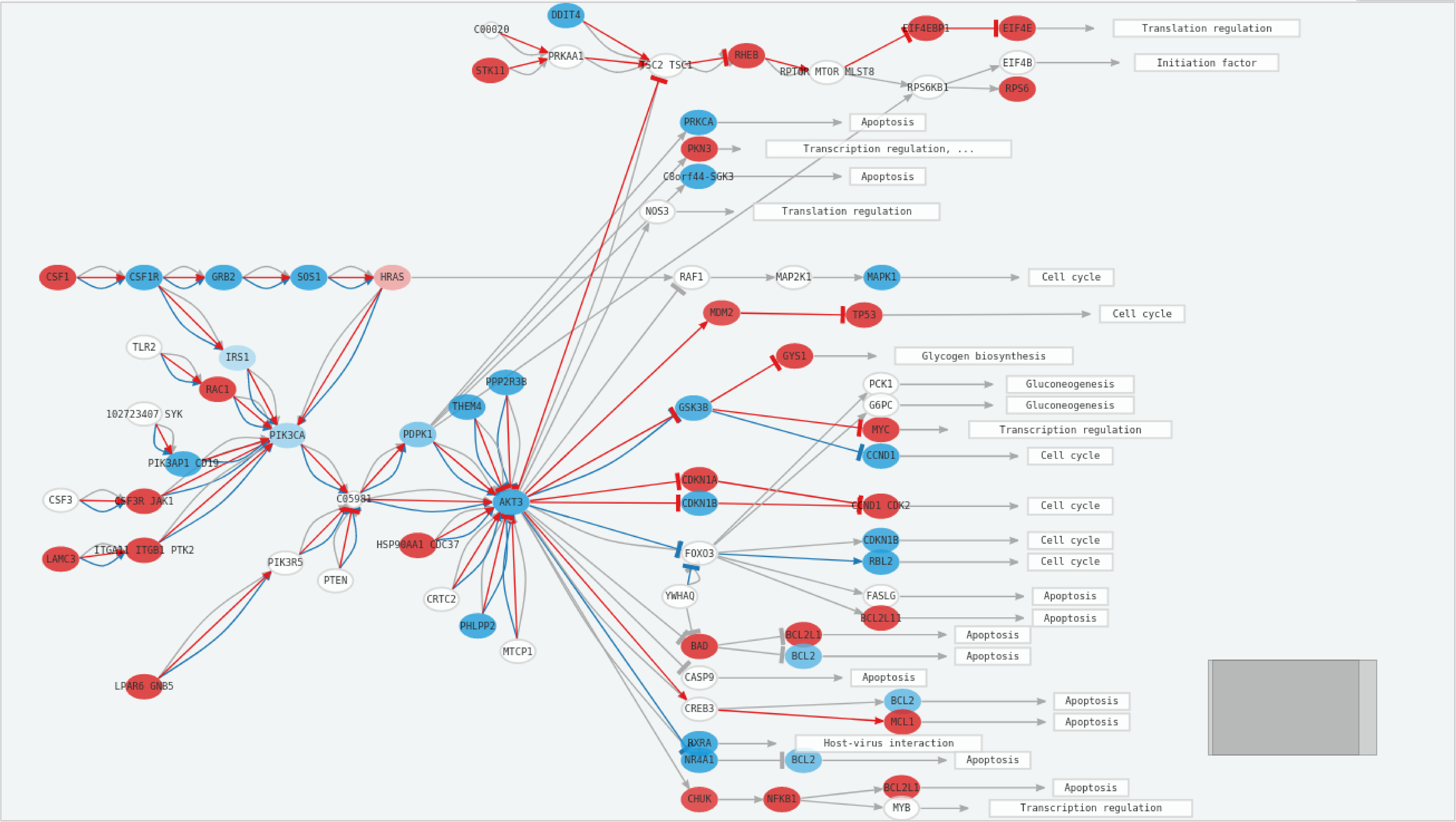

PI3K-Akt signaling pathway (hsa04151)

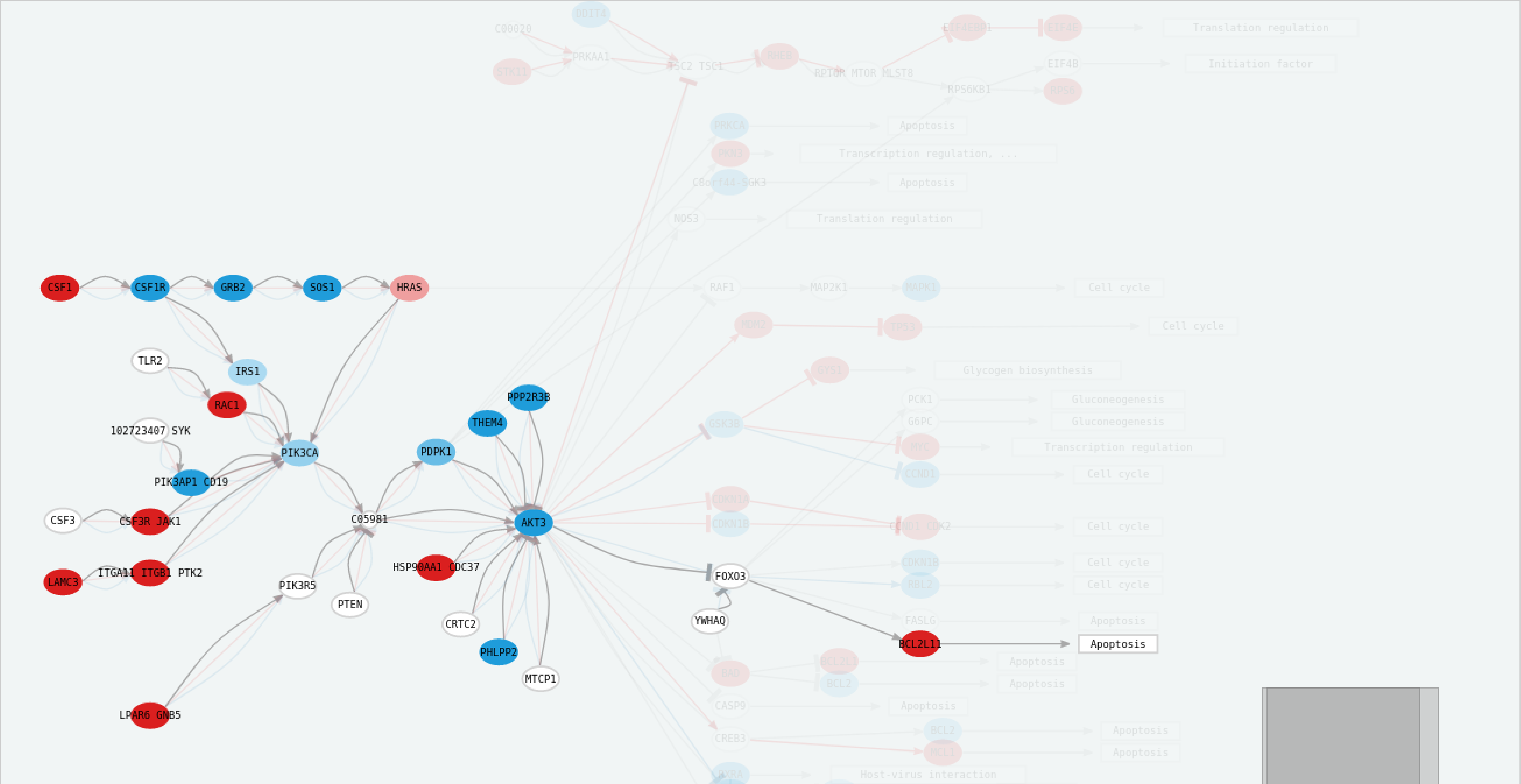

PI3K-Akt signaling pathway (hsa04151)

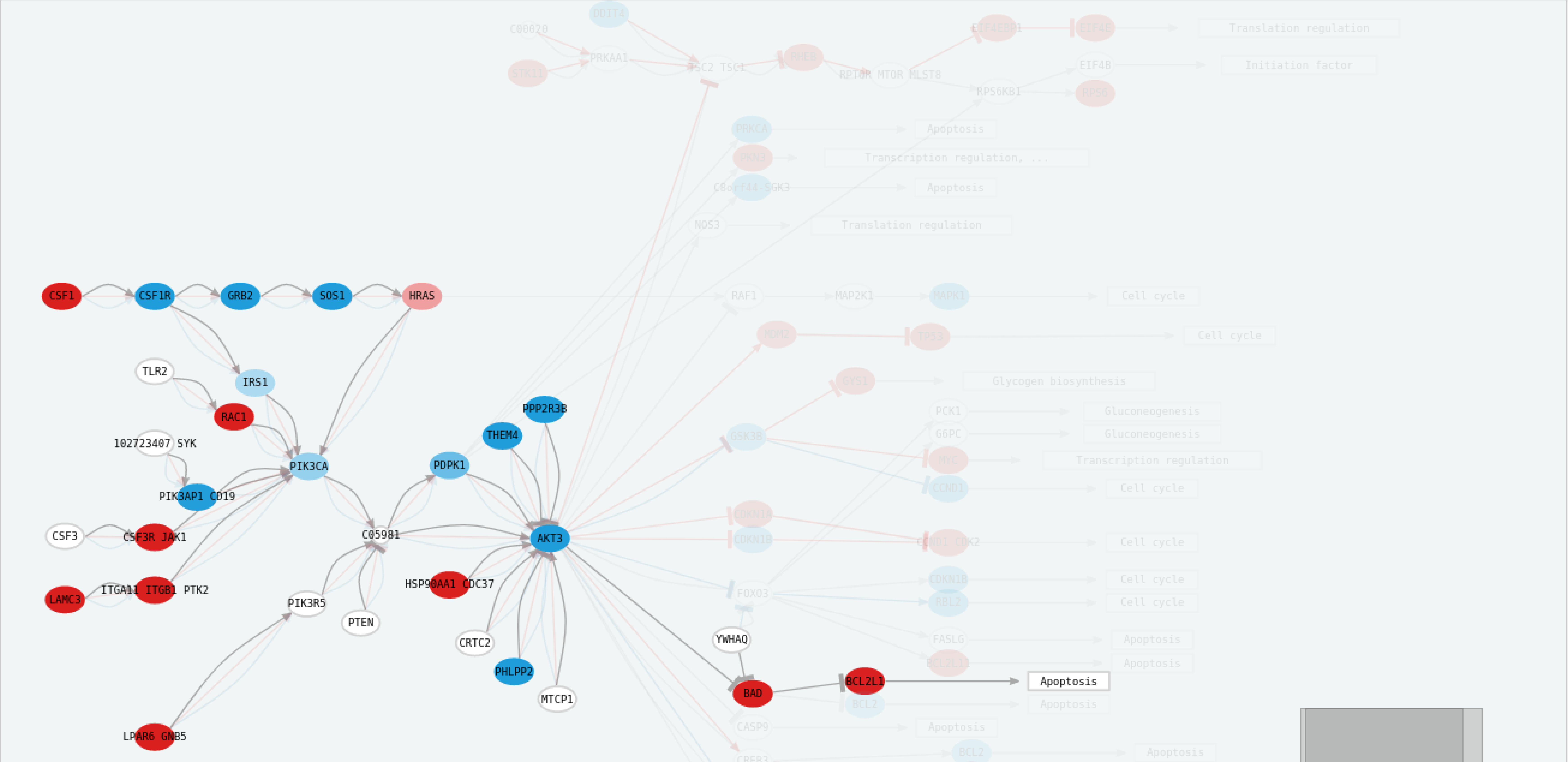

PI3K-Akt signaling pathway (hsa04151)

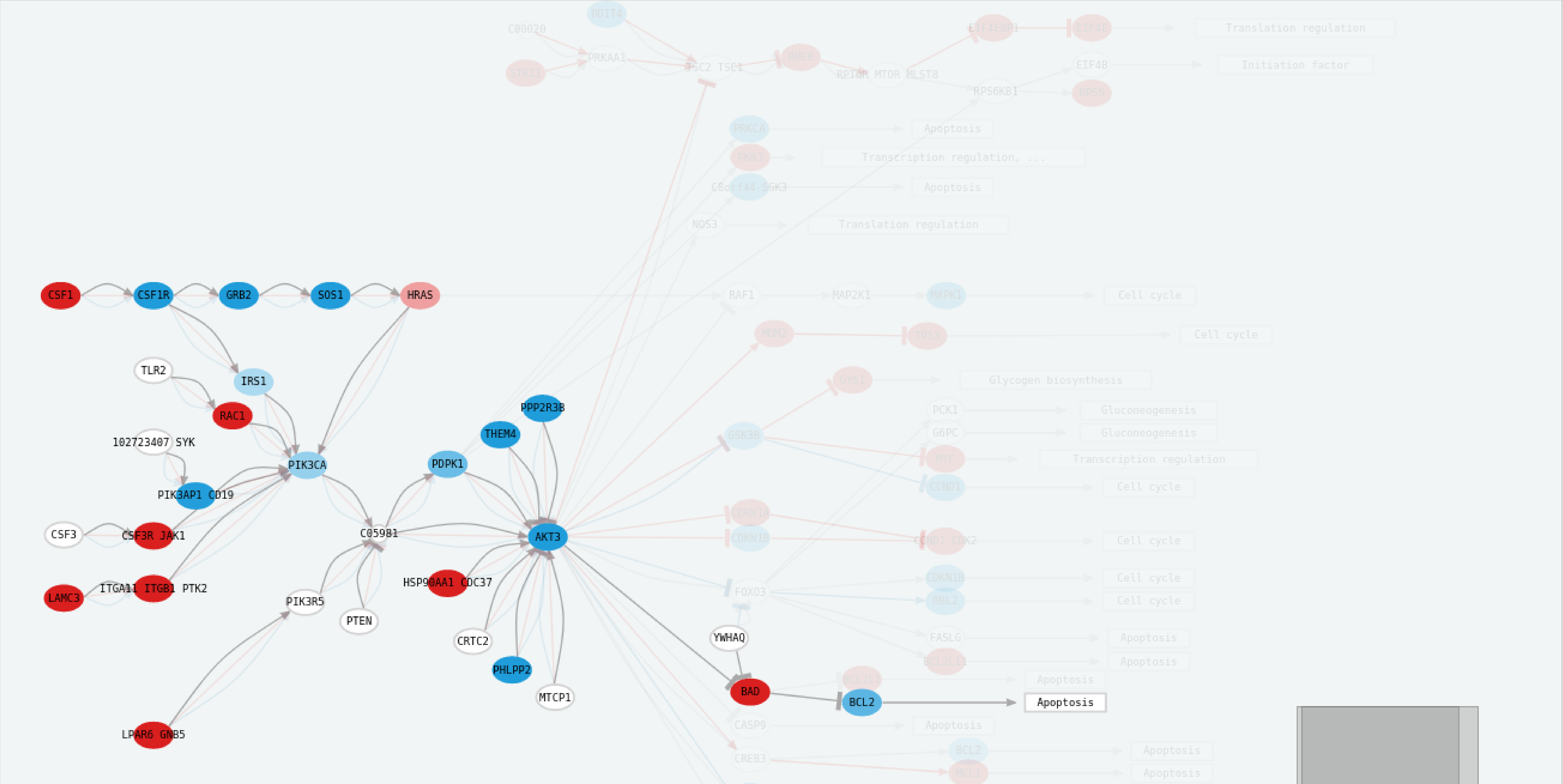

PI3K-Akt signaling pathway (hsa04151)

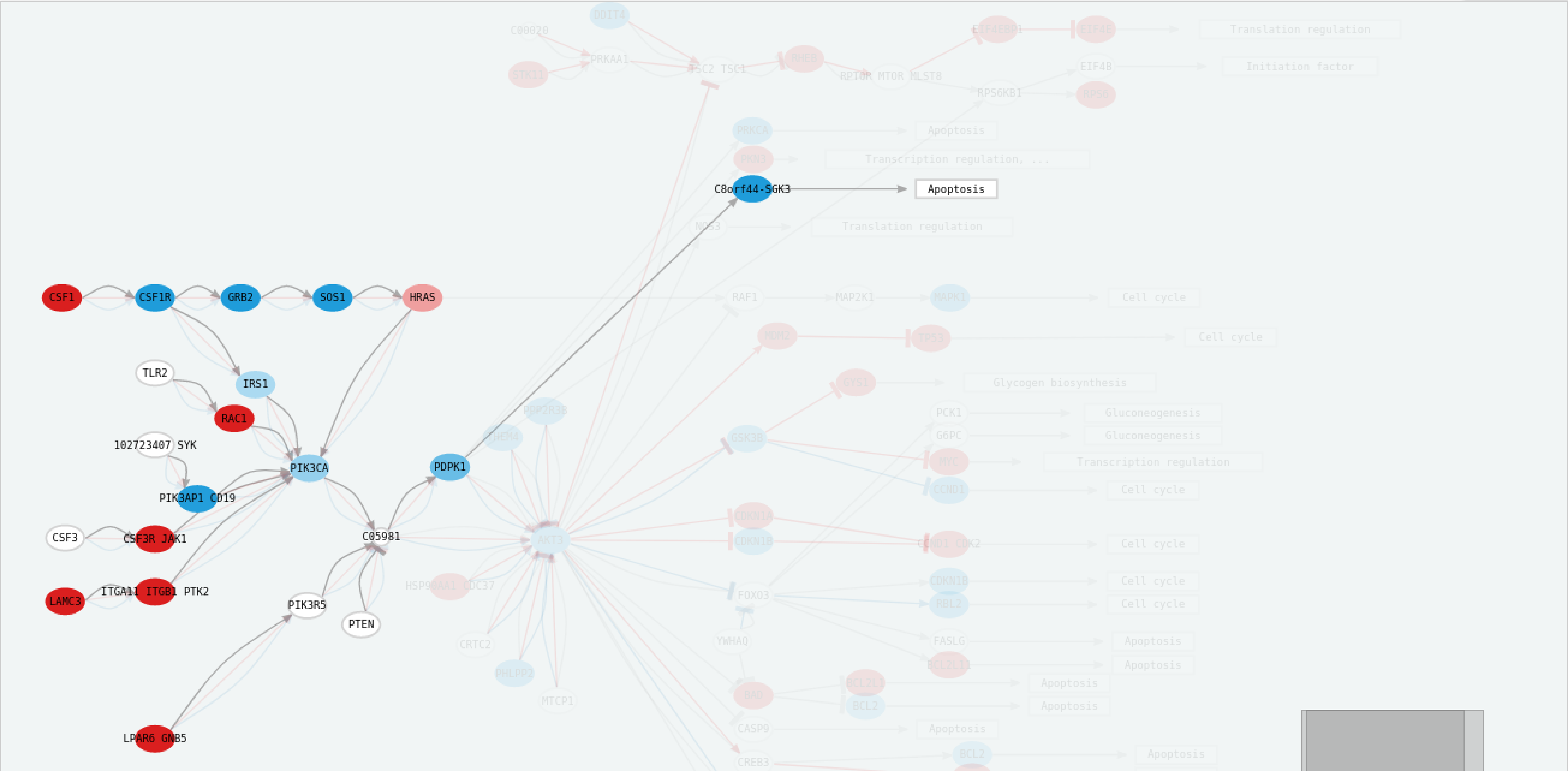

PI3K-Akt signaling pathway (hsa04151)

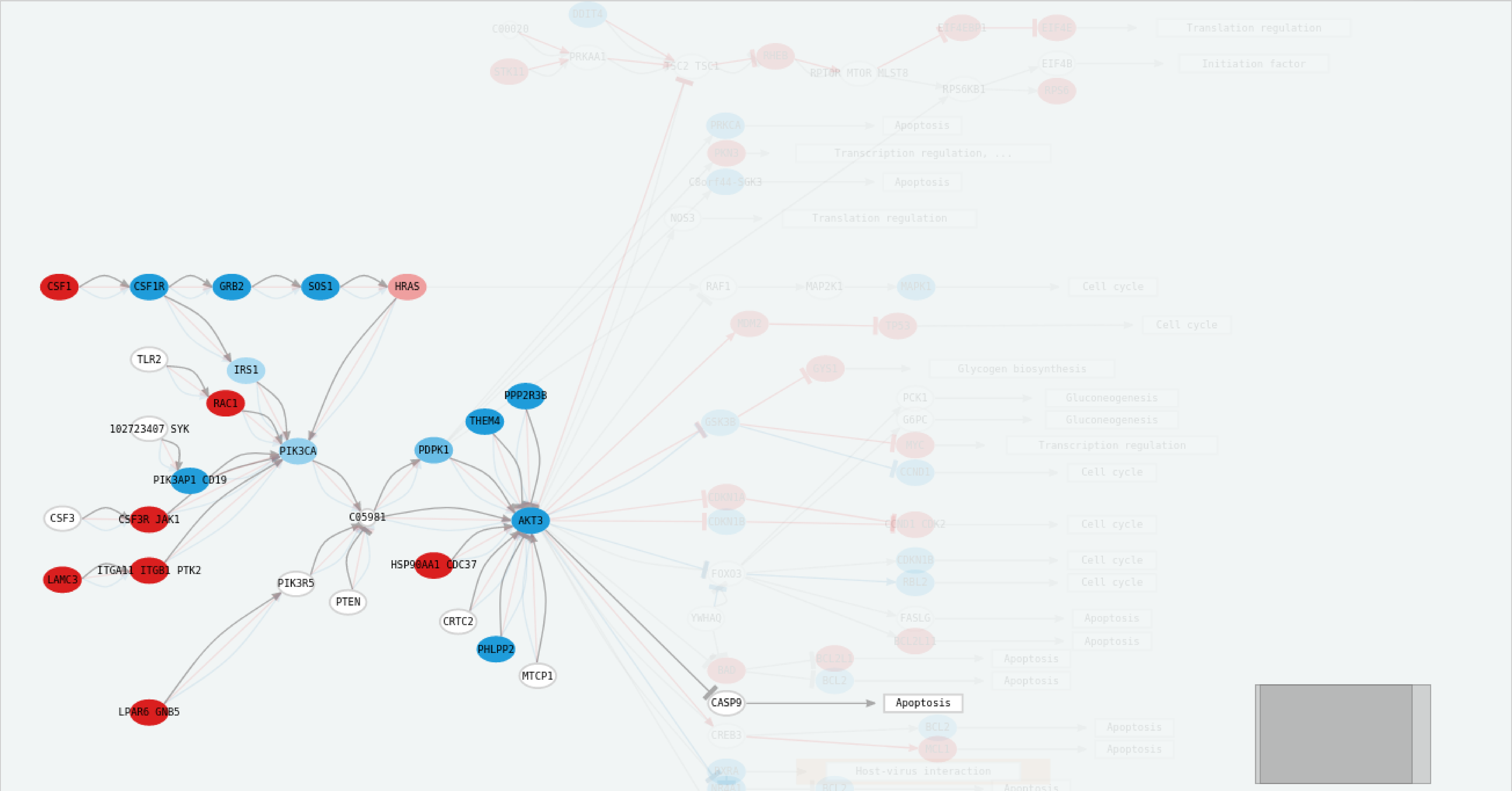

PI3K-Akt signaling pathway (hsa04151)

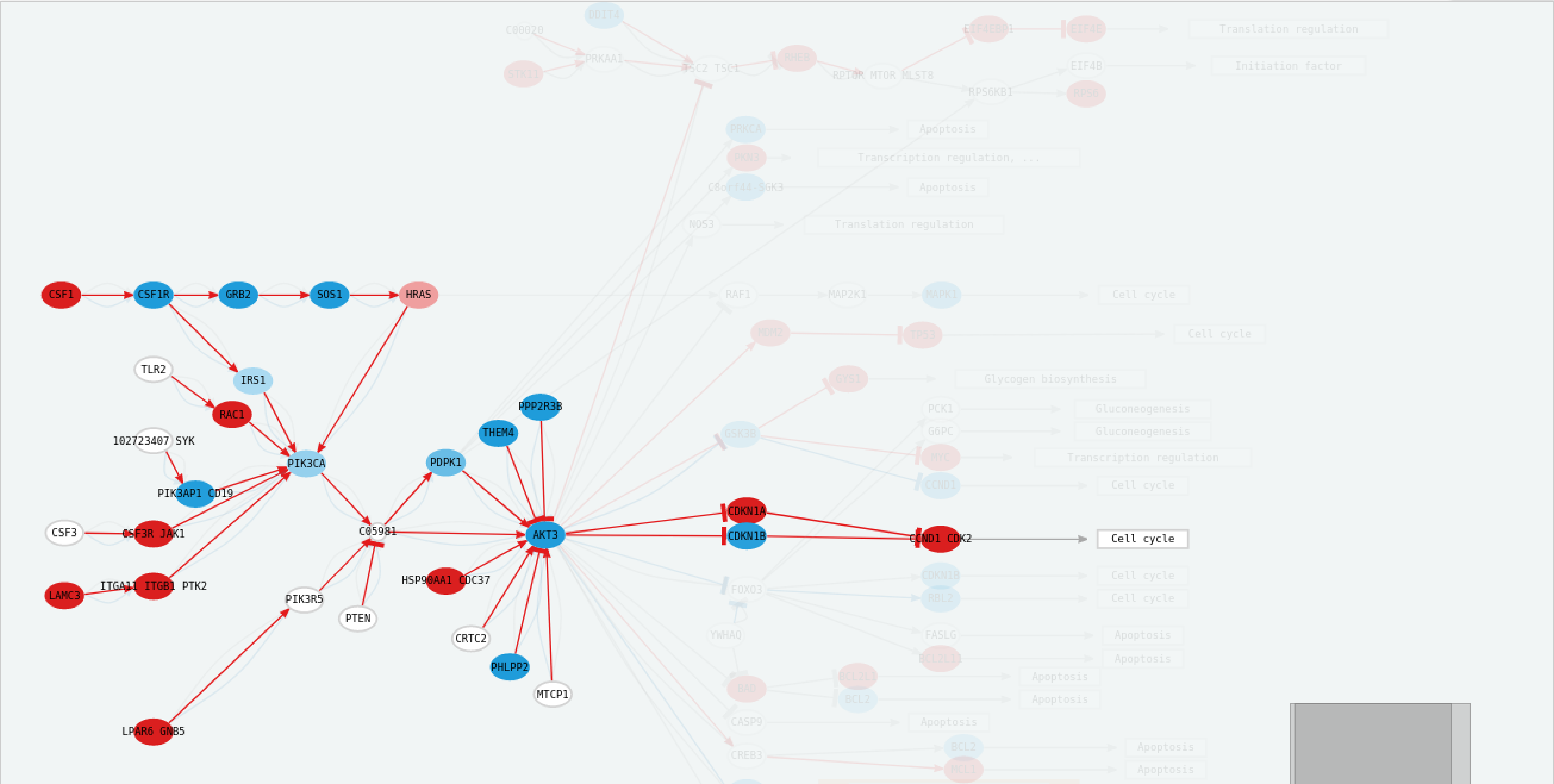

PI3K-Akt signaling pathway (hsa04151)

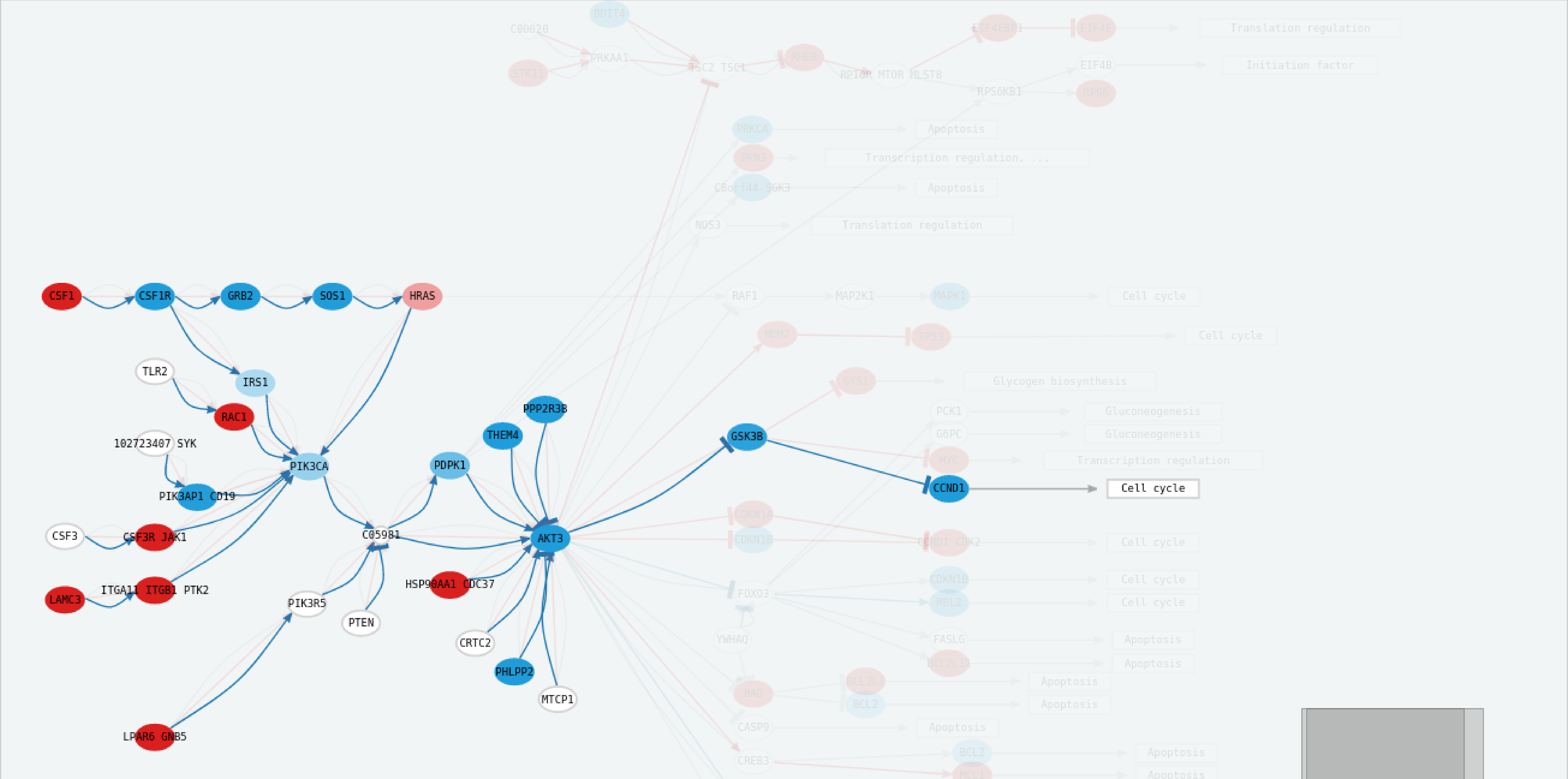

PI3K-Akt signaling pathway (hsa04151)

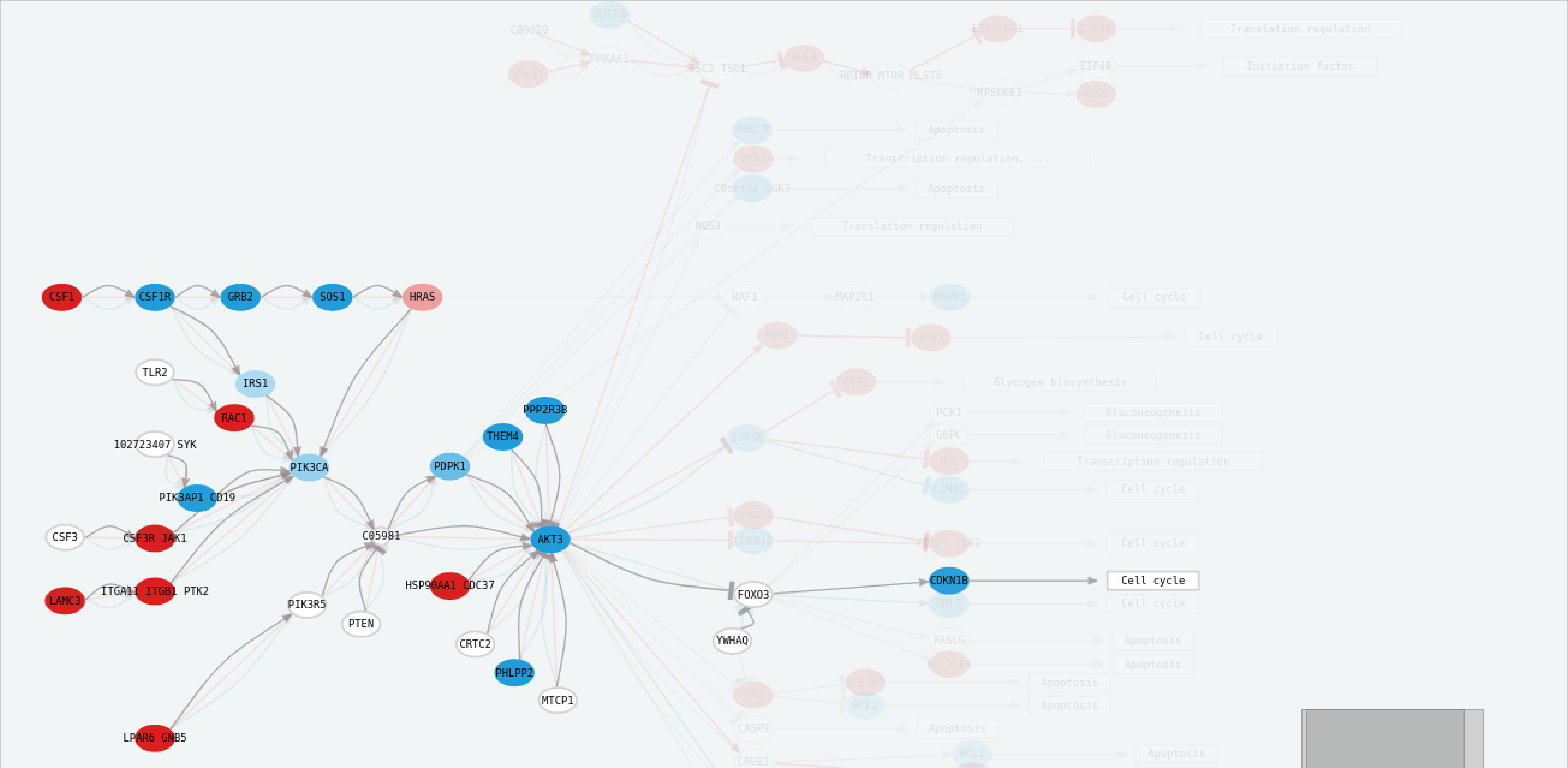

PI3K-Akt signaling pathway (hsa04151)

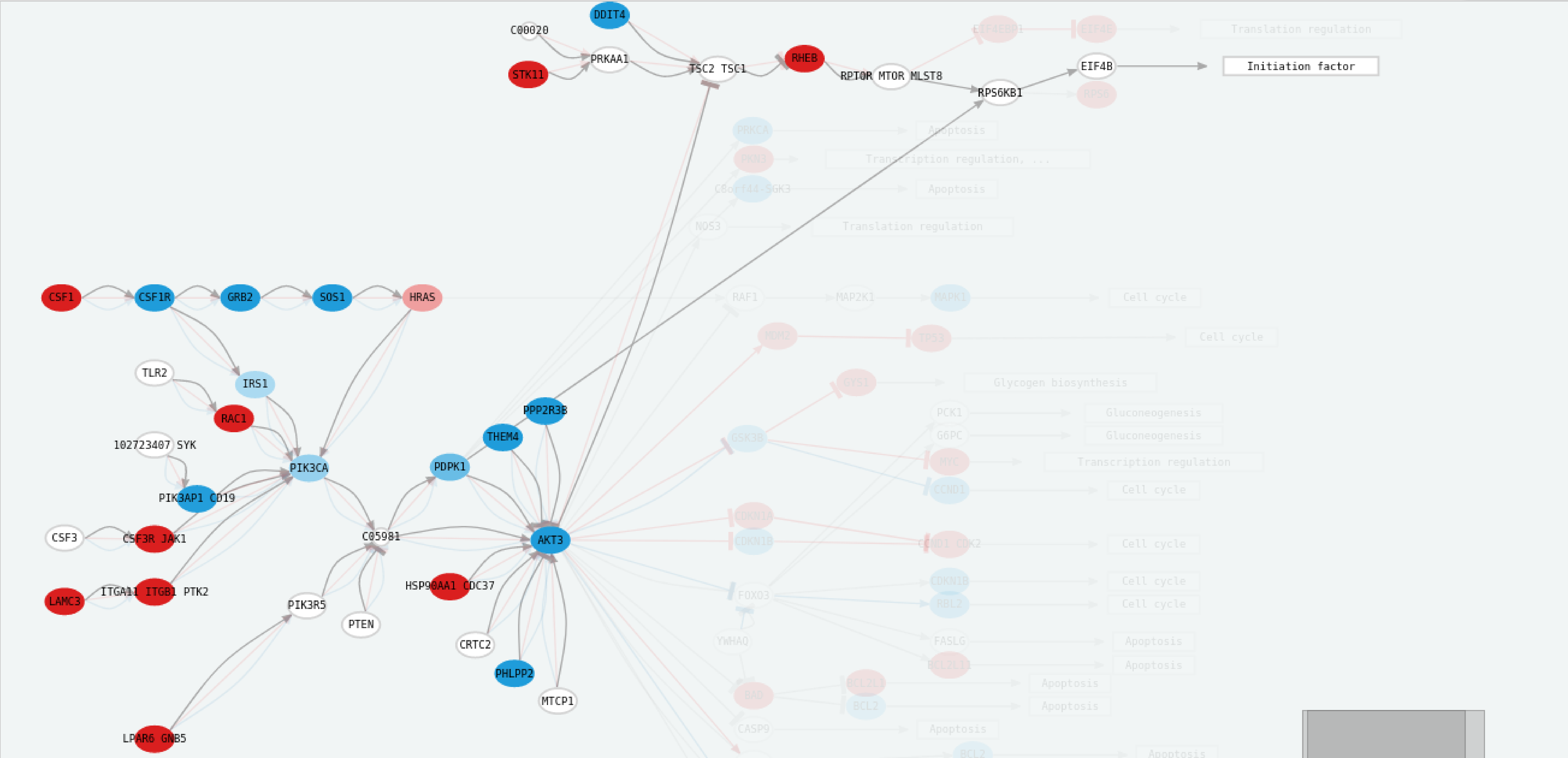

PI3K-Akt signaling pathway (hsa04151)

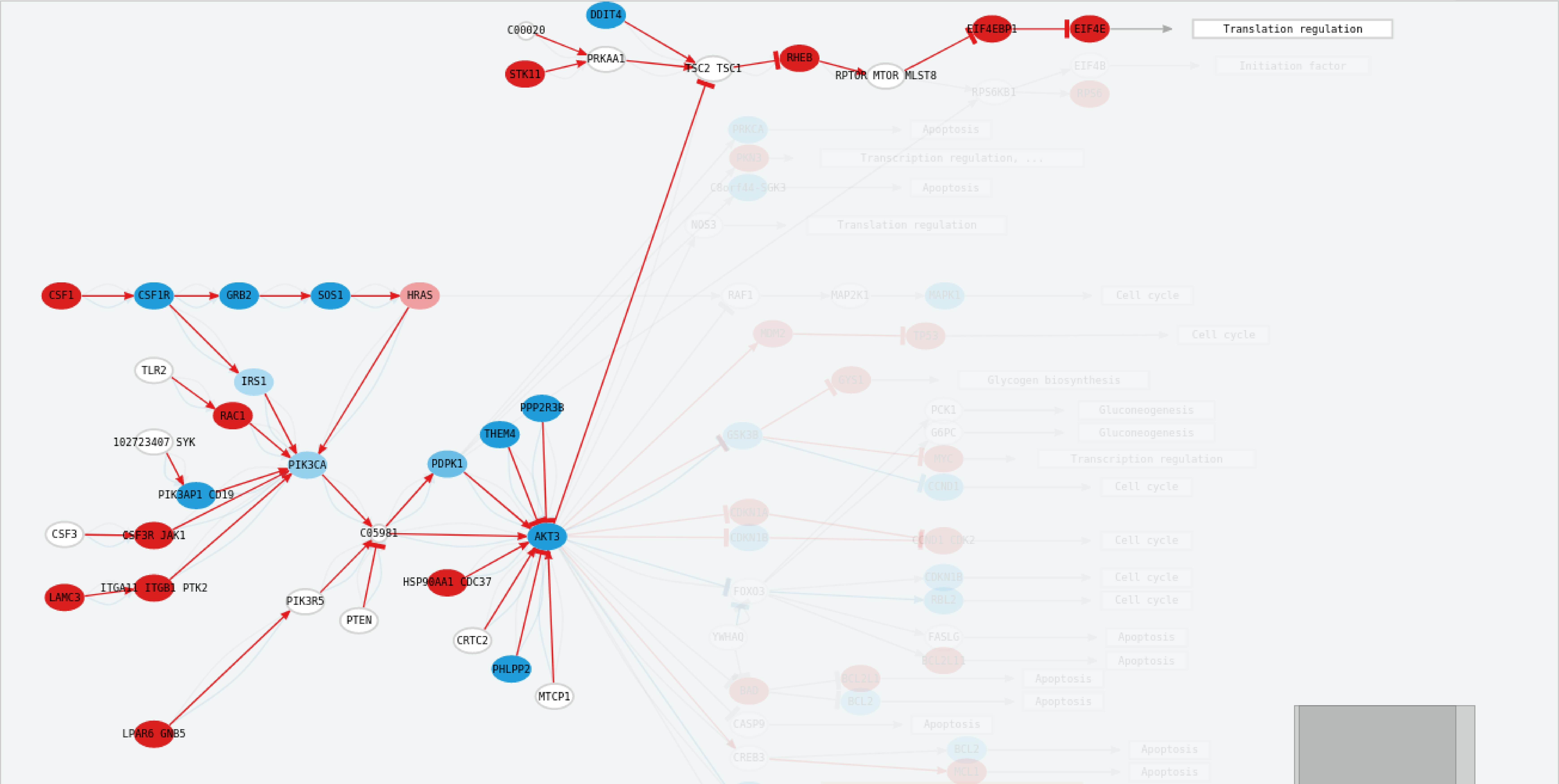

PI3K-Akt signaling pathway (hsa04151)

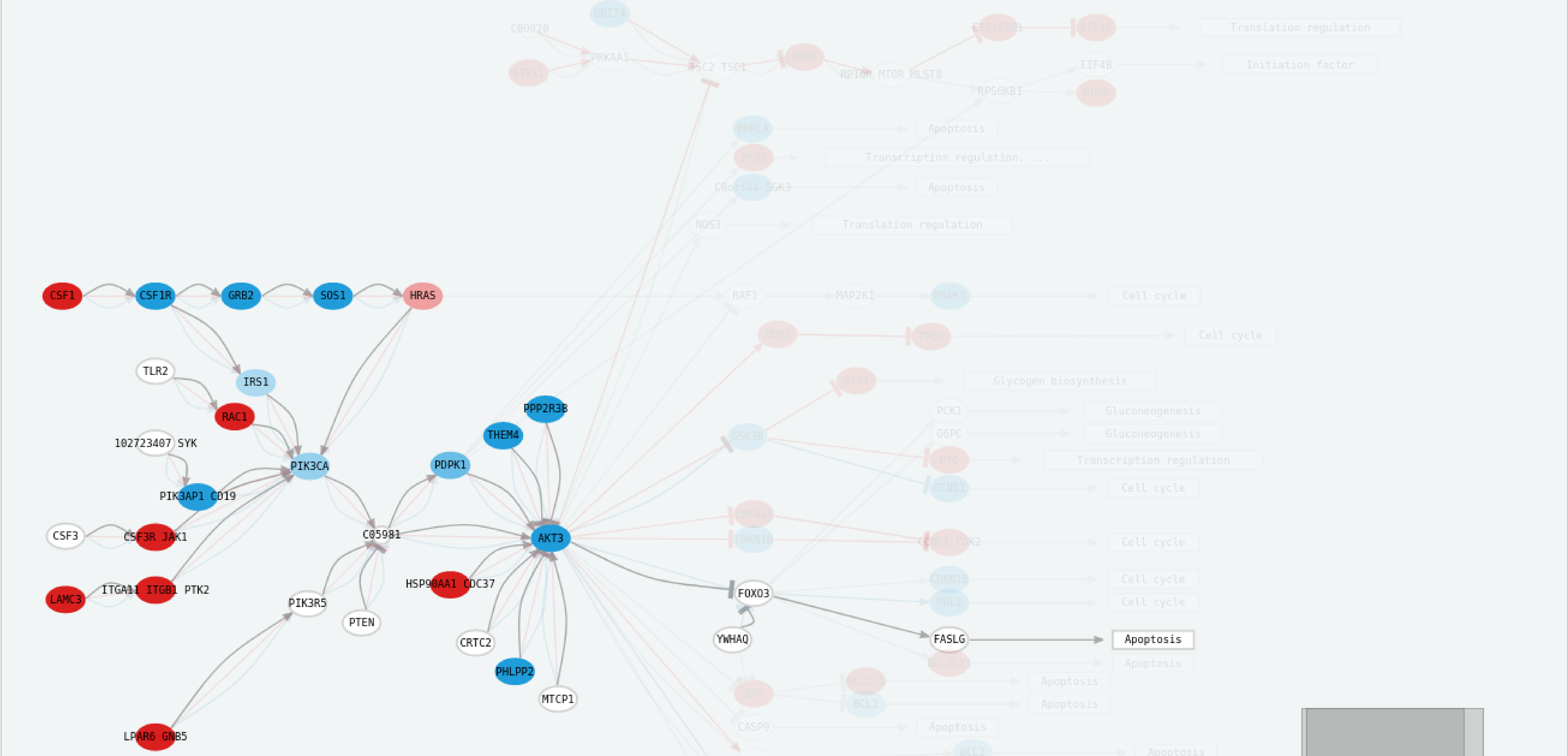

PI3K-Akt signaling pathway (hsa04151)

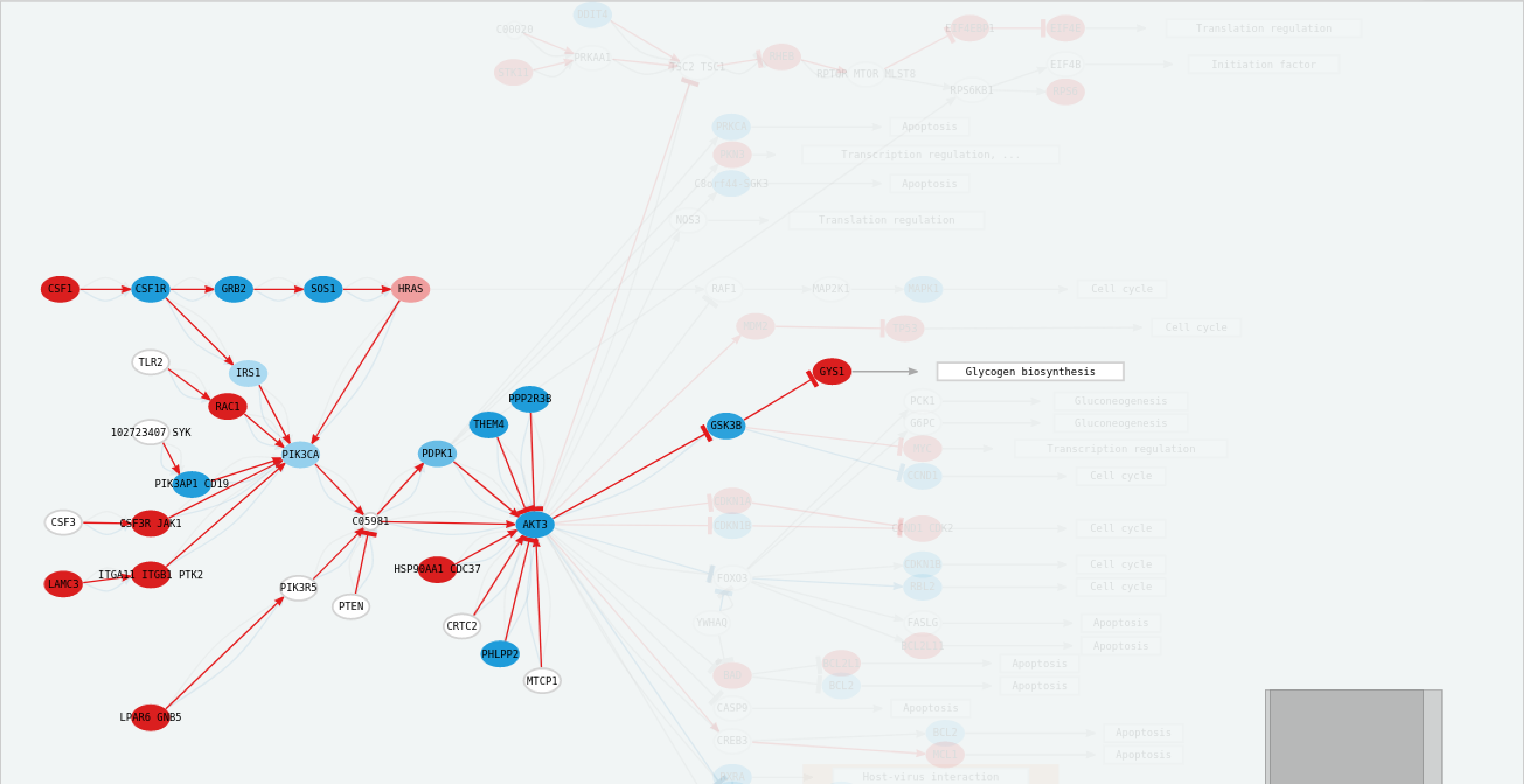

PI3K-Akt signaling pathway (hsa04151)

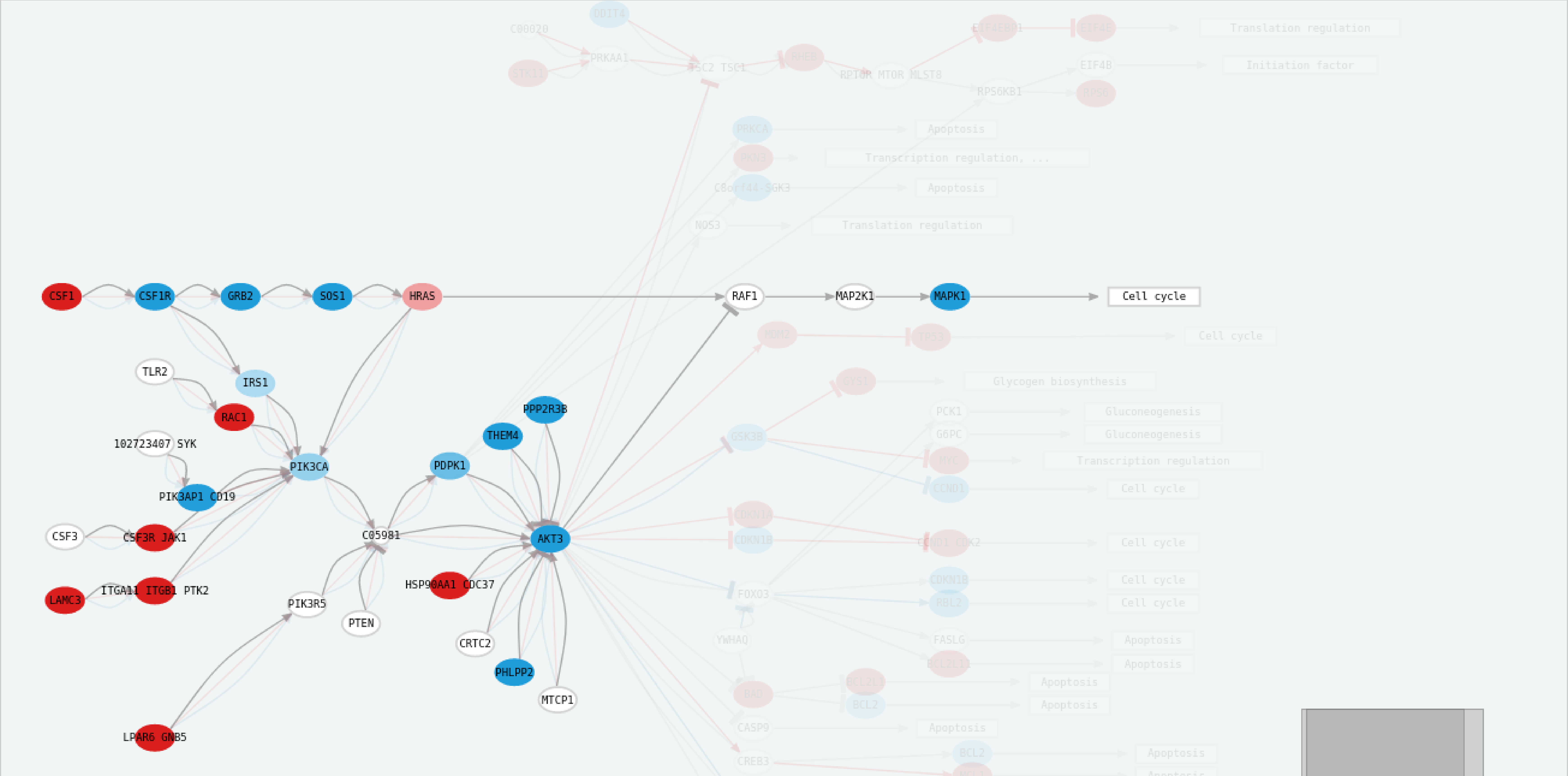

PI3K-Akt signaling pathway (hsa04151)

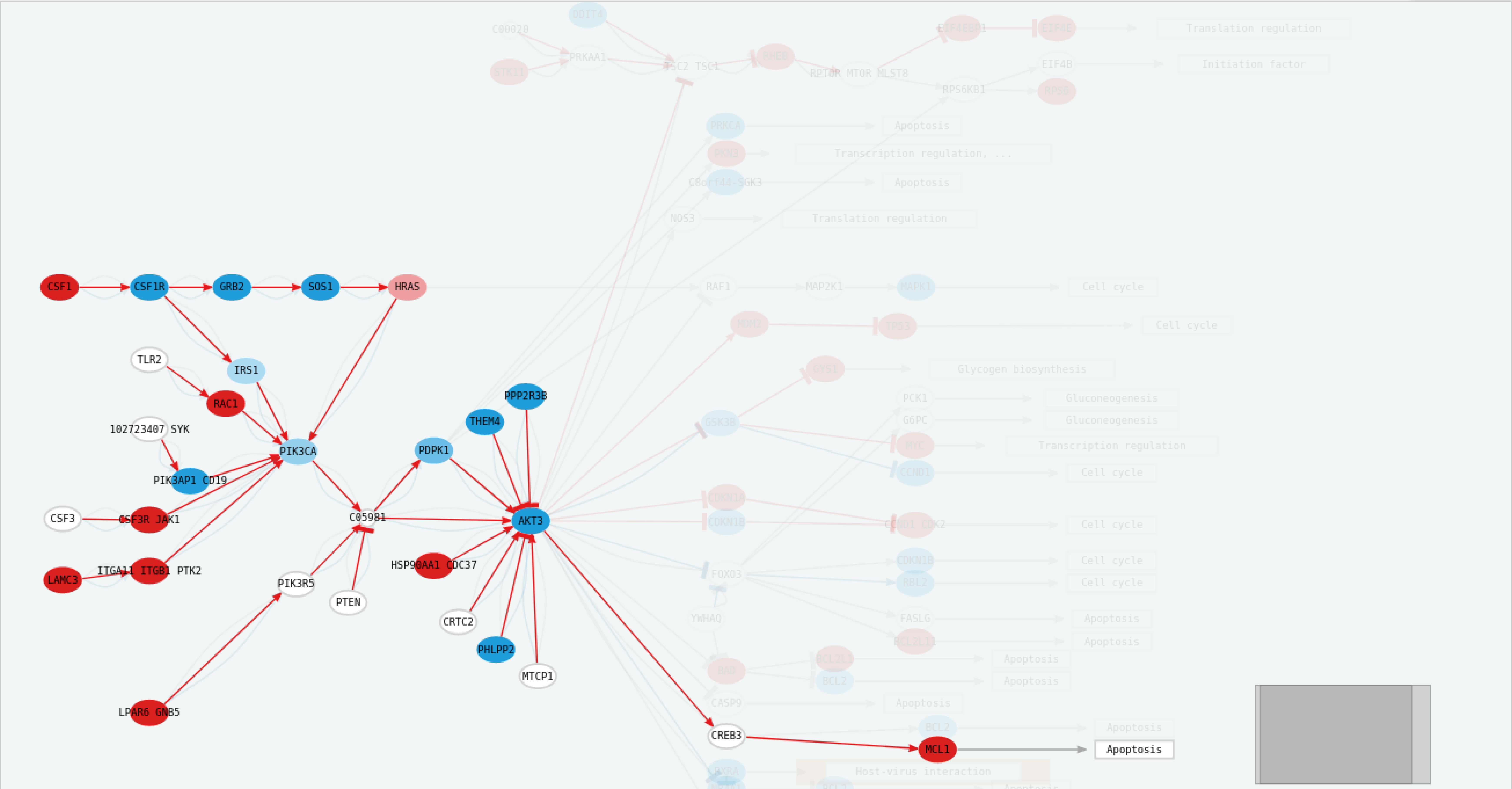

PI3K-Akt signaling pathway (hsa04151)

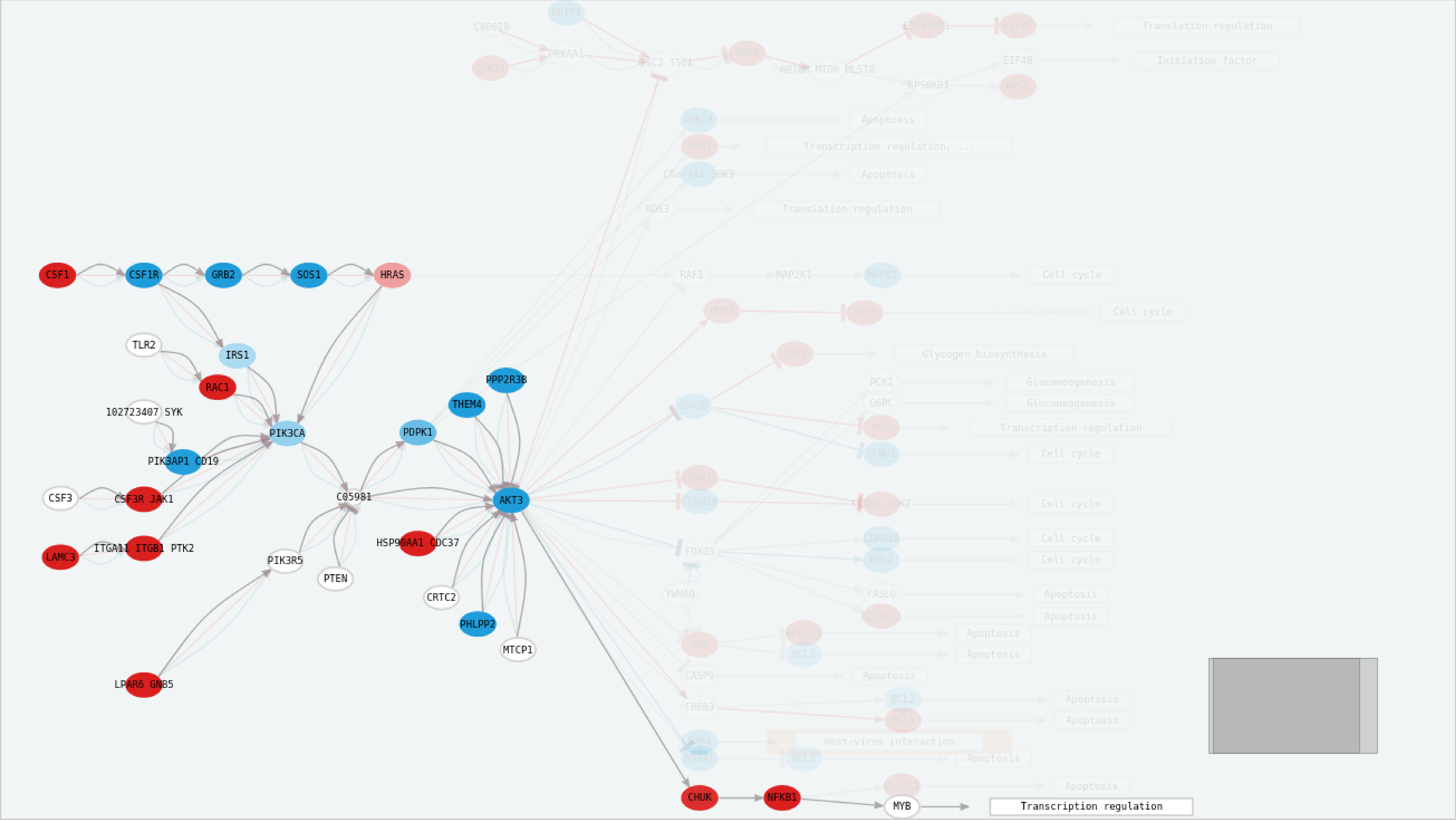

PI3K-Akt signaling pathway (hsa04151)

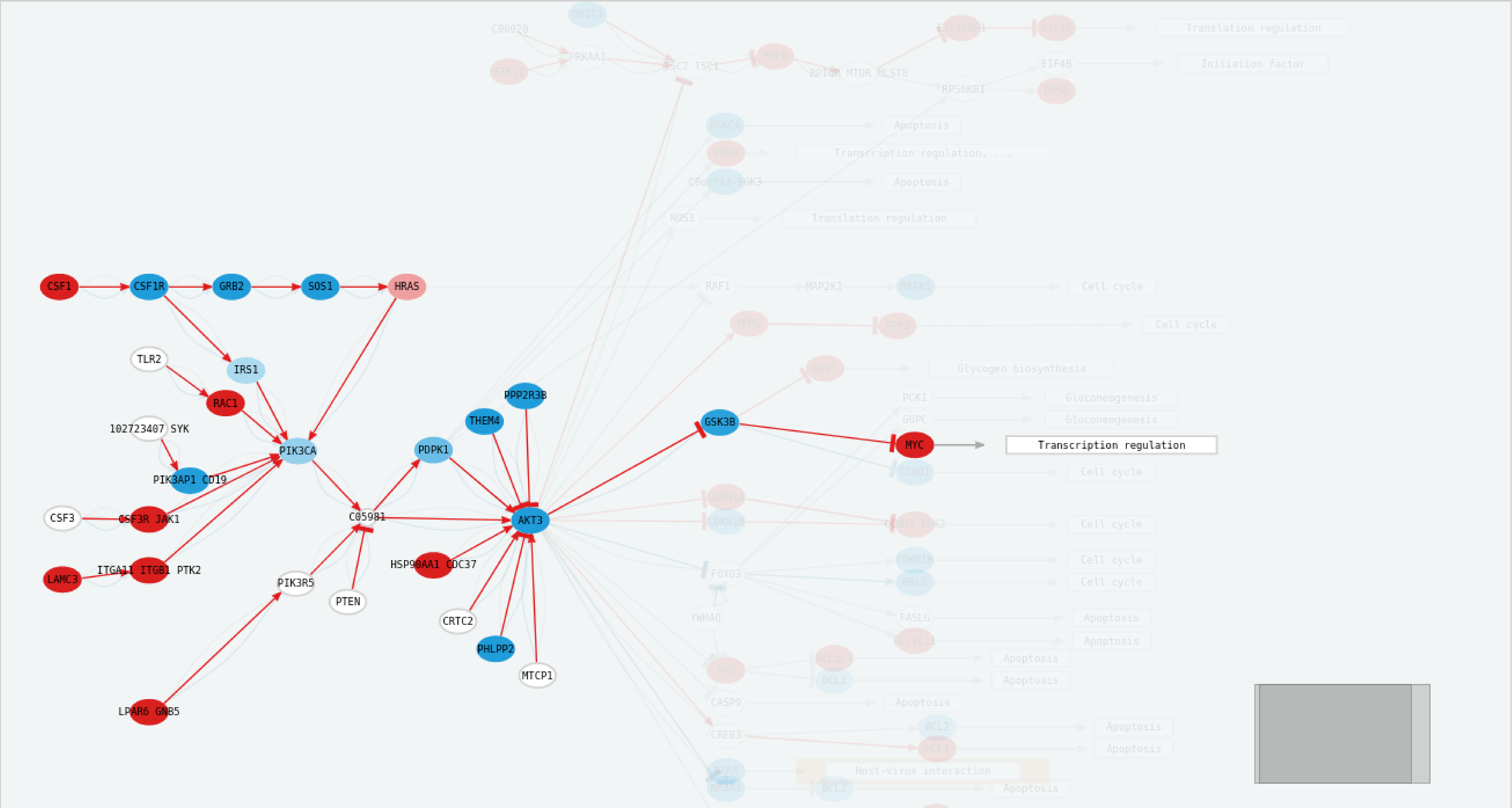

PI3K-Akt signaling pathway (hsa04151)

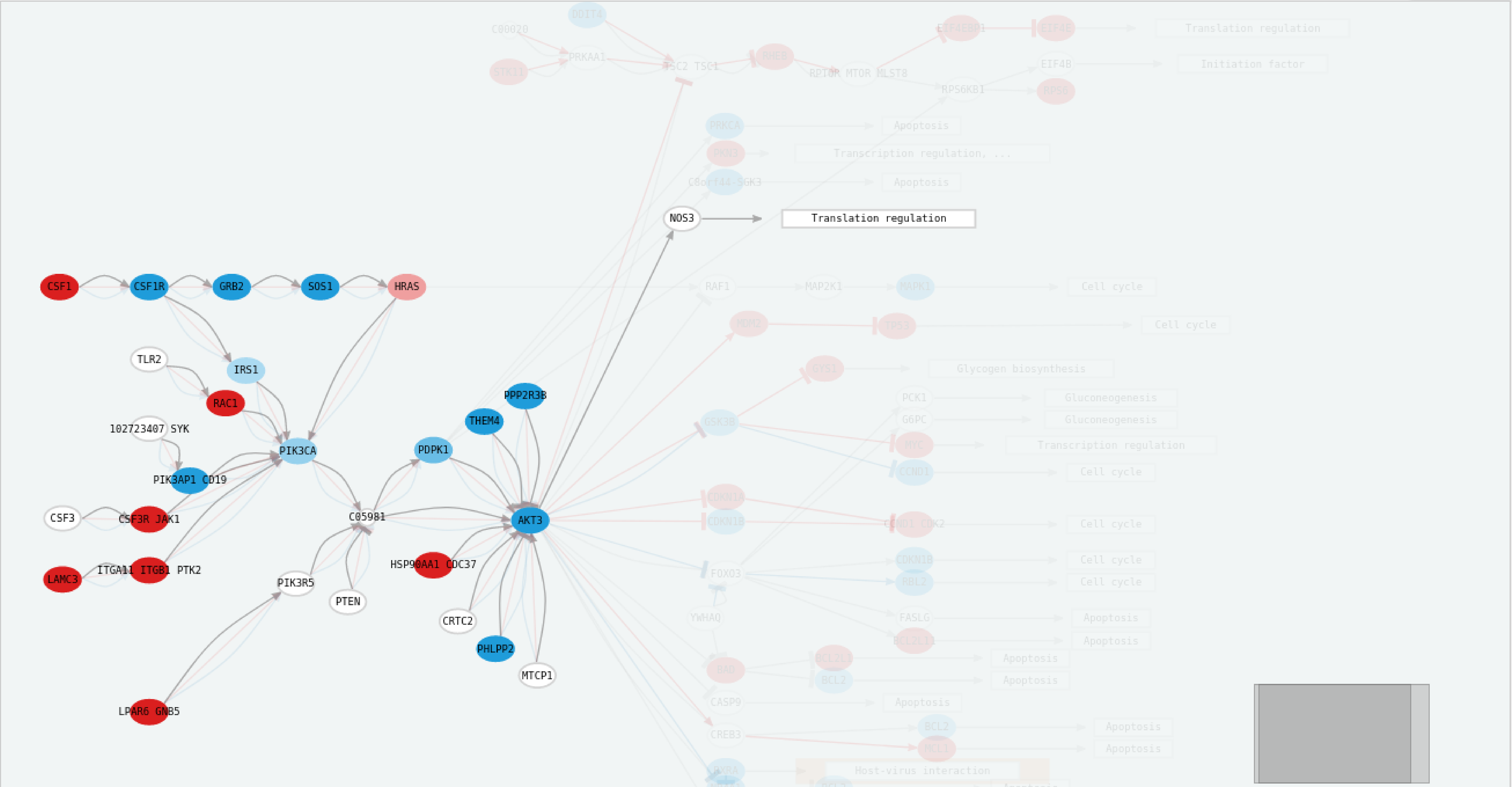

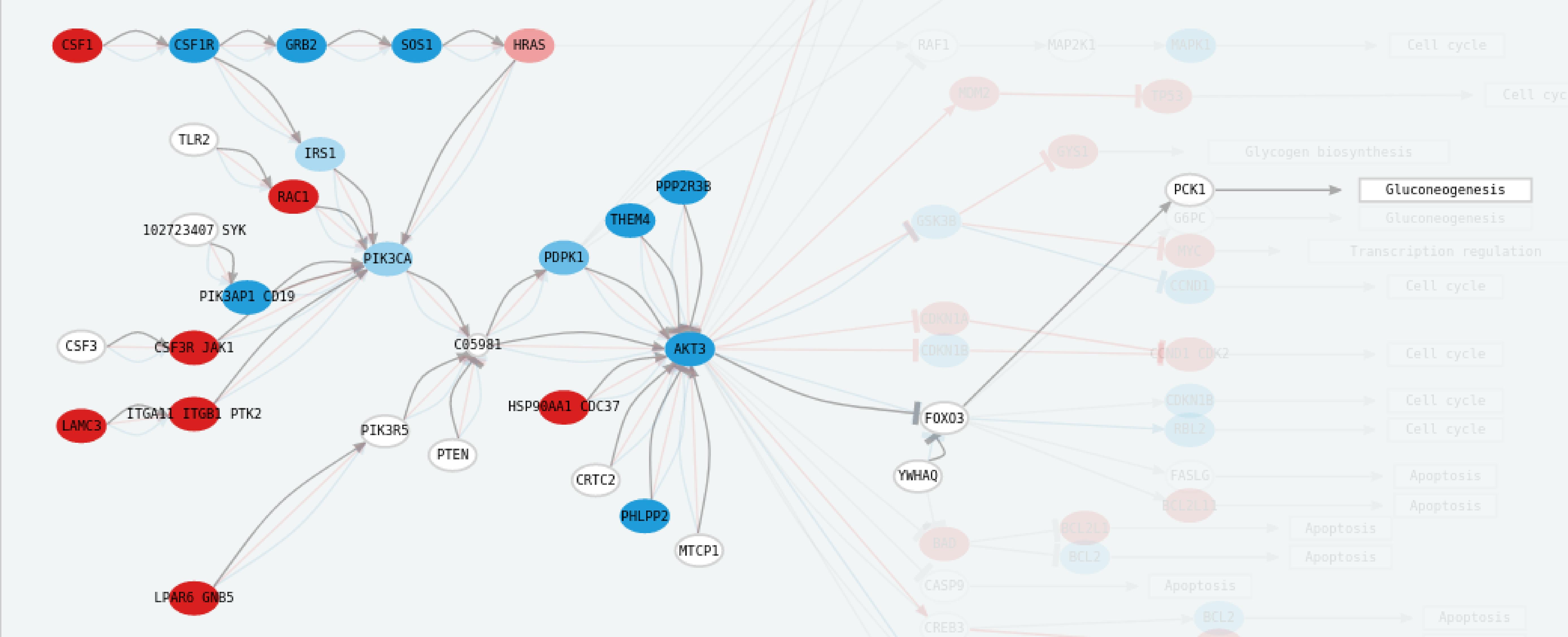

CSF1
CSF1R
GRB2
SOS1
HRAS
RAF1
MAP2K1
MAPK1
Cell cycle
MDM2
TP53
TLR2
IRS1
GYS1
Glycogen biosynthesis
RAC1
PCK1
Gluconeogenesis
G6PC
Gluconeogenesis
102723407 SYK
PPP2R3B
THEM4
GSK3B
MYC
Transcription regulation
PIK3CA
PDPK1
CCND1
Cell cycle
PIK3AP1 CD19
CDKN1A
CSF3
CSF3R JAK1
C05981
AKT3
CDKN1B
CCND1 CDK2
Cell cycle
HSP90AA1 CDC37
CDKN1B
Cell cycle
LAMC3
ITGA11 ITGB1 PTK2
FOXO3
PIK3R5
RBL2
Cell cycle
PTEN
CRTC2
YWHAQ
FASLG
Apoptosis
BCL2L11
Apoptosis
PHLPP2
MTCP1
BCL2L1
Apoptosis
BAD
BCL2
Apoptosis
CASP9
Apoptosis
LPAR6 GNB5
BCL2
Apoptosis
CREB3

PI3K-Akt signaling pathway (hsa04151)

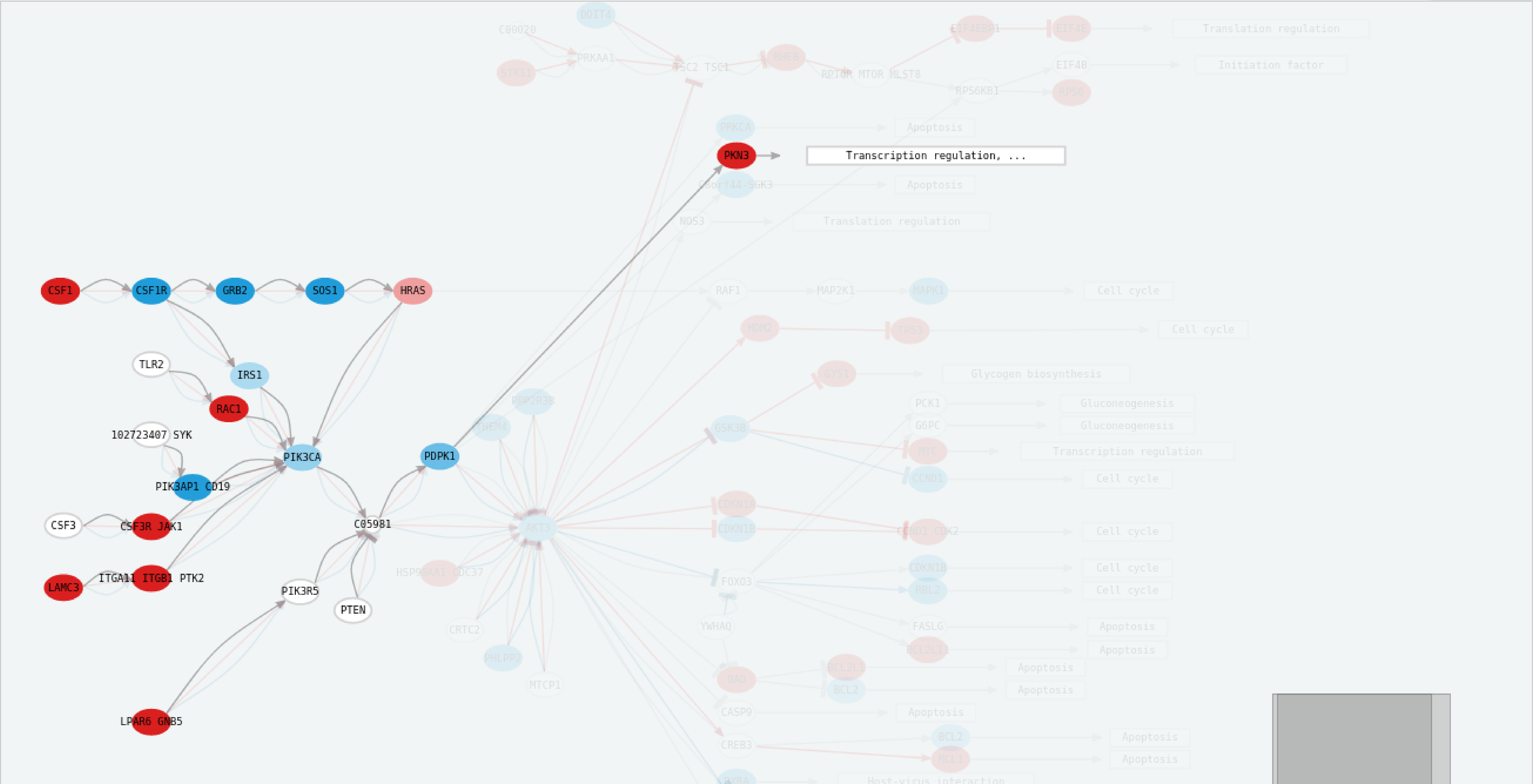

PI3K-Akt signaling pathway (hsa04151)

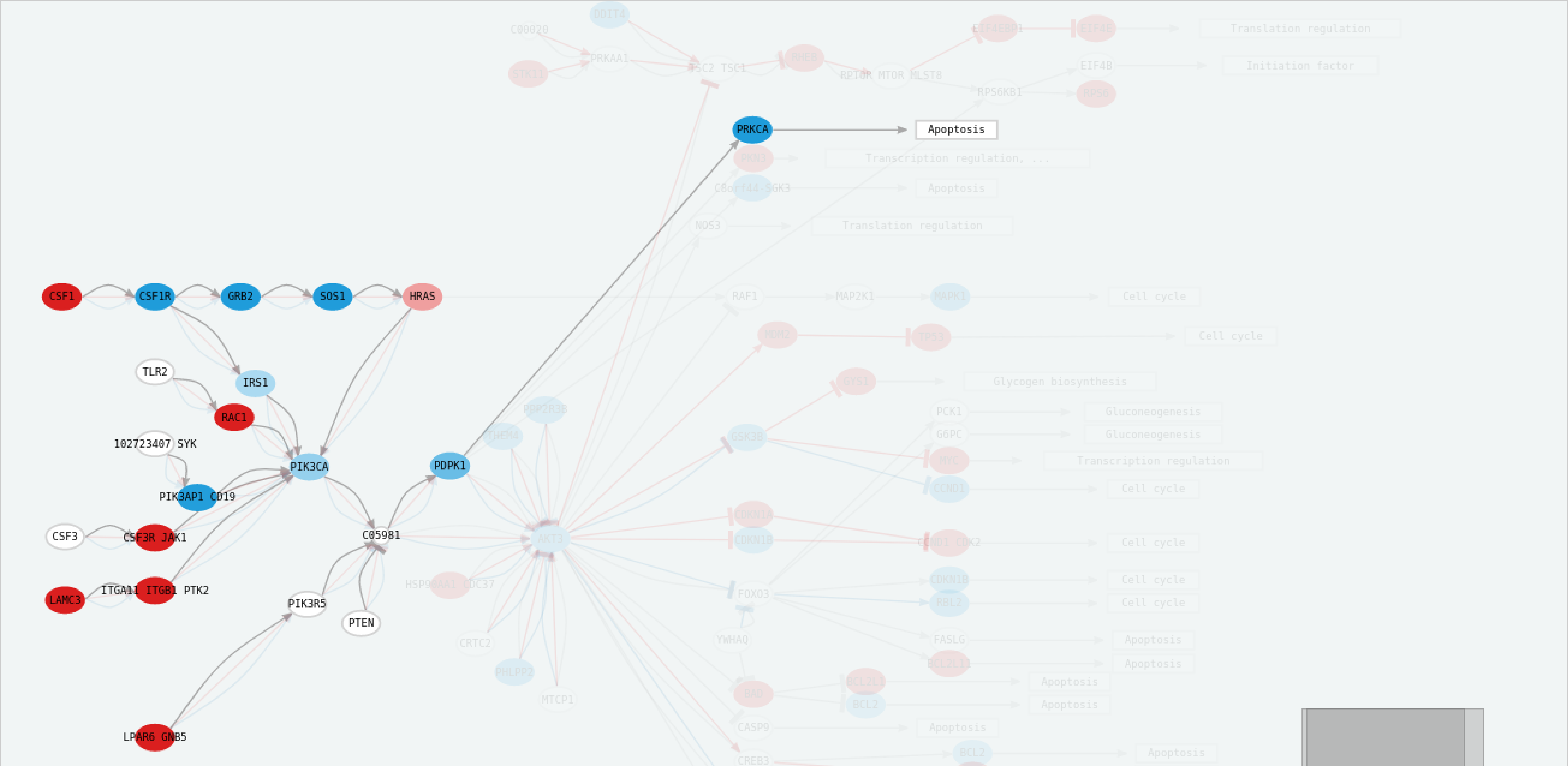

PI3K-Akt signaling pathway (hsa04151)

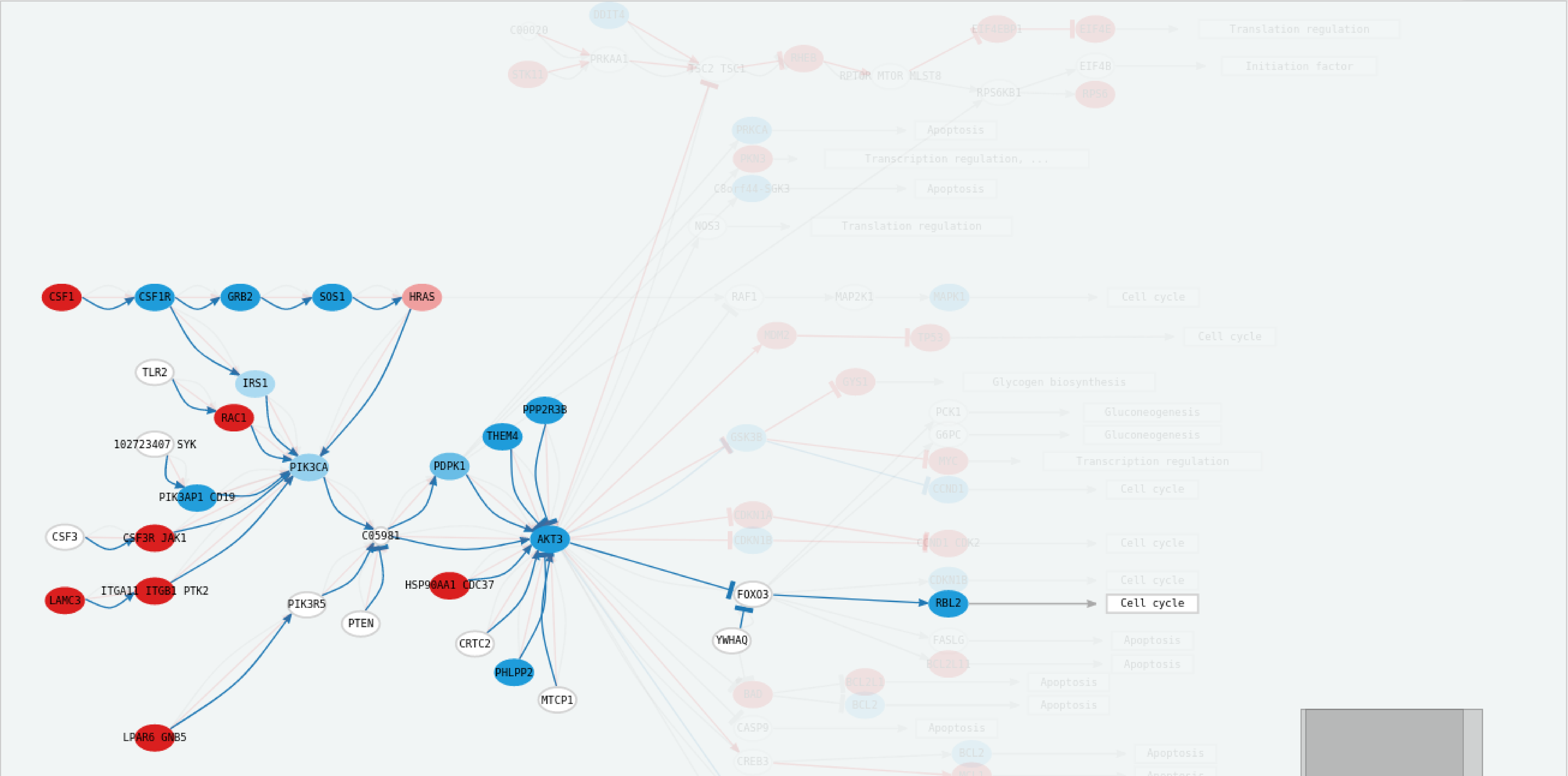

PI3K-Akt signaling pathway (hsa04151)

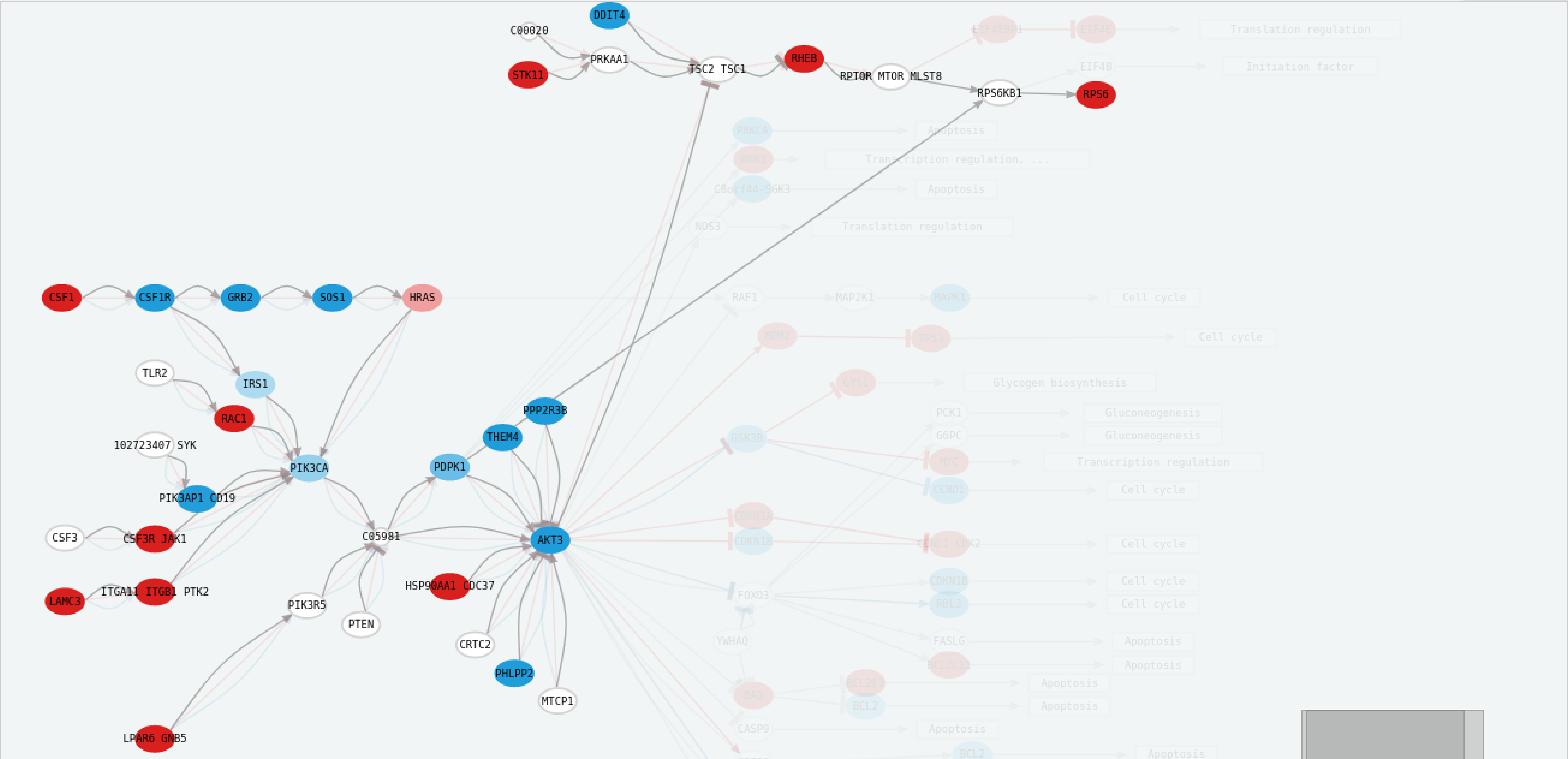

PI3K-Akt signaling pathway (hsa04151)

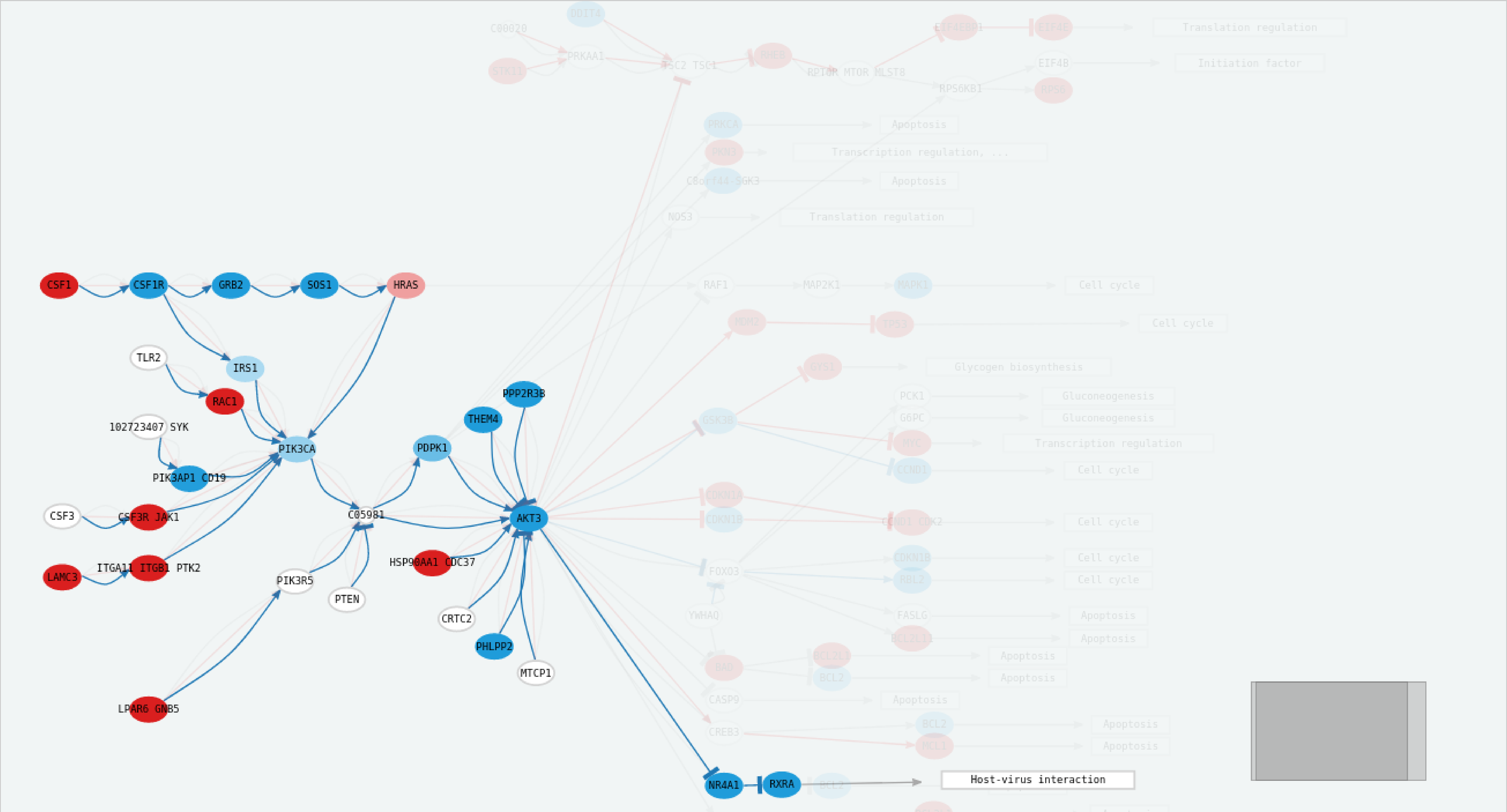

PI3K-Akt signaling pathway (hsa04151)

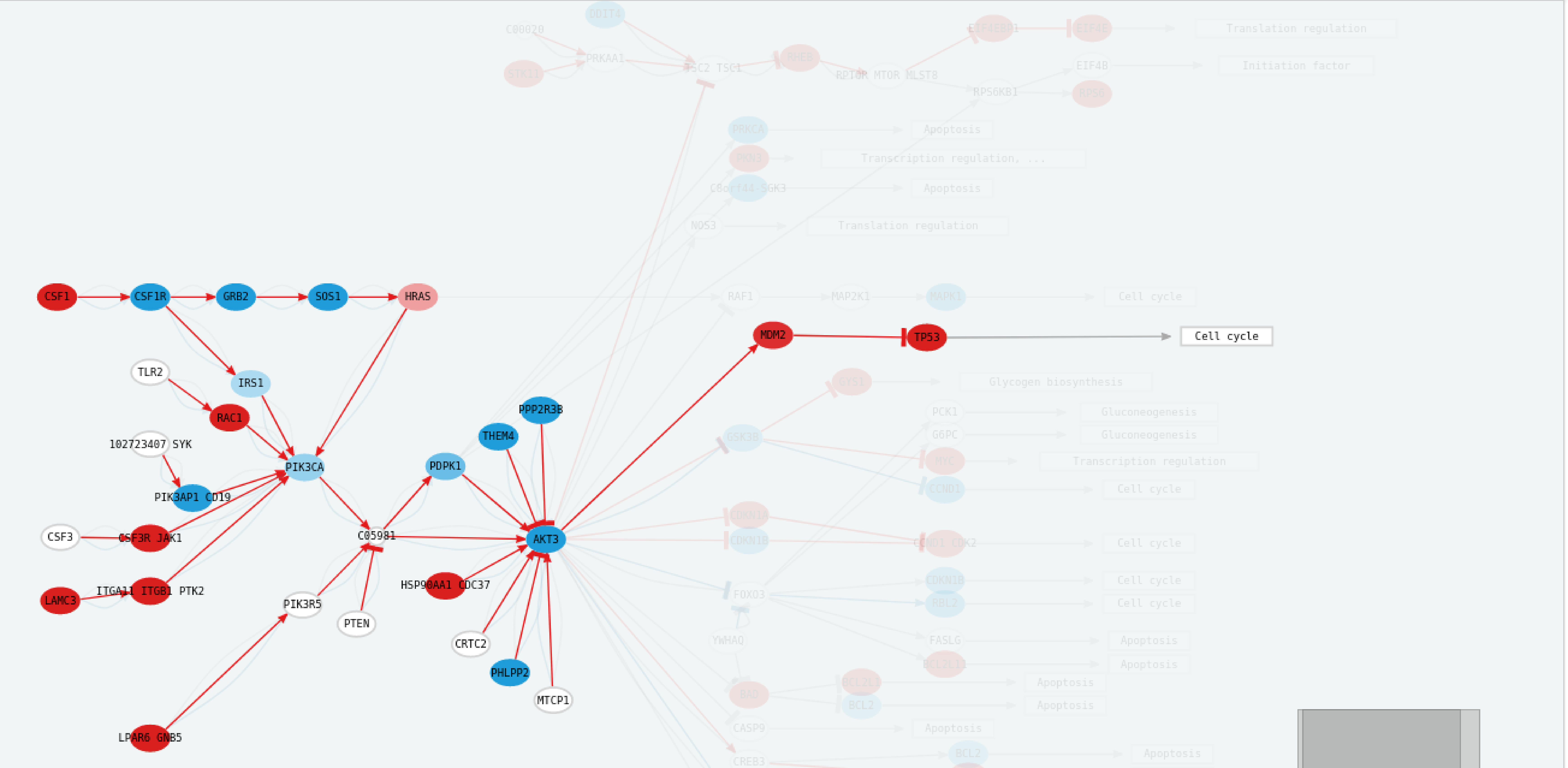

Rap1 signaling pathway (hsa04015)

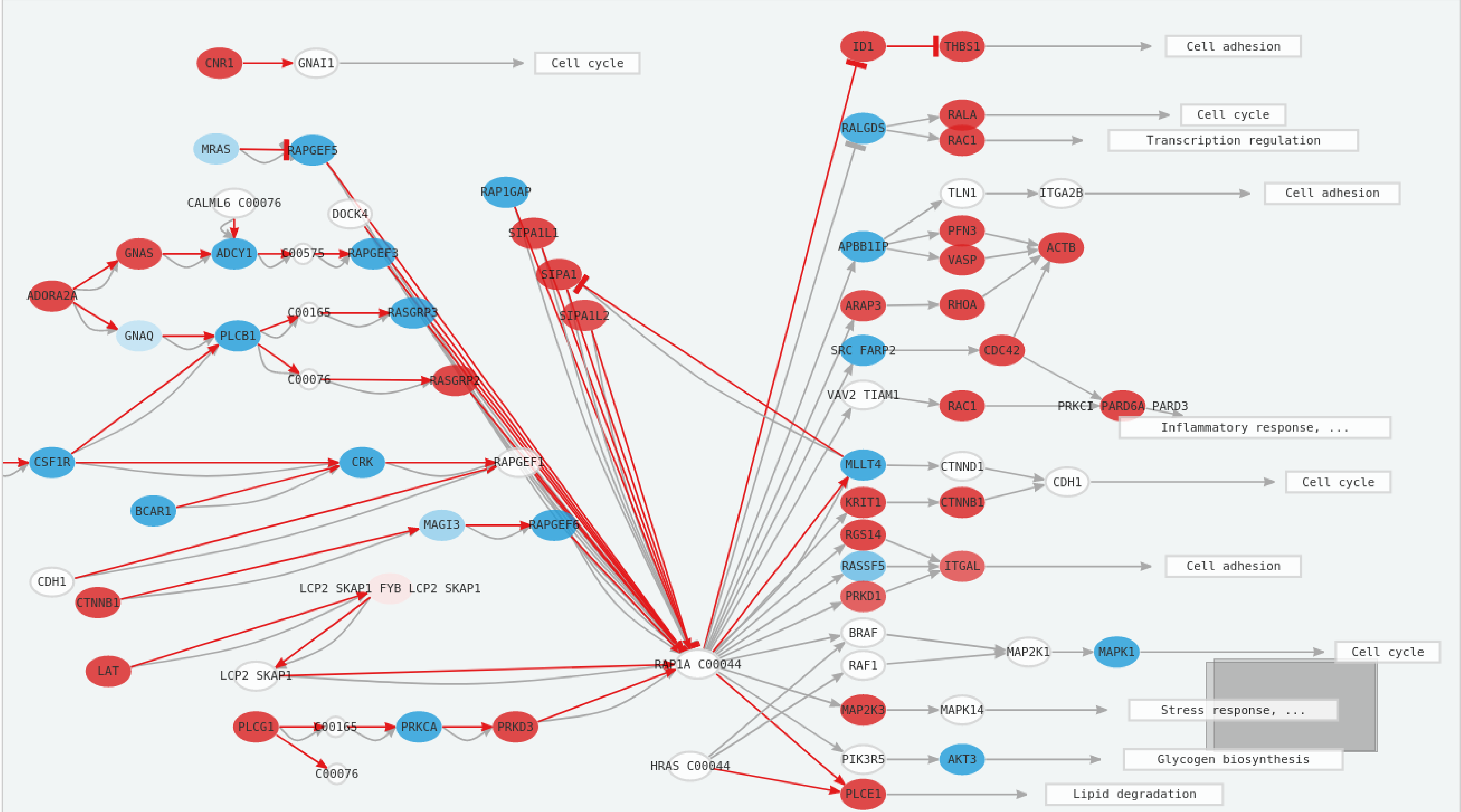

Rap1 signaling pathway (hsa04015)

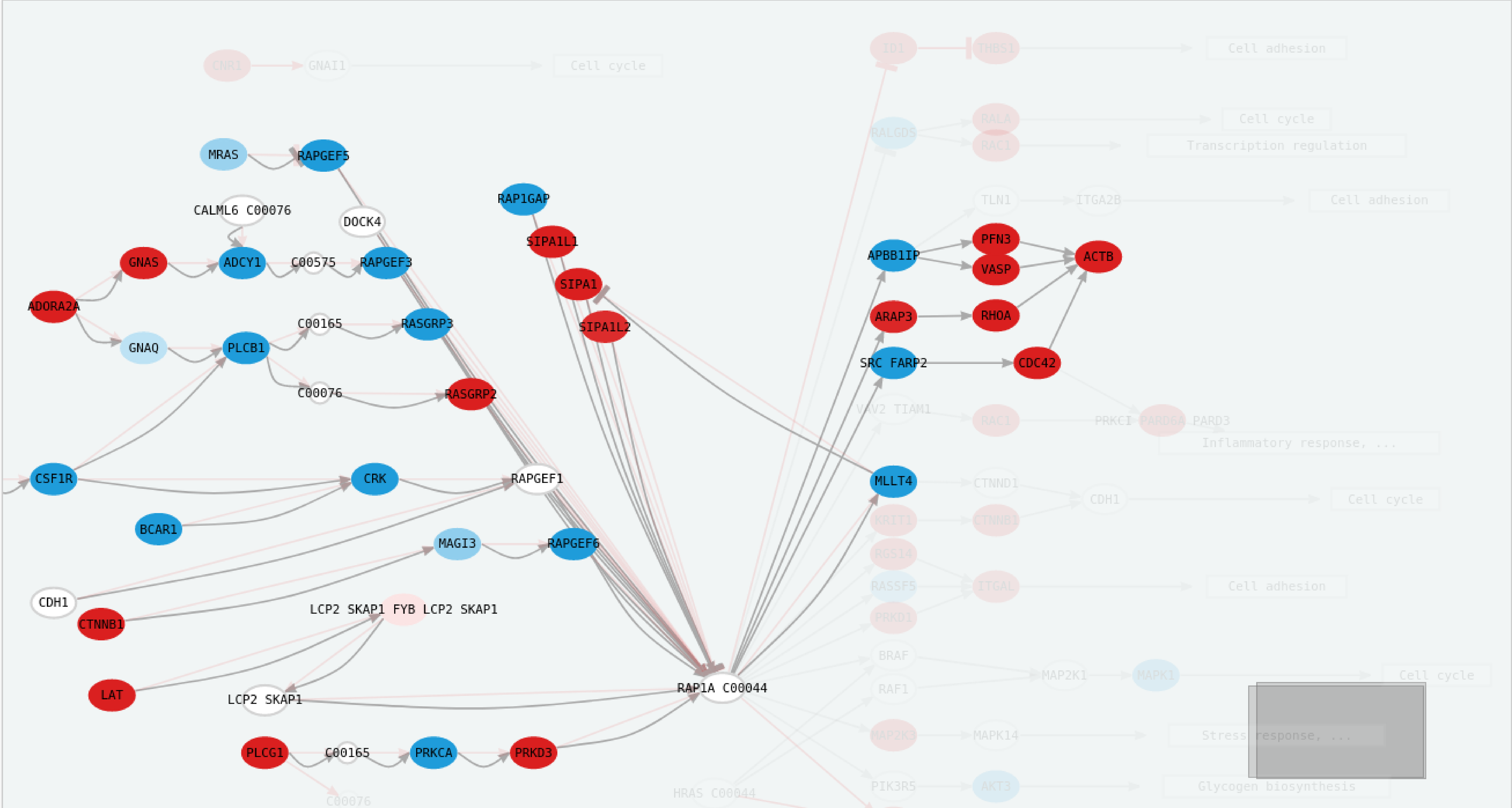

Rap1 signaling pathway (hsa04015)

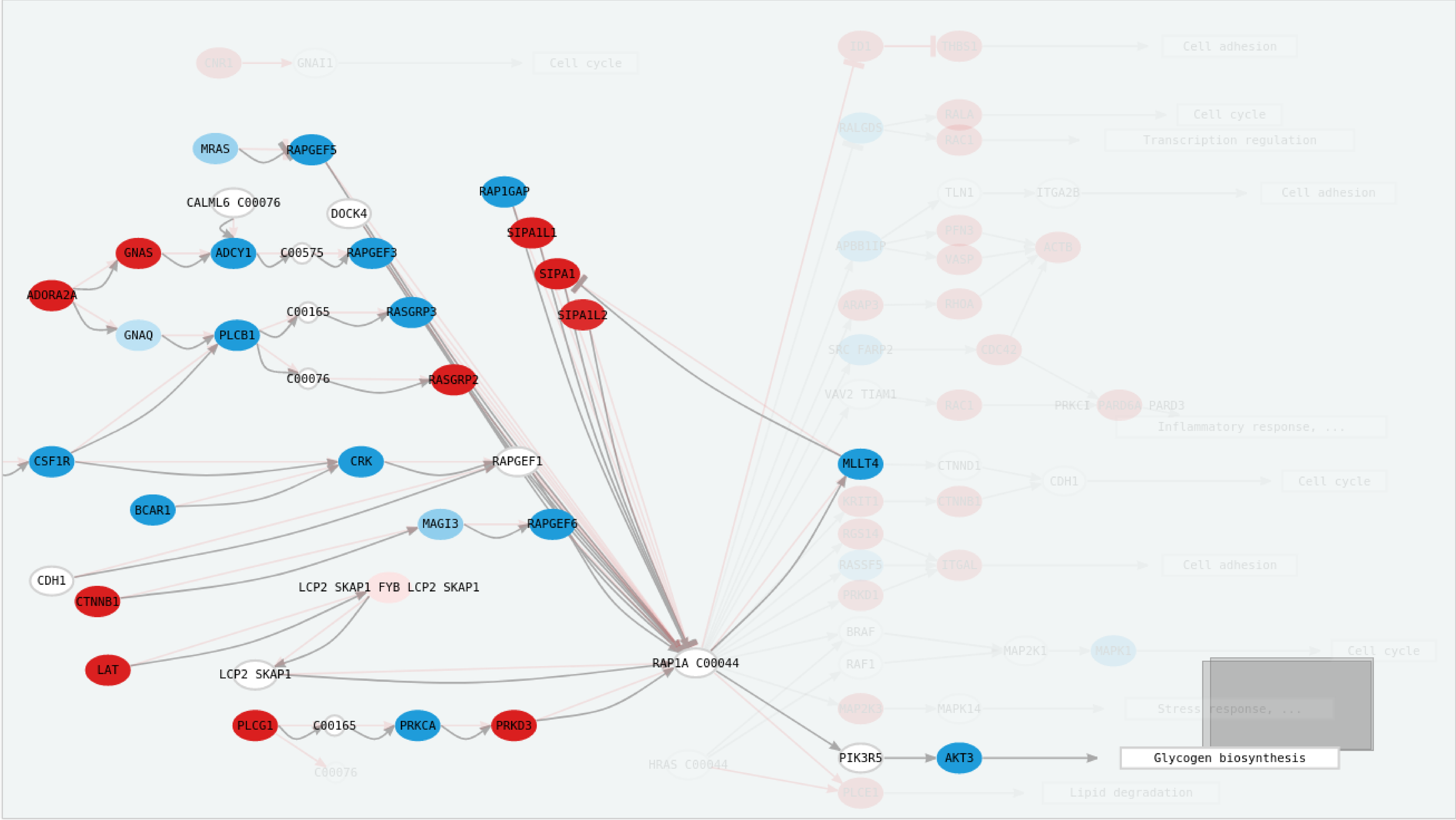

Rap1 signaling pathway (hsa04015)

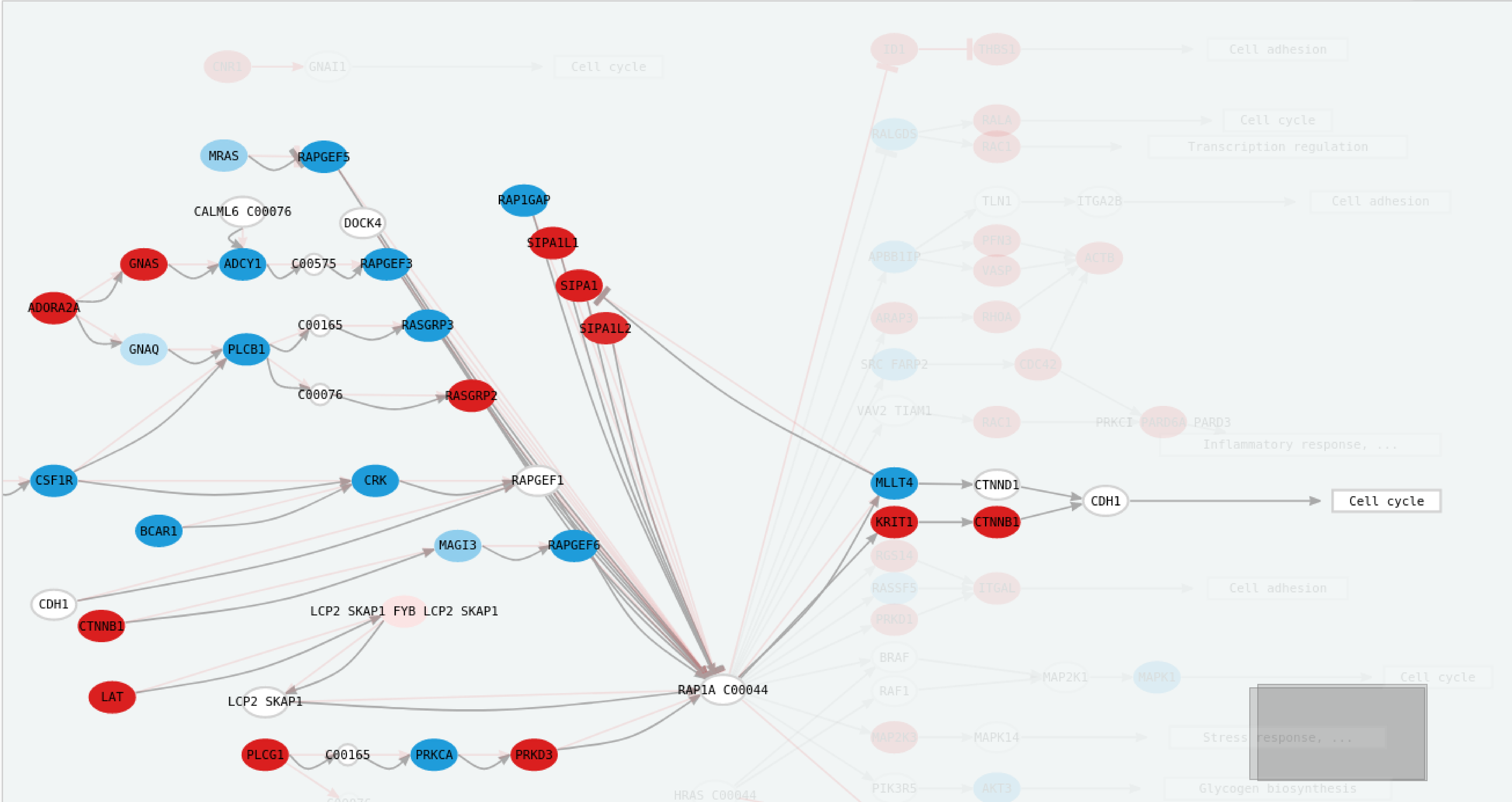

Rap1 signaling pathway (hsa04015)

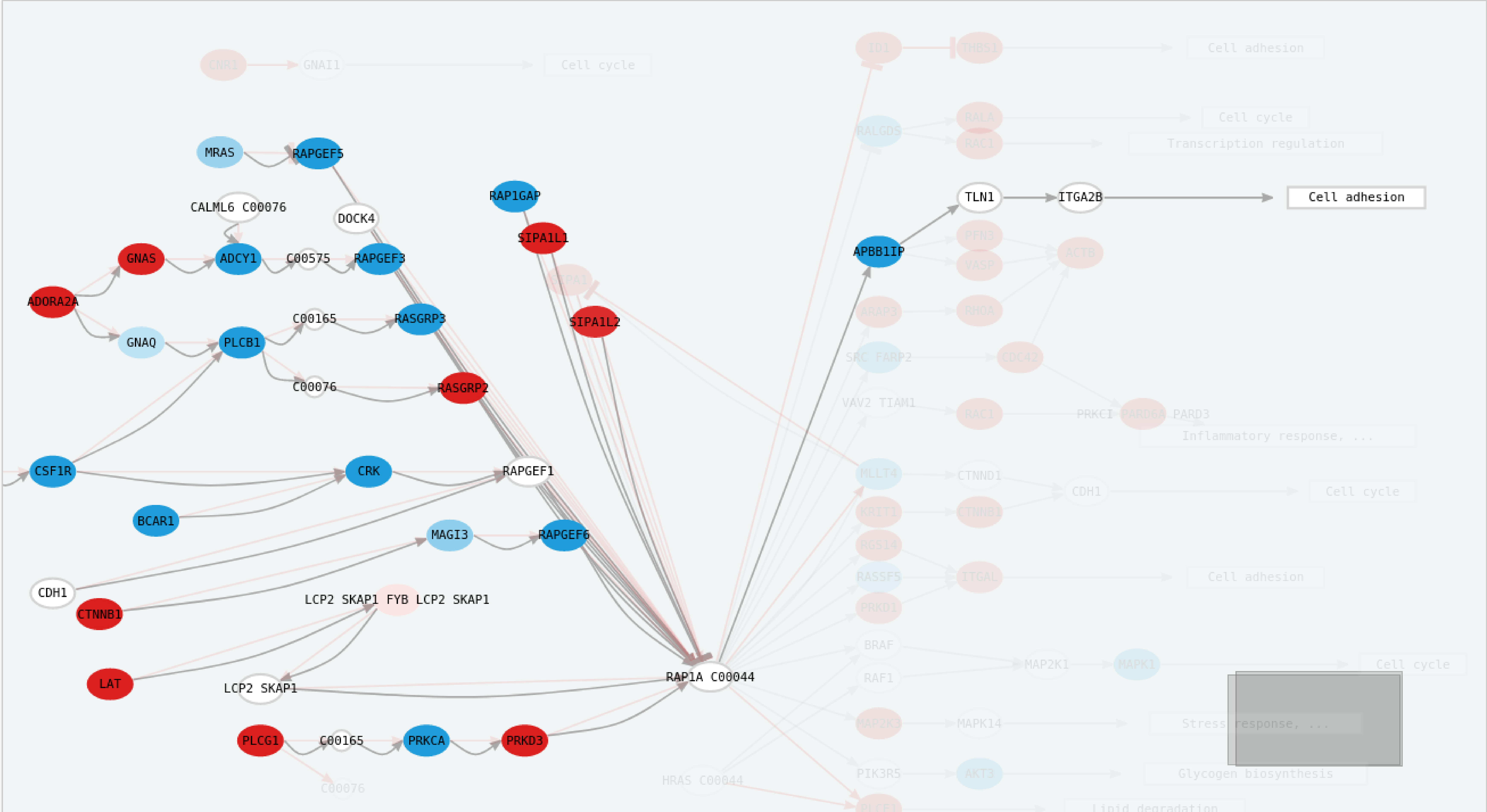

Rap1 signaling pathway (hsa04015)

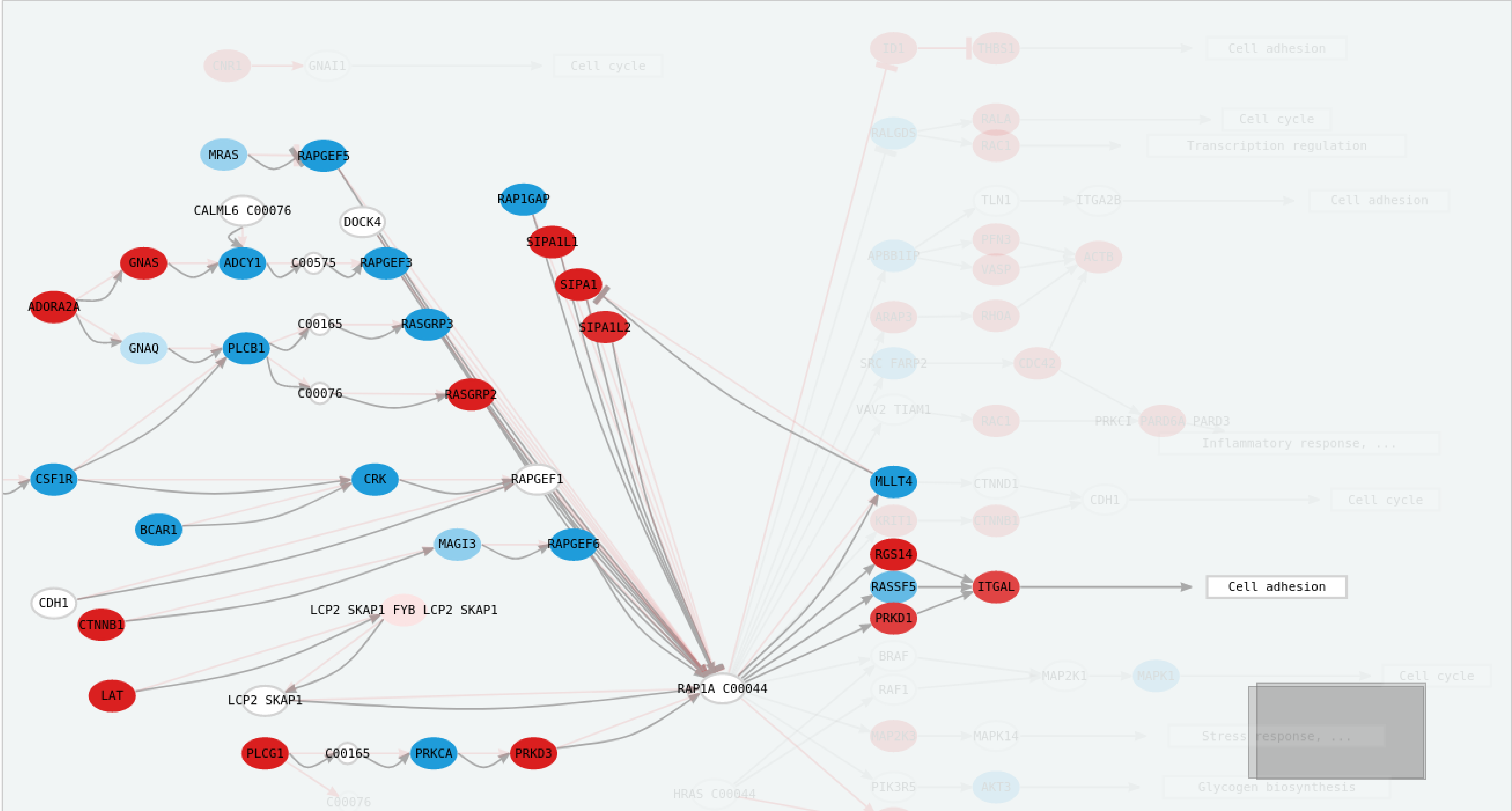

Rap1 signaling pathway (hsa04015)

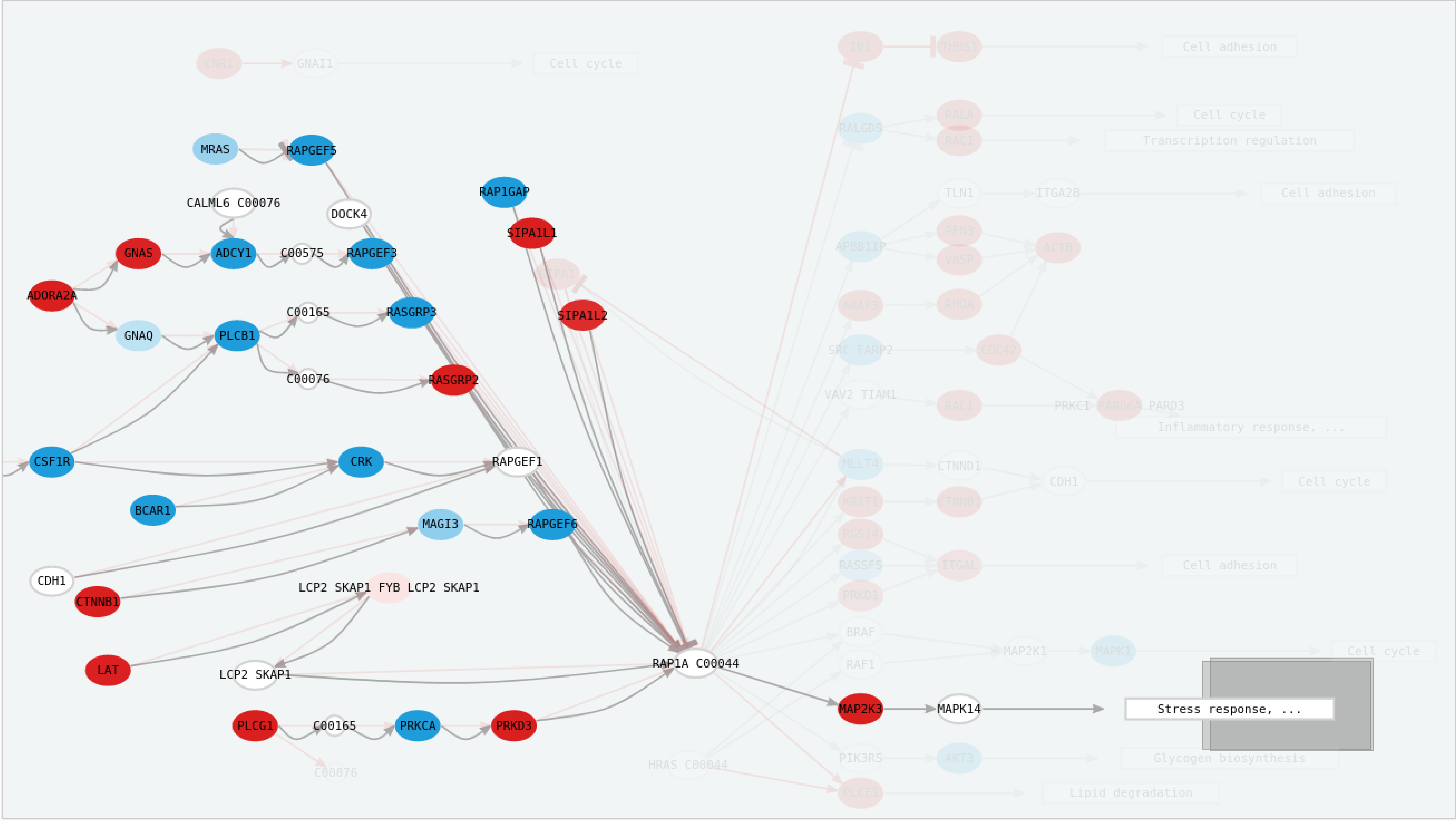

Rap1 signaling pathway (hsa04015)

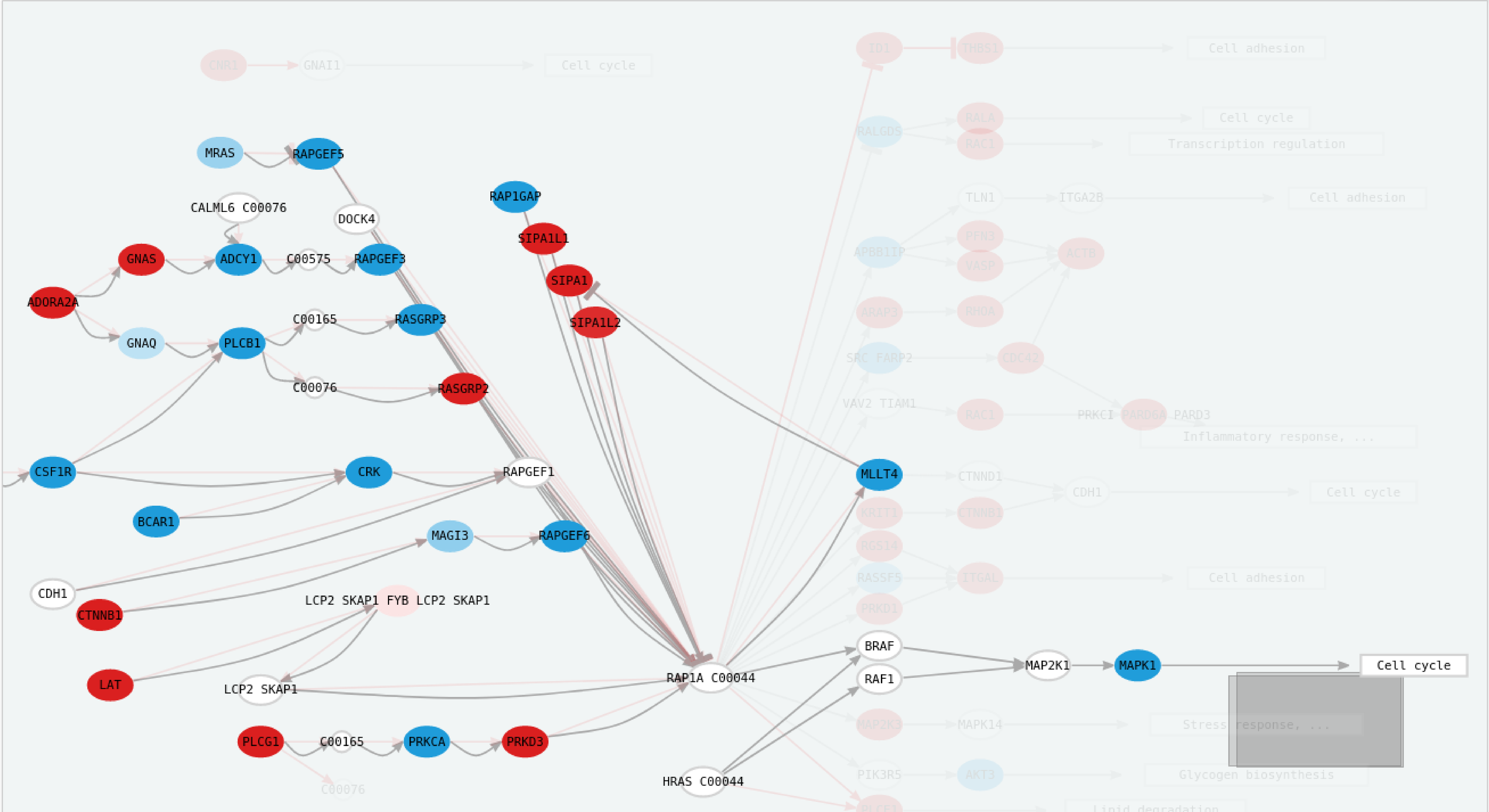

Rap1 signaling pathway (hsa04015)

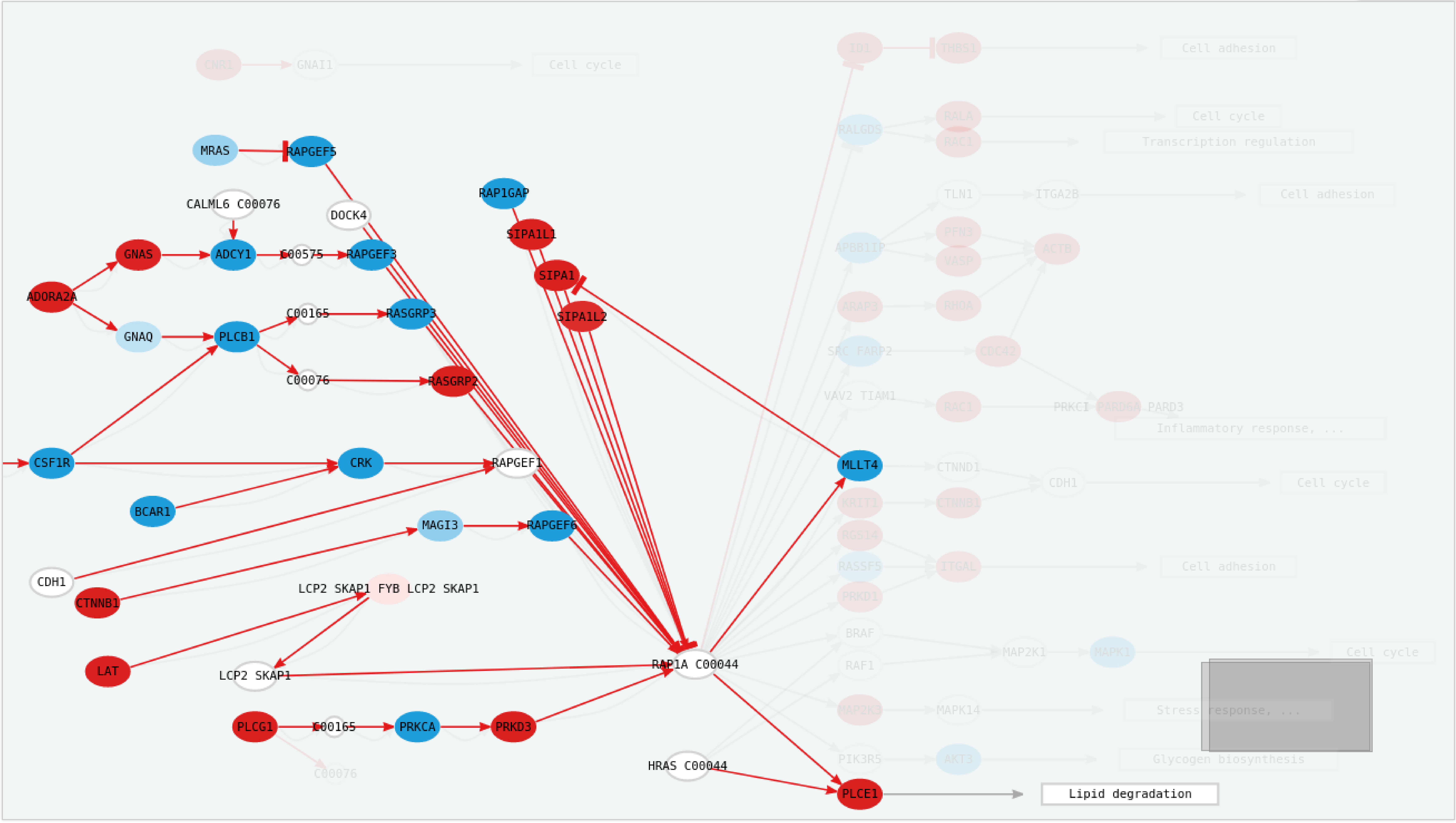

Rap1 signaling pathway (hsa04015)

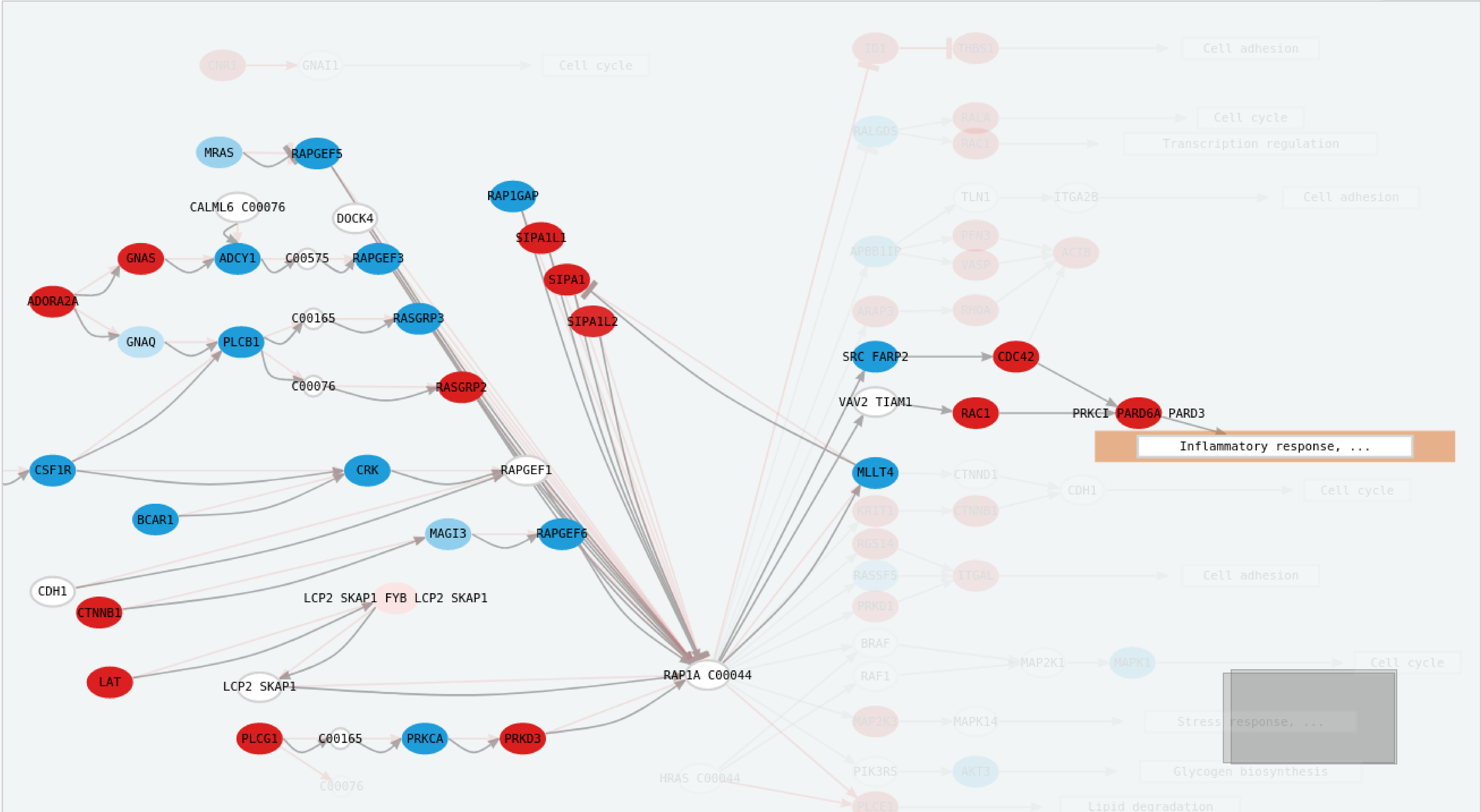

Rap1 signaling pathway (hsa04015)

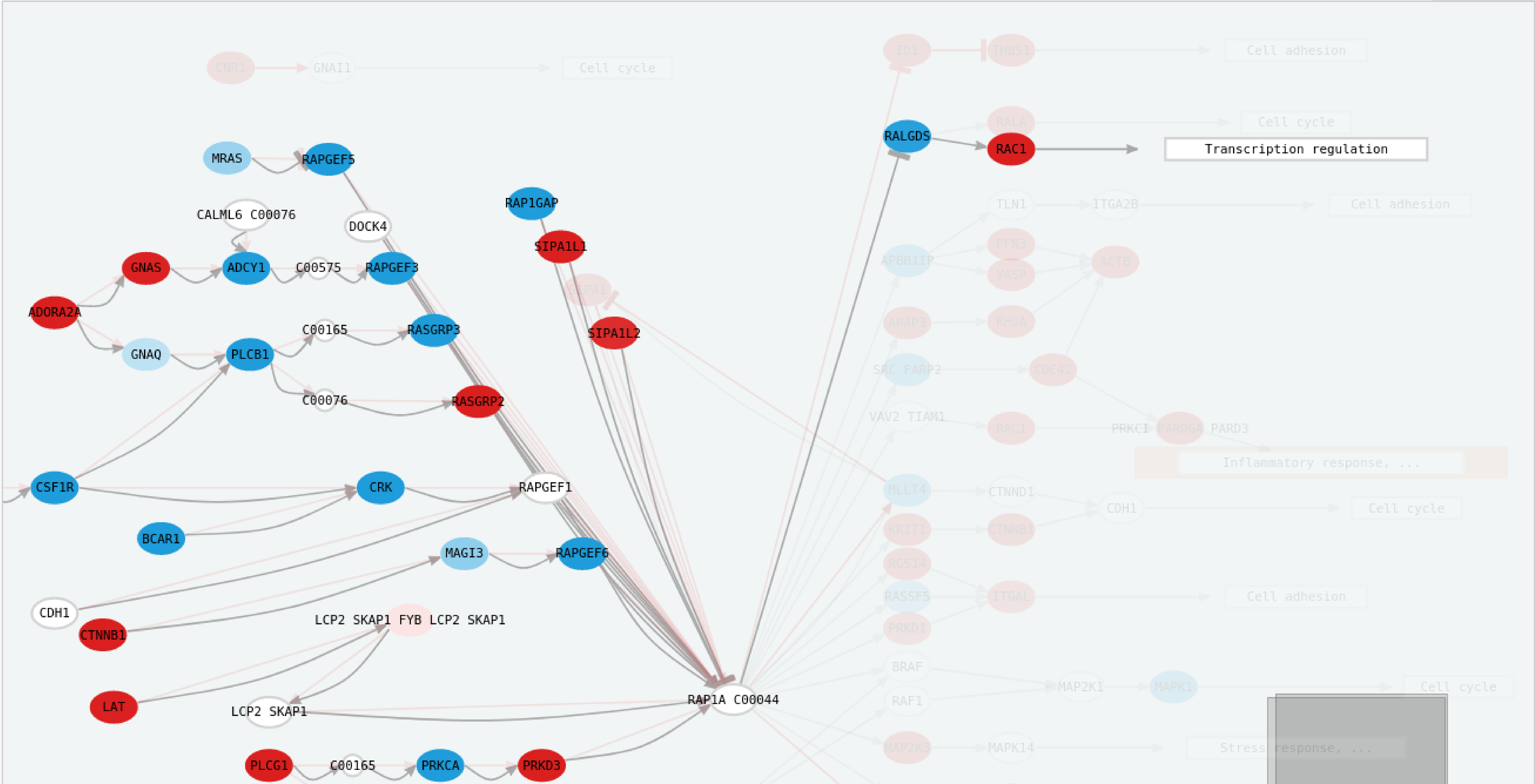

Rap1 signaling pathway (hsa04015)

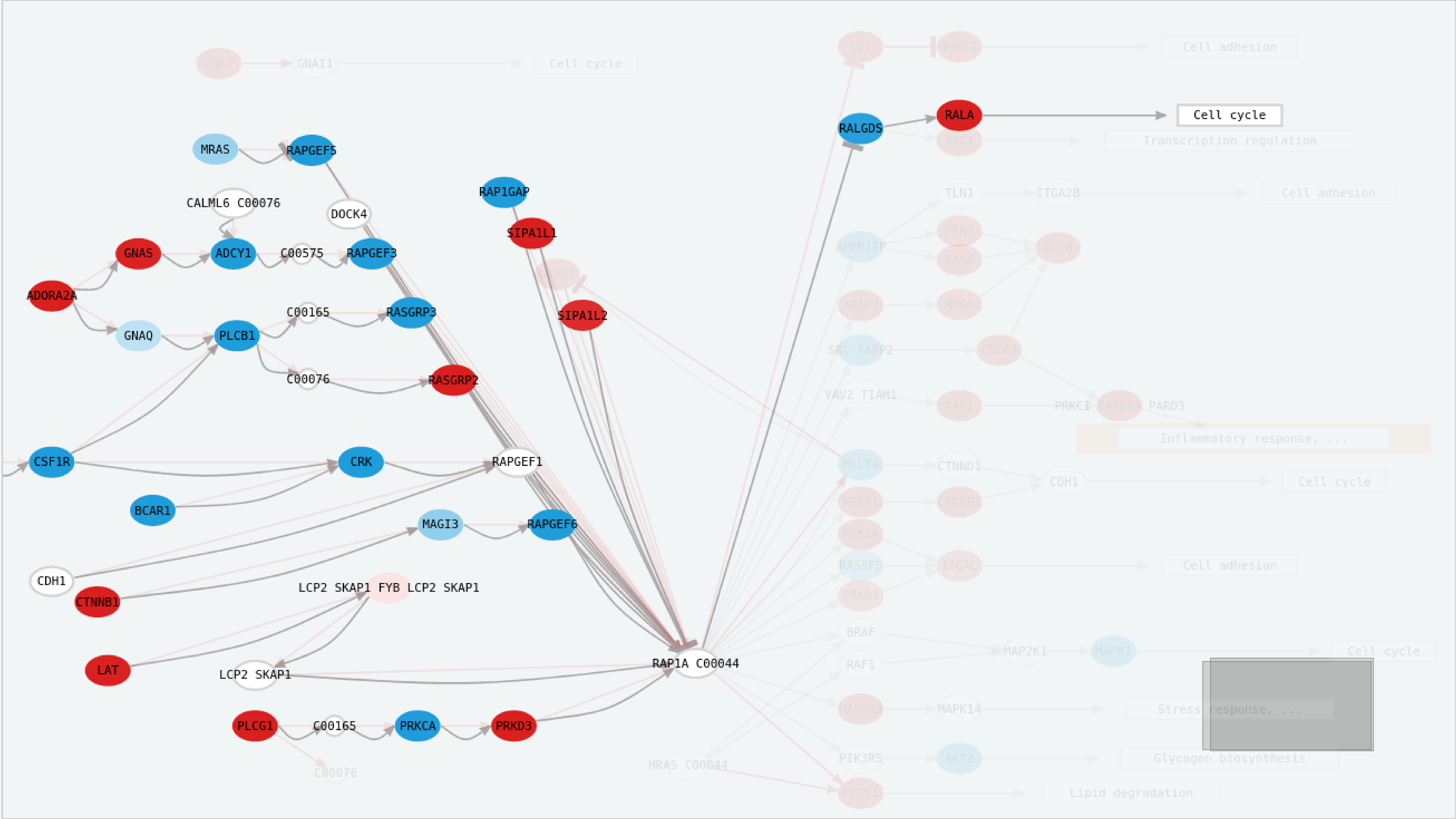

Rap1 signaling pathway (hsa04015)

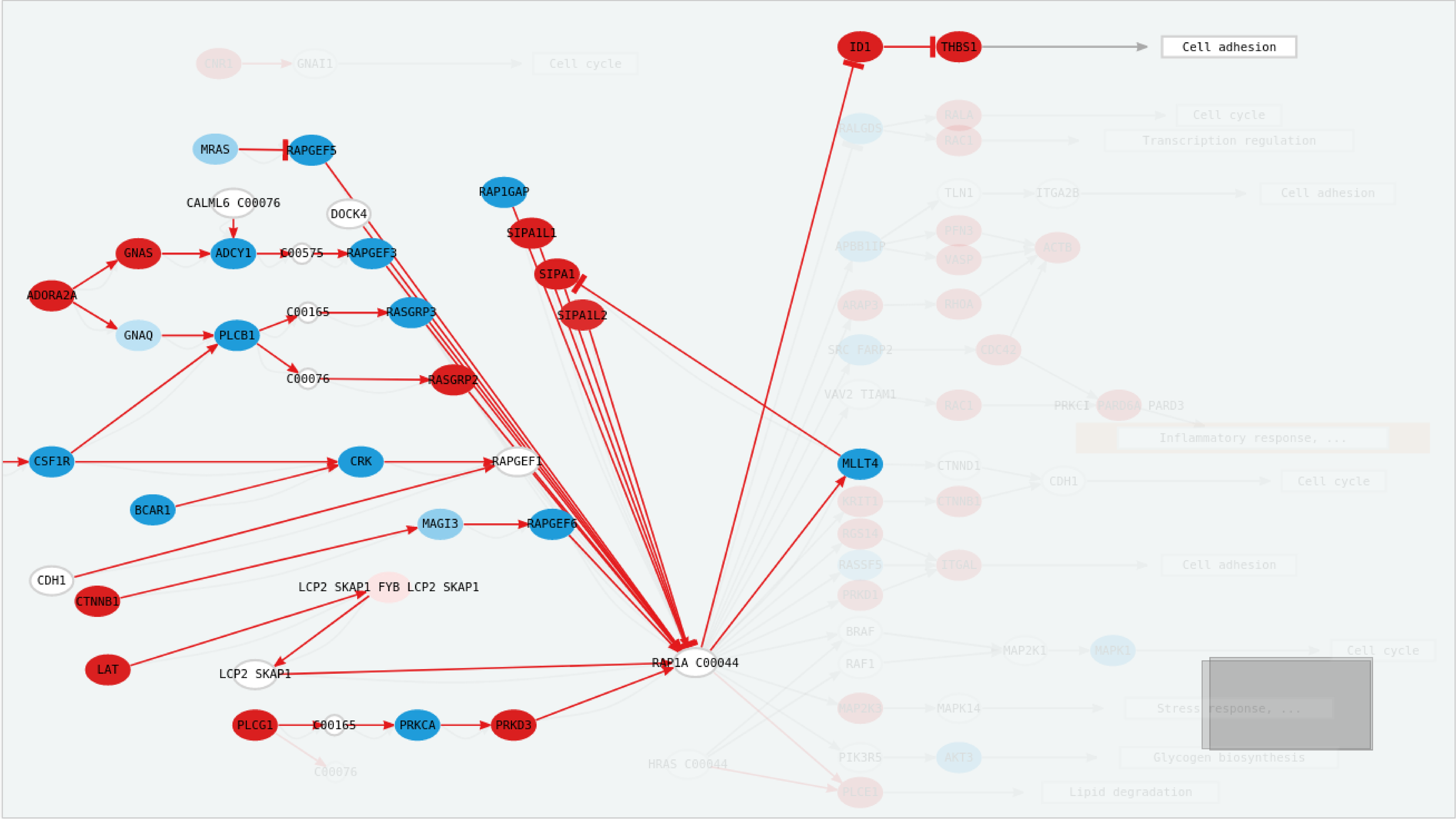

Ras signaling pathway (hsa04014)

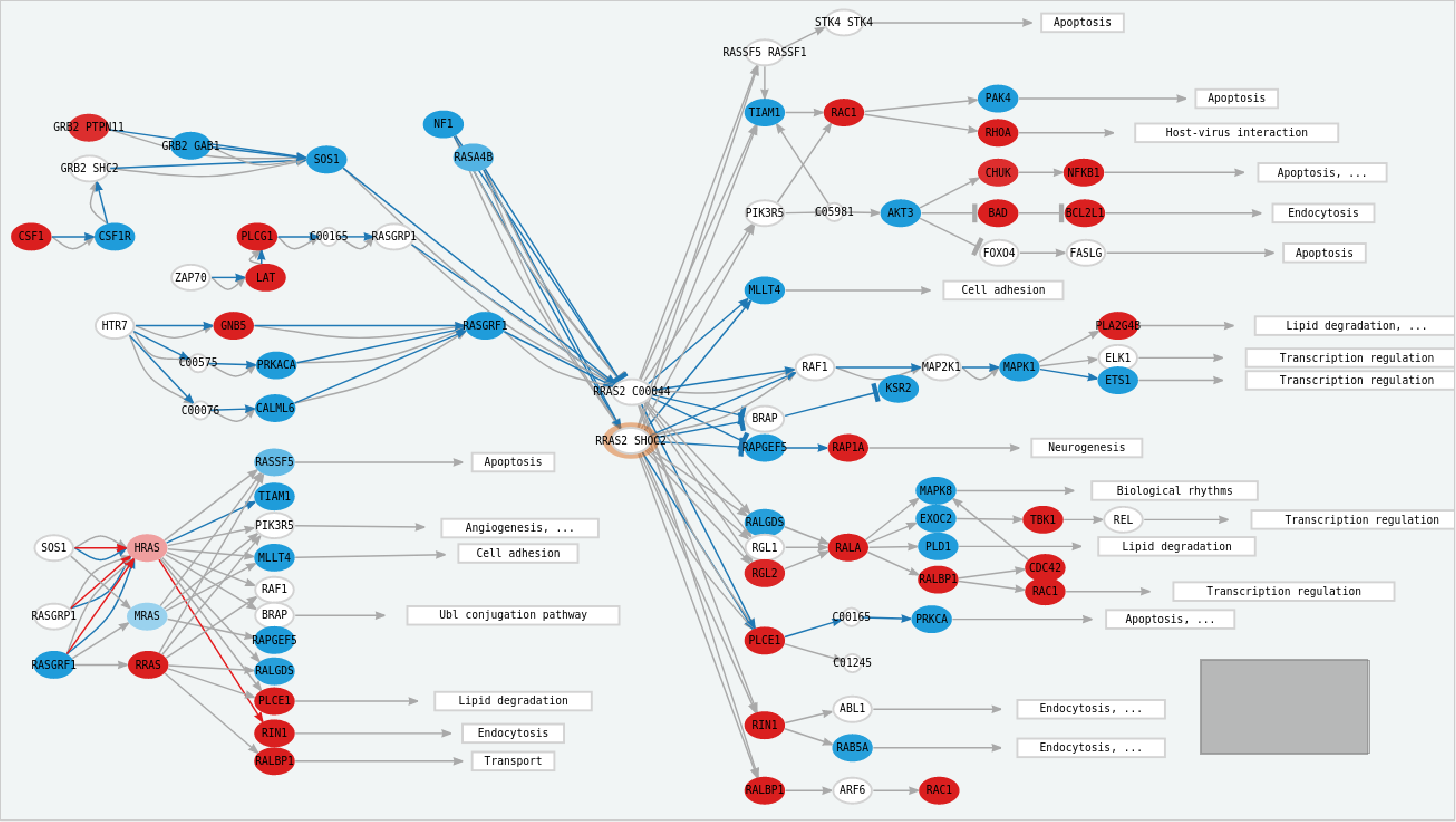

Ras signaling pathway (hsa04014)

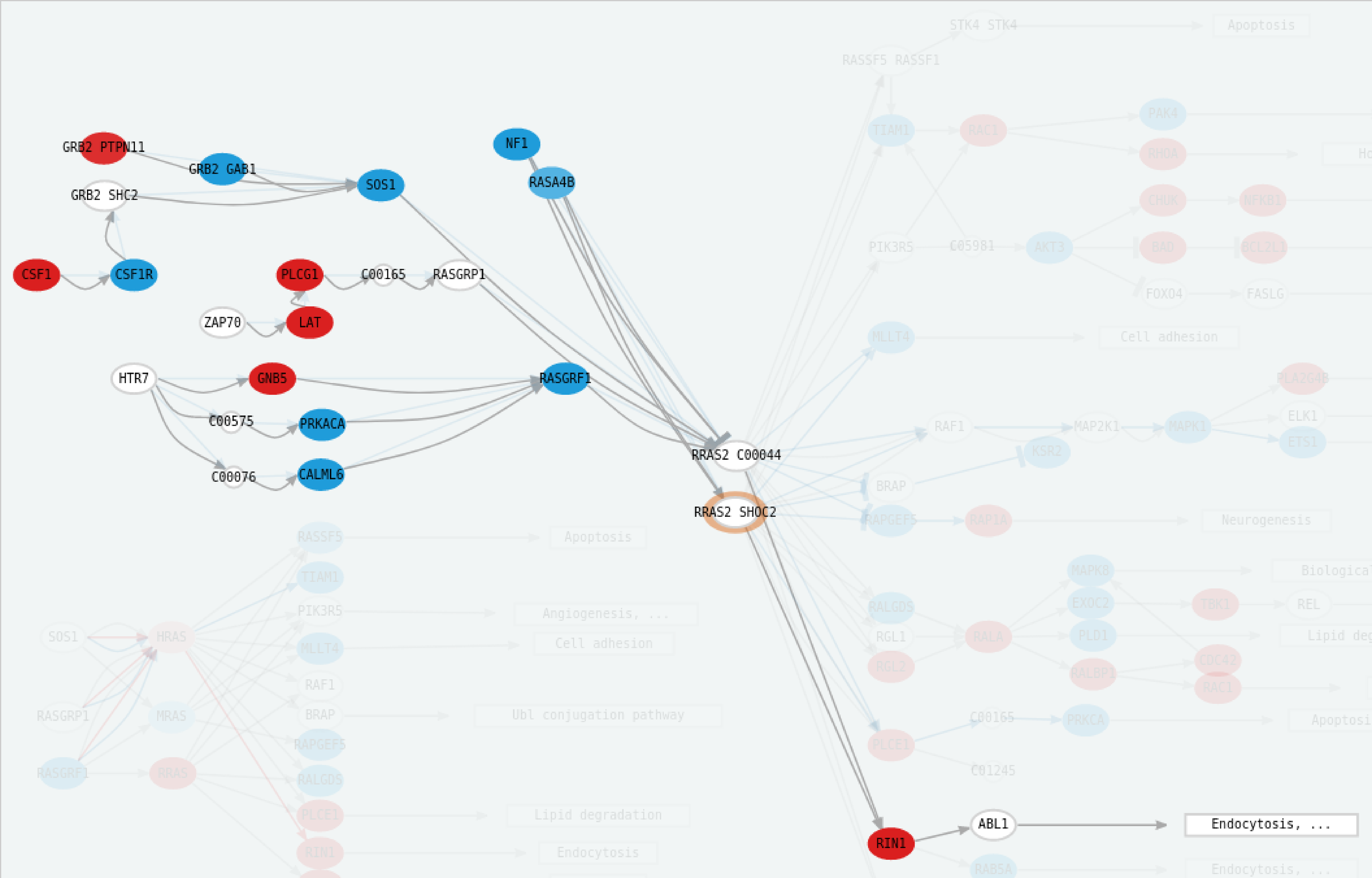

Ras signaling pathway (hsa04014)

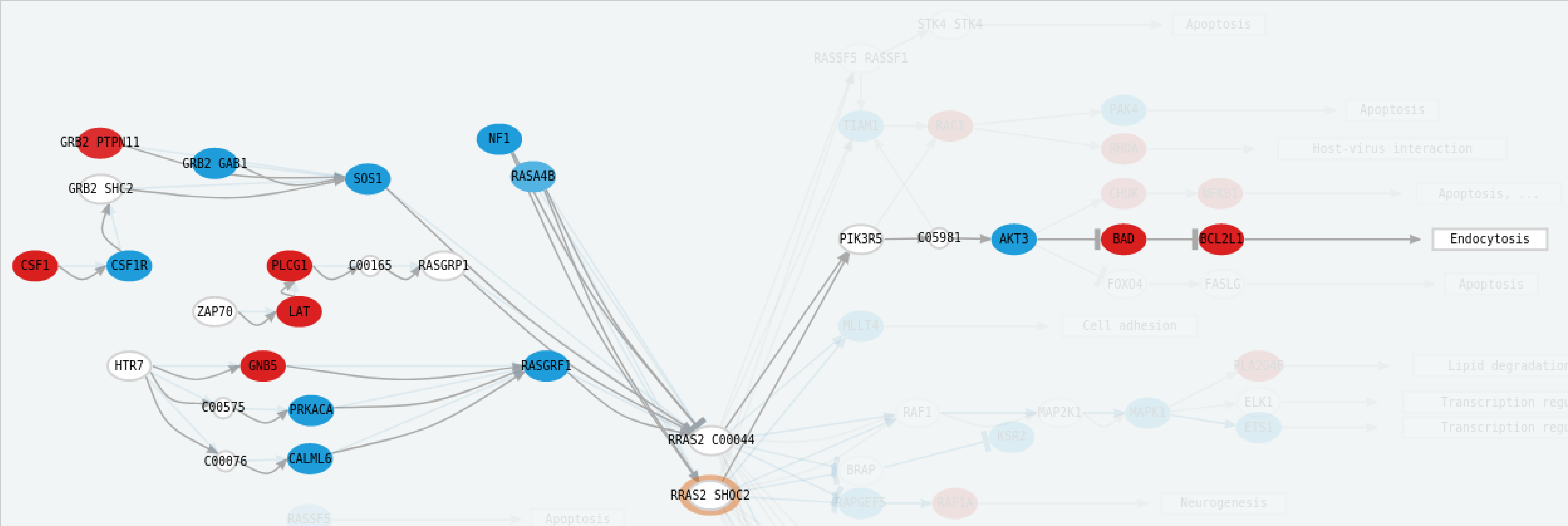

Ras signaling pathway (hsa04014)

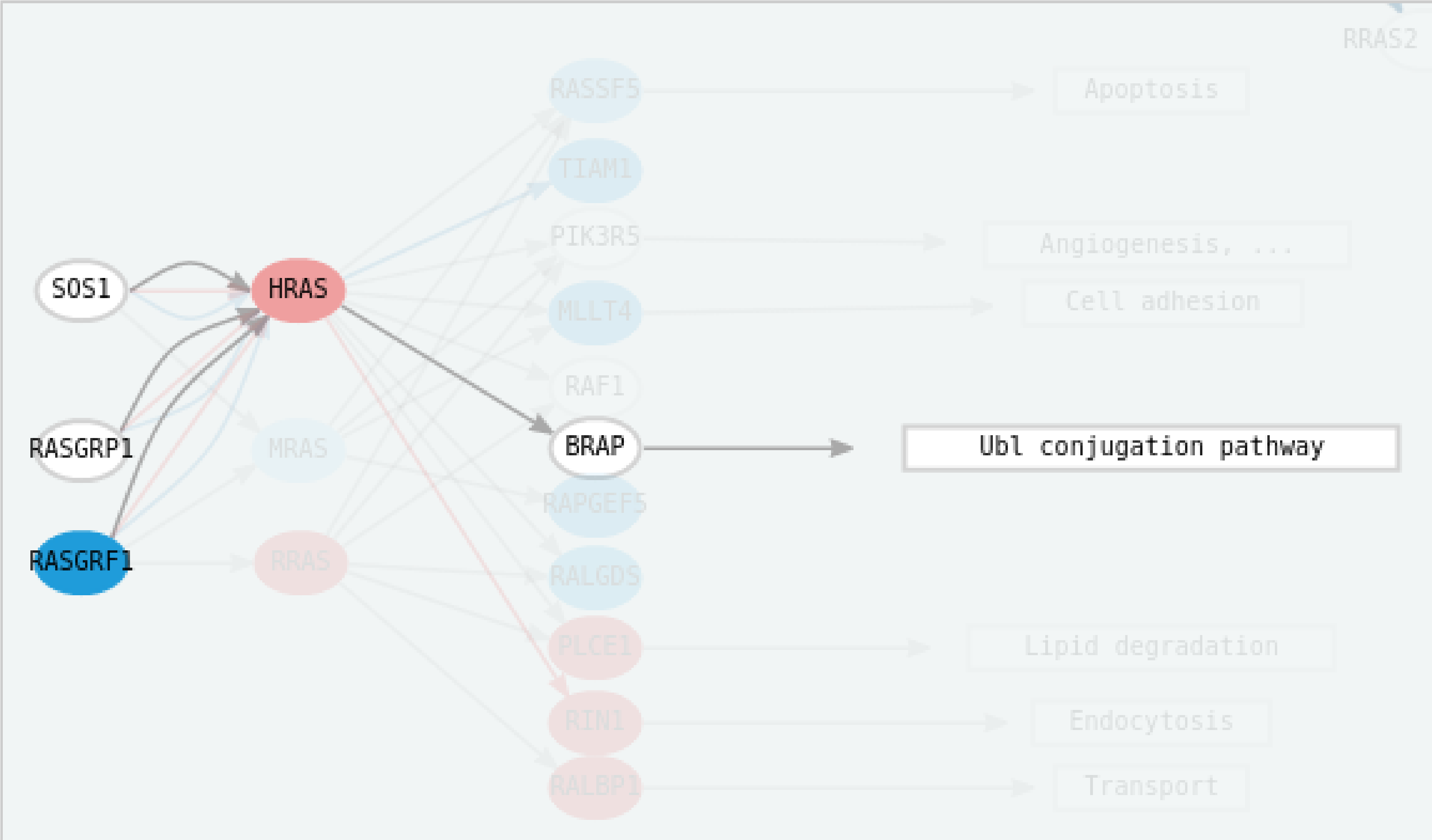

Ras signaling pathway (hsa04014)

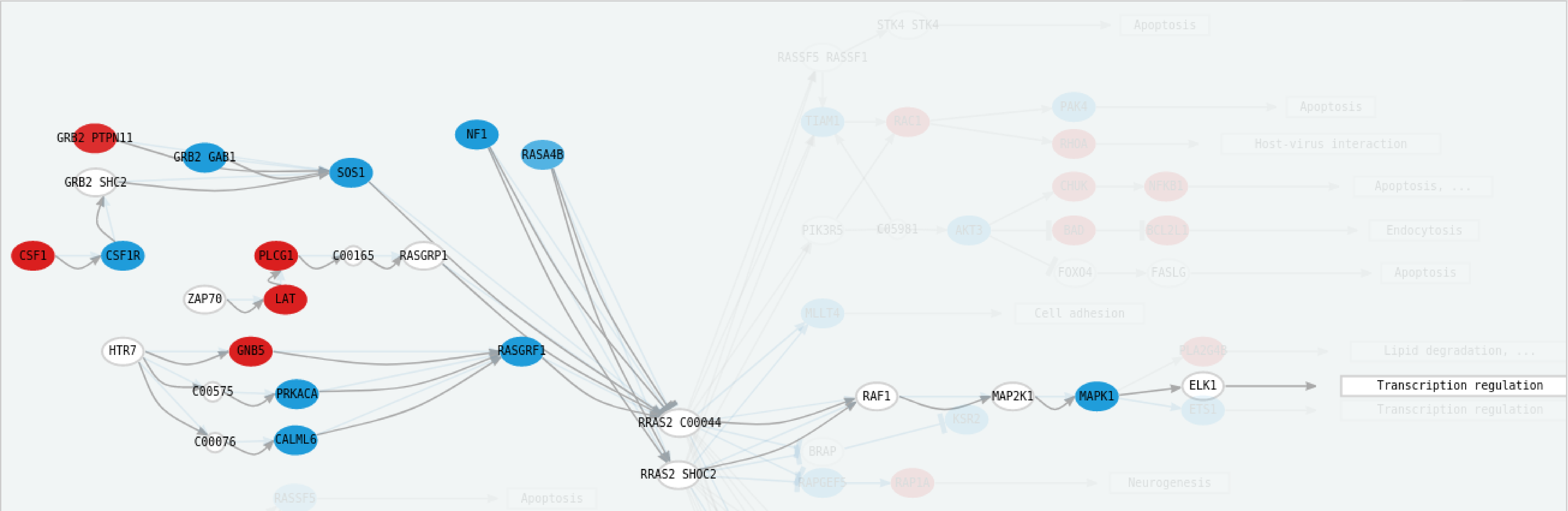

Ras signaling pathway (hsa04014)

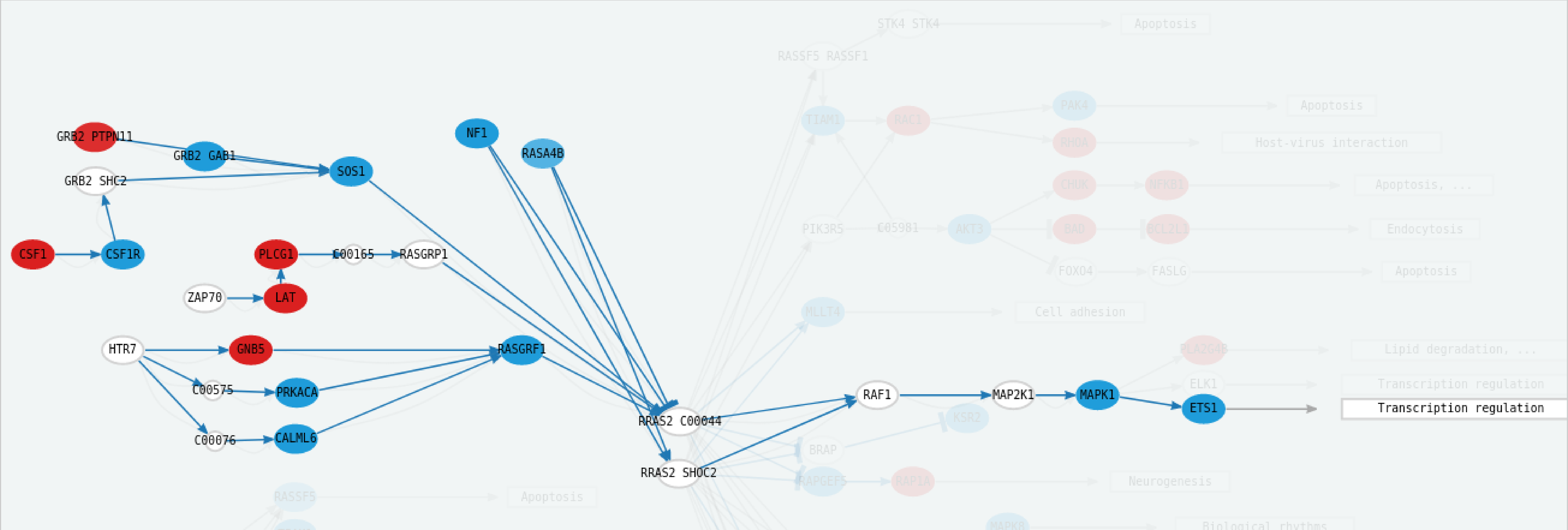

Ras signaling pathway (hsa04014)

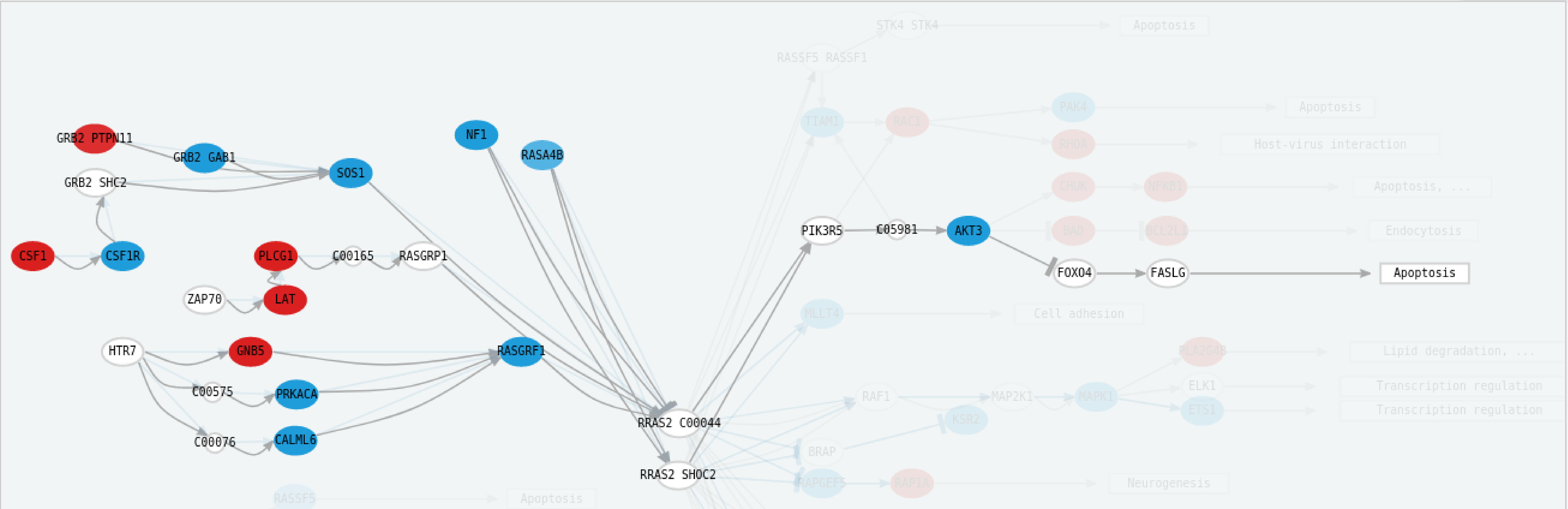

Ras signaling pathway (hsa04014)

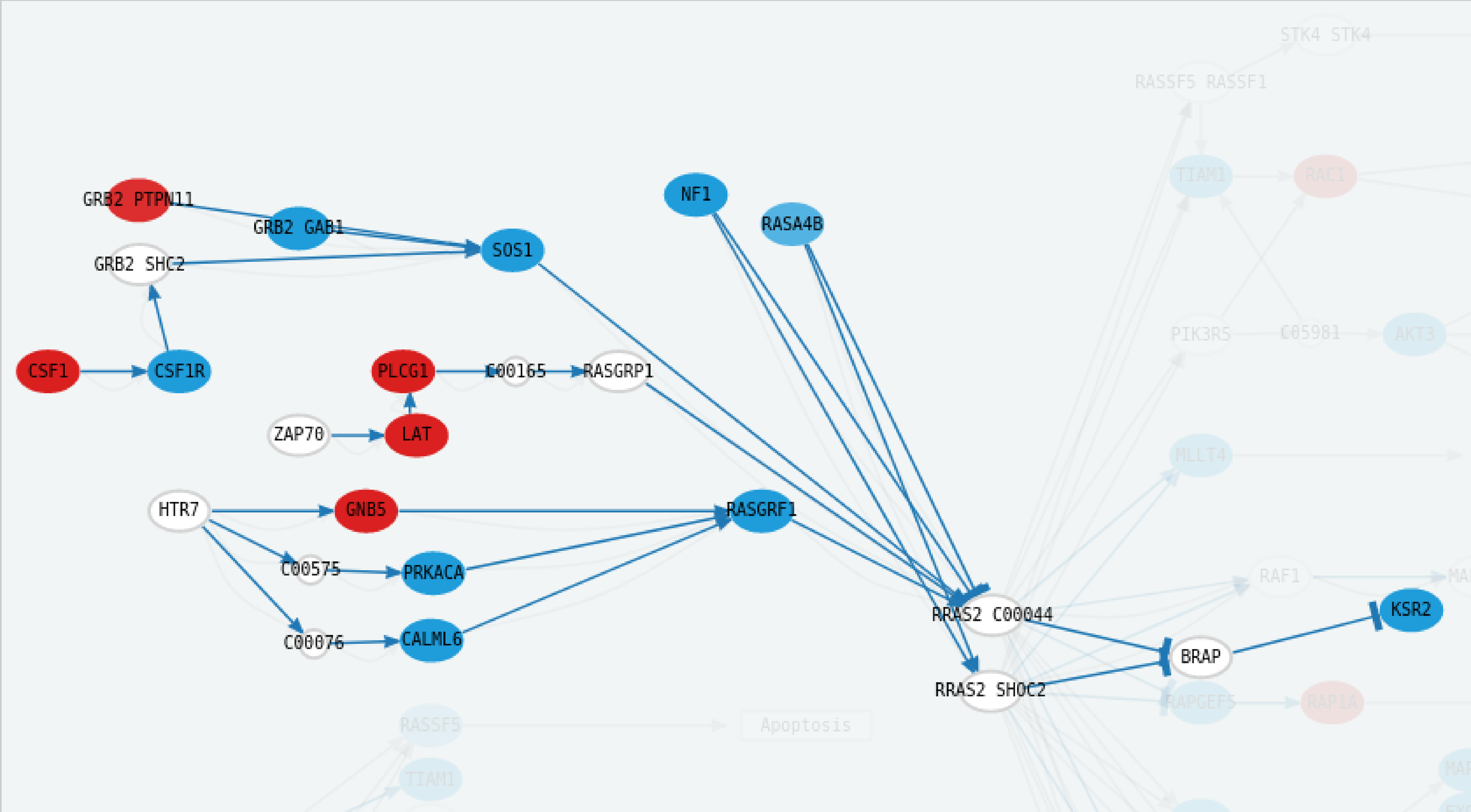

Ras signaling pathway (hsa04014)

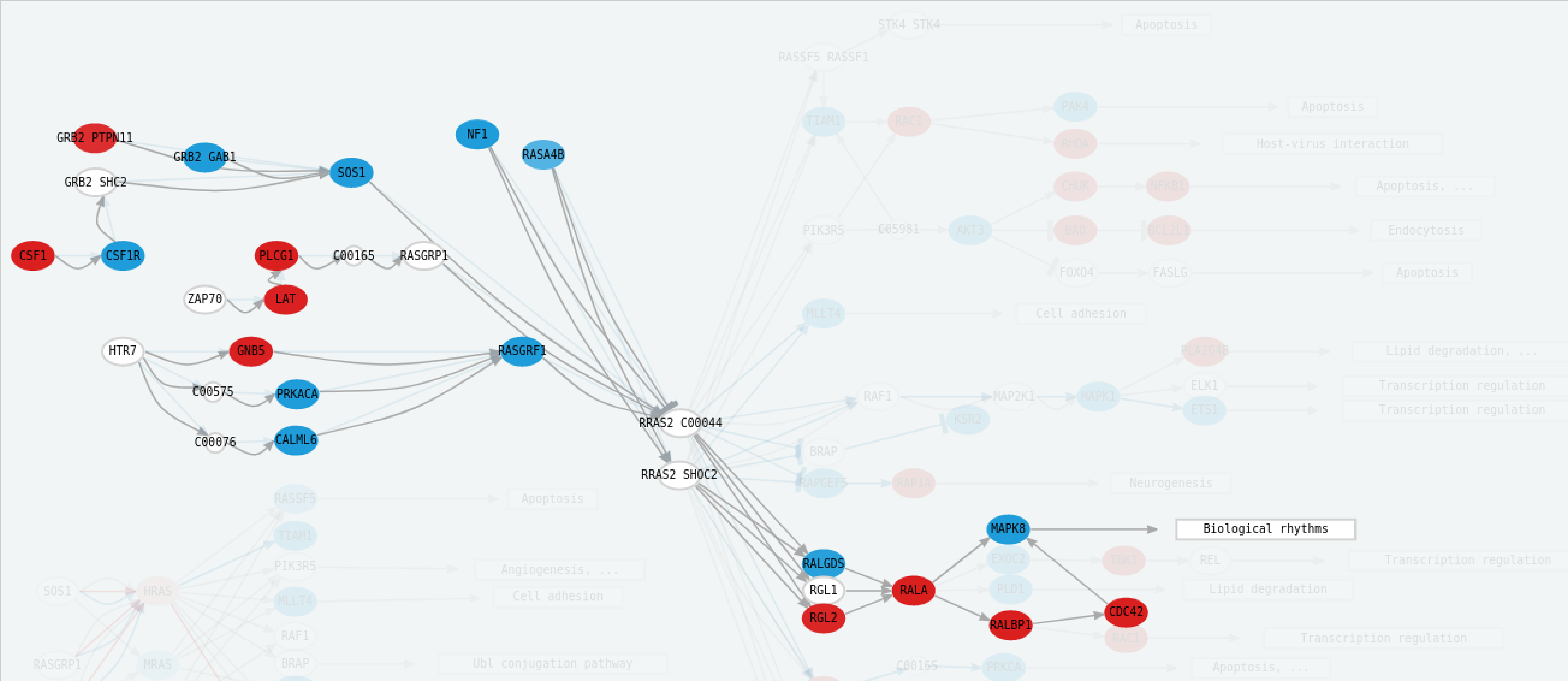

Ras signaling pathway (hsa04014)

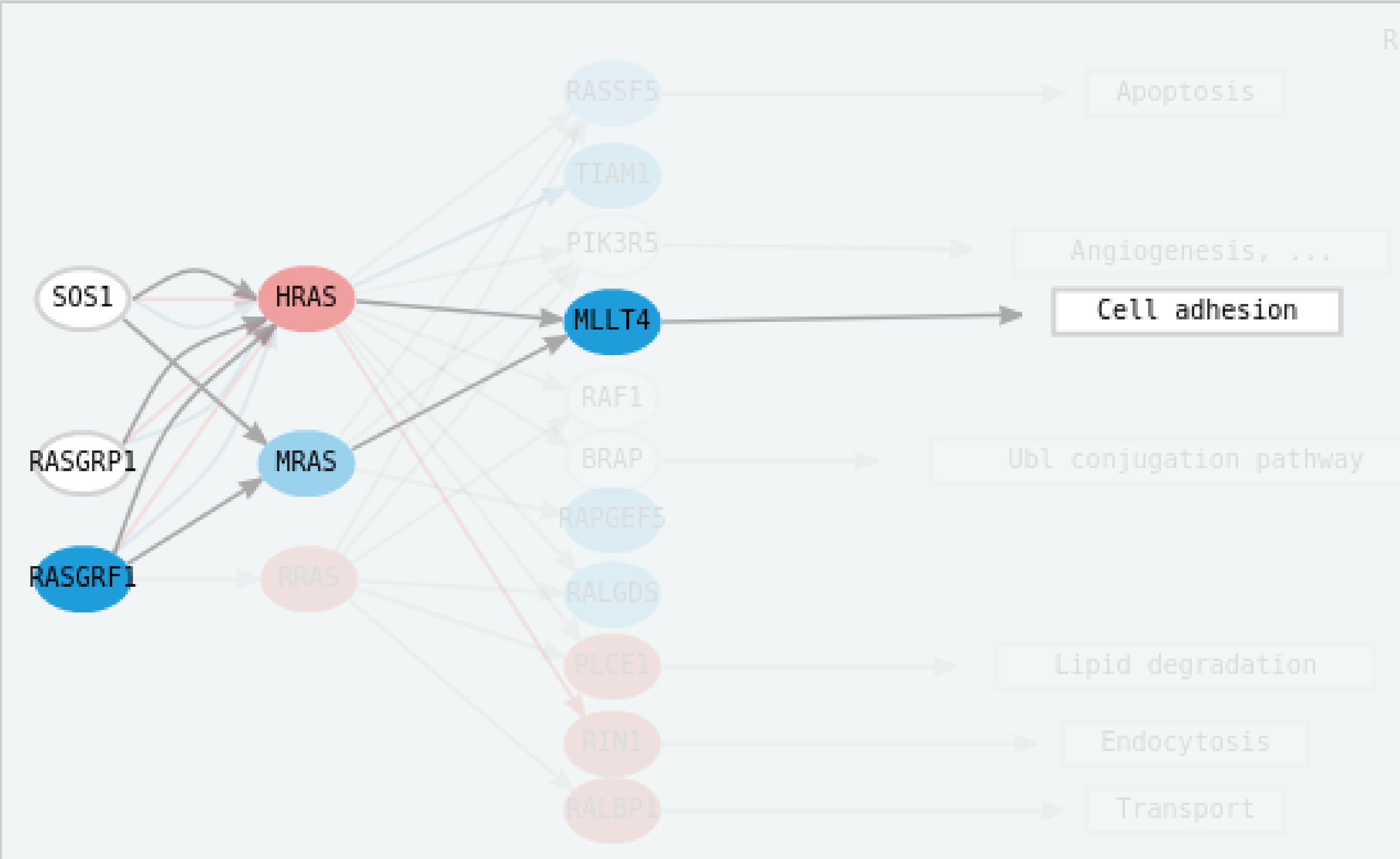

Ras signaling pathway (hsa04014)

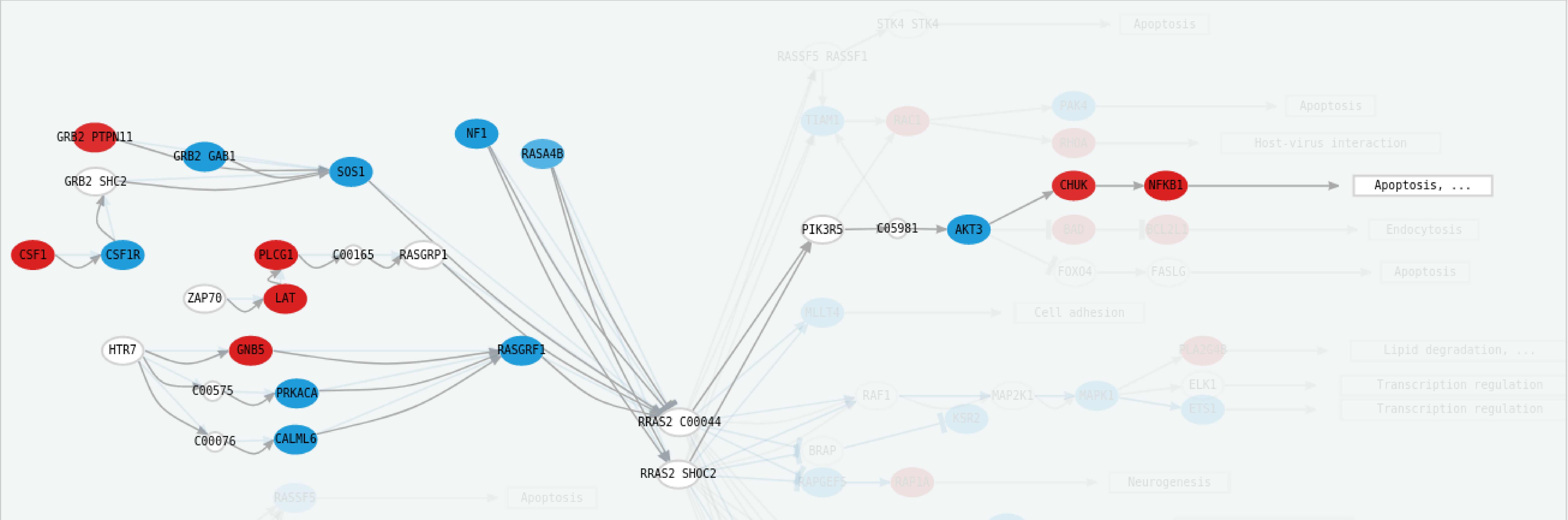

Ras signaling pathway (hsa04014)

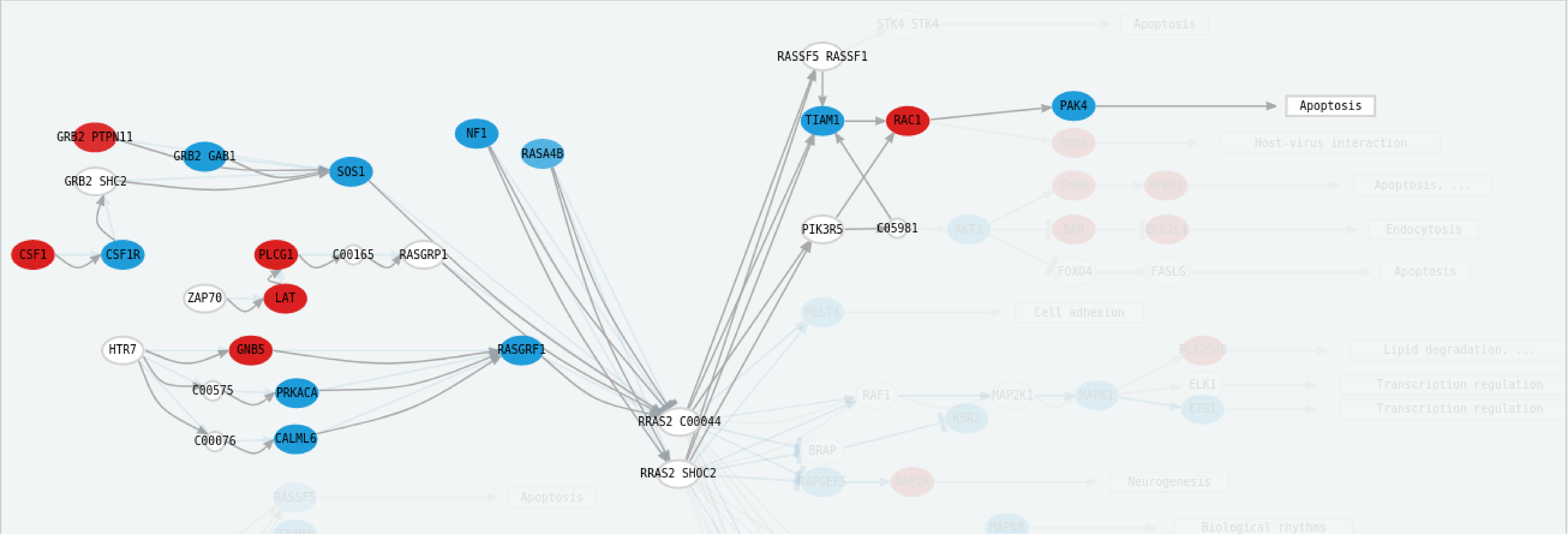

Ras signaling pathway (hsa04014)

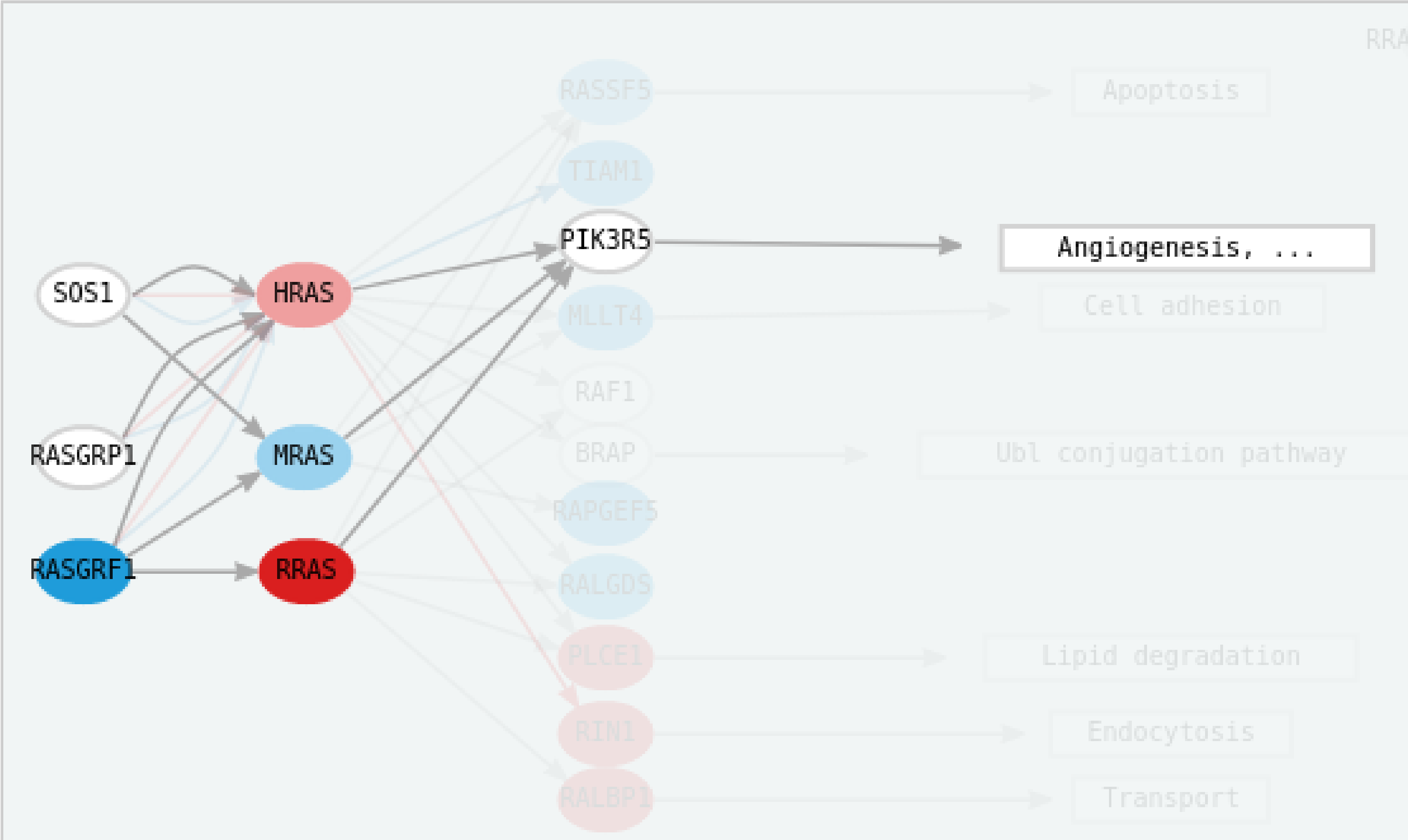

Ras signaling pathway (hsa04014)

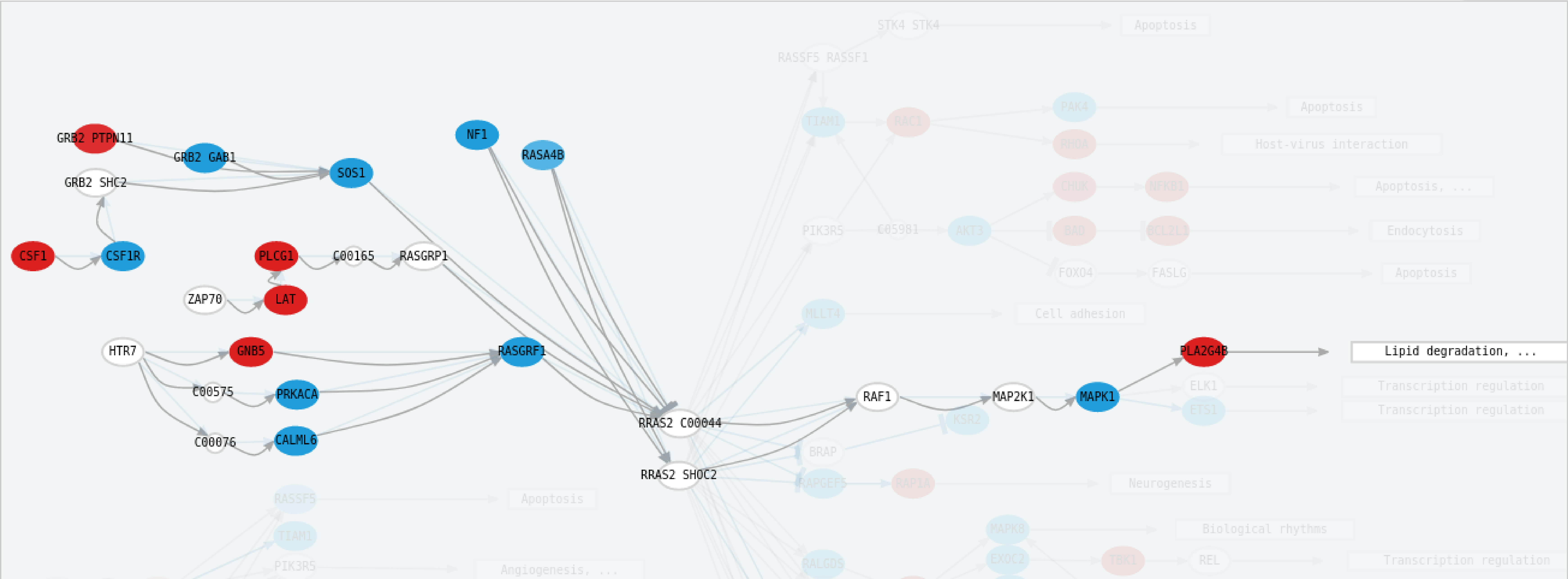

Ras signaling pathway (hsa04014)

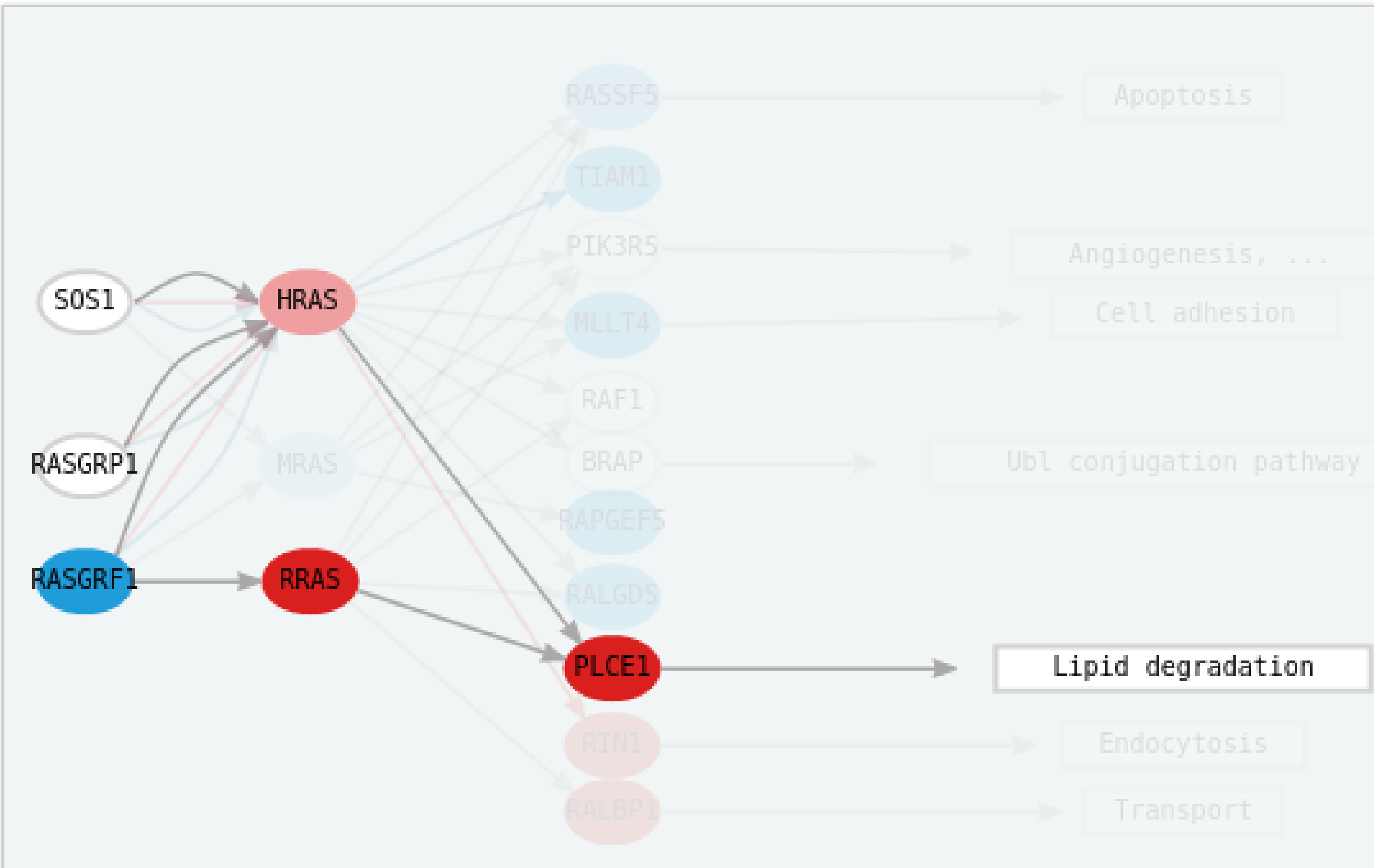

Ras signaling pathway (hsa04014)

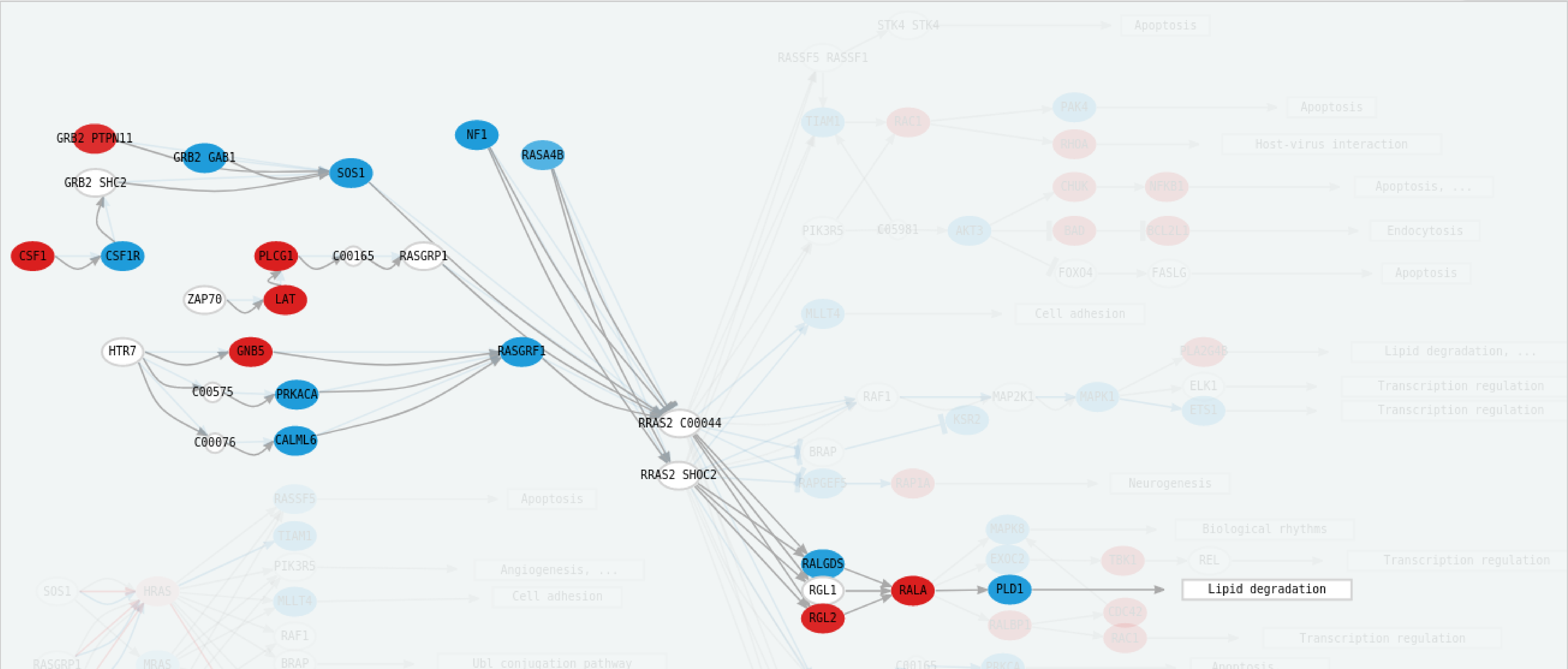

Ras signaling pathway (hsa04014)

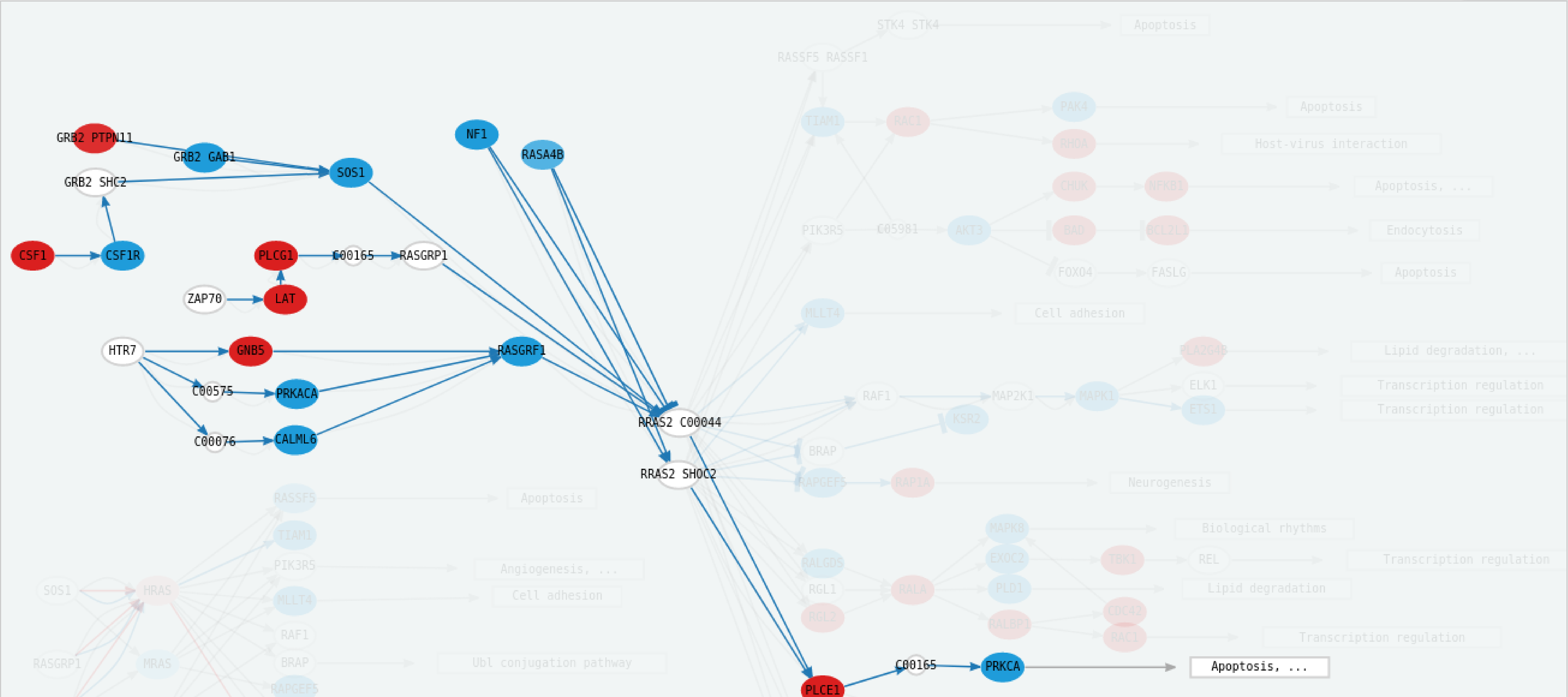

Ras signaling pathway (hsa04014)

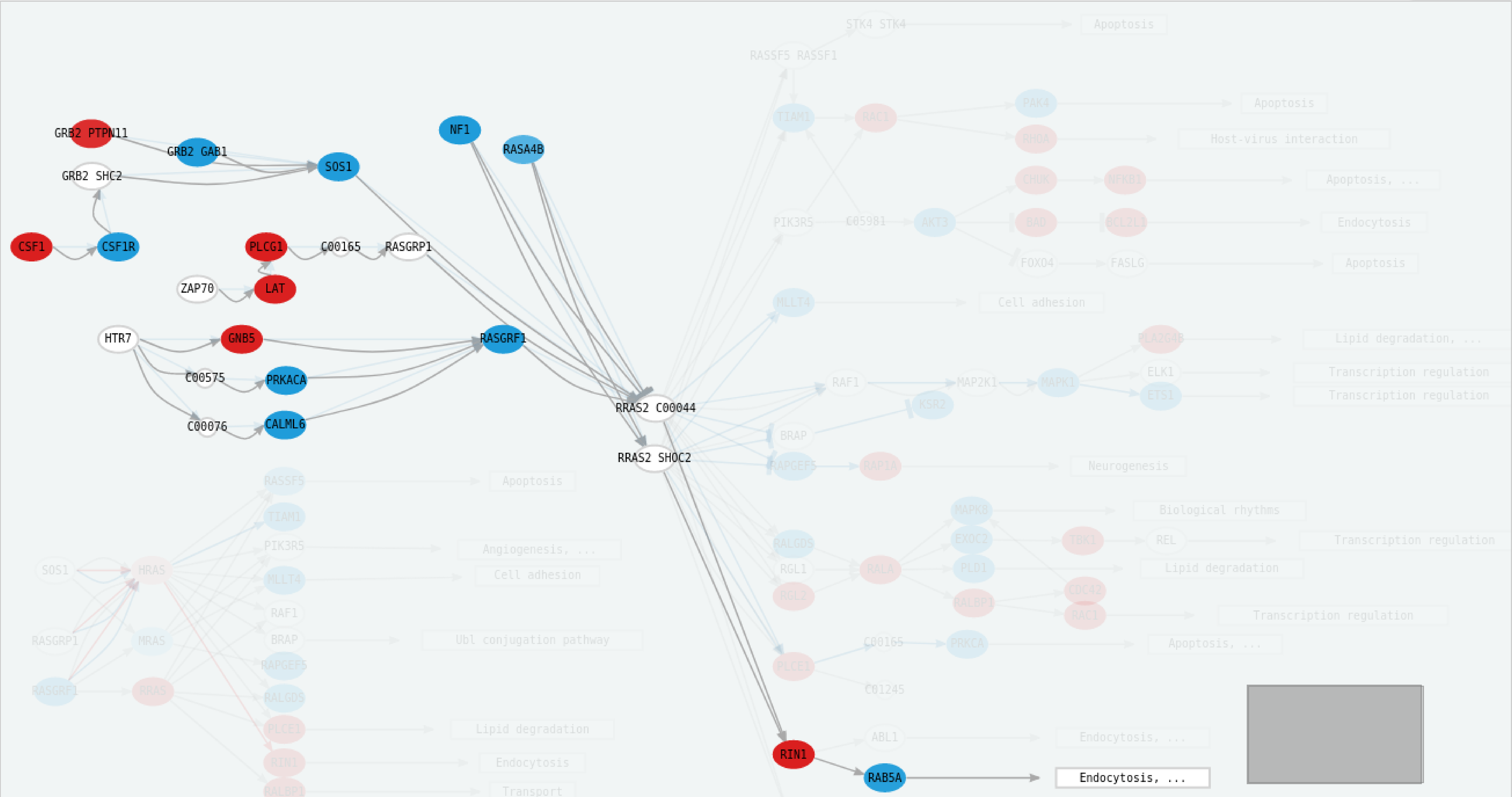

Ras signaling pathway (hsa04014)

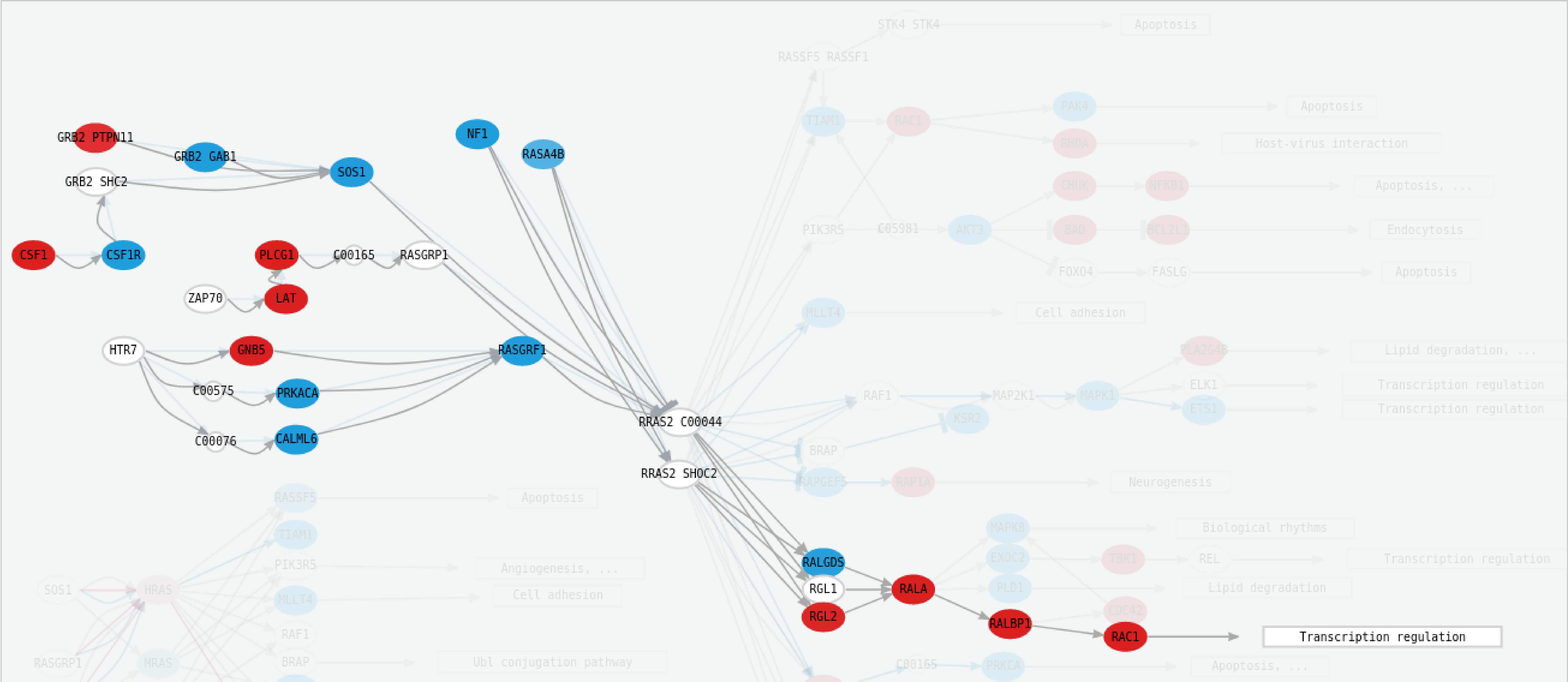

Ras signaling pathway (hsa04014)

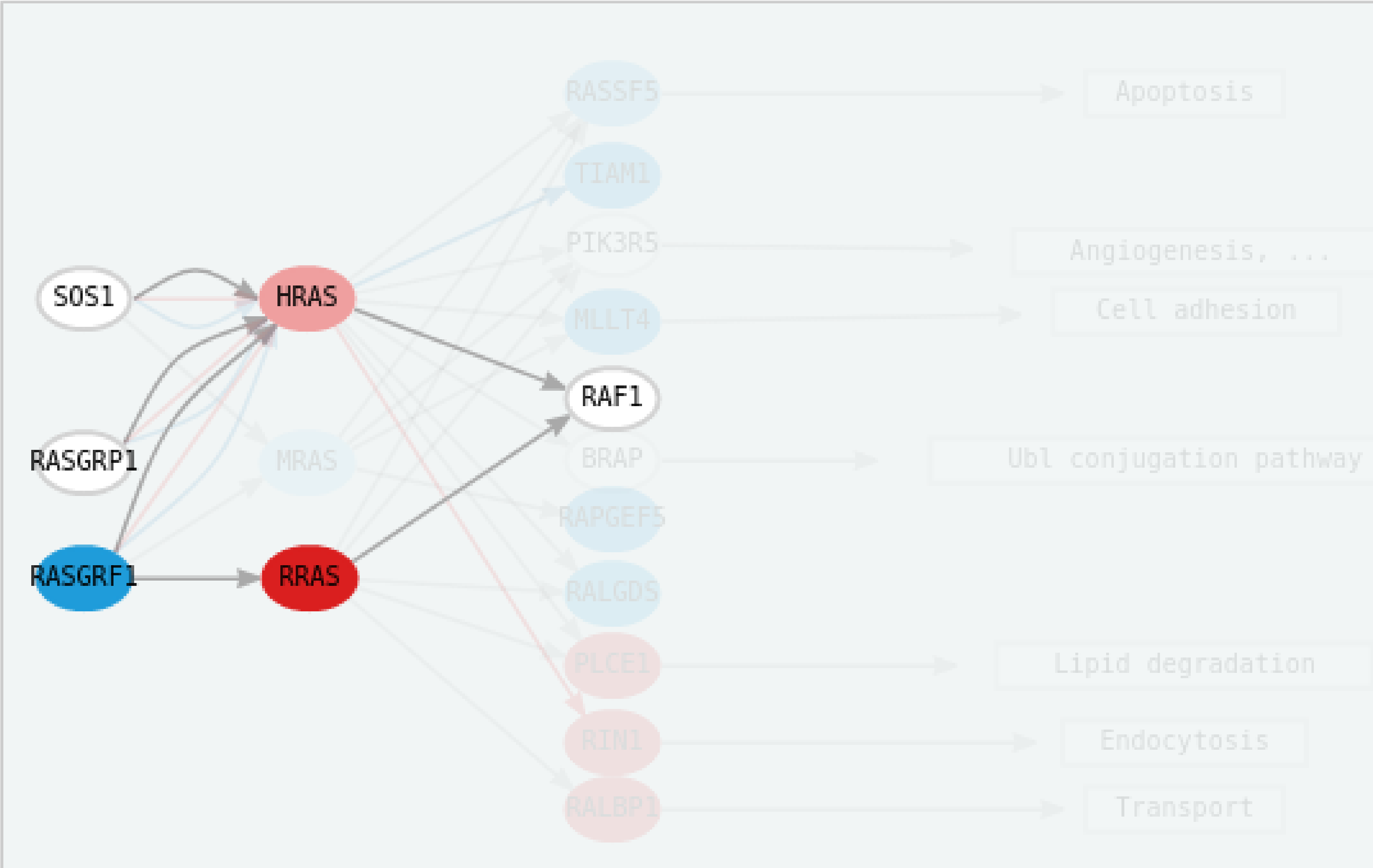

Ras signaling pathway (hsa04014)

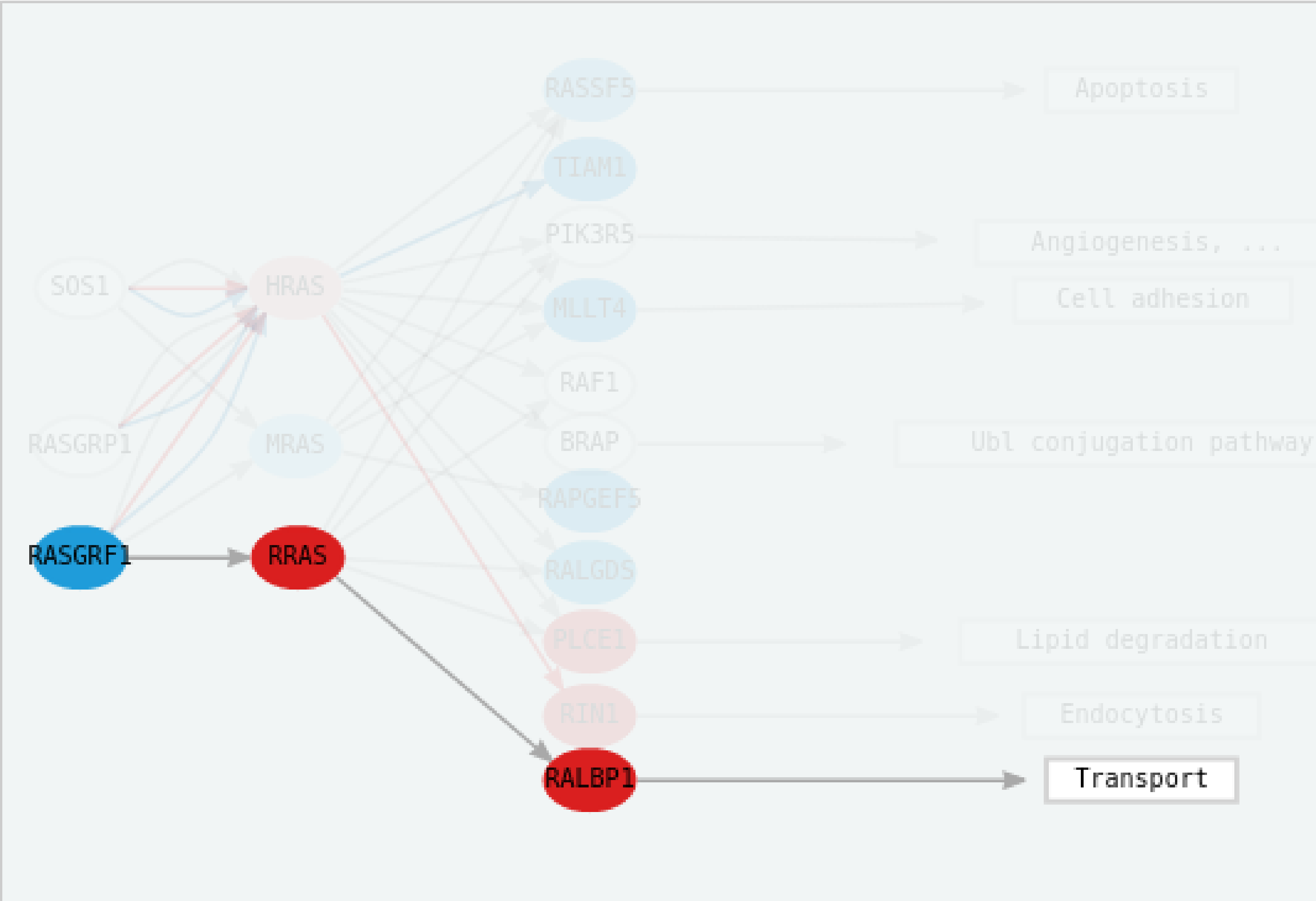

Ras signaling pathway (hsa04014)

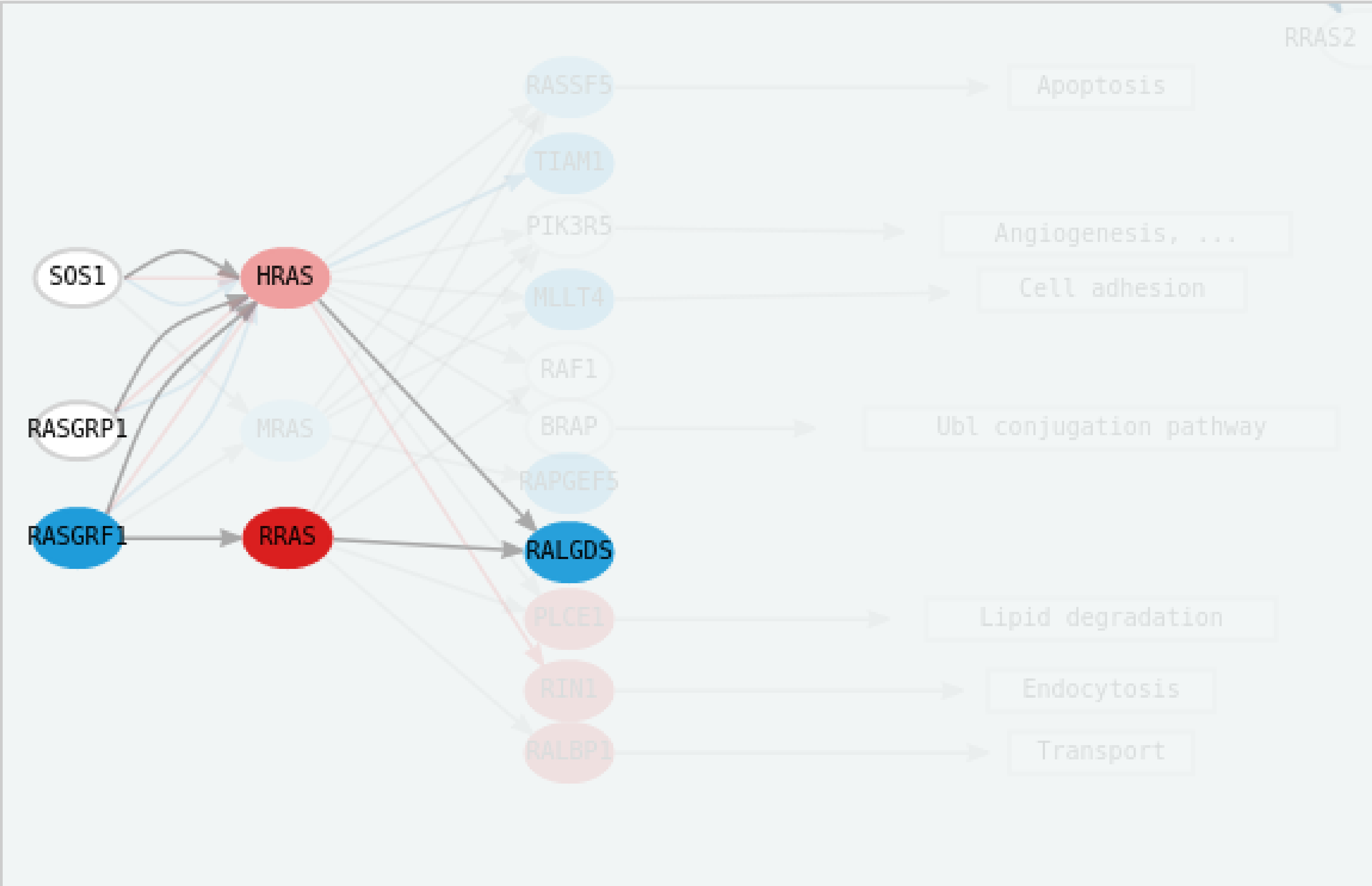

Ras signaling pathway (hsa04014)

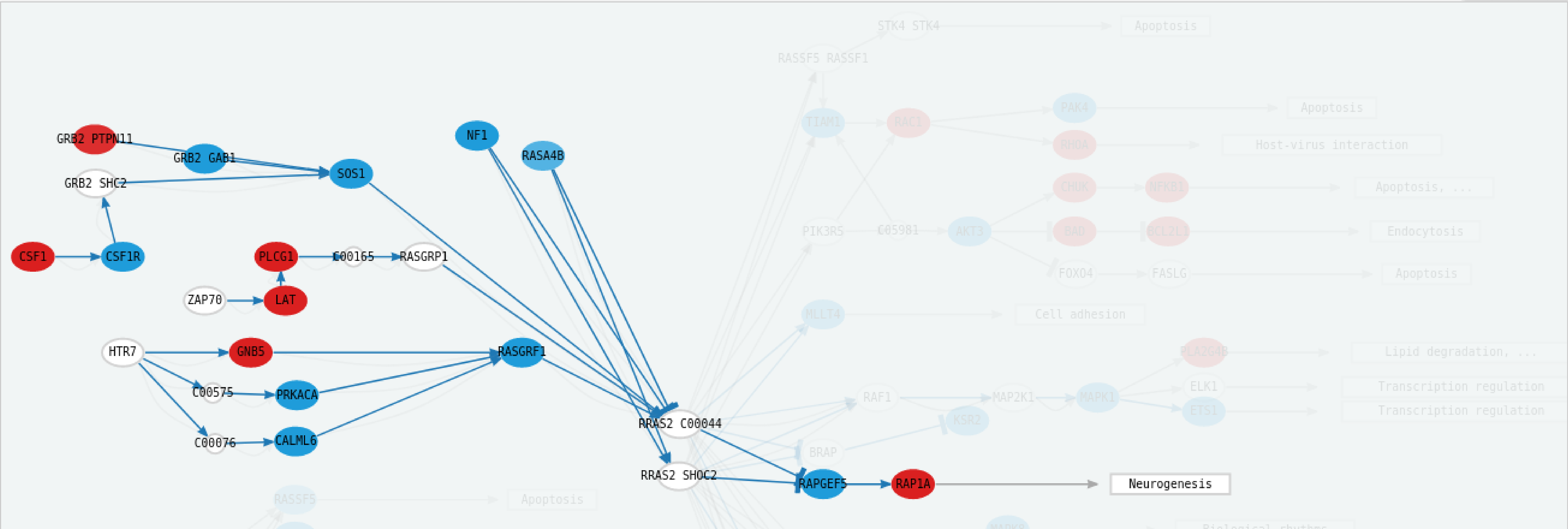

Ras signaling pathway (hsa04014)

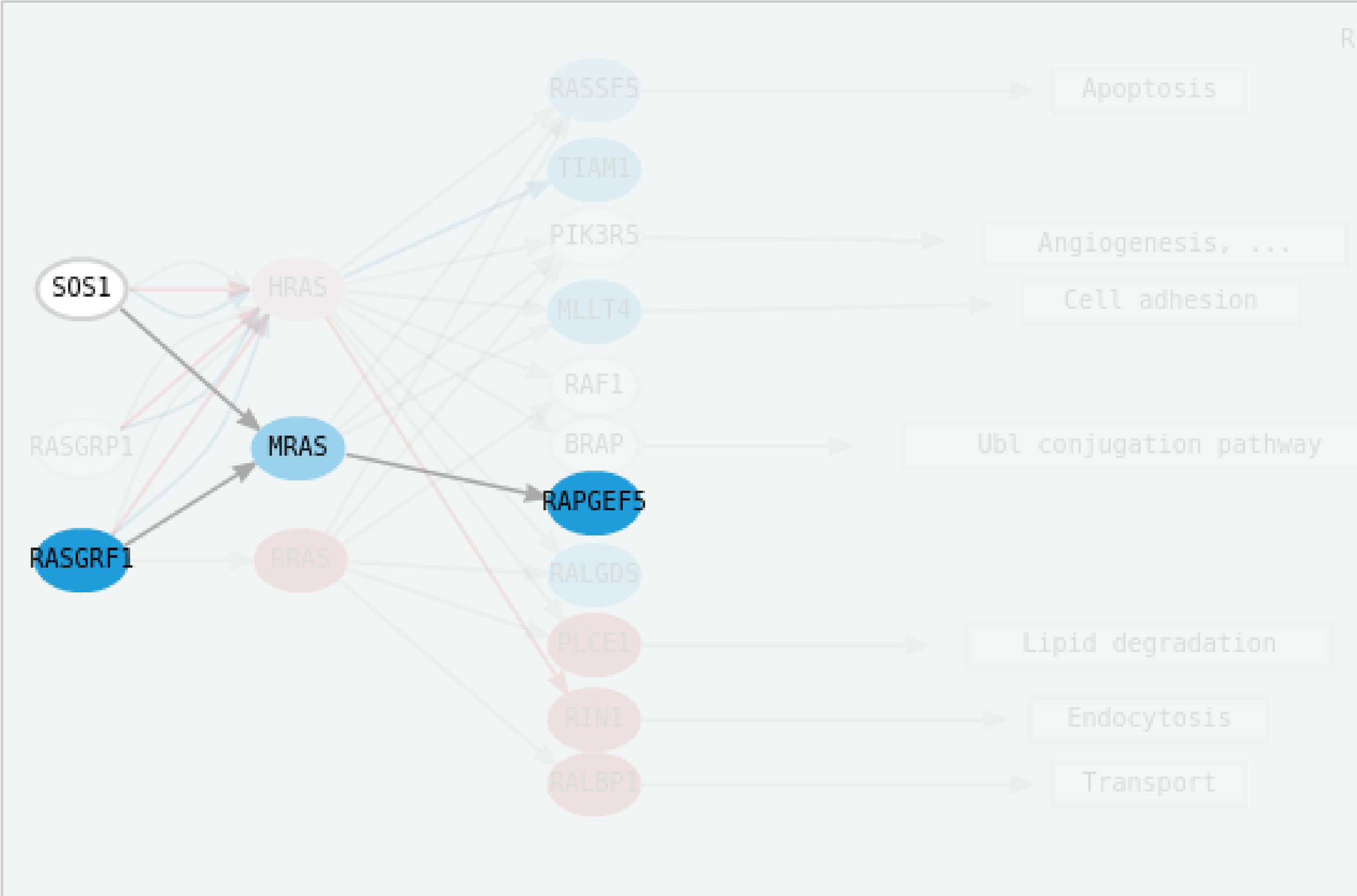

Ras signaling pathway (hsa04014)

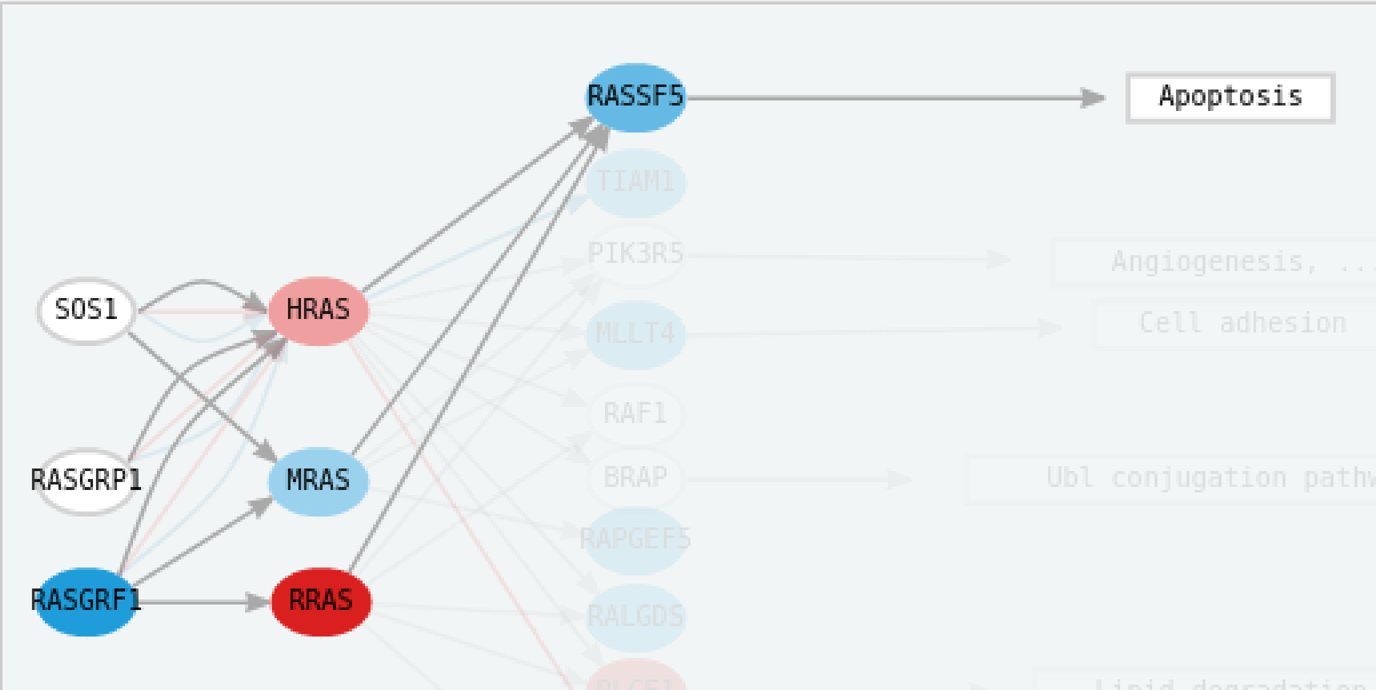

Ras signaling pathway (hsa04014)

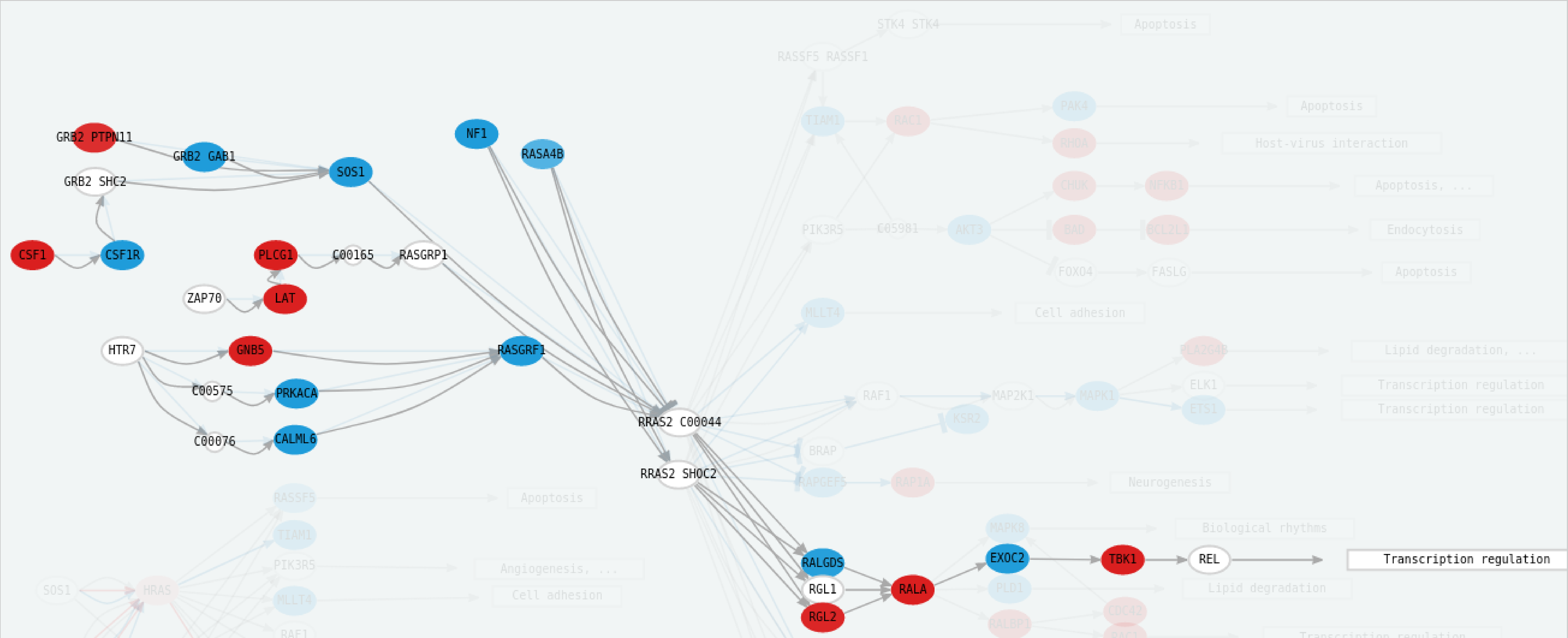

Ras signaling pathway (hsa04014)

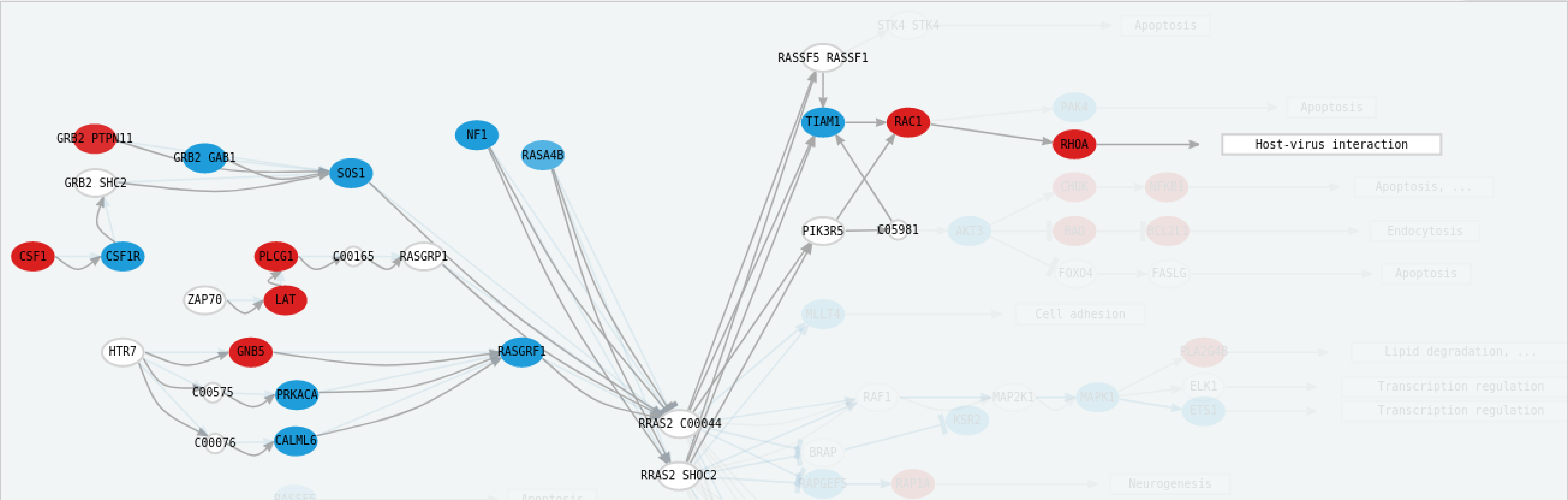

Ras signaling pathway (hsa04014)

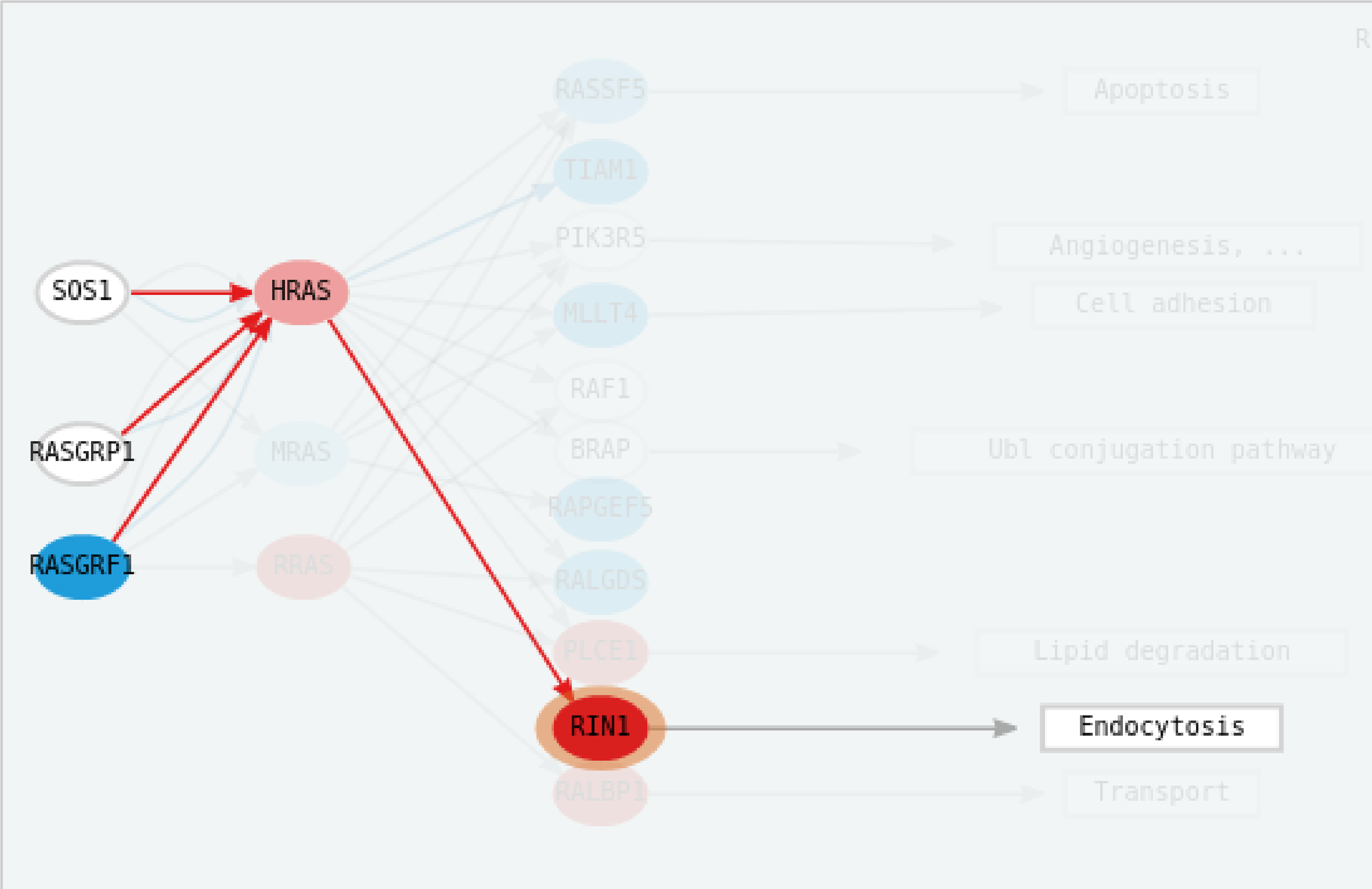

Ras signaling pathway (hsa04014)

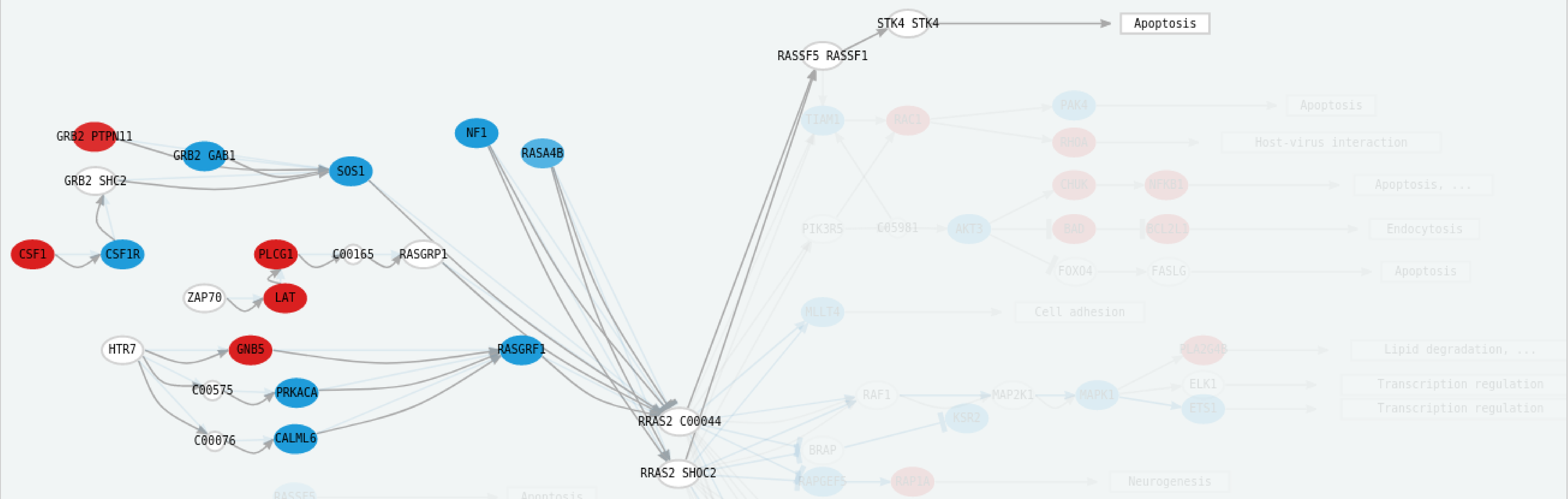

Ras signaling pathway (hsa04014)

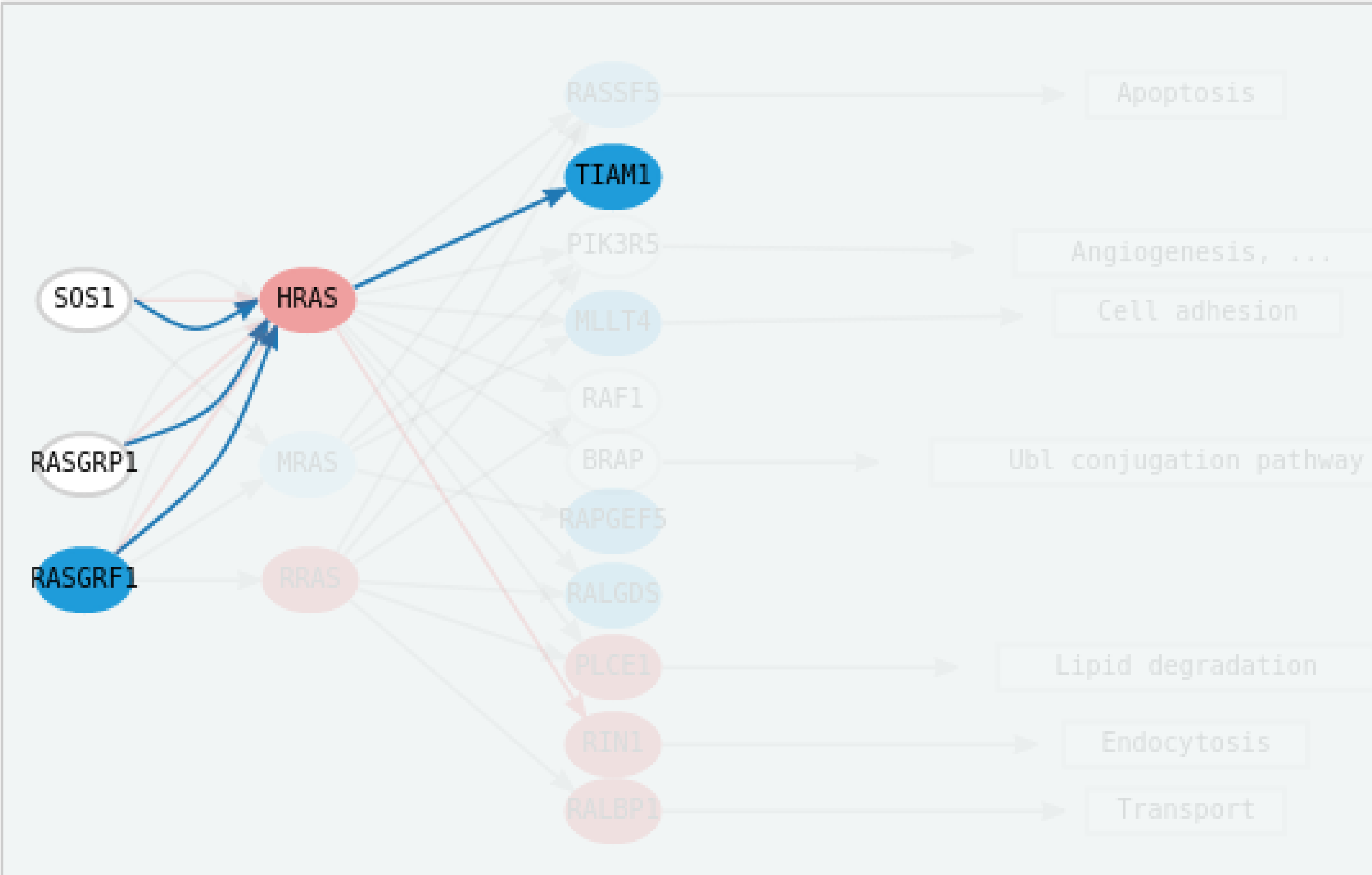

VEGF signaling pathway (hsa04370)

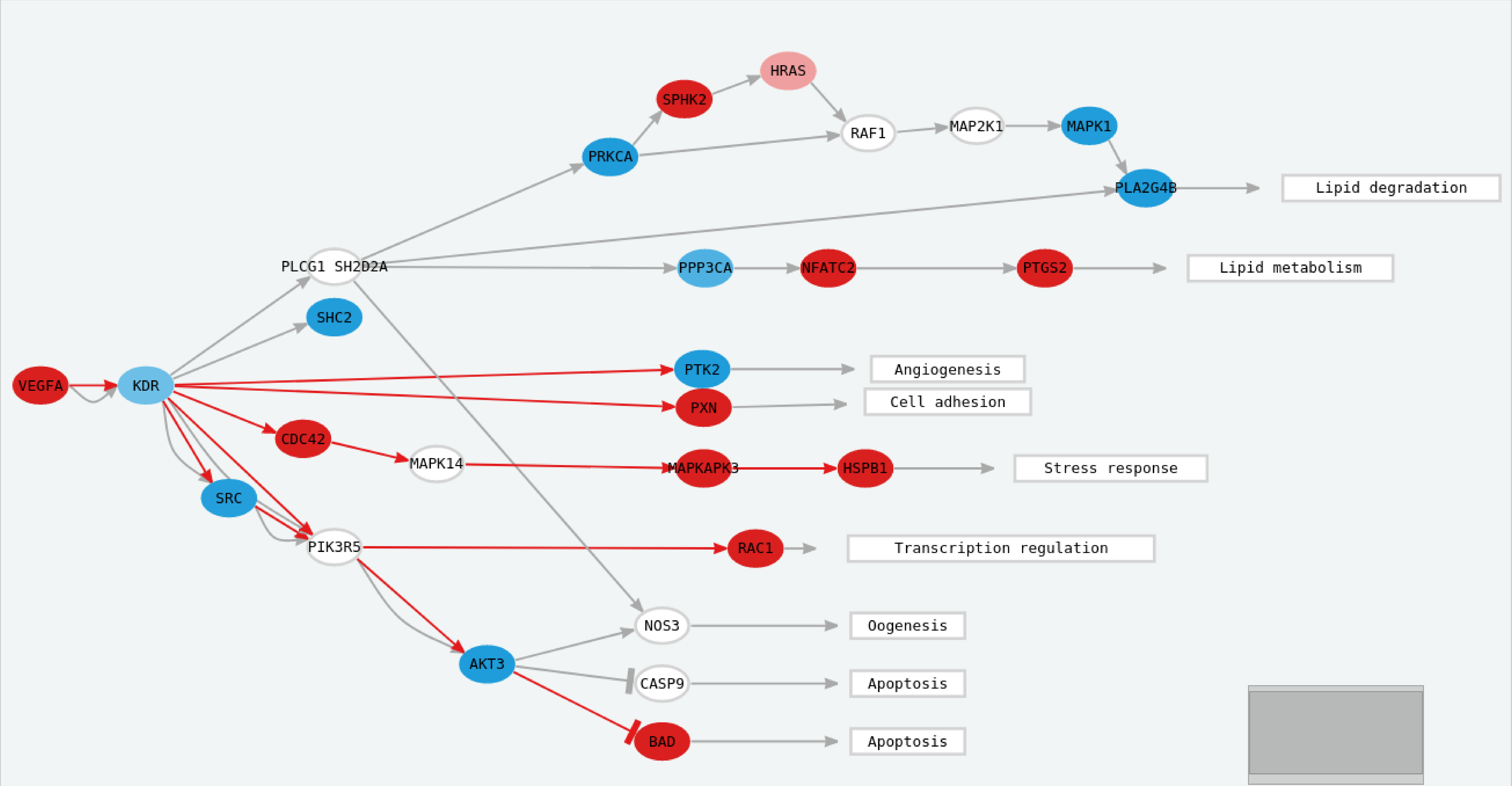

VEGF signaling pathway (hsa04370)

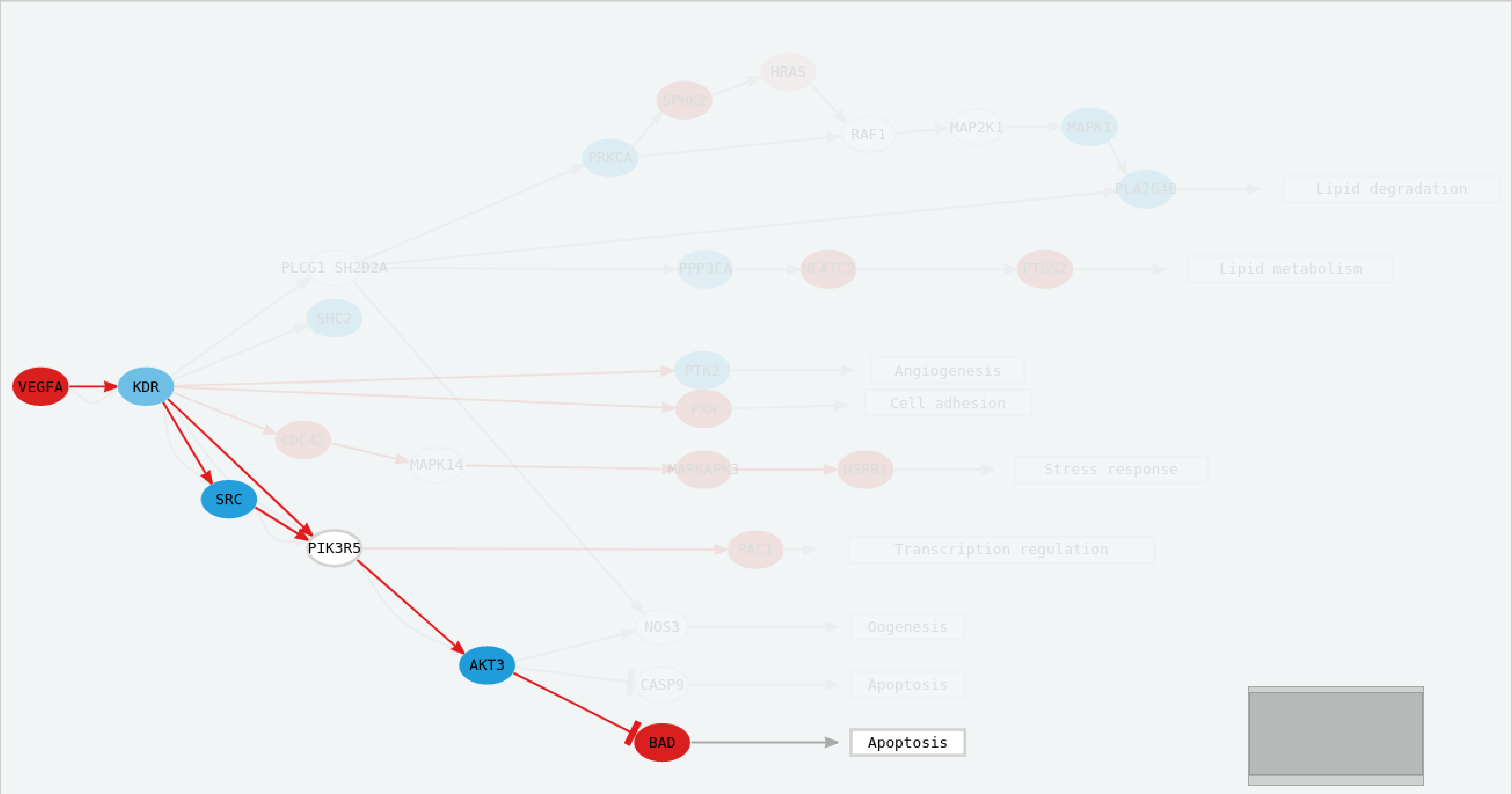

VEGF signaling pathway (hsa04370)

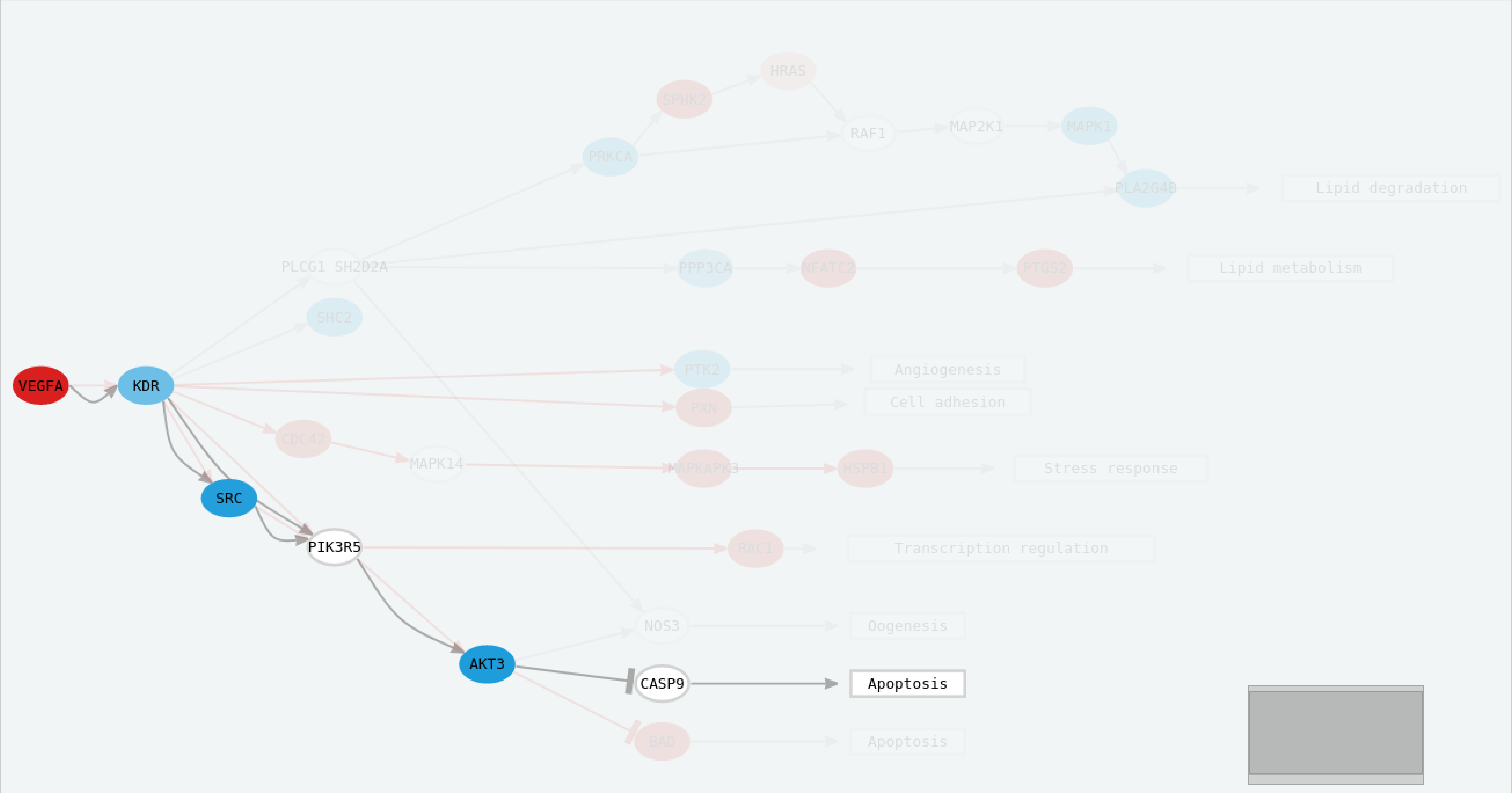

VEGF signaling pathway (hsa04370)

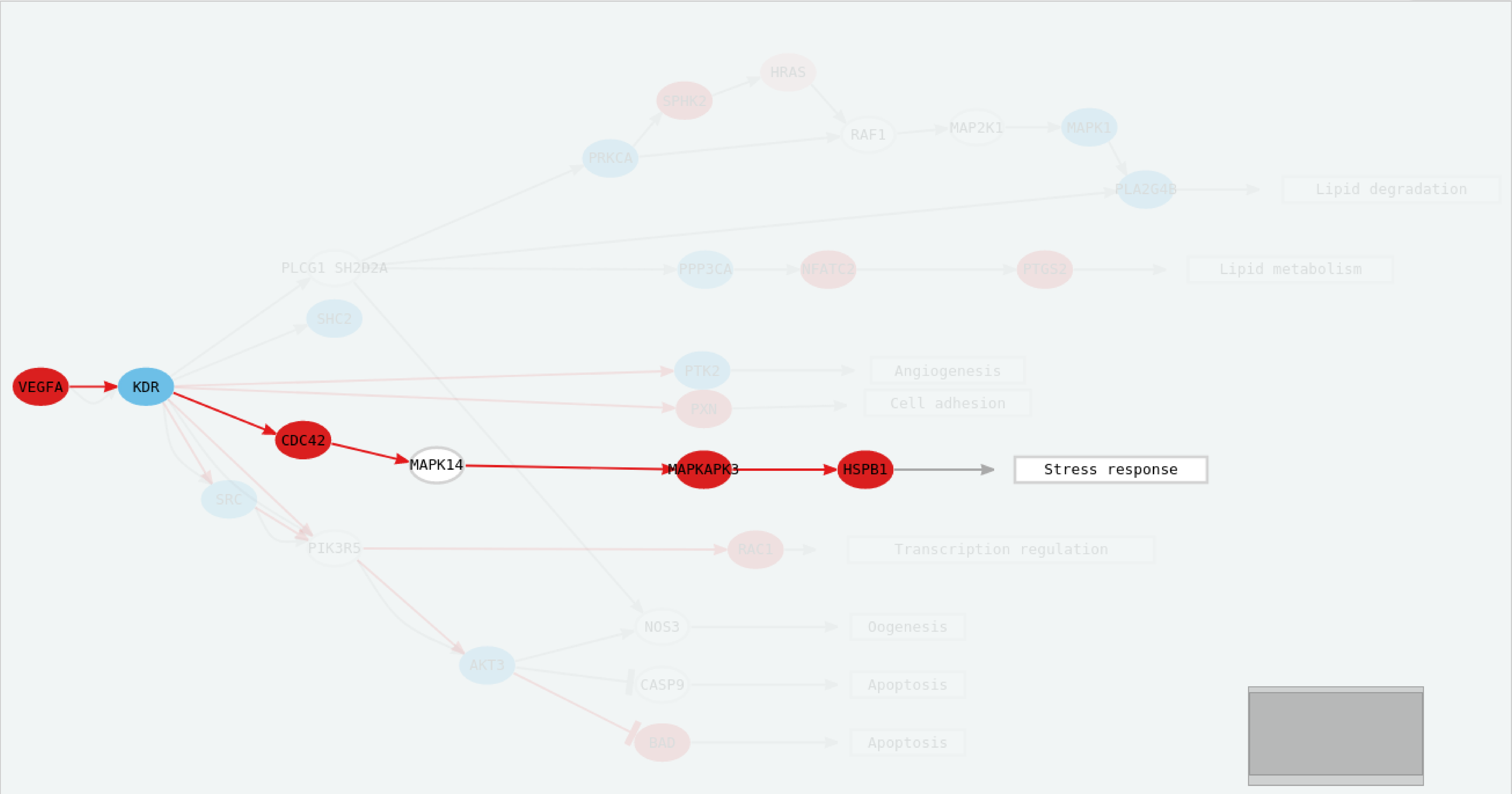

VEGF signaling pathway (hsa04370)

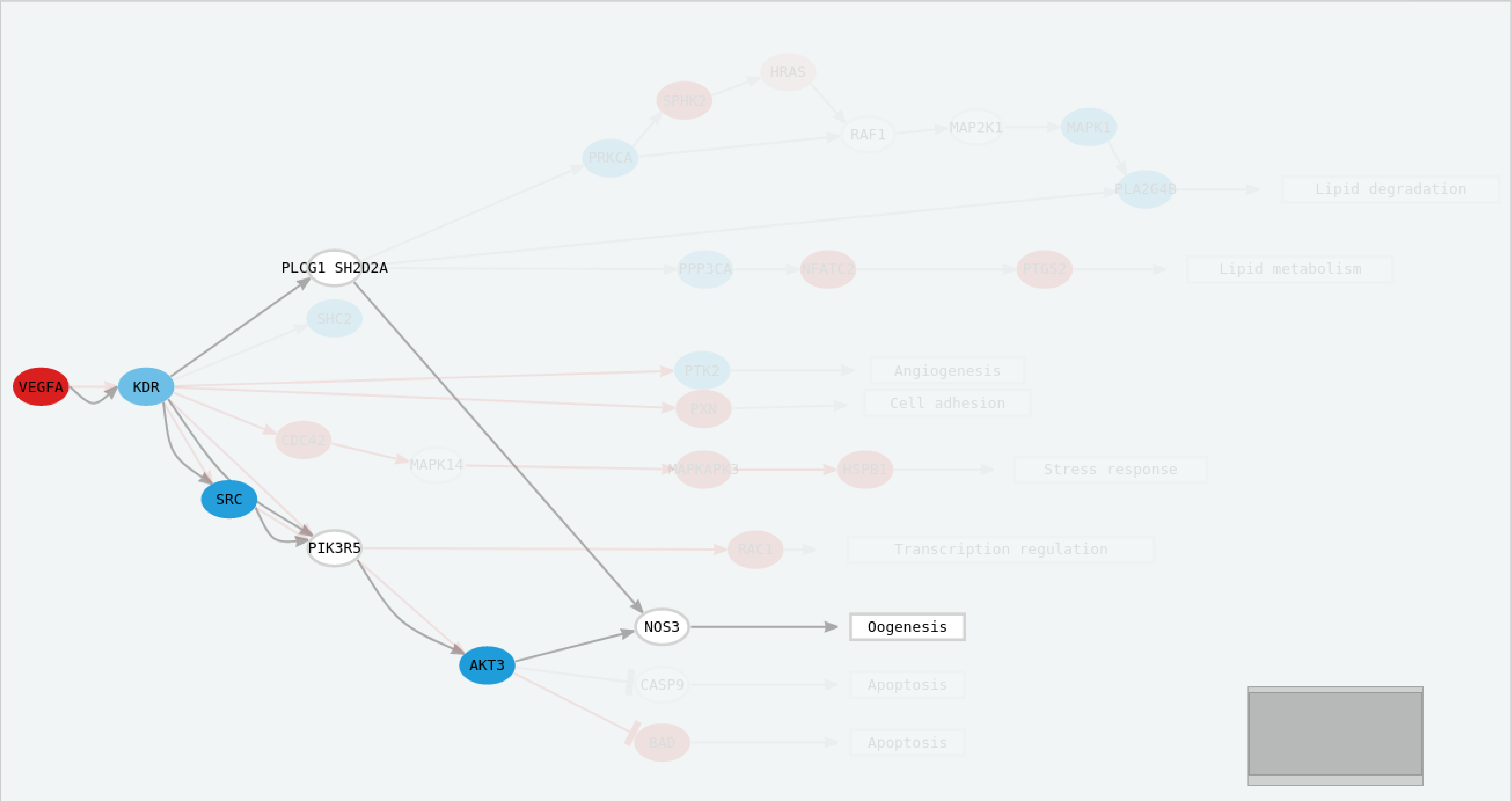

VEGF signaling pathway (hsa04370)

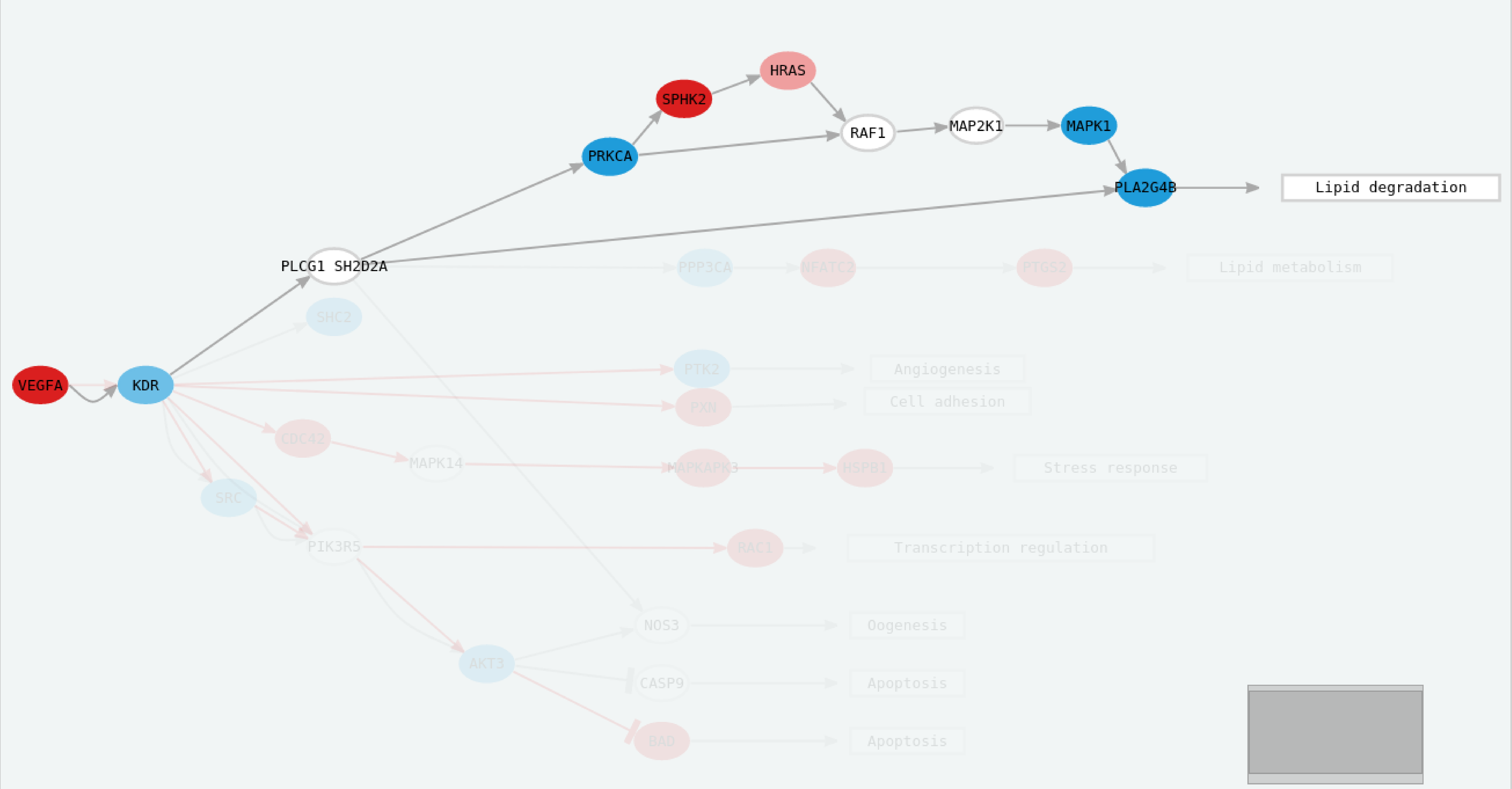

VEGF signaling pathway (hsa04370)

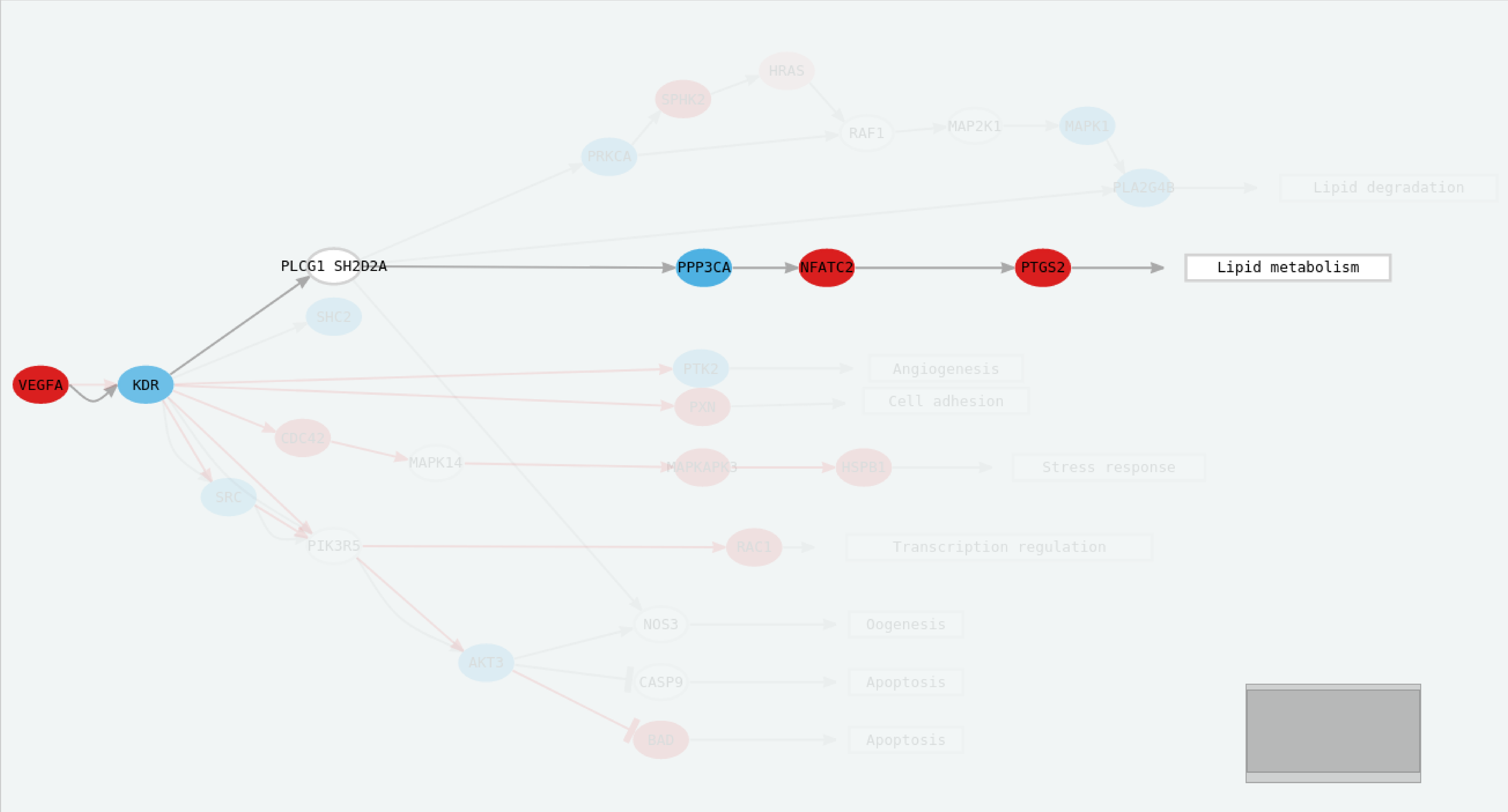

VEGF signaling pathway (hsa04370)

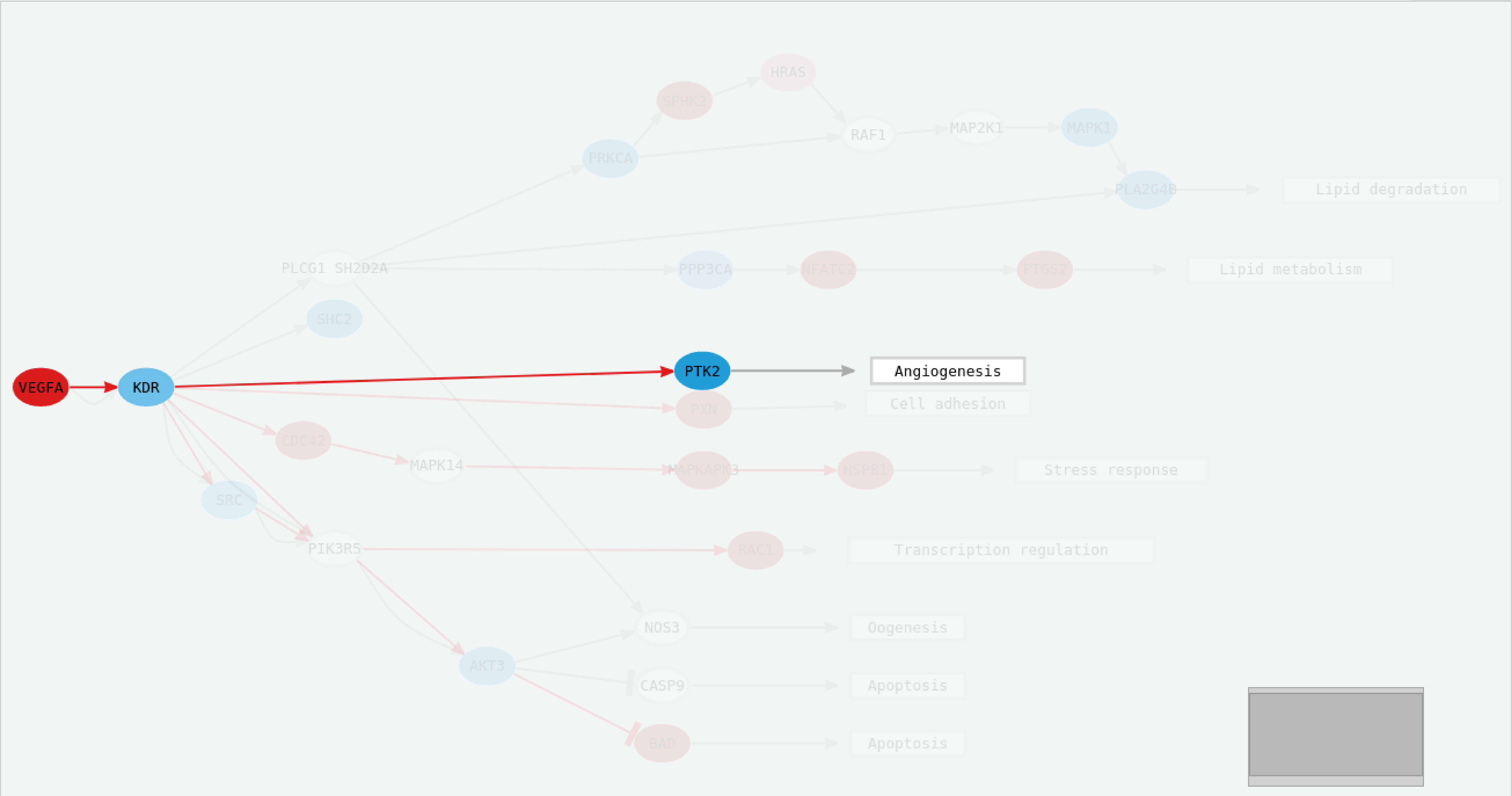

VEGF signaling pathway (hsa04370)

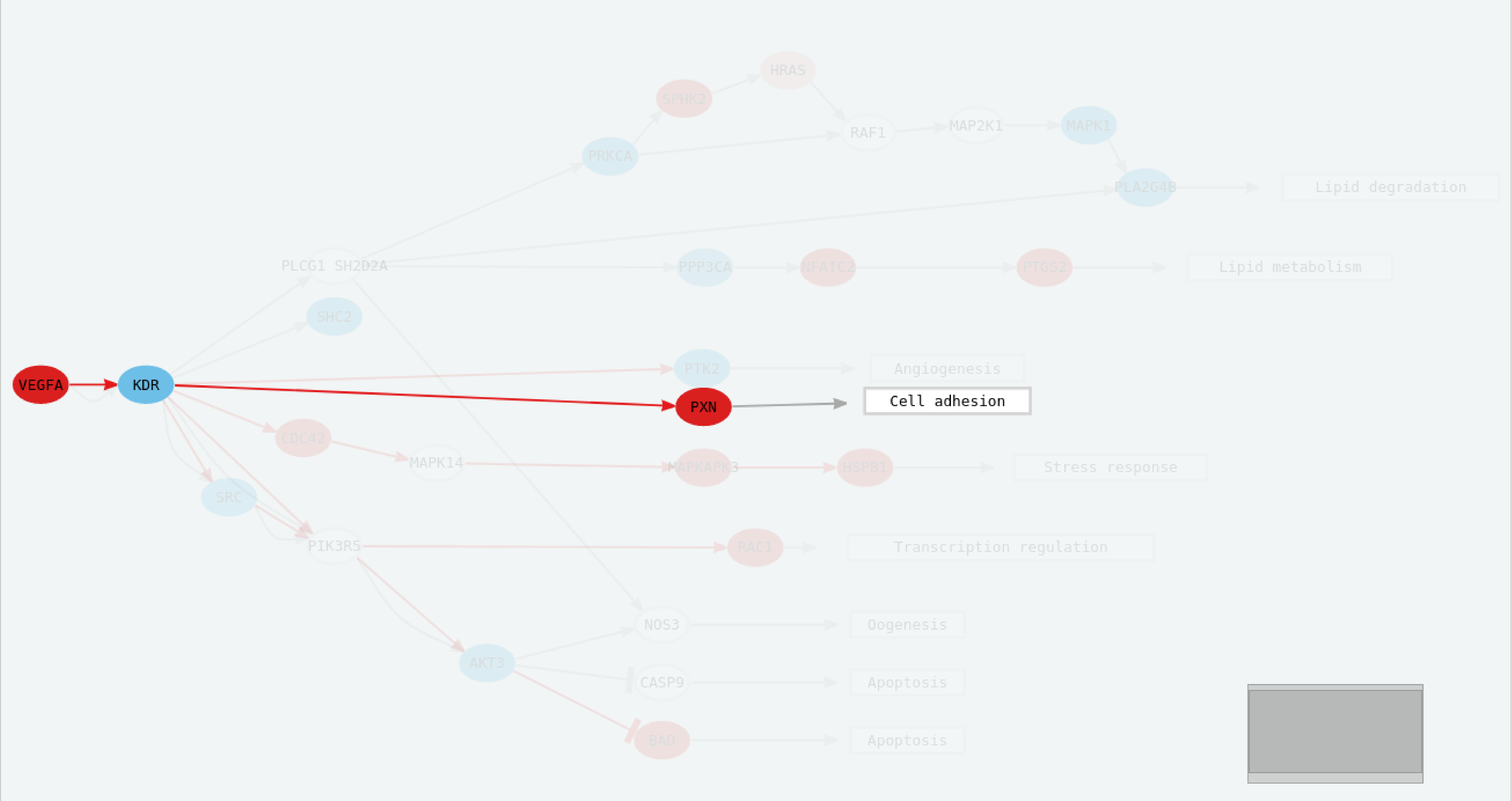

VEGF signaling pathway (hsa04370)

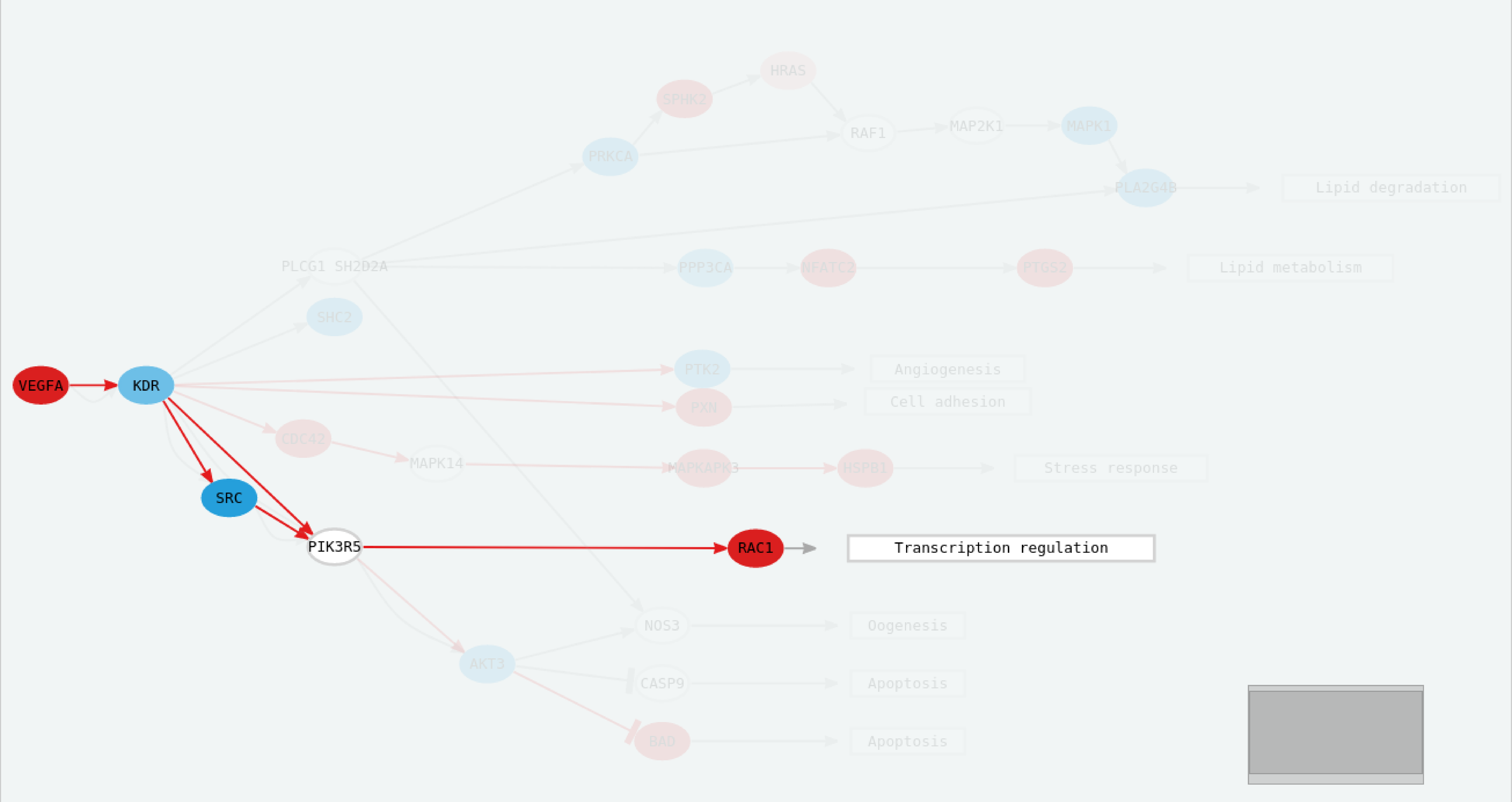

VEGF signaling pathway (hsa04370)

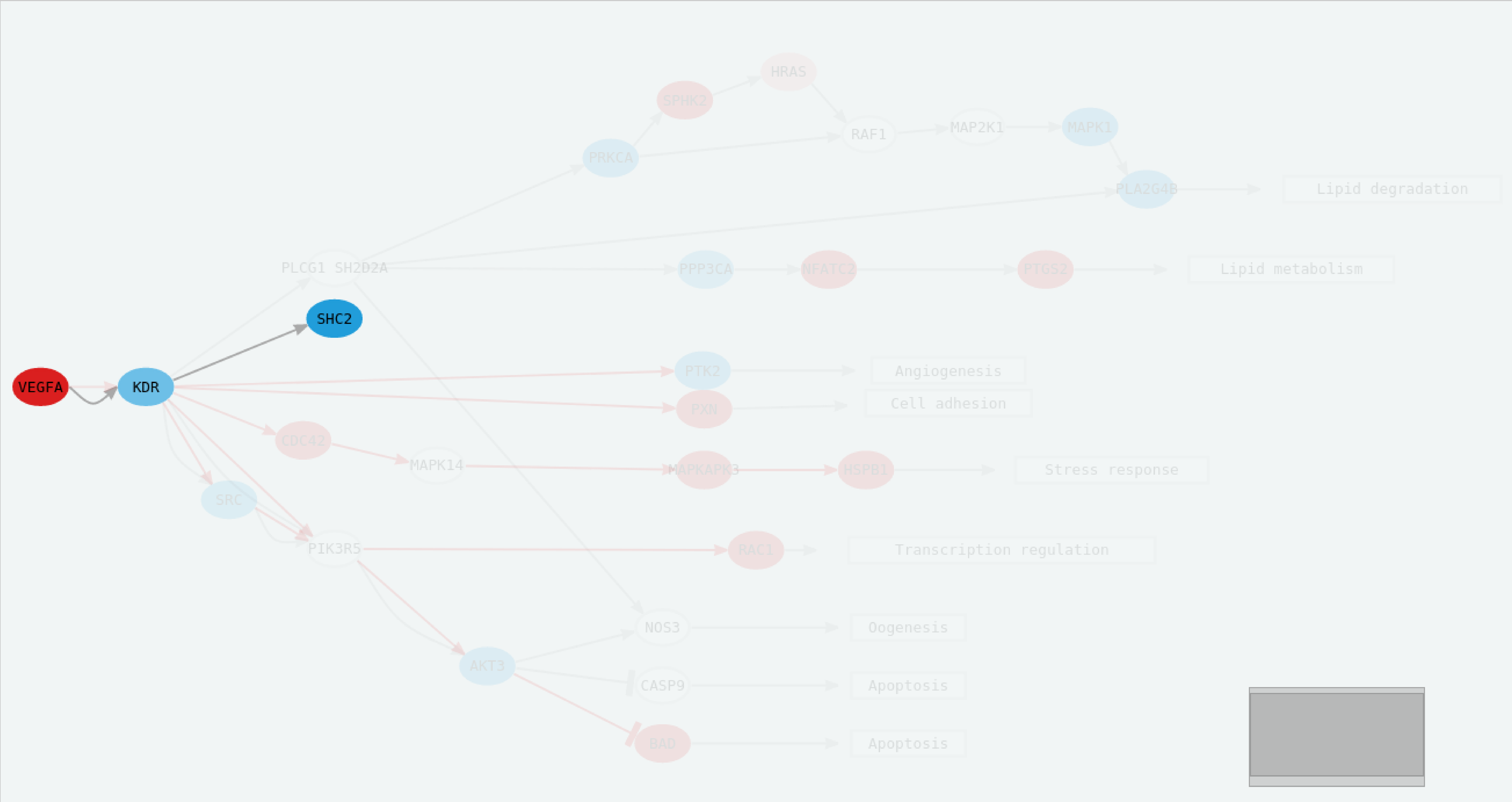

Supplement: zcaa011_Supplemental_Files [file narcancer_2_2_zcaa011_s5.zip › Supplementary Figures 1-5.pdf]
